# A Framework for Analyzing the Whole Body Surface Area from a Single View

**DOI:** 10.1371/journal.pone.0166749

**Published:** 2017-01-03

**Authors:** Marco Piccirilli, Gianfranco Doretto, Donald Adjeroh

**Affiliations:** Lane Department of Computer Science and Electrical Engineering, West Virginia University, Morgantown, WV 26506, United States of America; Universitat Rovira i Virgili, SPAIN

## Abstract

We present a virtual reality (VR) framework for the analysis of whole human body surface area. Usual methods for determining the whole body surface area (WBSA) are based on well known formulae, characterized by large errors when the subject is obese, or belongs to certain subgroups. For these situations, we believe that a computer vision approach can overcome these problems and provide a better estimate of this important body indicator. Unfortunately, using machine learning techniques to design a computer vision system able to provide a new body indicator that goes beyond the use of only body weight and height, entails a long and expensive data acquisition process. A more viable solution is to use a dataset composed of virtual subjects. Generating a virtual dataset allowed us to build a population with different characteristics (obese, underweight, age, gender). However, synthetic data might differ from a real scenario, typical of the physician’s clinic. For this reason we develop a new virtual environment to facilitate the analysis of human subjects in 3D. This framework can simulate the acquisition process of a real camera, making it easy to analyze and to create training data for machine learning algorithms. With this virtual environment, we can easily simulate the real setup of a clinic, where a subject is standing in front of a camera, or may assume a different pose with respect to the camera. We use this newly designated environment to analyze the whole body surface area (WBSA). In particular, we show that we can obtain accurate WBSA estimations with just one view, virtually enabling the possibility to use inexpensive depth sensors (e.g., the Kinect) for large scale quantification of the WBSA from a single view 3D map.

## 1 Introduction

Accurate determination of the whole body surface area (WBSA) is a topic that has been actively studied over the last century. Here, we use WBSA (as opposed to BSA) to emphasize the fact that we aim at the accurate estimation of the whole area of the body. From the initial estimate of Du Bois and Du Bois in 1916 [1], to recent work [2], and despite many critiques [3], the WBSA has attracted a lot of attention, driven primarily by the large variety of its applications. For many clinical purposes, the WBSA is a better indicator of metabolic mass than body weight since it is less affected by abnormal adipose mass [4]. WBSA is used in medecine and pharmacology to estimate drug dosage rates [1], since it is proportional to the drug absorption rate [5], WBSA and to determine strategies for anticancer drugs and radiation dose estimation [6, 7]. WBSA has been used as a normalizing factor for the glomerular filtration rate (GFR) [8], [4], and to quantify skin burn areas [9–11]. In [2, 8] the WBSA was used to account for different body sizes in patients with aortic stenosis. Here, the aortic valve area (AVA) is divided by the body surface area to calculate indexed AVA (AVAindex). Other areas where the BSA is often used include: plastic surgery [12], fashion industry [13], and in ergonomic design [14].

Certain health status indicators, such as obesity, are important risk factors for a range of cardiovascular diseases, as well as early death. Thus, there is a medical interest in a reliable, cheap and easy way to monitor health risks by turning physiological observations into quantitative indices. Besides body mass index (BMI), the primary measure of obesity [15, 16], the WBSA can have an equally significant role in determining health status. However, the WBSA is the measure of the surface area, in contrast with BMI, which is a composite attribute. This fact is fundamental, since the WBSA can be estimated more accurately with computer vision techniques, than with weight and stature. The WBSA can easily overcome the usual problems with BMI, namely, the inability to capture the distribution of body mass, and inability to distinguish between lean and fatty mass.

Historically, the only easy way to get this measure (WBSA) is through some empirical formulae that consider just two human body parameters (body weight and stature). The large variety of body shapes, body compositions, and races makes the use of a fixed formula highly questionable. Thus there has been a continuous stream of efforts to accommodate different individuals [11, 17–20]. Another recent approach is to use direct measurements from a three dimensional (3D) whole body scanner. The problem is that, such scanners are typically very expensive, costing hundreds of thousands of dollars, and have to be used by trained personnel, thus limiting their availability to users.

### 1.1 WBSA: Measurements and estimation

The common methods for WBSA calculation are through some well known formulae. The most widely used formula for WBSA calculation is the one devised by Du Bois and Du Bois in 1916 [1]. Moulds of plaster of Paris for 9 subjects were cut into small pieces in an attempt to measure the two-dimensional surface area of the skin. Each individual’s body/skin surface area was then calculated and Du Bois and Du Bois determined that WBSA was related to stature and weight by the formula: *WBSA* = 0.007184 × *W*^0.425^ × *H*^0.725^ [1], where W and H are the weight (in kg) and stature (in cm) of the subject. Notably, this formula was derived from 9 subjects only, one of whom was a child. Since the bodies of the subjects studied in the middle of the First World War are unlikely to be similar to the patients of the modern society, Mosteller proposed a new calculation of WBSA in 1987 [21].

Today there are many studies related to the verification of meaningful differences between WBSA measurements taken using a whole body three-dimensional (3D) scanner (criterion measure) and the estimates derived from each WBSA equation identified from systematic reviews [22], [23], [20], [24], [25]. In these studies, the 3D scanners used are often cumbersome and slow, and have to be operated by specially trained personnel. The formulae are still in use, but many corrective factors are appearing to adapt the formulas to today’s special cases (e.g. very obese people) [17–19], or race [11], [20]. Verbraecken et al [17] examined the WBSA based on Mosteller’s formula in normal-weight (BMI, 20–24.9 kg/m), overweight (BMI, 25–29.9 kg/m), and obese (BMI, >30 kg/m) adults (>18 years old) in comparison with other empirically derived formulas. With obesity, weight increases without a proportional increase in stature. Consequently, it is possible that the WBSA-predicting equations, which include stature coefficients, could systematically miscalculate WBSA for obese patients. Because many clinically important measurements are indexed to WBSA, systematic errors in WBSA estimation can adversely affect the clinical care of obese patients. Similarly, the work in [18] and [19] showed that the well known WBSA formulae (DuBois and Dubois) fail to predict the WBSA at the extreme of the normal weight range (10–80 kg). Different scenarios are analyzed in [11, 20, 26] each requiring a different modification of the basic WBSA formula.

#### 1.1.1 Measurements using body scanner

An alternative to the use of WBSA formulae is whole-body 3D scanning. There are three major issues with the 3D laser scanners: cost, speed, and physical space requirement. Classic 3D laser scanners use a laser beam to illuminate the surface. At the same time a receptor registers the beam distortion on the surface and computes the respective depth. The beam needs to cover all the space of the surface and it takes time to do so. This requires that the object be almost immobile and small movements can cause errors in the reconstruction. Modern laser scanners are fast enough to avoid this distortion, but still require a large room to contain the device.

The result of the scanning operation is usually “raw” data in the form of a 3D (*x*, *y*, *z*) point cloud. To reconstruct the mesh surface from the raw data, a surface reconstruction algorithm has to be applied. Without the face information it is not possible to relate the vertices to a face and thus compute the area of the surface. The 3D data, after surface reconstruction, is completed by other information than (*x*, *y*, *z*) points. The reconstruction with triangles, for instance, fits many small triangles every 3 points of the cloud. Then the calculation of the whole body surface area is reduced to a simple summation of the areas of all the triangles composing the mesh. This solution, unfortunately, is not as reliable and efficient as it looks. Key challenges in 3D body scanning include occluded areas [24], body parts registration [17], [27], device complexity and portability. Yu et al [24] provide more detailed analysis on some of these problems.

#### 1.1.2 Depth Cameras

State-of-the-art depth cameras are getting smaller, more accurate, and cheaper instead. This class of devices is led by the well-known Microsoft Kinect for XBox [28]. This device permits to acquire 3D data with a simple home setting. A surprising result was reported by Weiss et al in [27]. With only one device in a home setting, they develop a system capable of reconstructing the 3D mesh using four views of the subject. This methodology avoided the use of cumbersome 3D scanners, but still has some limitation. Acquiring many different views of the subject reguires a robust registration process. Moreover, the registered views are used to fit a model, in this case, the SCAPE model [29], to build the parametrized body model. This process, unfortunately, is still computationally intensive. The method in [27] requires almost 1 hour to reconstruct the body model. A solution adopted by some, is the use of multiple data sources: RGB, depth maps, pressure sensors, etc., designing a tailored solution for the specific task. In [30], Palmero et al presented an automatic sleep system recommendation using RGB, depth and pressure information. Unfortunately, this kind of system, without the use of a body model can be heavily affected by the pose of the subject, and the soft tissue deformation with the pose. Recently, Loper et al [31] introduced an innovative inverse rendering framework able to speedup the registration process, taking advantage of the modern GPU architecture. The method is significantly faster than [27] with almost the same accuracy, but more prone to errors in the differentiation process if the environment is not well constrained. A challenging problem for the 3D scanner methods, and for depth sensors in general, is how to measure occluded areas.

### 1.2 The Problem

The two major streams of work on WBSA (corrective factors for the formulae, and 3D techniques), have a common problem: both require trained personnel. In fact, the standard WBSA and BMI calculations use prediction equations which are accurate only for patients similar in size to the original study subjects. Using the formulae is quite intuitive, given the traditional way that physicians evaluate a subject through weight and height. However, this estimation misses a fundamental component: the body composition. Consider the behavior of the body mass index (BMI) between athletic and overweight subjects. Both have a BMI greater than 25, but one is an athletic healthy subject, the other is an overweight subject. This index, unfortunately, is not capable of distinguishing subjects with different body fat percentages. This fact is a common problem in measuring the radiation dose estimation [6] for obese people, where a wrong estimation in the surface area will create an underdosage of the treatment. To avoid this miscalculation, only trained personnel can establish when a predicting equation is sufficiently accurate or when to use a corrective factor for the given subject. At the same time, classical 3D body scanners, which give a better estimate, cannot be used without supervision either. The use of trained personnel, which can be expensive, could lead to human errors, and is not always feasible, like in an auto-assessment scenario. A simpler, faster and more reliable method to determine the WBSA could provide some significant advantages. Moreover, the formulae have some validity issues with young subjects (<15 years old) [32], the obese [17], and diverse races [26], [11]. Finding new variations or corrective terms for the formulae is very expensive, because using the old fashioned technique with wraps and moulds of plaster of Paris or using the modern 3D scanners will require the finding of these subjects, and then spending more time on the measurement process. Unfortunately, using common 3D datasets such as CAESAR 3D [33] does not solve the problem. In fact, these datasets are limited in subject diversity. Using datasets from different countries can be a solution, but at a cost, and such dataset are not always available.

#### 1.2.1 Previous work

Developing a vision system able to tackle the problem of WBSA calculation from a single view is not that easy. Surface analysis is the main step required in determining the Whole Body Surface Area (WBSA) through computer vision algorithms. Surface analysis is a key topic in computer graphics and the literature is very vast. We focus on WBSA analysis from 3D data. Previous work use bulky scanners based on structured light [34], three-dimensional photonic scanning [35], or portable stereo devices [36]. A set of body images are captured in multiple views [37] and after a long processing delay they obtain the overall body shape. A common pipeline in these methods is composed of the following steps: silhouette extraction, cleaned point cloud formation, smoothed surface, mesh reconstruction and horizontal transverse sections [35], [38]. In [37], the body shape is reconstructed from images taken at multiple views. However the acquisition setup is fairly unrealistic. The method used a turntable where a mannequin is posed. This environment is difficult to implement with a real human subject. Positioning the subject on a turntable requires a very constrained environment which is not feasible in practice. Using the mesh representation for an individual is not new in classical 3D scanning process [20, 39, 40], however, there is no work in the literature that analyzed the WBSA with respect to different view angles.

#### 1.2.2 Our solution: Virtual subjects and Virtual environment

Given these multiple problems, we decided to approach the WBSA calculation with an unusual methodology for this area. Our goal can be summarized with the following idea. *Using a simple Kinect device we want to obtain the accurate WBSA calculation of any given person regardless of differences in gender, race, obesity status*, *with the subject simply facing the device without the supervision of a trained personnel*. We want to use just one device that can acquire only one view of the subject, simplifying the setting required for an accurate estimation, and making it possible for accurate estimation in a home setting. The device will acquire just the visible portion of the body, and a subsequent prediction stage will reconstruct the overall WBSA.

Unfortunately, to be reliable, the described system needs training data, representing a large number of body shapes with significant diversity. Since the collection of this large amount of data is expensive, time consuming, and very difficult, we “virtualize” our training set, thus proposing a framework based on virtual subjects, computer vision and computer graphics techniques for the analysis and measurement of WBSA. Under this framework, which is motivated by similar approaches from the computer vision community [41], we generate a large number of virtual subjects (3D mesh data) that can capture variations in body shape and body size due to gender, race, and age.

**Fig 1:**
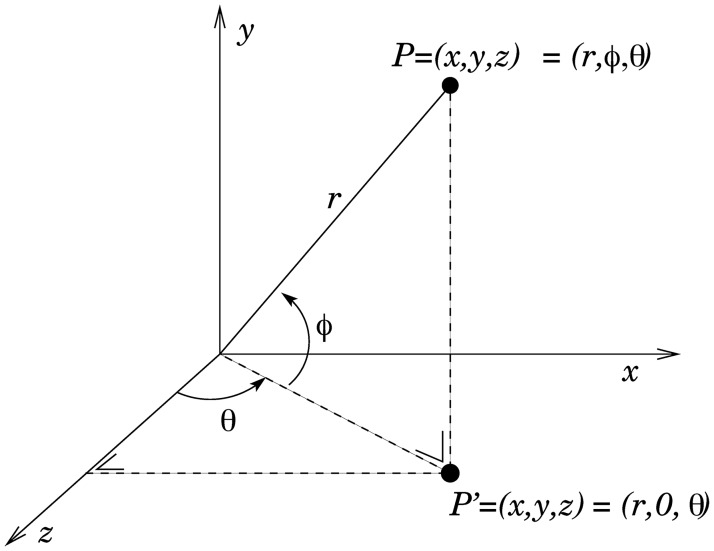
Polar coordinate system. Reference system used for the virtual camera model.

To obtain the same result of a real 3D acquisition process, like in a clinic, we need to simulate the acquisition process. This stage constitutes the main part of the system, able to reconstruct one view of the body (the side that has been viewed by the camera) from the whole mesh immersed in a virtual 3D room. Analyzing 3D data from a single point of view, usually away from the surface, adds more complexity, since only the visible part of the body can be acquired and analyzed. However, using a virtual environment and virtual subjects constitutes a huge advantage because we can control simultaneously the distortion caused by the acquisition process and the high variability of body measurements when acquired by a non-contact device. With this setup, we seek to find the relationship between the surface area computed from one single viewpoint (we call it view body surface area, VBSA) and the whole body surface area (WBSA) as a function of the camera position (distance, and orientation, see the reference system adopted in Fig 1), through different body shapes. Learning this relation will be extremely useful, since using only one view we can predict the WBSA of the subject more rapidly. However, the presented framework can be useful to study a more general problem, such as the behavior of the VBSA in a more unconstrained environment, such as the video surveillance environment. In this setting, the position of the body with respect to the camera, body pose, camera intrinsic parameters and camera lens distortion all play a huge role in the final measure. The proposed Virtual Environment considers all these parameters in one unique model capable of computing the WBSA from the single view (one 3D camera cannot measure the WBSA of the body in one single shot).

### 1.3 Contribution

Today there are many devices with very good imaging capabilities, and many of them are portable and easily available off-the-shelf. Weiss et al [27] show that it is possible to achieve an accuracy similar to cumbersome 3D laser scanners with a single low-cost depth sensor. However, to take advantage of these new devices and develop new algorithms, data collection is often a big obstacle. Our contribution is thus a computer vision environment able to analyze 3D data coming from these devices while simulating the data acquisition process. We apply the developed framework to a very reoccurring problem in health care: the estimation of the whole body surface area. We show that with the developed framework it is possible to obtain an accurate WBSA estimation using only one view of the body, even for overweight subjects, or for a particular category of subjects for which the WBSA with the formulae is particularly troublesome. The presented framework can be used to test new techniques and train machine learning algorithms to be used in different scenarios, such as in an Emergency Room for burn area detection.

## 2 Materials and Methods

**Fig 2:**
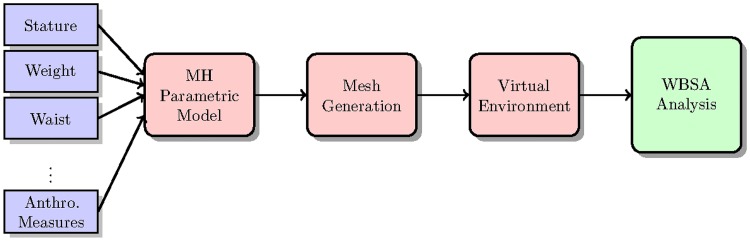
Pipeline for the proposed Virtual framework.

Fig 2 shows the basic pipeline for the proposed framework. The initial step is the creation of a virtual population of subjects. This virtual population needs to capture the statistical attributes of a real population with all the possible subject shapes present in nature. This stage is represented by the first two blocks in Fig 2. First, we generate the sets of parameters that define each subject body (parametric model). These parameters are used in the second step to synthesize the mesh. Subsequently, the Virtual Environment will process the mesh, obtaining a point cloud as a result of a ray tracing operation on the mesh, given the camera position (distance, orientation) with respect to the subject. Finally surface reconstruction and statistical analysis are performed to compute the model parameters for the prediction.

### 2.1 Datasets

The human body can assume different shapes with age, gender, race and health status, and it can also assume a variety of poses: it’s a non-rigid object. Though there has been significant work to lower the complexity in the analysis of body shape, it is still an open problem. The approach that seems most promising is to use a parametric model that can describe the body using a pool of parameters. In our solution, we use a parametric model to define each subject, then a graphics engine will create the final mesh given the model parameters and body pose data. Here, we focus more on shape analysis without motion, which is the most common situation in a physician’s clinic. However, the same framework can be used to track and analyze subjects in different poses.

#### 2.1.1 3D body model and virtual body framework

One of the most popular non-rigid parametric body model is the SCAPE method [29]. SCAPE is a data-driven method for building a human body model that spans variations in both shape and pose. The method is based on a representation that incorporates both articulated and non-rigid deformations. Learning in the model is constituted by two operations: learning a pose deformation model from a subject with multiple poses, and learning a shape model from many subjects with a neutral pose. The decoupling of shape and pose deformations in the SCAPE model has a major limitation: 3D meshes of different individuals can change in a similar manner for the same pose change. Various efforts have been made to improve accuracy and constraints of the SCAPE model. In our work we use a different body model. Makehuman [42] (MH) is an open source 3D computer graphics application, designed for the prototyping of photo realistic humanoids to be used in 3D computer graphics. MH takes advantage of 3D morphing. Starting from a standard (unique) human base mesh, the mesh can be transformed into a great variety of subjects (male, female, African, Caucasian, Asian, adult, kid, etc), obtained using a linear interpolation of different target models. Using this technique, it is almost possible to reproduce subjects with very different body shapes. MH uses a GUI with sliders that can change the main quantities: stature, weight, gender, ethnicity and muscularity (fat / muscle ratio) called macro parameters, and body part measurements called micro parameters. The macro and micro parameters constitute the parameter sets that define each subject. MH is specifically designed for modeling virtual humans as characters in virtual reality and gaming, with a simple and complete pose system that includes the simulation of muscular movement. We modify MH for our needs, generating two synthetic 3D datasets, complete with the anthropometric measurements of the subjects.

### 2.2 Generation of Virtual (Synthetic) Humans

MakeHuman has been used previously to create a dataset of realistic human bodies. The main applications have been in the generation of a human population for bed fitting [43], for learning a random forest in a computer vision system [44, 45], and on camera positioning [46]. To our knowledge, this is the first application in healthcare.

**Fig 3:**
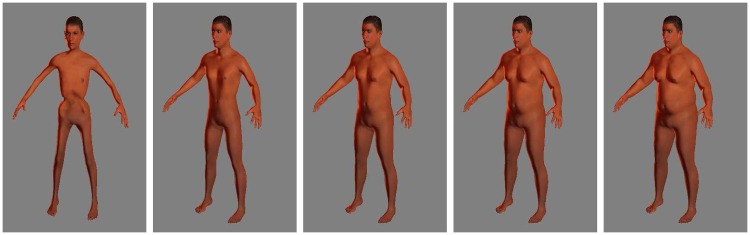
Sample Male subjects in Virtual NHANES dataset.

**Fig 4:**
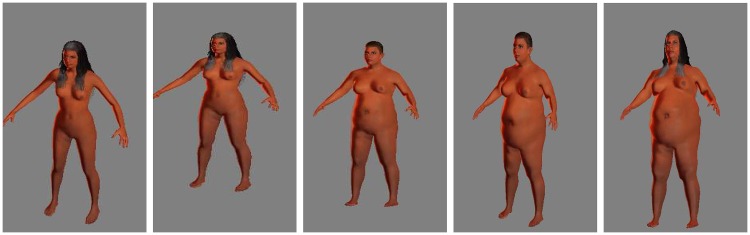
Sample Female subjects in Virtual NHANES dataset.

With MakeHuman, it is possible to create a subject, save the parameters, and export the mesh. Useful available options include adding skin texture and clothes as part of the model. In our work, we included the **caucasian** skin texture in all the subjects in the datasets as shown in Figs 3 and 4, but skin analysis wasn’t part of our work. We completely avoid the use of clothes in this project. That is, all subjects are assumed to be nude, or to be wearing tight-fitting clothes. MH does not provide a tool for creating a population of human bodies, but it has a very friendly interface to manipulate a single body. This is very useful for computer graphics environments, where you generate a few subjects in great detail, but not useful in our scenario. Thus, we developed a MH plugin able to create bodies and export them as mesh files (obj). With this plugin we can create thousands of bodies in a relatively small time (∼ 2h 30 min on a quad core CPU for 20000 subjects). In addition we take advantage of the MH measuring tool to create a table of body measurements in NHANES [47] style. We create two datasets: a **completely random virtual dataset** (19995 subjects) and one derived from the NHANES dataset [47] that we call **virtual NHANES dataset** (12471 subjects). Table 1 show the statistics on the two datasets.

**Table 1:** Statistics on the datasets. (S denotes the stature).

	Virtual NHANES	Virtual Random	EORTC
Total subject	12471	19995	3000
Males	6348	10049	NA
Females	6123	9946	NA
Kids (≤15) yrs	2380	5786	NA
Adults (>15 yrs)	10091	14209	NA
Small (*S* ≤ 130 cm)	3171	14209	NA
Normal (*S* = 130 − 200 cm)	9229	12449	NA
Big (*S* > 200 cm)	1575	4612	NA
Ages	12 − 70	12 − 70	adult
Mean WBSA (*dm*^2^)	137	167	173
SD WBSA (*dm*^2^)	51	66	NA

Virtual NHANES, and Virtual Random dataset composition respect to EORTC.

#### 2.2.1 Virtual NHANES dataset

This Virtual dataset has the goal to mimic a real human population. Since we can easily obtain datasets with body part measurements (CAESAR 1-D [33], NHANES [47]), we decided to use these measurements to build the respective virtual subjects constituted by 3D data (mesh composed of quads for MH). Specifically, we use the subject measurements available from the National Health and Nutrition Examination Survey III (NHANES III) dataset [47]. We select 500 subjects from NHANES aged between 15 and 75 (Table A in S1 File). Using the body measurements, we generate the corresponding parametrized body and subsequently 3D mesh. The parameters used for the generation are gender, age, height, race, breast size, upper leg height, upper arm length, upper arm circumference, thigh circumference, and waist circumference. MH represents all the macro parameters and some micro parameters as a normalized value between 0 to 1. For some of these parameters we know the range used by MH, in which case we can recover the real measure. For some, we do not. We decide to allow these parameters to be variable in the data range. The first reason in doing so is that, since MH use a normalized weight, it could be misleading to normalize the NHANES weight with the MH range. The second reason lies in the initial goal of this project: study the WBSA related to body shape. Changing weight and muscle ratio, but keeping the other parameters fixed is like varying the body mass of the subject. But at the same time, by varying the muscle/fat ratio, we obtain a *fat version* and a *skinny version* of the same individual. This is very interesting since it can be used to learn how the WBSA change with respect to variations in weight and muscle/fat ratio. In fact, analyzing the NHANES dataset [47], we discover that many individuals are very similar, and we couldn’t get a larger and continuous shape variations. Thus, we generated a population composed of the original subjects plus 25 variations of each subject for a total of 12500 subjects for the Virtual NHANES dataset.

#### 2.2.2 Virtual Random dataset

**Fig 5:**
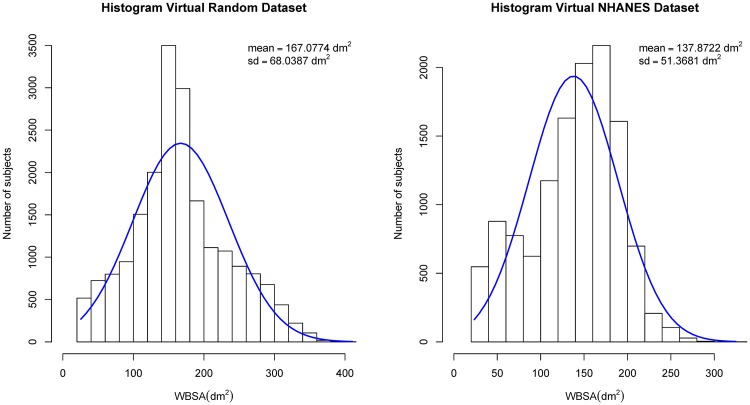
Distribution of WBSA values. Virtual Random dataset (Left), and Virtual NHANES dataset (Right).

The Virtual NHANES dataset is aimed at mimicking a real population and is composed of subjects derived from the NHANES dataset, with limited variations. Since we also want a very challenging dataset that contains any possible variation of body shapes, with some bodies that could be hard to find in a real population, we decided to generate one. This dataset, which we call **Virtual Random**, is created using the whole range of possible parameters variations in the MH engine. To create this dataset, we generated random values for the following macro parameters (stature, gender, race, weight, and muscle ratio). To increase the variability of the obtained bodies we generated these parameters using a uniform distribution rather than a normal distribution. Parameters distributions of real populations are closer to a normal distributions, however using a uniform distribution guarantees a higher number of subjects at the extremes of the possible ranges. In fact, as show in [48], the WBSA of subjects at the extremes (e.g. kids, and very obese subjects) can create significant problems in the prediction. However, to avoid the creation of subjects that are too dissimilar from real human bodies we restrict the randomly generated parameters to some possible intervals. The Virtual Random dataset is important in evaluating the performance of the proposed approach for extreme body shapes and body sizes. Fig 5 shows the distributions of the WBSA for the Virtual NHANES dataset, and Virtual Random datasets.

### 2.3 Virtual Environment (Camera)

The creation of the two virtual datasets is motivated mainly by the difficulty at collecting 3D from real subjects. Using a generative data-driven approach makes the analysis and developing of 3D techniques a less challenging task. To complement the generative method, and take completely advantage of the generated subjects we created a virtual laboratory, composed of a virtual camera setup. The Virtual Environment is the main tool for the analysis of 3D body mesh. It can simulate a real acquisition process by a large variety of devices, not just the new depth cameras sensors, but stereo systems, traditional 2D cameras, and all the devices that can be modeled with a pin-hole camera model [49].

In 3D animation, a virtual camera is a function of the animation software that works and behaves in the same way a camera or digital camera would in real-world situations. The virtual camera is based on mathematical calculations that determine how the object will be rendered based on the location and angle of the virtual camera. As with a real camera, when working with a virtual camera in 3D animation programs, you can use functions like pan and zoom or change focus and focal points. The method we used in our virtual camera is a simple ray casting method [50]. In ray casting, geometric rays are traced from the eye of the observer (camera) to sample the object (subject) toward the ray direction. The points of intersection between the rays and the object (body) constitute the (x, y, z) positions of the object (body) surface. In this form of ray casting (different from volume ray casting) the rays detect only the points of the first intersection (points that face the camera) and not the second intersection (points that don’t face the camera).

Using this virtual camera setup we can simulate the acquisition process of a virtual body with a depth camera (3D scanner, XBOX Kinect, TOF camera). The Virtual Environment permits us to change the camera position relative to the body (pan, tilt, zoom). We can use different camera models with different fields of view (FOVs) and noise models. Typical artifacts with depth cameras is the presence of holes in the acquired point cloud. This kind of noise is very important to consider in our analysis, since it directly affects the surface, and thus the surface area. As we will show later, the presented virtual camera, although based on well known computer graphics techniques, presents some novelties tailored for the WBSA analysis, capable to consider different sources of noise: device, body occlusions, reconstruction process, and others.

The Virtual Environment is designed with the primary goal to be able to analyze a large population of subjects. Following this idea, it includes a routine able to load the information about the subjects one at the time, generate the corresponding point cloud result of the ray casting, and store WBSA, VBSA and body measurements in cvs/xlsx tables with the respective settings (angles, resolution, noise model, camera parameters). The Virtual Environment can generate different outputs of the subject examined: point cloud, mesh, the faces of the original mesh visible from one direction (ground truth), the reconstructed mesh from point cloud of one view, the area of the reconstructed subject (only the area visible from camera direction), and the list of the faces visible from one view.

#### 2.3.1 Camera model

**Fig 6:**
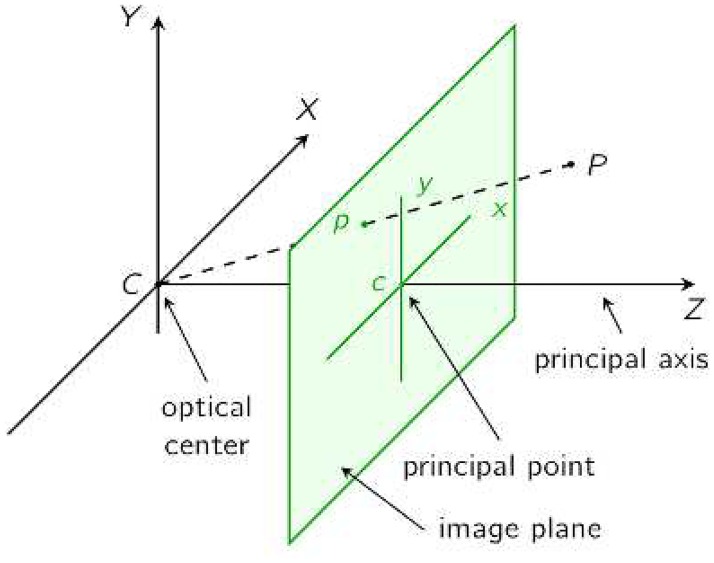
Pinhole camera model.

The ray casting method gives only the framework to reconstruct a view given the position of the camera. To simulate a real camera, we need to add to the ray casting algorithm, information about the camera lens characteristics, namely the intrinsic parameters [49]. We use the pinhole camera model to describe the image acquisition process, which is largely employed to parametrize a large number of cameras. The pinhole camera model defines the geometric relationship between a 3D point and its 2D corresponding projection onto the image plane. This geometric mapping from 3D to 2D is often called a perspective projection. We denote the center of the perspective projection (the point in which all the rays intersect) as the optical center or camera center and the line perpendicular to the image plane passing through the optical center as the optical axis. Additionally, the intersection point of the image plane with the optical axis is called the principal point. The pinhole camera (see Fig 6) models a perspective projection of 3D (*X*, *Y*, *Z*) points onto the image plane (*x*, *y*), and can be described as follows:

$$\begin{array}{c} {\left(X,Y,Z\right)}^{⊤}\overset{Projection}{\to }{\left(x,y\right)}^{⊤} \end{array}$$ (1)

$$\begin{array}{c} x=f\frac{X}{Z}\;y=f\frac{Y}{Z} \end{array}$$ (2)

where *f* is the focal length of the camera, i.e., the distance between the image plane and the pinhole.

**Intrinsic/Extrinsic Parameters**. The complete camera model can be represented with the following relation.

$$\begin{array}{c} \lambda \underset{x^{\prime }}{\underset{︸}{\left[\begin{array}{c} x^{\prime }\\ y^{\prime }\\ 1 \end{array}\right]}}=\underset{K}{\underset{︸}{\left[\begin{array}{ccc} fs_{x} & fs_{\theta } & o_{x}\\ 0 & fs_{y} & o_{y}\\ 0 & 0 & 1 \end{array}\right]}}\underset{\Pi_{0}}{\underset{︸}{\left[\begin{array}{cccc} 1 & 0 & 0 & 0\\ 0 & 1 & 0 & 0\\ 0 & 0 & 1 & 0 \end{array}\right]}}\underset{g}{\underset{︸}{\left[\begin{array}{cc} R & T\\ 0^{⊤} & 1 \end{array}\right]}}\underset{X_{0}}{\underset{︸}{\left[\begin{array}{c} X_{0}\\ Y_{0}\\ Z_{0}\\ 1 \end{array}\right]}} \end{array}$$ (3)

The matrix Π_0_ is the canonical projection matrix. The matrix *K* consists of the intrinsic parameters of the camera. Here *f* is the focal length of the camera, *s*_*x*_ and *s*_*y*_ give the relative aspect of each pixel. *o*_*x*_ and *o*_*y*_ specify the coordinates of the image center. *s*_*θ*_ is the skew in the shape of the pixel, i.e., its deviation from an axis aligned rectangle. The matrix *g* defines the pose of the camera. The elements of *g* constitute the extrinsic parameters of the camera (the position of the camera center relative to the world coordinates). Here, *R* is a 3×3 rotation matrix and ***T*** is a vector in ${\mathbb{R}}^{3}$. These two quantities represent rotation and translation of the camera relative the world coordinate. To find all the parameters (*K* and *g* matrices) of the camera model we need to calibrate the camera [51]. In our case the device is a 2.5D camera. In this kind of device the information acquired by the sensor is not the chromatic information (RGB) but an intensity value proportional to the distance of the point *P* (see Fig 6). These devices, however, still follow the pinhole camera model [49], but the camera calibration procedure is different [52], [53], and the final parameters are still the same as in the above equation.

We calibrate the Microsoft Kinect for Xbox [28] using the method in [53] and we use the calibration intrinsic data to simulate this camera in our Virtual Environment framework. Apart from the intrinsic parameters in the pinhole model, the Virtual Environment also needs to account for other non-ideal behavior of the device. The geometric characteristics of the camera are captured in the camera model, but we need to account for the electrical characteristics of the sensor. The sensor and the electrical components connected to it convert the light into electrical signals, and then into digital signals (gray level intensities). In this process, the signal is typically corrupted by noise, which in the case of a 2.5D device will result in distorted surfaces.

A number of general methods can be used to de-noise the depth map, and some proved to be very effective. However, in our Virtual Environment, the goal is to simulate a real camera, using a model that is able to replicate the real camera behavior. We implement the method proposed by Nguyen et al [54]. This method measures both lateral and axial noise distributions, as a function of both distance and angle of the Kinect to an observed surface. Using this procedure we are able to simulate different scenarios, add noise to the final acquisition, and implement de-noising strategies able to reduce the effect of noise on the WBSA calculation.

### 2.4 Whole body surface area from a single view

**Fig 7:**
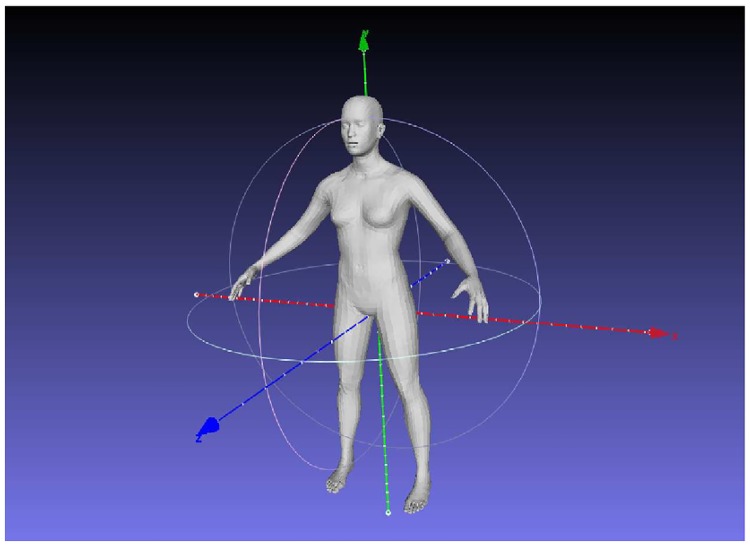
Makehuman mesh model.

**Fig 8:**
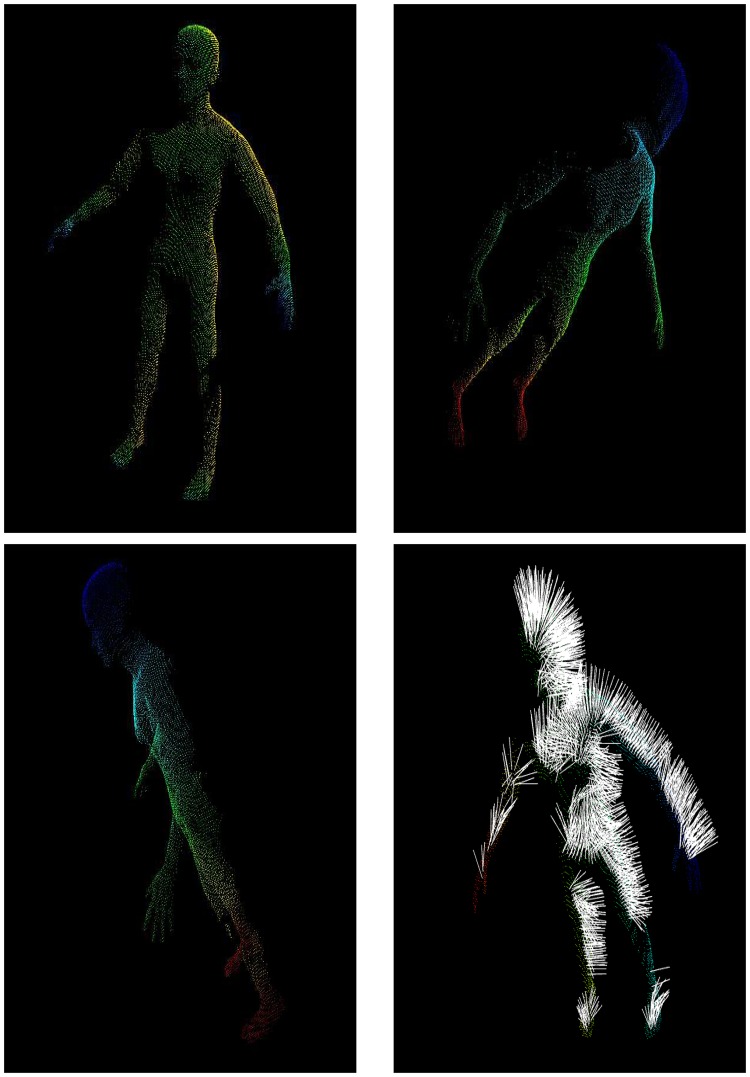
Point cloud results from the Virtual Environment. Subject 8 from Virtual NHANES dataset at *θ* = 60° *ϕ* = 60° seen at different angles. From these shots, it is possible to see the missing parts of the body due to the raycasting operation, given the camera position.

The whole body surface area (WBSA) is the 2D area of the superficial body skin. In our case, we are using the virtual subjects mesh as an approximation of the skin, and the respective area as WBSA. Common meshes are composed of vertices, faces and edges. The faces can be regarded as 2D polygonal with the given vertices that constitute the surface of the 3D object. Fig 7 shows the wireframe representation of the body mesh used. An object acquired with a 3D scanner can have around 50000 faces. The computation of the total area of a mesh is nothing more that the sum of the 2D area of each face [55]. The area calculation can be done using normal geometric formulae using the edge lengths of each face. Unfortunately, there are some complications in this apparently simple operation. As mentioned, the human body can assume a large variety of poses, and it can assume different shapes from a different observation angle. In this situation, occlusions and surface curvature make the area calculation from a single view a more complex problem. The result of the ray cast method is a point cloud obtained by the intersection of the rays with the subject (Fig 8). The density of the point cloud depends on the resolution of the sensor and the distance from the camera (Eq 2). To calculate the surface area from the point cloud we need to reconstruct the mesh surface. The literature on this topic is vast, especially in computer graphics. Traditional methods include marching cubes [56], Poisson surface reconstruction [57], greedy surface reconstruction [58]. Unfortunately, this process can generate additional noise and nuisances in the form of topological errors, holes, and surface distortion. However, to analyze the WBSA-VBSA relation without the reconstruction noise, we need to calculate the area of the mesh triangles visible to the camera directly from the original mesh without any distortion, creating what we consider the ground truth value of the VBSA. Using the vanilla implementation of the ray cast algorithm will not permit to retrieve the values for the areas, but just the (x, y, z) position of the rays intercepting the mesh. The simple, but efficient contribution, is the use of a list of triangle identifier (IDs). The triangle ID is a unique code associated with one and only one triangle of the mesh. For each ray incident on the mesh surface, we retrieve the triangle identifier (ID), other than the (x, y, z) point lying on the triangle surface. The collection of points will constitute the point of cloud, the triangle IDs will constitute the list of visible triangles. We order, sort and eliminate multiple IDs, since multiple rays can hit the same triangle. Finally, we retrieve the areas of the triangles in the list from the original mesh. This procedure will give us the “ground truth” VBSA, without any additive noise due to intermediate processing. This method gives us very accurate results, but it can overestimate the real area if the mesh has a low number of triangles (coarse resolution). In fact, if a triangle is partially visible, this method will still compute the whole triangle area. However, since each body mesh is composed of roughly 28000 triangles, each triangle has a very small contribution, and a portion of a triangle has an even smaller contribution. A solution is to increase the number of triangles using a surface subdivision algorithm. The list of triangles is very important, because we can study the WBSA-VBSA relationship and the camera position without the additive noise coming from the sensor, the surface reconstruction process, or shaders. Without loss of generality, the presented contribution is extremely useful for learning a new machine learning algorithm robust to a large variety of nuisances.

Another typical issue with modern depth cameras is the surface reflection problem. When the surface of the object is reflective, or illuminated by a strong source of IR light (Sun), the acquired depth map of the surface present annoying holes. Different techniques has been presented to “fill” the holes [59], but it can be very difficult when the surface can assume very complex shapes. These kind of reflection, or translucent materials has been extensively simulated with ray casting techniques. Adding a light source in the Virtual Environment, other than the camera, we can take advantage of shading models for a better formalization of the problem. A more robust solution will be to use a data-driven approach, able to learn the surface deformation, the occluded areas, and the artifact due to noisy data. Our developed system constitute a framework able to learn a so defined machine learning system. Given the list of observable triangles, we can randomly select a subset and eliminate some triangles from the list. At the same time, we can erase the respective points from the point cloud. The effect is the creation of holes in the depth map obtaining the usual artifact of the depth sensor. The defined generative data-driven system can easily train a supervised machine learning algorithm. Another application is the analysis of a new reconstruction algorithm able to tackle more challenging situations. For example, under this framework, we can acquire the ground truth of the surface area directly from the original mesh, then we can calculate the distortion introduced by the reconstruction algorithm, and the prediction error using the VBSA. Under our framework, all these processes can be treated separately, each with its own additive noise model.

#### 2.4.1 Surface area calculation

**Fig 9:**
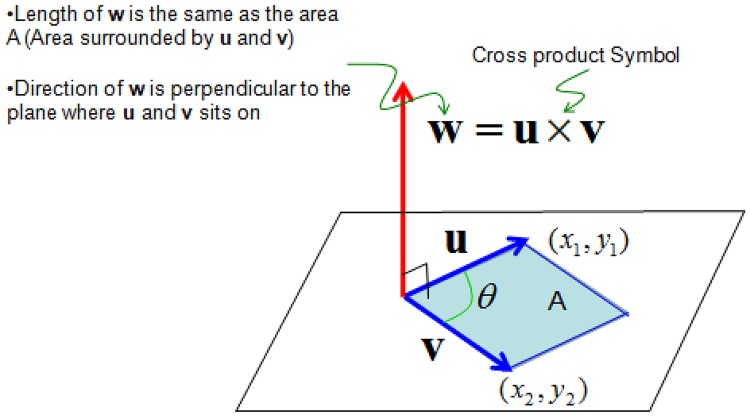
Mesh surface calculation. Graphical interpretation of the cross product operation.

As an application of the presented framework, we analyze the relationship between WBSA and VBSA while varying the camera position. The initial value of the surface area for the whole mesh has been calculated in the MH plugin and stored in the tables (see Table B in 4.4). After ray casting operation, we need to calculate the visible surface area, VBSA from the visible part of the mesh. Given the edges **u** and **v** (see Fig 9) of a triangle, to obtain the surface area we use the standard relation:

$$\begin{array}{c} A=\frac{1}{2}\left|u\times v\right| \end{array}$$ (4)

where × denotes the cross product between the two vectors **u** and **v**, and | | denotes the magnitude of the cross product. The magnitude of the cross product is the area of the parallelogram whose edges have length **u** and **v** (see Fig 9). This is twice the area of the triangle whose edges are **u** and **v**. The result of this operation is the surface area of a single triangle. Our initial MH mesh is composed of about 28000 faces, but given the simplicity of this operation it can be done almost in real time.

#### 2.4.2 WBSA-VBSA formulation

Having obtained the view body surface area from a given view, say VBSA(*θ*, *ϕ*) the next step is the analysis of the WBSA-VBSA relation. Du Boise and Du Boise [1] calculate the WBSA as the sum of the areas from different body parts. In [24] the subdivision is given by the acquisition process, where the frontal part of the body account for more than 50%(52%) of the WBSA. In a similar way, we decompose the WBSA as the observable body surface area (VBSA) and un-observable or missing one. The goal is to infer the WBSA from the VBSA:

$$\begin{array}{c} WBSA=f(VBSA,c) \end{array}$$ (5)

where the vector **c** contains the unknowns of the prediction problem. The approach is as follows. We process the bodies generated in the Virtual NHANES dataset and Virtual Random dataset with the Virtual Environment, positioning the camera at different locations, that span a solid angle covering all the possible camera views of the body. The camera positions comprises in the solid angle within −90 ≤ *θ* ≤ 90 and −90 ≤ *ϕ* ≤ 90. Since the virtual datasets are composed of symmetric subjects, we limit the azimuth angle on the left side of the subject covering the angles from the front left side to the back left side (see Fig 1). We limit the body pose to the default pose in Fig 7, maintaining a constant distance between the subjects and the camera (4.3 meters). This distance has been found empirically by considering the tallest subject in the dataset.

### 2.5 Statistical analysis

**Fig 10:**
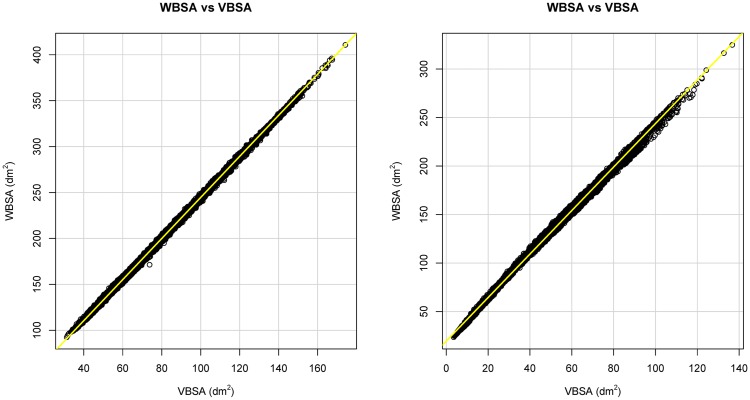
Relationship between WBSA and VBSA. (Left) Using Virtual Random dataset at *θ* = 0°, *ϕ* = 0°. (Right) Using Virtual NHANES dataset at *θ* = 0°, *ϕ* = 0°.

The result from the Virtual Environment is the VBSA-WBSA pair for each camera view (Table B in S1 File). To find the relation between WBSA and VBSA, we need to assume a statistical model to be used for the inference. From a first plot of VBSA vs WBSA (Fig 10) we can see that a linear regression model can potentially obtain good results. The vector **c**(*θ*, *ϕ*), which is a function of the camera position *θ*, *ϕ* is composed by the linear regression parameters:

$$\begin{array}{c} c=\{c_{1},c_{0}\} \end{array}$$ (6)

where *c*_0_ is the intercept, and *c*_1_ is the view area linear coefficient. Given a certain location of the camera (*θ*, *ϕ*), to find the linear parameters we need to solve the least squares problem:

$$\begin{array}{c} \min_{c_{1},c_{0}}\sum_{i}||WBSA_{i}-\left(c_{1}\left(\theta ,\phi \right)\cdot VBSA_{i}\left(\theta ,\phi \right)+c_{0}\left(\theta ,\phi \right)\right){\left|\right|}_{2} \end{array}$$ (7)

To avoid overfitting problem in the prediction, we use a *k*-fold cross validation modality with *k* = 10. The result is the vector **c** of linear coefficients as a function of the two angles. We repeat the training for different partitions of the dataset: males, females, kids, adults, small stature (*S* ≤ 140*cm*), normal stature (*S* = 140 − 200*cm*), big stature (*S* > 200*cm*). Another interesting analysis is the use of some measurements in the prediction. As we can see from the correlation matrix in Table 2, some measurements are highly correlated with the WBSA (*ρ* > 0.9). We conduct this analysis with the intent to show possible gains in the use of some body measurements. From the relation in Eq 5, the extension of the linear least squares method to multiple inputs takes the form:

$$\begin{array}{c} WBSA=c_{1}VBSA+c_{2}Stature+\cdots +c_{0} \end{array}$$ (8)

where the vector **c** = {*c*_0_, *c*_1_, …, *c*_*n*_} is composed by the unknown linear regression parameters. The performance improvement using the anthropometric measurements is strictly dependent by the accuracy of these measurements. Theoretically, with a system able to capture other measurements with high accuracy it is possible to obtain a better prediction. However, this often is not the case, because holes and occlusions play a crucial role in the accuracy. We report the results of the regression without considering any measurement error.

**Table 2:** Virtual Random dataset, correlation matrix. All subjects at *θ* = 0° *ϕ* = 0°.

circ.	Stature Neck circ.	WBSA Frontchest	VBSA	Gender	Age	Hips circ.	Waist circ.	Bust circ.	Underbust		
Stature	1	0.9742	0.9701	0.0929	0.4657	0.8814	0.7798	0.8404	0.8303	0.8103	0.8976
WBSA	0.9742	1	0.9992	0.1396	0.4991	0.9228	0.8582	0.8933	0.891	0.8851	0.9241
VBSA	0.9701	0.9992	1	0.1445	0.5064	0.9216	0.8638	0.8912	0.893	0.885	0.9187
Gender	0.0929	0.1396	0.1445	1	-0.0091	0.0363	0.1508	0.187	0.3839	0.3969	0.2713
Age	0.4657	0.4991	0.5064	-0.0091	1	0.6306	0.5493	0.6237	0.5491	0.5237	0.5737
Hips circ.	0.8814	0.9228	0.9216	0.0363	0.6306	1	0.9539	0.9371	0.9158	0.8848	0.9319
Waist circ.	0.7798	0.8582	0.8638	0.1508	0.5493	0.9539	1	0.9039	0.9342	0.8902	0.8728
Bust. Circ	0.8404	0.8933	0.8912	0.187	0.6237	0.9371	0.9039	1	0.9476	0.9331	0.9499
Underbust circ.	0.8303	0.891	0.893	0.3839	0.5491	0.9158	0.9342	0.9476	1	0.9663	0.9571
Neck circ.	0.8103	0.8851	0.885	0.3969	0.5237	0.8848	0.8902	0.9331	0.9663	1	0.9398
Frontchest	0.8976	0.9241	0.9187	0.2713	0.5737	0.9319	0.8728	0.9499	0.9571	0.9398	1

VBSA is highly correlated with WBSA and stature.

## 3 Results

### 3.1 Generated Virtual datasets

Fig 7 shows the mesh model for one of the generated subjects, while Fig 5 shows the distribution of WBSA in the datasets. Examples of males and females in the dataset under different muscle/fat ratios are shown in Figs 3 and 4. MH defines a texture for a given subject based on gender, age and races. It is also possible to add some other structures such as short or long hair, however this feature was not used in this work. Table 1 shows the compositions of the generated datasets. We have included information on the EORTC (European Organization for Research and Treatment of Cancer [7, 60]) dataset for comparison. For the generated datasets the WBSA is computed from the original mesh. For the EORTC, the WBSA is computed using the traditional formulae. The Virtual Random dataset has a notably larger variance comprising many varieties of subjects. The Virtual Random dataset contains subjects hard to find in the general population (notice that in Fig 5, left, there are subjects with WBSA approaching 400 dm^2^!). The EORTC has an average WBSA higher than the Virtual NHANES. Since the EORTC considers cancer patients, it is composed almost exclusively of adults. Our datasets instead is comprised of a large varieties of ages.

### 3.2 View BSA is strongly correlated with WBSA

First, we analyzed the correlation between the available quantities (WBSA, VBSA and body measurements). Table 2 shows the correlation matrix for the analyzed quantities for all subjects in the Virtual Random dataset for (*θ* = 0°, *ϕ* = 0°). We use the Spearman’s *ρ* as our statistic. The WBSA is strongly correlated with the VBSA (*ρ* = 0.9992). The WBSA is also strongly correlated (*ρ* > 0.9) with the following quantities: stature, hip circumference, frontal chest. Other measures that have high correlation (*ρ* > 0.8) with the WBSA include: waist circumference, bust circumference, underbust circumference and neck circumference. The correlation between WBSA and stature is trivial since the stature is one of the parameters directly connected with the body surface area (all the WBSA formulae are based on stature and weight). The correlation is an interesting indicator, since it can help us to determine which parameters can give a better prediction of the WBSA.

### 3.3 Linear regression analysis

**Fig 11:**
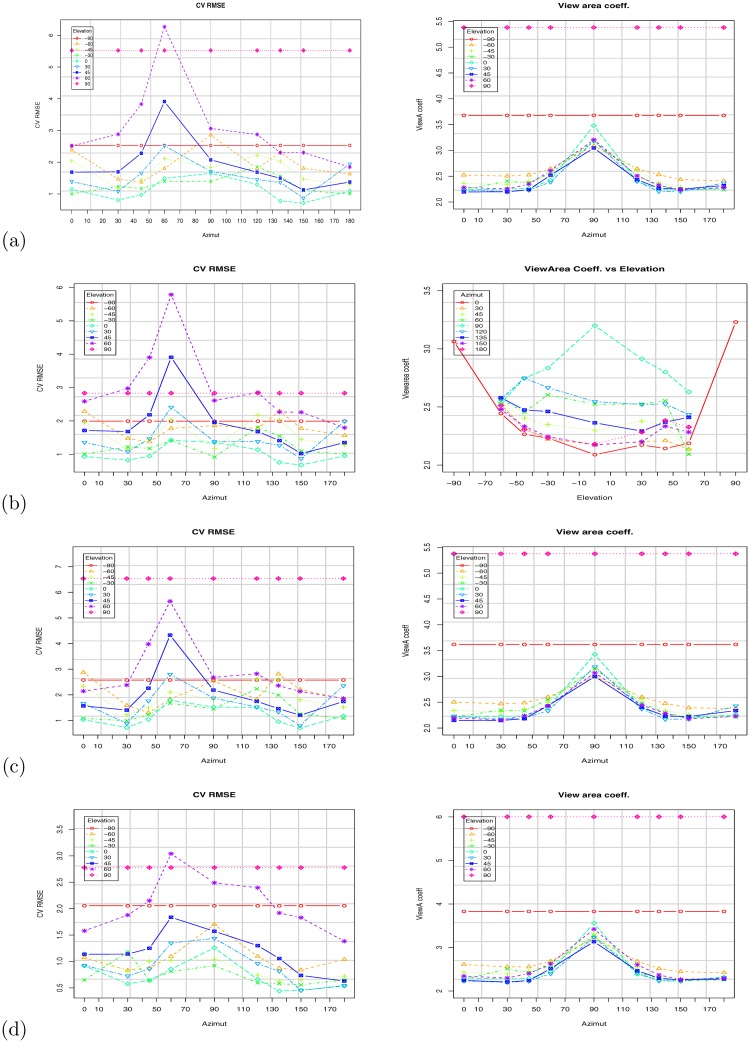
Results based on the Virtual Random dataset. Impact of camera orientation (azimuth and elevation) on the VBSA prediction coefficient (Right), and on the cross validation root mean square error (Left): (a) All subjects; (b) All subjects with stature; (c) Adult subjects; (d) Kid subjects.

**Fig 12:**
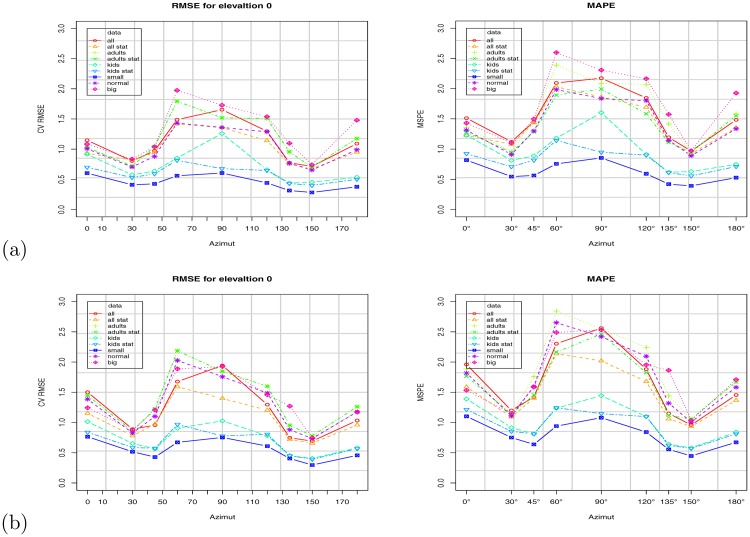
WBSA prediction errors at elevation angle. *ϕ* = 0°: (a) Using Virtual Random dataset, (b) Virtual NHANES dataset. Results for different groups: all subjects, adults, kids, small, normal, big stature. W/O stature.

Fig 10 shows the scatter plot using the VBSA (x-axis) and WBSA (y-axis). Each subject is represented by a point in the coordinate (VBSA, WBSA). The relation is clearly linear, thus we analyze the performances of a linear regression model. We use the R regression DAAG Tool model [61] to find the unknowns of the model. We use a *k*-fold (k = 10) cross-validation to calculate the prediction error for the linear models defined in Eqs 5 and 8, repeating the experiments using different partitions of data (all subjects, males, females, adults, kids, small stature, normal stature, big stature). Figs 11 and 12 and Tables C-AF in S1 File show the results of this prediction. Each row in the Tables corresponds to an orientation of the camera with respect to the subject. For each subject, we analyzed the angles *θ* = 0°, 30°, 45°, 60°, 90°, 120°, 135°, 150°, 180° for the azimuth, and *ϕ* = 0°, ±30°, ±45°, ±60°, ±90° for the elevation. For *ϕ* = ±90°, we only had *θ* = 0°, since changing the azimuth angle will not affect the view area. We report (in S1 File) some statistical indicators needed to evaluate the validation of the fit, as t-value, standard deviation, and different prediction errors given by R: residual standard error (SE), cross validation root mean square error (RMSE), cross validation mean square prediction error (MSPE), cross validation mean absolute prediction error (MAPE).

The linear regression model is validated using the QQ plot (S1 Fig) of the residuals, and by checking that the assumption of constant variance (homoscedasticity) needed for least mean square estimation is met. We show the complete analysis in S1 File.

### 3.4 Impact of Camera Distance

The distance of the depth sensor from the subject can significantly affect the inferred WBSA. We hypothesized a fixed distance like in a real clinical scenario, where the subject is standing in front of the camera. In this situation the distance is determined by the field of view of the camera. To accommodate a large varieties of subjects with different statures, we compute the distance of the subject from the camera center using the pinhole model: [49]:

$$\begin{array}{c} \text{max}\;\text{Stature}=2\tan(\psi )D \end{array}$$ (9)

where *ψ* is the depth sensor vertical field of view and *D* is the distance of the subject from the camera center. We find that a distance of 4.3 meters can accommodate all the subjects in the two dataset. The reported results are all relative to the default distance of 4.3 meters. In S1 File we report the results for different distances in the range 3.5–5 meters. It’s interesting to note that for distances below 4.3 meters some bodies cannot fit in the frame, a situation typical in a video surveillance setup with fixed camera. The results show that the WBSA-VBSA relation quickly diverge from linearity. The distance heavily affect the final result, since the accuracy of the sensor deteriorates with the distance, following the square law [54]. In our analysis this behavior is noticeable from the reconstruction error analysis, where we combine the different noise components.

### 3.5 Impact of Azimuth and Elevation on computed WBSA

**Fig 13:**
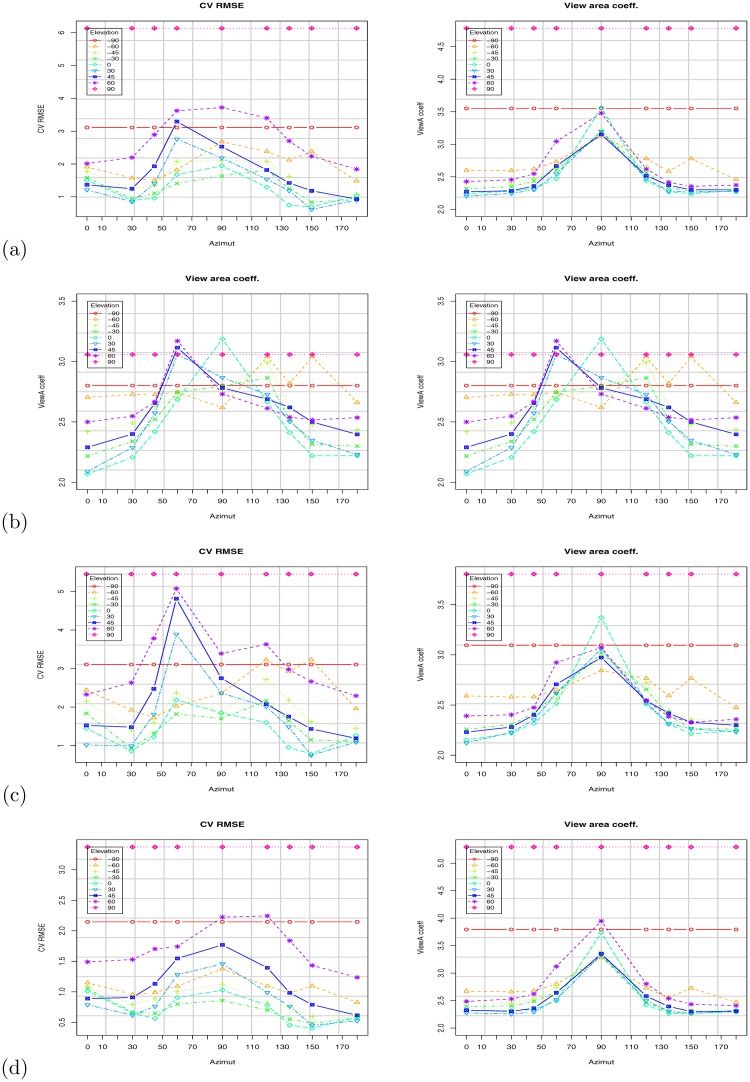
Results based on the Virtual NHANES dataset. Impact of camera orientation (azimuth and elevation) on the VBSA prediction coefficient (right), and on the cross validation root mean square (left): (a) All subjects; (b) All subjects with stature; (c) Adult subjects; (d) Kid subjects.

As expected, the camera orientation (as captured by the azimuth *θ* and elevation *ϕ*) has a significant impact on the computed WBSA. Figs 11(a) and 13(a) (see also Tables in S1 File) show the variation of the VBSA linear coefficient and the regression RMSE (Root Mean Square Error) as the azimuth angle change for different elevation angles.

For azimuth angle *θ* = 0°, the subjects appear with the maximum area (VBSA) of the body facing the camera, and the linear coefficient (vbsa) is at the minimum value. As the azimuth angle increases to *θ* = 90°, the VBSA decreases and the linear coefficient increases. At *θ* = 90°, the area facing the camera is at the minimum, since this is the angle where the camera can see only one side of the body. As the azimuth angle goes from 90° to 180°, the body shape is similar to the frontal part, but due to the body pose, the occluded areas make the difference: the VBSA increases, since the area facing the camera increases and the VBSA coefficient decreases. For the cross validation error, (Fig 11(a) left), the RMSE has a singular behavior. For all the azimuth angles *θ* ≤ 90° the error increases, but it reaches the maximum at *θ* = 60° and not at *θ* = 90° as predicted. This unexpected behavior is confirmed for the elevation angles *ϕ* = 30°, 45°, 60°. Intuitively the azimuth angle with the lowest accuracy should be *θ* = 90° because the body presents the least area to the camera. Instead, this is true for the angle *θ* = 60°. The explanation for this behavior is that, for this angle the body presents more overlapped areas. For the reciprocal angle *θ* = 150° (reciprocal with respect to 90°), this does not happen, because the arms are slightly bent upwards and hence, thus reducing the occluded area. This behavior does not happen for every elevation angle. At *ϕ* = 0°, the lowest accuracy is at *θ* = 90°. The reason is that, with the camera aligned with the body center the maximum overlap is observed at *θ* = 90°, but, as the elevation increases, less and less area faces the camera, thus more artifacts can appear.

**Fig 14:**
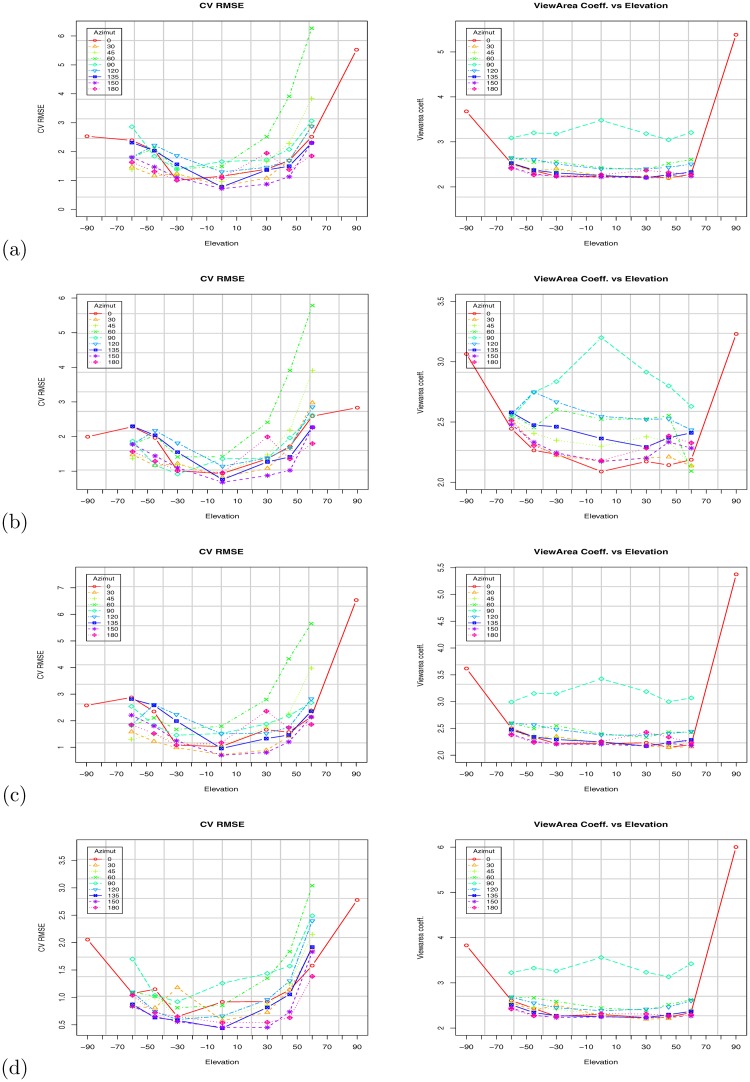
Results based on the Virtual Random dataset. Impact of camera orientation (Azimuth and elevation) on the VBSA prediction coefficient (right), and on the CV RMSE (left) computed WBSA. (a) All subjects. (b) All subjects with stature. (c) Adults Subjects. (d) Kid Subjects.

Varying the elevation angle, just as changing the azimuth angle, less area is viewed by the camera. But, differently from the azimuth case, this behavior is not linear and smooth as described above. In fact, observing the plots in Fig 14, we can see that the VBSA coefficient does not always increase linearly. See the results at *θ* = 30°, 45°, 60°. For these angles, the overlapped areas due to the left arm and leg play the role of a non-linear component making the relation to diverge from linearity. The intercept has a maximum at approximately *θ* = 60°. The intercept is associated with the bias of the prediction, an offset to add at the linear increase of the VBSA. The WBSA and VBSA are linearly correlated. For *θ* = 60°, there are many occlusions, and the legs are overlapped. In this situation we register the highest prediction error.

To evaluate the prediction, we used a *k*-fold cross validation setup. We randomly compose the folds using the function from the CVTOOL package in R [61]. We measure BSA prediction performance in terms of prediction errors: root mean square error (RMSE) under *k*-fold cross validation, denoted as CV RMSE in the tables, but also CV MSPE (mean squared prediction error), and CV MAPE (mean absolute prediction error). Since the WBSA and VBSA are calculated from a mesh with the base unit in decimeter (*dm*), the WBSA is in decimeter squared (*dm*^2^). The cross validation root mean square error (CV RMSE) is in decimeter squared too, while the cross validation mean absolute prediction error (CV MAPE) is in percentage (%). CV RMSE varies from maximum value of 5.5 *dm*^2^ when the camera is at the unfavorable position of 90° in elevation, to a minimum of 0.71 *dm*^2^. These can be considered relative to the average WBSA, for the virtual dataset, average is 167 *dm*^2^ and the relative error is 0.6%. The behavior of the CV RMSE is not straight forward, but it seems connected with the occluded areas and consequently with the position of the camera. In all the predictions, CV RMSE was higher for the positions of the camera in front of the subject (azimuth 0–90) and generally has a high value at around *θ* = 60°. The highest value, however, is for the elevation of 90° where the camera can see mainly the footprint of subject. In this case the stature component is totally missing from the image, but the prediction can still get a decent estimate for obese subjects.

### 3.6 Regression with stature

Since the WBSA is dependent on the stature and given the high correlation, (see Table 2), we expect improved results by including the stature in the prediction model (Eq 8). Figs 11(b), 12 and 13(b) show the impact of including stature in the model. In general, we obtain a lower prediction error for most analyzed angles, as can be seen in Fig 12, though the improvement may not be as significant in some cases, e.g., as azimuth angle *θ* moves away from 90°. Although it is not always possible to acquire the stature with accurate precision, this is not the case in a physician’s clinic, where the controlled environment, always permits the detection. However, in a more unconstrained environment, we should consider the stature detection error and its influence on the WBSA estimation. This is out of the scope of this work.

### 3.7 Regression with grouping

**Fig 15:**
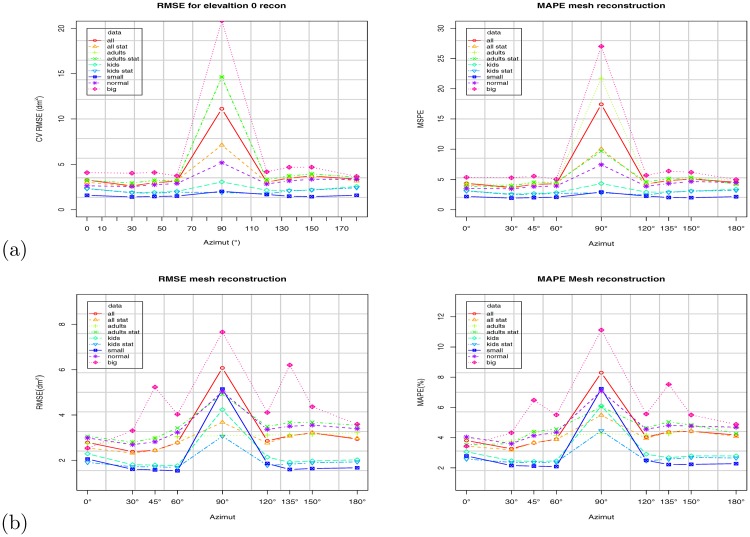
Mesh reconstruction results at elevation angle *ϕ* = 0°. (a) Virtual Random dataset; (b) Virtual NHANES dataset.

We investigate the performances of the system for different specified human categories. We grouped our virtual subjects in 5 different classes (males/females, adults/kids, small/normal/big stature), and we use these partitions to learn the linear system. Then using 10-fold cross validation we compute the prediction error. Figs 11–15 show the VBSA coefficient, the RMSE error, and a comparison of the errors for cases with and without grouping. Grouping has a different effect on the prediction error. Surprisingly, grouping did not always lead to an improvement. In fact, the errors for adults seems larger than the error obtained for all subjects. Instead, we have some improvement for kids, small stature and normal stature. Using the stature in the group models results in a significant improvement for most groups (see Figs 11 and 13).

## 4 Discussion

In this work, we presented an integrated computer vision framework to infer the relation between the Whole Body Surface Area (WBSA) and the View Body Surface Area (VBSA) for a given viewpoint of the subject. In this section we discuss some observations from the obtained results. Fig 11, 13(b) and 12 show the WBSA prediction errors for different experimental settings using the two datasets. Other plots that show the WBSA error behavior can be found in S1 File. In all the WBSA prediction plots, we can see a logically intuitive pattern: the error remains low for azimuth angles between 0 − 45° and 135 − 180°, but higher for the angles 60 − 150°. From this common behavior, we discuss a number of interesting observations, some of which are not so apparent. Some of these are very difficult to observe.

### 4.1 Frontal VBSA Vs Rear VBSA

An interesting observation is the difference in behavior of the VBSA (and hence computed WBSA) when the subject is viewed from the front or from the back. As reported in [1] and [24], the front accounts for more than 50% of the total WBSA. As we can see from the RMSE (Fig 11(a) and 11(b)), the errors from the rear part are always inferior relative to those from the corresponding angles from the front. There could be several explanations. Since the hands are bent slightly upfront, the occlusions are greater seeing the mesh from the front. Moreover, the frontal part of the human body and consequently the mesh has many more curved surfaces in the front, and hence more challenging to model. This can be noted by comparing the RMSE from males and females: despite some irregular behavior (males have higher RMSE at *θ* = 90°, than at *θ* = 60°) the average RMSE for the males is lower, for the frontal angles (0° ≤ *θ* ≤ 90°), than for females.

### 4.2 Non-Linearity in the WBSA-VBSA relationship

**Fig 16:**
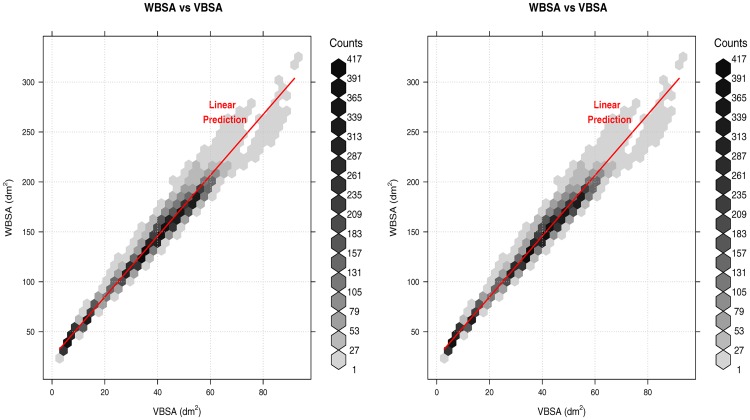
Non-linearity in the VBSA-WBSA relation at *θ* = 60° *ϕ* = 60°. (Left) Virtual Random dataset. (Right) Virtual NHANES VBSA-WBSA dataset.

Figs 11, 12 and 13 show the RMSE increases as the azimuth angle approaches 90°. The same behavior can be seen as the elevation angle *ϕ* approach ±90°. Intuitively, for these angles the VBSA can hardly infer the WBSA of the subject. In these situations, there are many overlapped areas other than the usual occlusions (feet, armpit, crotch, etc), obtaining an higher error in linear prediction thus the VBSA-WBSA relation diverges from linearity. Fig 16 shows the VBSA-WBSA relation for the two datasets at *θ* = 60° *ϕ* = 60°. Observe that for these angles the homoscedasticity condition (constant variance) fails. People with small WBSA have small variance, while big people have very large variance. In this situation, a linear model can still be used, assuming that we accept a slight decrease in performance, for subjects with small WBSA, but it will strongly impact the performance for high WBSA subjects. Fig 12 shows the performance of the linear model for different categories. For high and very high WBSA values, a different approach (non-linear) has to be considered.

### 4.3 Evaluating WBSA Measurements

Measuring the WBSA of real people is very challenging, often laborious, cumbersome, and inconvenient to the subject (e.g. mold of Paris wrap). Thus the availability of ground truth data in WBSA studies is always a problem. In these kind of studies real people have to be measured to constitute the ground truth. Our method, instead, uses the ground truth of virtual subjects. We generate these subjects making sure that they are very close to real people. We proposed a generative data-driven approach, commonly used in computer graphics and more recently in AI and machine learning environments [41, 62]. The proposed framework can simulate a real environment with real subjects, and real devices, considering all the possible distortions, and artifacts present in the real process. In this virtual environment, we have full control of the different components, and this is ideal for the study of the real-life process, and to learn machine learning algorithms. However, we still cannot compare our learned algorithms with traditional methods (e.g. formulae, and 3D scanner), due to the scarcity of ground truth data. For instance, the direct comparison of the learned models with the formulae will be an ill posed problem, since we don’t know which method is closer to the real measure. Using a simultaneous 3D scanner and Microsoft Kinect acquisition will give the same ill posed problem. This situation, however, can be useful for comparing the learned system to the real scenario. Although, it’s very important to understand the accuracy of the 3D scanner, and the setup used in the acquisition, unfortunately, we don’t have this capability at the moment, and we encourage any research lab to test and improve our framework, which is built from open source tools.

### 4.4 Reconstruction

As explained in Section 2.4.1, the WBSA retrieval is based on the triangular mesh area calculation. The subjects in the datasets are already represented as a mesh with the WBSA calculated from the MH plugin. However, the ray cast result is a point cloud as shown in Figs 3 and 8. A fundamental step in determining the VBSA is surface reconstruction. Since surface reconstruction is a hot topic in computer graphics, and is beyond our goals in this work, we decided to study its impact with just one known algorithm: the greedy surface reconstruction algorithm [58]. In this experiment, we use the default setting that should give good results.

**Fig 17:**
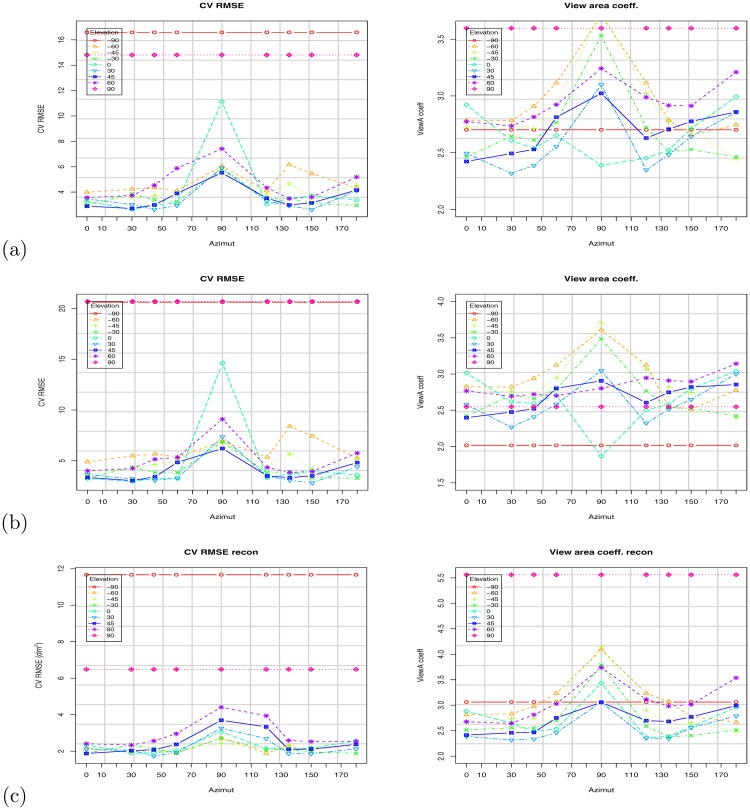
Results based on Virtual Random dataset. Impact of mesh reconstruction error on WBSA prediction for given camera orientation (azimuth and elevation): Input VBSA prediction coefficient (right), and on CV RMSE (left): (a) all subjects. (b) adult subjects. (c) kid subjects.

Before applying the reconstruction algorithm, an intermediate step is the points normal calculation. For this, we used an algorithm based on integral images [63] implemented on the PCL library. We estimated a reconstruction time of ≈ 0.5*s* on average for one subject (subjects with more overlapped areas slow down the algorithm). Fig 15 shows the cross validation error for linear regression using the VBSA computed from the reconstructed surface for all the subjects. Fig 17 shows the impact of the reconstruction error on WBSA prediction for the given camera orientation, and for different class of subjects in the Virtual Random dataset. We observe that, at the indicated elevation angle *ϕ* = 0°, the errors are still generally lower for the azimuth angle 0° ≤ *θ* ≤ 45° and 135° ≤ *θ* ≤ 180° (less than about 6% for MAPE, and less than about 5.0 for RMSE). These results show the reconstruction error as an additive noise on the VBSA. This noise is composed by two main components. The first is the error due to the points normal computation. In fact, errors in the normal direction will impact the subsequent surface reconstruction. Unfortunately, due to the very complex nature of the human body, and the additional complexity due by the perspective view in the ray cast operation, computing the normals is not that easy. Missing neighboring points, surfaces with weird angles due to the non-rigid nature of the body make this operation more complex and prone to errors. Fig 8(d) shows the result of the normal calculation. The surface reconstruction operation is the second source of noise. This basic operation is responsible for transforming a raw or basic representation of the subject (i.e. cloud of data points) into a closed manifold mesh. One of the main challenges to surface reconstruction algorithms is hole filling. A hole in the mesh structure is possibly caused by gaps in the mesh structure, which if left untouched would result in a surface with numerous jagged boundaries. This phenomena is the main source of error in the surface area calculation. In fact, since we just compute the areas of the single triangles, erroneous reconstruction will add boundaries that increase the calculated surface area. This behavior has been observed during the software setup. To correct this effect, we have performed smoothing with least mean square and sampling with voxels before the final reconstruction.

Usually, every surface reconstruction algorithm, is tested using SSD or similar measures. Unfortunately, since the surface area computation is based on triangle area computation, the usual measures don’t always consider the reconstructed topology of the final mesh. These distortions in the topology can drastically degrade the surface area calculation.

All these methods are accurate and can produce a reliable surface, however, they need a significant amount of time to reconstruct the partial surface of the subject. Simulating a depth sensor, gives us what is called an organized point cloud (the (x, y, z) points are organized in a matrix fashion like the pixels of an image), and we can use faster and simpler methods for the reconstruction, for example [64].

## Supporting Information

S1 File Supporting Information to the manuscript. Complete results of the Virtual Environment and linear regression.
(PDF)

S1 Fig Relationship between WBSA and VBSA. (Left) Using Virtual Random dataset at *θ* = 0°, *ϕ* = 0°. (Right) Using Virtual NHANES dataset at *θ* = 0°, *ϕ* = 0°.
(TIF)

S2 Fig Residuals analysis. (a) QQ plot of residual. (b) Residuals. (c) Residuals histogram. (d) Residuals scale plot.
(TIF)
