# Supplementary material for: A Framework for Analyzing the Whole Body Surface Area from a Single View
Source: PLoS One. 2017 Jan 3;12(1):e0166749. doi: 10.1371/journal.pone.0166749 (PMC5207503; doi:10.1371/journal.pone.0166749)
Supplement: S1 File — Complete results of the Virtual Environment and linear regression. (PDF) [file pone.0166749.s001.pdf]

Supporting Information:

A Framework for Analyzing the Whole Body Surface Area from a Single View

Marco Piccirilli, Gianfranco Doretto, Donald Adjeroh
Lane Dept. CS and EE, West Virginia University, Morgantown, WV, USA
∗ E-mail: Corresponding mpiccir1@mix.wvu.edu,
Gianfranco.Doretto@mail.wvu.edu, Donald.Adjeroh@mail.wvu.edu

Regression model validation

To verify that the linear regression model is valid we plot some useful quantities. Figure S2 Fig(a) shows the normalized QQ plot of the residuals. As we can see, we can fit a straight line between the first and third quartiles. This means that the normality condition is verified for the residuals. Figure S2 Fig(b) shows the residual-leverage plot. The leverage is very low ($\sim 10^{-4}$). That means that the outliers are not distorting the linear model. There is a point with high negative standardized residual. This point doesn't affect the overall result. Figure S2 Fig(d) shows together with Figure S1 Fig that the assumption of homoscedasticity (constant variance) required to use the OLS (Ordinary Least Squares) estimator is verified. We calculated this quantities for ($\theta = 0°, \phi = 0°$) position of the camera, we'll see later that for some position of the camera the model will diverge from the linear model, and will see if a linear model still can be used with good performances.

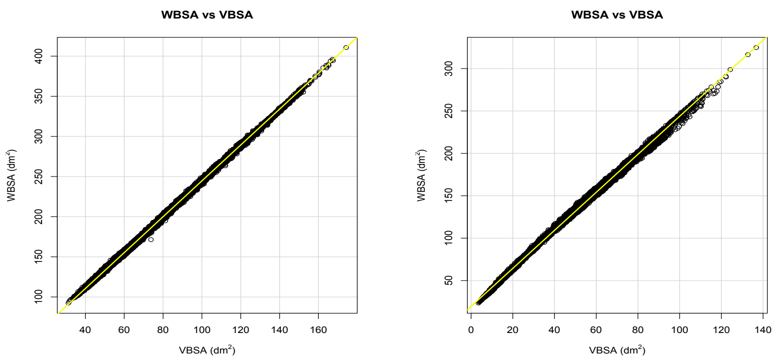

S1 Fig. Relationship between WBSA and VBSA. (Left) Using Virtual Random dataset at $\theta = 0°, \phi = 0°$. (Right) Using Virtual NHANES dataset at $\theta = 0°, \phi = 0°$.

As we can see from Figure S1 Fig the relation is linear and can be easily fitted. The values for Multiple R^2 and adjusted R^2 are very high (0.9975)

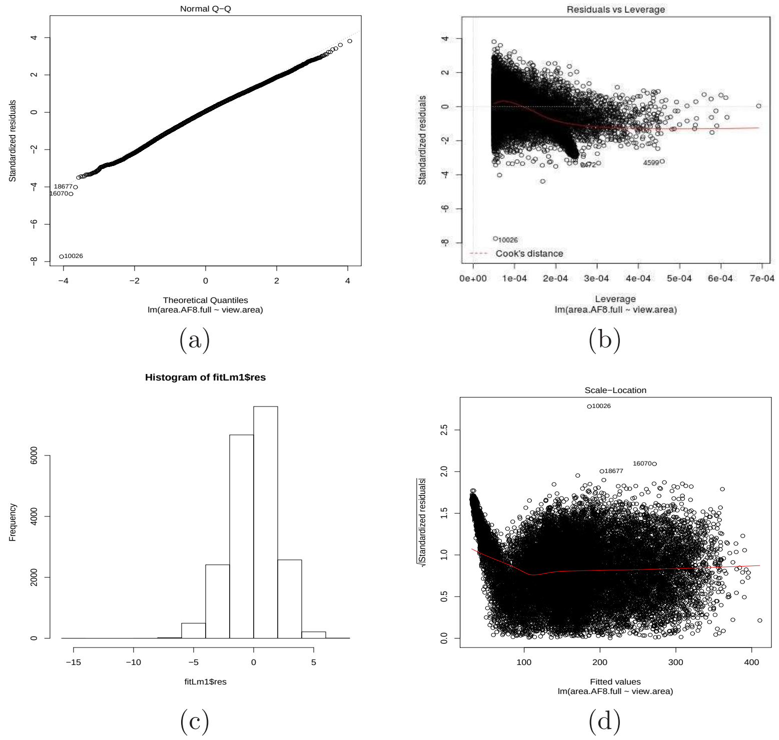

S2 Fig. Residuals analysis. (a) QQ plot of residual. (b) Residuals. (c) Residuals histogram. (d) Residuals scale plot.

that means that the residuals are closer to the linear model (low variance), but doesn't tell much about the best fit. From Figure S1 Fig we can see that the residuals are close to the median value, and hence the R^2 value is high, but in this situation, we cannot use this value to establish which prediction is better. A more useful statistic is the standard error (se) of the residuals. Also indicative is the distribution of the residual given by min max 1st and 3rd quantile. The linear regression model is validated using the QQ plot of the residuals, and by checking that the assumption of constant variance (homoscedasticity) needed for least mean square estimation is met.

Additional Tables

Table A. Body measurements entries for the NHANES dataset (Sample of 500 subjects)

SEQN	RIAGENDR	RIDRETH1	BMXWT	BMXHT	BMXBMI	BMXLEG	BMXARML	BMXARMC	BMXWAIST	BMXTHICR	BMXTRI	BMXSUB	AGE
3	2	3	32.9	136.6	17.63	34.1	25.5	19.7	64.7	38.2	8	6.5	10.4166666667
5	1	3	92.5	178.3	29.1	45.2	39.7	35.8	99.9	56.2	17.4	38.6	49.75
6	2	5	59.2	162	22.56	39.7	34.5	26	81.6	47	20.3	16.8	19.1666666667
7	2	4	78	162.9	29.39	43	38.1	31.7	90.7	55.7	26.4	34.2	59.3333333333
8	1	3	40.7	162	15.51	41.6	36.7	20.1	64.1	39.1	6.2	5.2	13.25
9	2	4	45.5	156.9	18.48	42.2	32	22.1	64.6	45.3	11.1	7.6	11.0833333333
10	1	4	111.8	190.1	30.94	46.6	43	37.6	108	64	15.5	26.6	43.1666666667
11	1	3	65	171.9	22	40.5	37.2	29.2	76.5	39.9	8	7.8	15.25
20	2	1	59.8	158.9	23.68	42.2	34.5	26.1	81	46.6	15.6	17.4	23.75
21	1	1	112.9	168.5	39.76	42.3	36	33.5	125.7	61.4	15.4	38	18.5833333333
22	2	4	50.9	162.2	19.35	42.7	37.3	22.8	66.9	45.2	12.2	9.5	13.25
23	2	4	75	169.1	26.23	42.8	38.8	32.8	92.6	56.3	25.4	21.6	12.25
24	2	3	69.9	164.2	25.93	35.5	36	30.2	90.6	51.2	22.4	21.3	53.6666666667
25	2	3	104.5	166.7	37.6	31.4	38	42.6	114.5	59.7	26.4	38.8	42.3333333333
26	2	1	85.6	163.1	32.18	42.1	40.2	35.9	96.3	58.5	31	21.3	14.3333333333
27	1	1	72.2	161.3	27.75	38.8	38.8	29.8	100.7	50.6	10.6	18.8	18.75
28	1	3	62.7	179.6	19.44	43	36.7	26.4	73.4	47.5	9.2	8	18.9166666667
31	2	1	47.5	164	17.66	40.5	31.2	22.5	67.8	44.8	10	11.4	15.75
32	1	4	43.5	151.1	19.05	34.8	31.7	23.8	64.4	42.6	6.6	6.3	10.75
34	2	4	67.4	162.2	25.62	41	37.5	30.5	85.6	52	22.7	20.7	38.25
40	2	1	67.2	149.1	30.23	25.8	34.6	35.5	99.3	45	28.4	26.2	68.9166666667
42	2	4	84.2	163.7	31.42	40.5	36	32.7	99.8	58.1	22.8	37.6	18.5833333333
44	2	3	61.3	162.5	23.21	39.6	36.6	27.8	76.8	51.5	15.4	16.1	15.3333333333
45	2	1	75.8	166.1	27.47	42.6	36.1	33.4	87.7	55.9	24.8	22.2	33.75
52	2	4	57.4	165.4	20.98	42	36	28.2	71.7	50.4	15.2	10.4	0.25
56	1	3	81.7	182.1	24.64	42.4	39.4	31.4	92.5	52.4	11.4	16.5	61.3333333333
57	1	3	88.6	172.6	29.74	39	38	35.4	110.4	53.3	13	24.2	21.75
58	2	1	57.1	154.1	24.05	34.2	33.6	28.1	81	45.5	13.9	18.9	39.5833333333
59	1	1	95.3	180.4	29.28	44	41.5	32.1	107.5	51.6	9.4	15	18
62	2	5	41.5	154.6	17.36	35.8	32.8	23.9	63.2	42.8	13	10.4	4.8333333333
64	2	4	54.2	162.7	20.48	38.2	35.4	24	73.9	42.9	20.3	10	0.5833333333
65	1	5	37.9	139.5	19.48	32	29.2	22.3	67.8	42.7	13	9.5	63.8333333333
66	1	1	81.8	164.1	30.38	36.1	36.1	40.4	95.1	53.9	12.8	28.6	9.8333333333
67	1	1	48	143.5	23.31	36	34.6	26.8	76.4	44.3	16.2	12.2	37.75
68	2	1	57.8	155.4	23.93	39	33.7	28.5	79	49.8	25	19.4	13.75
72	2	4	54.1	168.8	18.99	43.6	36.5	24.6	72	45.1	12.2	7.9	3.3333333333
74	2	1	49.2	157.3	19.88	36.6	33.5	26	68.4	46.6	14.8	10.2	15.9166666667
75	1	1	27.2	134.5	15.04	30.1	29.7	19.3	61.4	33.2	15	6.2	7.25
76	2	1	55.6	164.2	20.62	42.5	34	26.8	70.5	46	9.4	11.2	13.1666666667
77	1	3	80.7	176.7	25.85	43.7	40.4	33	93	52.4	15	26.2	8.8333333333
78	1	3	39.3	151.8	17.05	39.6	32.5	21.9	65.4	40.3	10.4	6	13.9166666667
81	1	3	90.1	177.2	28.69	42.8	38.5	34.2	102.2	57.9	18.4	28.2	2.4166666667
83	2	4	90.5	167.8	32.14	40.4	38	34.5	111.4	55.1	29	28.9	30.3333333333
84	1	3	51.1	166.5	18.43	40.2	36	23.7	70.7	43.4	6	6.2	3
85	1	1	79.9	165.6	29.14	39.5	37.1	33.8	93.7	57.6	16.2	18.4	60.9166666667
87	2	1	56.8	157.3	22.96	38.3	33	25.7	75.3	48.5	16	14.4	18.6666666667
88	2	1	42.8	143.3	20.84	33	30.3	23.7	73.8	43.6	19.6	19.4	63.3333333333
96	2	4	70.6	160.1	27.54	39.9	35.6	33	78.9	56.5	23.4	14.8	17
97	1	4	87	175.1	28.38	48	36.2	35.7	88.3	59	6.2	10.6	31.25
101	1	1	70	172.4	23.55	43.4	38	30.1	91.8	47.3	14.1	13	61.4166666667
102	2	5	63.4	155.2	26.32	38.4	33.6	34	80.9	53.3	26.4	27.8	45.4166666667
106	1	1	71.84	157.2	29.07	40	35	34.2	92.5	52.5	13.8	25	0.3333333333
109	2	3	48.8	160	19.06	40.4	34.4	24	70	45.4	13.8	10.4	41.0833333333
110	1	1	32.7	145.9	15.36	34.5	31	19.6	56.5	38.4	12.2	6.4	3
112	1	1	61.2	167.3	21.87	41.3	36.5	28.6	73.8	50.4	8.8	13	37.3333333333
113	1	3	77.7	181.7	23.53	44.6	40	32.7	83.5	51.5	5.2	10	39.9166666667
114	1	3	83	175.4	26.98	44.3	38.7	33.5	92.2	58.1	22.8	22.2	30.6666666667
115	2	3	56.6	164.6	20.89	36.6	33.8	27.8	76	45.7	25.2	17.5	22.1666666667
120	2	3	77.6	174.1	25.6	48.3	38.2	31.2	88.7	54.7	32.2	35.4	27.8333333333
121	1	1	81.6	169.5	28.4	39.4	35.9	33	92.9	52.8	9.7	19.2	14.5833333333
124	1	1	54.5	167.4	19.45	38.3	35.3	24.3	71	46.1	7.8	7	34.5
128	2	2	29.6	131.5	17.12	31.5	24.5	21.2	58.3	39.4	9.2	9	0.3333333333
130	1	3	76.6	171.1	26.17	42.6	38.2	30	94.9	50.5	12.8	12.4	38.5833333333
131	1	3	88.3	181.4	26.83	40.3	38.6	34.9	97.1	53.7	13.2	25.9	64.9166666667
132	2	3	125.2	172.8	41.93	38.2	40.8	38.2	132.6	57.7	24.2	24.2	76.5
135	1	4	55.1	151.9	23.88	41.8	33.9	28.3	78.7	50.4	30	24.4	39.5
139	2	4	53.9	157.2	21.81	39.4	35.4	25.2	73.8	48.2	14.2	13.8	8.4166666667
140	2	5	93.3	168.7	32.78	43.2	33.6	38.5	94.7	63	36	30.2	63.75

Table B. Virtual Environment result for the Virtual Random $\theta = 0°$ $\phi = 0°$ (only 70 subjects).

filename	Stature	WBSA	VBSA	View cells	Tot Cells	gender	Age	hips circ	waist circ	bust circ	underbust circ	neck circ	frontchest	upperarm lenght	upperarm circ	lowerarm lenght	wrist circ	shoulder dist	upperleg height	thigh circ	calf circ	ankle circ
1-0.0	8.11084	44.4981	11.7776	2986	28792	0	3.1854	47.6972	40.4591	44.8797	40.2419	22.3629	13.3064	9.8063	14.0304	11.8465	8.3803	3.7521	16.3638	27.1634	18.1661	11.5497
2-0.0	19.779	195.852	76.5401	5319	28788	0	12.4205	99.19	74.4447	85.7825	71.5506	33.1097	28.0021	23.7443	25.0241	26.7819	14.7358	9.9943	44.2876	53.5784	37.6602	22.6656
3-0.0	15.504	159.91	63.8571	5062	28792	0	28.4965	92.4454	72.7132	109.0005	79.0535	37.7841	28.9144	16.7136	32.4551	19.829	13.8644	7.1632	35.3609	54.9589	37.6159	16.9985
4-1.0	13.679	121.377	44.4295	4756	28796	1	12.5267	80.7306	64.4248	84.4454	73.6152	34.2688	26.9519	16.4165	24.2976	17.4372	13.0222	7.0102	29.4875	44.5917	31.7901	18.6163
5-1.0	8.3492	42.943	10.8167	3026	28784	1	3.8627	43.3066	34.849	44.9569	42.2694	20.2488	13.3554	10.4466	11.1945	11.7983	7.2408	4.2415	16.8886	21.192	15.8614	11.3731
6-0.0	16.52	147.376	57.0991	4953	28792	0	11.3646	86.0719	64.4382	79.4706	65.9223	30.585	25.1375	19.6052	24.7341	22.6557	12.8617	8.0505	37.5691	48.5264	33.1886	19.1004
7-0.0	20.977	224.316	91.1745	5697	28788	0	63.9601	111.2573	81.7125	96.2799	77.6798	35.2717	31.0804	25.2396	27.086	28.3524	15.7224	10.4356	46.1136	56.1296	40.8358	23.1267
8-0.0	15.26	147.559	57.0255	5105	28796	0	39.1227	96.1499	71.9456	87.6356	70.8275	34.3382	28.1127	16.0866	25.9621	18.7039	13.017	8.4125	31.2725	51.9997	36.0232	17.688
9-0.0	17.282	157.162	59.759	4986	28796	0	10.3877	89.3878	67.1335	79.3362	66.065	30.8924	25.577	20.63	23.7118	23.6365	13.1729	8.7753	38.9281	49.2401	34.1717	20.2168
10-0.0	24.086	263.018	106.85	5872	28792	0	23.8362	108.9459	80.2397	106.4404	89.7884	36.5535	32.2658	28.0623	24.5952	31.8684	17.3073	12.3434	54.8359	54.0202	39.3684	24.8432
11-1.0	14.458	127.711	47.4913	4865	28796	1	15.847	81.5642	64.3193	83.5418	72.8648	33.2631	27.7	17.6795	22.3396	18.7529	12.4506	7.5021	30.2905	43.0897	31.6468	19.5677
12-1.0	17.74	165.223	63.2309	5277	28796	1	22.4875	85.0941	63.6326	86.6848	79.4163	30.9249	29.011	22.0796	22.4136	25.1479	14.2439	9.8989	38.4104	42.3831	32.823	21.7908
13-0.0	11.709	77.3293	25.2628	3946	28796	0	6.6205	63.2911	49.4971	54.1244	49.6383	23.9175	17.8748	13.8718	15.3882	16.1658	9.6221	6.3718	24.7278	32.8273	23.449	14.6412
14-1.0	20.528	209.777	82.8631	5580	28788	1	10.8373	96.8014	77.9618	92.5998	84.925	36.7835	29.5641	26.0999	24.5776	27.5662	15.1664	9.6054	46.6518	50.2421	37.0675	24.9551
15-1.0	8.4173	52.6058	14.8298	3360	28788	1	4.669	53.0206	44.8767	53.9905	48.0526	26.261	16.332	10.0339	15.6334	10.4869	8.9016	4.0447	16.1227	29.7535	20.1315	12.9421
16-1.0	11.442	79.3825	26.56	4037	28792	1	4.276	61.36	52.2033	61.0663	54.488	27.9837	18.4936	14.442	18.9359	16.3255	10.8466	5.0594	23.9801	35.0845	23.8714	15.4815
17-1.0	23.906	295.676	123.566	6117	28784	1	46.634	119.2637	93.8469	118.7251	103.41	45.1344	37.6916	29.2638	32.5796	32.7745	18.7814	11.1739	55.2293	64.4297	45.7259	28.8661
18-1.0	17.06	189.208	76.5853	5452	28792	1	53.8395	101.0656	80.7138	113.6429	94.2689	42.2197	35.3724	20.5256	34.1153	22.4679	16.8074	8.5187	38.883	57.0177	40.7397	22.1251
19-0.0	8.3997	49.1279	14.0025	3202	28788	0	4.2715	51.0272	42.5336	50.1922	42.9434	23.0911	14.6823	9.9277	15.8564	12.2037	8.5715	4.0714	17.3729	29.6829	19.5865	11.693
20-1.0	13.13	117.986	42.5657	4707	28796	1	11.5642	85.6734	72.6585	83.8472	75.9735	34.7134	25.5653	15.9545	22.8194	15.2709	12.5359	6.8336	27.763	45.3692	31.3796	18.677
21-0.0	14.028	114.17	41.6436	4529	28796	0	18.0319	80.5688	60.2351	70.956	60.3474	27.9008	23.5437	15.2309	21.0546	18.7623	11.0962	7.4391	30.7073	44.1625	30.575	15.6917
22-1.0	14.7	142.984	54.8262	5149	28796	1	18.772	88.5984	70.1045	93.4659	80.7512	37.896	29.6535	17.3967	26.2742	18.7307	13.9994	7.7076	30.5167	48.9193	34.2688	20.1861
23-1.0	17.011	166.537	65.2533	5226	28796	1	67.1448	96.8619	76.4513	97.6554	84.5428	35.7119	32.0097	19.6984	23.918	21.8574	13.566	9.1611	37.8955	47.2666	36.657	22.7758
24-1.0	10.513	79.3682	26.0425	4105	28792	1	7.6232	66.7205	55.0126	67.4496	60.2466	30.5801	21.039	12.1463	19.181	11.9178	10.706	5.5327	21.396	36.528	25.6007	15.7194
25-1.0	6.81	37.7603	8.7955	2708	28784	1	2.7776	44.9534	39.6211	45.3628	40.9583	23.986	13.1318	8.0348	13.8022	8.486	8.0719	3.1382	12.6188	25.7734	16.9836	11.1731
26-0.0	13.736	113.557	42.0991	4492	28792	0	16.3539	78.4974	59.1878	75.563	62.6754	28.9019	23.5111	15.1981	23.7854	19.0861	11.2928	6.741	31.2322	44.9639	30.6538	15.3593
27-1.0	16.199	157.071	60.7694	5154	28796	1	27.7513	90.0942	70.0895	94.4554	83.5227	35.8553	30.5724	18.6658	24.701	22.7851	14.0083	8.7053	36.0518	48.5572	35.6935	21.3509
28-0.0	15.804	150.67	58.1298	5072	28796	0	69.4552	98.7846	71.192	91.6774	68.6139	32.4447	27.3875	18.4798	24.3521	22.0025	12.8217	8.5632	32.6514	48.8874	35.7529	18.0756
29-0.0	13.765	114.733	41.5791	4584	28796	0	18.1698	81.2395	60.6295	76.6462	62.261	29.3637	23.948	14.6837	19.9952	16.6249	11.2224	7.1002	30.3705	44.4227	30.6273	16.0592
30-1.0	17.285	208.125	83.5473	5486	28796	1	30.6009	122.8793	106.8761	115.7709	104.3986	42.1631	34.536	22.156	36.1246	22.2645	19.8721	9.0332	38.2914	65.3793	41.5814	23.4309
31-1.0	16.313	175.003	68.5579	5332	28796	1	86.4767	110.3801	93.3344	106.4812	95.1601	40.5987	31.6902	17.7015	25.7018	18.0104	14.4872	9.122	36.8786	54.4092	39.0156	23.0935
32-1.0	16.98	168.469	65.6098	5237	28796	1	44.5096	95.9101	75.1527	94.9469	81.0783	34.9977	32.8621	20.8035	25.7597	21.1519	14.4804	8.9737	38.3225	47.9376	35.8893	22.7103
33-1.0	14.933	136.444	50.672	4894	28796	1	17.6606	84.587	66.2279	86.8994	76.0512	34.335	28.7486	17.8434	22.7908	19.0599	12.967	7.9158	32.6745	44.6595	32.9632	20.2921
34-1.0	17.584	189.105	75.1906	5393	28796	1	23.8896	100.5406	78.5187	104.0456	90.0332	39.2002	33.7403	21.069	30.9272	25.2987	16.7652	9.0994	39.6707	55.8778	39.1503	22.6234
35-1.0	10.683	73.4883	23.4179	3960	28792	1	7.2452	61.204	51.4305	61.2611	56.8133	27.0321	18.8507	12.813	15.6169	13.3198	9.2848	5.551	22.2608	31.4504	23.1653	15.0221
36-1.0	16.254	165.721	63.3617	5338	28796	1	28.3426	93.9678	72.5002	97.0539	86.6207	37.8543	31.7662	18.2489	24.8023	20.4188	14.6656	9.2979	35.772	49.4193	36.4099	22.4882
37-1.0	17.904	188.402	74.8614	5348	28796	1	62.9301	103.9837	83.8382	103.2828	91.7687	38.8421	32.5228	20.3215	26.4765	24.7277	14.8516	9.5823	40.1294	53.9656	39.4766	23.5013
38-0.0	23.997	288.392	119.959	6062	28788	0	58.4003	127.746	93.81	105.5715	90.875	39.8529	34.986	28.6129	29.2822	32.2156	17.7687	11.7742	53.1394	64.9337	45.0461	26.3136
39-1.0	15.645	165.191	65.2877	5333	28792	1	31.5775	93.7343	73.8037	105.5257	88.1505	40.4714	33.3842	18.7142	32.8386	20.1261	16.2991	7.8422	34.3192	54.0622	37.8503	20.7786
40-0.0	7.7957	48.3264	12.9603	3154	28788	0	5.1191	53.3545	44.7171	50.1485	42.8091	24.6948	14.7163	8.3745	15.2105	8.3579	8.4959	4.3692	14.6919	29.8366	19.9429	12.0752
41-0.0	19.135	192.169	76.1068	5329	28796	0	39.4082	105.5664	78.5222	86.999	73.4744	32.9718	29.4123	21.4995	24.8558	25.2372	14.2951	10.2347	40.9569	54.4034	38.5136	20.7973
42-0.0	21.67	248.125	102.191	5719	28788	0	30.919	114.8219	86.8959	102.3706	84.7341	38.0794	32.4788	25.0513	31.6857	29.1193	17.3172	10.5884	48.3724	63.0601	43.5346	24.3774
43-0.0	7.7529	42.1131	10.5449	2914	28788	0	3.8549	47.2084	38.8326	43.3459	39.0304	21.6427	13.0692	9.0608	12.951	10.5179	8.1364	4.0177	15.0199	25.8018	18.0981	11.3472
44-0.0	23.46	268.995	110.338	5823	28788	0	23.4199	114.4564	85.7234	106.2403	86.765	38.6842	33.2325	27.7541	30.0235	31.8991	17.0564	10.3284	54.2665	63.2878	43.3464	24.6675
45-1.0	17.397	172.172	66.8454	5285	28796	1	40.2055	94.6448	75.9553	94.4842	86.2632	34.5561	30.4441	20.5263	23.8726	24.2517	14.234	9.5798	39.5244	48.4203	35.7522	22.2748
46-0.0	20.658	236.814	96.0402	5721	28792	0	89.6771	118.496	85.376	111.904	83.0092	38.9946	31.8243	26.1382	31.1663	28.7984	16.5081	10.0752	45.8373	58.8138	42.8634	23.9439
47-0.0	12.381	94.1925	31.6455	4326	28796	0	15.1677	74.8012	55.0875	61.601	53.9532	26.1893	21.6709	12.865	16.8607	13.3423	10.2995	7.9255	24.6687	37.4236	27.6286	15.506
48-1.0	20.019	234.661	96.2497	5733	28796	1	35.4818	109.296	85.8375	115.3749	97.8139	43.1753	36.6763	24.6063	34.2192	28.0841	18.099	9.7249	45.8058	61.0504	42.8051	24.9544
49-0.0	14.259	132.62	50.4356	4886	28796	0	21.5134	89.82	67.826	87.2375	68.1994	33.2145	26.2983	14.5419	25.0821	16.5322	12.5161	7.807	30.3395	50.1517	33.9374	17.0097
50-0.0	13.363	120.698	44.4165	4727	28796	0	18.6118	89.5552	69.056	78.4986	65.6338	31.5922	25.0318	13.3531	22.939	14.4746	12.3938	8.0498	26.8985	48.2444	32.7606	17.9247
51-0.0	22.287	233.934	94.9871	5831	28796	0	65.5776	105.987	77.2322	104.8064	85.6053	35.0533	30.7741	26.8577	24.1782	29.92	16.3143	11.3034	50.4945	50.3618	37.608	23.868
52-1.0	23.505	282.363	117.657	6027	28792	1	57.0971	118.2767	92.335	115.6159	99.8553	42.5877	38.2194	28.9431	30.623	31.0661	18.0572	11.3461	54.6935	60.4107	44.8526	29.0988
53-1.0	16.268	157.346	60.8888	5096	28796	1	82.4548	96.3686	77.5218	97.2019	86.0271	36.6841	30.0446	17.3365	22.027	19.7439	12.4945	9.0235	36.9624	47.5021	36.547	22.0522
54-0.0	12.584	90.8365	31.3272	4192	28788	0	7.8516	69.0182	52.9451	61.4784	53.0466	25.7888	19.8677	14.8606	18.454	18.0174	10.4332	6.4595	26.7484	37.9584	26.4019	15.5398
55-0.0	15.269	145.137	56.3061	4947	28796	0	23.0269	91.3479	69.5435	87.0048	71.8861	33.8819	27.7864	16.092	27.3078	19.7066	12.9894	7.9912	32.7284	52.0478	35.581	17.0935
56-1.0	17.124	198.766	78.6628	5383	28796	1	49.0141	120.4635	107.2564	113.792	103.7077	41.4007	33.1272	21.627	32.7244	22.1654	17.8829	8.8637	37.1439	62.4482	40.9265	23.1676
57-0.0	15.643	151.984	58.0973	5089	28796	0	82.7097	103.096	74.077	99.1791	72.6846	32.2008	27.3916	17.9229	22.8641	20.1559	12.7325	8.9268	32.4539	49.6902	35.228	18.814
58-1.0	25.424	329.112	138.321	6322	28776	1	39.7234	124.388	96.3337	123.0467	108.5827	46.4067	39.8469	30.9238	32.859	35.5322	19.7099	12.272	59.158	66.7425	48.6444	31.0918
59-1.0	18.032	199.253	81.2653	5541	28792	1	22.1456	100.7458	78.7129	108.0831	91.947	40.3215	34.9936	22.5068	34.7386	26.2169	17.895	8.8249	39.3195	57.6935	40.5826	22.7818
60-0.0	19.952	215.878	88.088	5513	28784	0	36.1254	112.2732	84.2757	93.654	79.0644	35.3985	30.8384	22.5083	28.5119	26.6039	16.0456	10.5639	43.2389	59.6541	41.5281	22.9678
61-1.0	9.516	64.2782	19.2152	3714	28784	1	6.3522	58.2901	48.6513	59.1435	53.9566	27.6993	18.0808	11.0295	15.3485	10.8778	9.1186	4.967	19.0859	31.0473	22.4525	14.4108
62-1.0	23.684	293.364	122.045	6086	28780	1	18.254	119.7238	93.7455	114.0998	101.2348	44.4185	37.0655	29.6309	33.309	32.8276	19.9365	11.0983	54.3219	65.085	45.3559	28.9544
63-1.0	19.827	225.818	91.6079	5655	28788	1	28.3878	111.6696	91.7632	108.2703	98.1832	40.9445	34.1537	24.2808	29.8484	28.9393	16.7084	9.9293	43.8208	59.7709	41.8012	25.2119
64-0.0	15.39	143.528	55.143	5059	28796	0	59.3186	93.1771	68.0421	85.9176	70.1138	33.0477	26.7689	17.7385	24.1163	20.0131	12.5238	7.2363	34.3204	48.8763	34.3712	17.2818
65-1.0	21.872	252.984	103.286	5841	28776	1	26.4793	109.4558	85.1585	109.7267	97.4268	41.6812	35.5964	26.5514	30.0594	31.1536	17.4874	10.6483	49.996	59.756	42.9249	26.8984
66-1.0	20.266	245.288	100.134	5757	28776	1	82.3188	128.2449	110.9452	119.5373	107.2777	43.3477	34.9338	24.5439	32.0108	24.6762	18.4575	10.0692	46.4173	64.4088	43.8137	26.1182
67-0.0	15.39	142.868	53.5766	5064	28796	0	72.3377	97.5005	70.596	79.6507	68.4566	31.572	27.0322	17.0974	21.5462	19.5851	12.6551	9.0932	30.9483	46.4266	34.0156	18.4518
68-0.0	15.203	173.949	69.9459	5085	28796	0	27.3296	116.3681	111.1792	119.02	97.1819	38.743	31.4269	14.4833	28.9273	17.2776	14.3656	8.8153	27.0727	59.513	40.1504	22.7454

Table C. Virtual Random dataset, all subjects. $c_0 = Int \quad c_1 = viewa$.

Dist	El	Az	Int	Int se	Int Tvalue	viewa	viewa se	viewa Tvalue	R se	CV RMSE	CV MSPE	CV MAPE
D35	E0	A0	35.43	0.05213	679.5	2.349	0.0008267	2842	3.382	1.829	11.44	2.541
D35	E0	A30	24.12	0.02743	879.3	2.262	0.0003919	5773	1.666	0.9405	2.777	1.286
D35	E0	A45	24.58	0.03874	634.5	2.274	0.0005579	4075	2.359	1.197	5.568	1.736
D35	E0	A90	5.531	0.05166	107.1	3.414	0.001006	3393	2.833	1.508	8.029	2.103
D35	E0	A150	22.69	0.04851	467.7	2.391	0.0007269	3290	2.922	1.644	8.541	2.241
D35	E0	A-60	24.66	0.05282	466.9	2.444	0.000818	2987	3.217	1.784	10.35	2.47
D35	E0	A-90	3.497	0.07134	49.01	3.459	0.001393	2483	3.869	1.805	14.97	2.701
D35	E0	A-135	21.34	0.03603	592.4	2.246	0.0005032	4464	2.154	1.026	4.641	1.516
D37	E35.5	A30	23.72	0.0356	666.1	2.197	0.0004929	4457	2.158	1.225	4.657	1.656
D37	E35.5	A60	27.11	0.0856	316.7	2.416	0.00133	1817	5.28	2.827	27.88	3.998
D43	E0	A0	21.14	0.03173	666.2	2.235	0.0004404	5075	1.895	1.147	3.592	1.511
D43	E0	A30	20.39	0.02458	829.5	2.253	0.0003425	6577	1.463	0.8062	2.14	1.121
D43	E0	A45	20.76	0.03388	612.6	2.264	0.0004755	4762	2.019	0.9729	4.079	1.453
D43	E0	A60	20.64	0.04672	441.7	2.423	0.0007013	3456	2.782	1.488	7.74	2.094
D43	E0	A90	19.25	0.04662	413	3.481	0.0009974	3490	2.754	1.654	7.588	2.173
D43	E0	A120	18.13	0.04264	425.3	2.4	0.000625	3840	2.504	1.297	6.271	1.846
D43	E0	A135	16.89	0.0304	555.7	2.253	0.0004154	5424	1.773	0.7769	3.146	1.194
D43	E0	A150	17.58	0.02131	824.8	2.22	0.000288	7708	1.248	0.7165	1.558	0.9678
D43	E0	A180	17	0.0326	521.4	2.271	0.0004492	5055	1.903	1.091	3.621	1.483
D43	E30	A0	20.56	0.05329	385.9	2.229	0.0007354	3031	3.171	1.391	10.06	2.13
D43	E30	A30	22.46	0.03084	728.2	2.199	0.0004244	5181	1.856	1.081	3.446	1.444
D43	E30	A45	22.99	0.05851	393	2.229	0.0008187	2723	3.529	1.66	12.46	2.454
D43	E30	A60	25.71	0.07664	335.4	2.388	0.001167	2046	4.691	2.516	22.01	3.545
D43	E30	A90	20.29	0.05114	396.7	3.183	0.001006	3163	3.039	1.713	9.237	2.345
D43	E30	A120	22.81	0.04536	502.7	2.401	0.0006829	3516	2.735	1.455	7.479	2.057
D43	E30	A135	23.18	0.04224	548.8	2.205	0.0005852	3768	2.552	1.363	6.514	1.916
D43	E30	A150	21.36	0.02867	745	2.204	0.000393	5609	1.715	0.8719	2.941	1.267
D43	E30	A180	13.69	0.07349	186.3	2.368	0.001038	2282	4.207	1.944	17.71	2.921
D43	E45	A0	26.41	0.04908	538.2	2.195	0.0006896	3183	3.02	1.687	9.12	2.315
D43	E45	A30	26.71	0.05033	530.8	2.199	0.0007095	3099	3.102	1.698	9.621	2.33
D43	E45	A45	27.36	0.07664	357	2.24	0.001105	2027	4.736	2.282	22.43	3.35
D43	E45	A60	29.34	0.1131	259.5	2.519	0.001856	1357	7.05	3.913	49.72	5.437
D43	E45	A90	26.7	0.06323	422.3	3.046	0.001235	2466	3.895	2.075	15.17	2.933
D43	E45	A120	26.36	0.05378	490.1	2.433	0.0008371	2906	3.307	1.686	10.94	2.436
D43	E45	A135	25.95	0.04921	527.3	2.269	0.0007128	3183	3.02	1.495	9.121	2.208
D43	E45	A150	23.38	0.03704	631.2	2.248	0.0005239	4292	2.241	1.128	5.022	1.644
D43	E45	A180	19.25	0.05158	373.2	2.319	0.0007352	3155	3.047	1.376	9.286	2.114
D43	E60	A0	31.81	0.07209	441.3	2.281	0.001086	2099	4.572	2.512	20.91	3.482
D43	E60	A30	33.97	0.08675	391.6	2.255	0.001309	1722	5.569	2.882	31.02	4.101
D43	E60	A45	35.41	0.1181	299.9	2.348	0.001873	1254	7.627	3.833	58.18	5.608
D43	E60	A60	38.61	0.1623	237.9	2.608	0.002919	893.3	10.64	6.267	113.2	8.418
D43	E60	A90	35.29	0.08756	403	3.207	0.001895	1693	5.665	3.063	32.1	4.312
D43	E60	A120	33.75	0.07945	424.8	2.506	0.001331	1883	5.095	2.876	25.97	3.92
D43	E60	A135	31.42	0.06965	451.1	2.328	0.001069	2178	4.408	2.301	19.44	3.281
D43	E60	A150	31.53	0.07002	450.4	2.237	0.001033	2165	4.435	2.304	19.67	3.29
D43	E60	A180	28.96	0.06336	457.1	2.279	0.000938	2429	3.954	1.848	15.64	2.764
D43	E90	A0	28.26	0.1932	146.3	5.381	0.006745	797.7	11.88	5.525	141.1	8.453
D43	E-30	A0	17.79	0.03305	538.3	2.238	0.000451	4963	1.938	1.006	3.756	1.442
D43	E-30	A30	30.15	0.03935	766.1	2.405	0.0006192	3885	2.475	1.238	6.128	1.798
D43	E-30	A45	20.9	0.03673	569.1	2.368	0.0005393	4390	2.191	1.174	4.801	1.654
D43	E-30	A60	19.31	0.04643	415.8	2.558	0.0007303	3503	2.744	1.399	7.532	2.012
D43	E-30	A90	19.22	0.04604	417.5	3.179	0.0008994	3534	2.72	1.396	7.401	2.009
D43	E-30	A120	16.12	0.06148	262.2	2.509	0.0009319	2693	3.569	1.857	12.74	2.665
D43	E-30	A135	16.13	0.05797	278.3	2.31	0.000809	2856	3.365	1.557	11.33	2.357
D43	E-30	A150	17.69	0.03635	486.5	2.233	0.0004945	4515	2.13	1.105	4.537	1.569
D43	E-30	A180	17.79	0.03305	538.3	2.238	0.000451	4963	1.938	1.006	3.756	1.442
D43	E-45	A0	21.91	0.05339	410.4	2.36	0.000786	3002	3.201	2.04	10.25	2.602
D43	E-45	A30	22.18	0.0383	579	2.343	0.0005606	4179	2.301	1.164	5.295	1.674
D43	E-45	A45	22.56	0.04955	455.3	2.4	0.0007446	3223	2.983	1.443	8.899	2.142
D43	E-45	A60	23.75	0.06253	379.9	2.55	0.001005	2536	3.787	2.115	14.35	2.909
D43	E-45	A90	20.01	0.05765	347.1	3.2	0.001139	2810	3.419	1.839	11.69	2.577
D43	E-45	A120	17.84	0.07139	249.8	2.611	0.001137	2297	4.182	2.218	17.49	3.167
D43	E-45	A135	18.16	0.06933	261.9	2.373	0.001005	2361	4.068	2.03	16.55	2.957
D43	E-45	A150	19.02	0.04715	403.4	2.287	0.000662	3455	2.782	1.468	7.742	2.072
D43	E-45	A180	19.19	0.04449	431.3	2.266	0.0006194	3659	2.628	1.307	6.906	1.918
D43	E-60	A0	22.87	0.07213	317.1	2.522	0.001141	2210	4.346	2.387	18.89	3.309
D43	E-60	A30	23.18	0.05232	443.1	2.503	0.000823	3041	3.16	1.469	9.988	2.218
D43	E-60	A45	23.31	0.04736	492.3	2.524	0.0007518	3358	2.863	1.375	8.197	2.041
D43	E-60	A60	23.87	0.05096	468.4	2.644	0.0008499	3111	3.09	1.8	9.549	2.433
D43	E-60	A90	26.13	0.07524	347.3	3.087	0.001484	2079	4.617	2.858	21.32	3.726
D43	E-60	A120	23.87	0.05096	468.4	2.644	0.0008499	3111	3.09	1.8	9.548	2.433
D43	E-60	A135	21.32	0.07685	277.4	2.529	0.001209	2092	4.588	2.312	21.05	3.387
D43	E-60	A150	21.85	0.05861	372.8	2.435	0.00089	2735	3.513	1.804	12.34	2.593
D43	E-60	A180	21.22	0.05275	402.2	2.41	0.0007901	3050	3.151	1.631	9.928	2.336
D43	E-90	A0	30.42	0.07542	403.4	3.678	0.001818	2023	4.744	2.53	22.51	3.569
D50	E0	A0	29.99	0.04469	671.1	2.316	0.0006764	3424	2.808	1.582	7.884	2.166
D50	E30	A0	37.26	0.05857	636.1	2.246	0.0008976	2502	3.84	1.885	14.75	2.766
D50	E45	A0	33.71	0.04802	701.9	2.225	0.0007136	3117	3.083	1.589	9.509	2.275
D50	E60	A0	43.04	0.08247	521.9	2.363	0.001379	1714	5.594	2.837	31.3	4.103
D50	E90	A0	40.46	0.1756	230.4	5.554	0.006808	815.8	11.62	6.009	135.1	8.604
D50	E-30	A0	30.53	0.05408	564.5	2.394	0.0008488	2820	3.408	2.078	11.61	2.709
D50	E-30	A45	3.898	0.1615	24.13	2.824	0.002583	1093	8.729	4.303	76.22	6.189
D50	E-30	A60	15.52	0.04893	317.1	2.48	0.0007305	3395	2.832	1.426	8.021	2.067
D50	E-30	A90	19.52	0.04103	475.7	3.079	0.0007774	3960	2.428	1.231	5.897	1.785
D50	E-30	A120	11.65	0.06878	169.3	2.448	0.0009925	2466	3.895	2.108	15.17	2.935
D50	E-30	A135	19.33	0.09051	213.6	2.545	0.001417	1796	5.34	2.664	28.53	3.861
D50	E-30	A180	27.24	0.03668	742.7	2.31	0.000545	4239	2.268	1.128	5.147	1.662
D50	E-45	A0	31.23	0.06342	492.4	2.487	0.001039	2395	4.011	2.511	16.09	3.218
D50	E-45	A30	23.33	0.03744	623.2	2.357	0.0005551	4247	2.264	1.171	5.128	1.675
D50	E-45	A45	15.75	0.05235	300.9	2.363	0.0007458	3169	3.033	1.529	9.203	2.201
D50	E-45	A60	4.607	0.1423	32.37	3.001	0.002427	1236	7.731	4.013	59.78	5.551
D50	E-45	A90	24.58	0.1856	132.5	53.62	0.06317	848.8	11.18	6.267	125	8.643
D50	E-45	A120	23.88	0.128	186.5	2.852	0.002305	1237	7.728	4.377	59.74	5.95
D50	E-45	A135	8.639	0.07747	111.5	2.418	0.001087	2225	4.316	2.351	18.63	3.233
D50	E-45	A150	20.11	0.04634	433.9	2.29	0.0006554	3494	2.751	1.456	7.572	2.057
D50	E-45	A180	29.91	0.04462	670.4	2.358	0.0006872	3431	2.802	1.362	7.852	2.033
D50	E-60	A0	33.21	0.06951	477.8	2.682	0.001242	2159	4.446	2.462	19.77	3.401
D50	E-60	A30	30.43	0.05044	603.4	2.591	0.0008563	3026	3.176	1.554	10.09	2.3
D50	E-60	A45	27.91	0.04697	594.2	2.563	0.0007772	3298	2.915	1.43	8.5	2.106
D50	E-60	A60	26.19	0.05109	512.6	2.638	0.0008615	3062	3.139	1.767	9.856	2.435
D50	E-60	A90	27.66	0.07386	374.5	2.999	0.001428	2100	4.572	2.831	20.91	3.693
D50	E-60	A120	23.79	0.07879	301.9	2.716	0.00135	2012	4.77	2.722	22.76	3.746
D50	E-60	A135	26.35	0.07227	364.5	2.549	0.001179	2162	4.441	2.207	19.72	3.261
D50	E-60	A150	30.08	0.05647	532.7	2.5	0.0009231	2708	3.548	1.622	12.59	2.504
D50	E-60	A180	32.83	0.05229	627.9	2.539	0.0008822	2878	3.34	1.65	11.16	2.433
D52	E30	A-30	37.3	0.05034	740.9	2.264	0.0007779	2910	3.303	1.589	10.91	2.332

Table D. Virtual Random dataset, males. $c_0 = Int \quad c_1 = viewa$.

Dist	El	Az	Int	Int se	Int Tvalue	viewa	viewa se	viewa Tvalue	R se	CV RMSE	CV MSPE	CV MAPE
D35	E0	A0	36.248	0.074826	484.44	2.3488	0.0011167	2103.4	3.4013	1.7537	11.576	2.4933
D35	E0	A30	24.509	0.043116	568.45	2.257	0.00057939	3895.5	1.838	1.087	3.3796	1.4492
D35	E0	A45	24.716	0.052321	472.4	2.2723	0.00070867	3206.5	2.2326	1.107	4.9863	1.6186
D35	E0	A90	4.4168	0.079012	55.9	3.4345	0.0014564	2358.2	3.0345	1.5883	9.2108	2.2416
D35	E0	A150	22.337	0.066855	334.11	2.3989	0.00094388	2541.5	2.8159	1.5924	7.9318	2.1649
D35	E0	A-60	24.719	0.06868	359.91	2.4427	0.0010001	2442.5	2.9299	1.583	8.5875	2.2259
D35	E0	A-90	1.772	0.11689	15.16	3.4922	0.0021627	1614.8	4.427	2.0386	19.609	3.1016
D35	E0	A-135	21.214	0.05155	411.53	2.2518	0.00067906	3316	2.1589	0.98533	4.6626	1.4642
D37	E35.5	A30	23.697	0.047036	503.82	2.1949	0.00061201	3586.4	1.9962	1.1984	3.9857	1.5755
D37	E35.5	A60	27.352	0.10902	250.89	2.4066	0.0015869	1516.5	4.7125	2.3838	22.213	3.4585
D43	E0	A0	21.59	0.042785	504.62	2.2322	0.00055982	3987.4	1.7956	1.0521	3.2249	1.411
D43	E0	A30	20.694	0.039158	528.48	2.2473	0.00051336	4377.7	1.6357	0.93828	2.6765	1.2751
D43	E0	A45	20.823	0.045074	461.98	2.2611	0.00059496	3800.4	1.884	0.89237	3.5505	1.3361
D43	E0	A60	20.571	0.059387	346.39	2.422	0.00083859	2888.2	2.4783	1.3344	6.1438	1.8763
D43	E0	A90	18.639	0.068499	272.1	3.4875	0.0013787	2529.6	2.8291	1.7044	8.0068	2.2358
D43	E0	A120	17.592	0.058349	301.5	2.4069	0.00080604	2986.1	2.3972	1.2275	5.7486	1.7602
D43	E0	A135	16.569	0.043915	377.3	2.2597	0.00056648	3989	1.7949	0.74106	3.223	1.1614
D43	E0	A150	17.591	0.02809	626.23	2.2206	0.00035797	6203.3	1.1544	0.68093	1.3331	0.90888
D43	E0	A180	17.102	0.045185	378.5	2.2719	0.00058765	3866.1	1.8519	1.0986	3.4311	1.4627
D43	E30	A0	19.077	0.081001	235.51	2.256	0.0010571	2134.1	3.3523	1.4291	11.242	2.2181
D43	E30	A30	22.522	0.042267	532.84	2.1958	0.00054672	4016.3	1.7827	1.0763	3.1785	1.4113
D43	E30	A45	23.315	0.078357	297.55	2.2222	0.0010302	2157	3.3169	1.4395	11.005	2.1796
D43	E30	A60	25.856	0.095779	269.95	2.3803	0.0013676	1740.5	4.1083	2.1531	16.882	3.0584
D43	E30	A90	19.096	0.068991	276.79	3.1882	0.0012725	2505.5	2.8563	1.6234	8.1602	2.2065
D43	E30	A120	22.188	0.065791	337.25	2.4158	0.00093465	2584.7	2.769	1.3822	7.6693	2.0198
D43	E30	A135	23.364	0.067488	346.2	2.205	0.00088064	2503.8	2.8582	1.5192	8.1719	2.1445
D43	E30	A150	21.395	0.040782	524.61	2.1987	0.00052504	4187.6	1.7098	0.84842	2.9244	1.248
D43	E30	A180	12.561	0.12486	100.6	2.3849	0.0016656	1431.9	4.9897	2.4828	24.909	3.5752
D43	E45	A0	26.239	0.068372	383.77	2.2005	0.00090426	2433.4	2.9408	1.7316	8.6513	2.3011
D43	E45	A30	26.581	0.063432	419.05	2.1958	0.00083868	2618.1	2.7336	1.6202	7.4748	2.1362
D43	E45	A45	27.443	0.091102	301.23	2.2367	0.0012329	1814.1	3.942	1.8835	15.543	2.7559
D43	E45	A60	28.946	0.13671	211.74	2.5131	0.0020967	1198.6	5.9548	3.0531	35.469	4.3881
D43	E45	A90	25.554	0.090496	282.37	3.0614	0.0016592	1845.1	3.876	2.1236	15.026	2.9469
D43	E45	A120	25.208	0.076693	328.69	2.452	0.001124	2181.4	3.2798	1.4671	10.762	2.2809
D43	E45	A135	25.812	0.074038	348.64	2.2736	0.0010094	2252.3	3.1768	1.4851	10.093	2.2657
D43	E45	A150	23.086	0.048549	475.51	2.2451	0.00064404	3486	2.0537	0.99999	4.219	1.4774
D43	E45	A180	18.306	0.079235	231.04	2.3311	0.0010641	2190.6	3.2661	1.4556	10.675	2.2553
D43	E60	A0	32.565	0.092904	350.52	2.2683	0.0013117	1729.3	4.1347	2.3636	17.101	3.2215
D43	E60	A30	33.89	0.10263	330.23	2.2446	0.0014446	1553.8	4.6	2.5371	21.166	3.4906
D43	E60	A45	34.832	0.12742	273.36	2.3417	0.0018816	1244.5	5.7363	2.8412	32.919	4.1584
D43	E60	A60	39.258	0.20434	192.12	2.5804	0.0034136	755.91	9.3927	5.2384	88.245	7.2331
D43	E60	A90	35.4	0.13006	272.19	3.2058	0.0026377	1215.4	5.8731	3.2773	34.508	4.5285
D43	E60	A120	32.182	0.11624	276.84	2.5247	0.0018232	1384.8	5.1586	2.7033	26.621	3.8317
D43	E60	A135	30.071	0.098138	306.41	2.3409	0.0014103	1659.8	4.3071	2.0815	18.559	3.0829
D43	E60	A150	30.805	0.094682	325.35	2.2321	0.0013027	1713.5	4.1727	2.0235	17.418	2.9827
D43	E60	A180	27.876	0.076309	365.3	2.2814	0.0010558	2160.8	3.311	1.3876	10.966	2.1623
D43	E90	A0	25.022	0.19909	125.68	5.5419	0.0065955	840.25	8.4639	3.6128	71.677	5.6517
D43	E-30	A0	18.156	0.040304	450.49	2.2288	0.00051705	4310.5	1.6611	0.89656	2.7602	1.263
D43	E-30	A30	30.992	0.060917	508.75	2.4012	0.00090237	2661	2.6896	1.4022	7.2383	1.9915
D43	E-30	A45	21.095	0.048868	431.67	2.3688	0.00067676	3500.3	2.0454	1.0973	4.1855	1.5493
D43	E-30	A60	19.163	0.055377	346.06	2.5563	0.00081919	3120.5	2.294	1.1704	5.2652	1.6751
D43	E-30	A90	19.356	0.069069	280.24	3.1783	0.0012717	2499.3	2.8634	1.6295	8.2016	2.2215
D43	E-30	A120	15.576	0.068964	225.86	2.5218	0.00098776	2553.1	2.8032	1.4862	7.8601	2.1002
D43	E-30	A135	16.127	0.073277	220.08	2.3144	0.000966	2395.9	2.9867	1.2468	8.9237	1.9535
D43	E-30	A150	17.947	0.045564	393.9	2.2291	0.00058398	3817	1.8757	1.0139	3.5192	1.4176
D43	E-30	A180	18.156	0.040304	450.49	2.2288	0.00051705	4310.5	1.6611	0.89638	2.7606	1.2631
D43	E-45	A0	22.964	0.050724	452.72	2.3764	0.00071177	3338.7	2.1442	1.2046	4.5997	1.6502
D43	E-45	A30	22.874	0.048423	472.37	2.3386	0.00066834	3499.1	2.0461	1.0868	4.188	1.5355
D43	E-45	A45	22.686	0.054852	413.58	2.401	0.0007765	3092	2.3151	1.1176	5.3623	1.6689
D43	E-45	A60	24.064	0.076423	314.88	2.536	0.0011513	2202.7	3.2482	1.7859	10.554	2.481
D43	E-45	A90	20.286	0.084217	240.88	3.1889	0.0015636	2039.5	3.5075	2.0438	12.308	2.7275
D43	E-45	A120	17.477	0.081696	213.93	2.6069	0.0012217	2133.8	3.3528	1.6356	11.244	2.4231
D43	E-45	A135	18.03	0.095728	188.34	2.3738	0.0013074	1815.6	3.9387	1.7757	15.52	2.6883
D43	E-45	A150	19.247	0.067672	284.42	2.2867	0.00089592	2552.3	2.804	1.4593	7.8644	2.0757
D43	E-45	A180	19.625	0.061409	319.57	2.2552	0.00080339	2807.1	2.5498	1.2726	6.504	1.8619
D43	E-60	A0	23.76	0.055993	424.35	2.5497	0.00084662	3011.6	2.3769	1.2928	5.6508	1.8066
D43	E-60	A30	23.893	0.058824	406.18	2.5022	0.00087349	2864.6	2.4987	1.2727	6.2466	1.8463
D43	E-60	A45	23.45	0.051239	457.66	2.5196	0.00076432	3296.5	2.1716	1.1839	4.7183	1.6551
D43	E-60	A60	23.719	0.059116	401.23	2.6359	0.00092387	2853.1	2.5088	1.4298	6.2963	1.9469
D43	E-60	A90	26.104	0.10872	240.1	3.0621	0.002	1531.1	4.6679	2.8882	21.797	3.7479
D43	E-60	A120	23.719	0.059116	401.23	2.6359	0.00092387	2853.1	2.5088	1.4298	6.296	1.9469
D43	E-60	A135	21.247	0.090337	235.19	2.5207	0.0013325	1891.6	3.781	1.6644	14.299	2.56
D43	E-60	A150	22.066	0.086733	254.42	2.4269	0.0012372	1961.7	3.6463	1.8401	13.3	2.6495
D43	E-60	A180	21.551	0.080557	267.53	2.4163	0.0011409	2117.9	3.3779	1.7993	11.413	2.5362
D43	E-90	A0	29.354	0.12222	240.17	3.6885	0.0027571	1337.8	5.3387	2.8441	28.507	4.0123
D50	E0	A0	30.696	0.061978	495.28	2.3156	0.0008839	2619.7	2.732	1.4484	7.4681	2.0494
D50	E30	A0	37.989	0.085024	446.8	2.2383	0.0012213	1832.7	3.9022	1.945	15.233	2.809
D50	E45	A0	33.946	0.067718	501.28	2.2272	0.00094589	2354.6	3.0391	1.51	9.2409	2.2018
D50	E60	A0	44.135	0.1151	383.44	2.3481	0.0017978	1306.1	5.4675	2.8108	29.908	4.0146
D50	E90	A0	37.465	0.19484	192.29	5.7045	0.0071207	801.11	8.8711	3.8795	78.73	6.0241
D50	E-30	A0	31.628	0.066502	475.59	2.4055	0.00099036	2428.9	2.9463	1.5392	8.6857	2.1842
D50	E-30	A45	2.4419	0.25439	9.599	2.8607	0.0038723	738.74	9.6071	4.8401	92.347	6.9199
D50	E-30	A60	15.31	0.06002	255.08	2.4751	0.00084254	2937.7	2.4366	1.274	5.9394	1.7992
D50	E-30	A90	19.478	0.05966	326.49	3.0788	0.0010647	2891.6	2.4754	1.3607	6.1289	1.89
D50	E-30	A120	11.148	0.080816	137.95	2.4668	0.0011068	2228.8	3.2103	1.7739	10.309	2.4439
D50	E-30	A135	16.675	0.12287	135.71	2.5769	0.0018091	1424.4	5.0157	2.2724	25.172	3.428
D50	E-30	A180	27.231	0.048414	562.47	2.3052	0.00067437	3418.3	2.0944	1.0637	4.3887	1.5375
D50	E-45	A0	32.532	0.071429	455.44	2.5068	0.0011141	2249.9	3.1801	1.7054	10.118	2.3928
D50	E-45	A30	24.033	0.050896	472.2	2.3513	0.00071072	3308.4	2.1639	1.1781	4.6843	1.6498
D50	E-45	A45	15.614	0.061508	253.86	2.3651	0.00082634	2862.1	2.5009	1.2952	6.2569	1.8342
D50	E-45	A60	4.6077	0.19971	23.072	3.0058	0.0032271	931.42	7.6454	4.0269	58.48	5.5257
D50	E-45	A90	26.577	0.25001	106.3	54.892	0.082795	662.98	10.682	6.2052	114.13	8.4062
D50	E-45	A120	19.702	0.13282	148.34	2.8877	0.0022267	1296.8	5.5064	3.1541	30.332	4.2374
D50	E-45	A135	8.722	0.10941	79.717	2.4177	0.0014508	1666.4	4.2901	2.19	18.41	3.1059
D50	E-45	A150	20.27	0.067871	298.66	2.2879	0.00090391	2531.1	2.8275	1.4738	7.9972	2.096
D50	E-45	A180	30.193	0.064712	466.57	2.3506	0.00093424	2516	2.8444	1.4099	8.0956	2.084
D50	E-60	A0	33.904	0.072776	465.87	2.7119	0.0012375	2191.4	3.2649	1.6525	10.664	2.3963
D50	E-60	A30	31.192	0.06568	474.91	2.5899	0.0010506	2465.2	2.9029	1.4468	8.4298	2.1107
D50	E-60	A45	28.126	0.056186	500.58	2.5619	0.00087407	2931.1	2.4421	1.2908	5.9657	1.8237
D50	E-60	A60	26.076	0.062101	419.9	2.6344	0.00098241	2681.6	2.6691	1.4626	7.1273	2.0444
D50	E-60	A90	27.667	0.10571	261.72	2.9744	0.001905	1561.3	4.5778	2.7396	20.964	3.6389
D50	E-60	A120	23.705	0.086989	272.5	2.6919	0.0013884	1938.8	3.6892	1.886	13.617	2.72
D50	E-60	A135	26.091	0.086024	303.3	2.5383	0.0013115	1935.5	3.6956	1.5657	13.663	2.4782
D50	E-60	A150	30.297	0.080272	377.44	2.4836	0.0012253	2027	3.5291	1.5883	12.46	2.4759
D50	E-60	A180	32.96	0.075898	434.26	2.5446	0.0012046	2112.4	3.3868	1.6594	11.475	2.4659
D52	E30	A-30	37.639	0.073214	514.09	2.2599	0.0010596	2132.7	3.3545	1.6623	11.258	2.3932

Table E. Virtual Random dataset, females. $c_0 = Int \quad c_1 = viewa$.

Dist	El	Az	Int	Int se	Int Tvalue	viewa	viewa se	viewa Tvalue	R se	CV RMSE	CV MSPE	CV MAPE
D35	E0	A0	35.024	0.069375	504.86	2.3417	0.0011796	1985.1	3.161	1.6766	9.9957	2.3527
D35	E0	A30	23.696	0.034175	693.37	2.2687	0.00052419	4328	1.4513	0.80591	2.107	1.1103
D35	E0	A45	24.461	0.058148	420.66	2.2751	0.00089868	2531.6	2.4799	1.2838	6.1515	1.8481
D35	E0	A90	6.7174	0.065975	101.82	3.3889	0.0013681	2477	2.5344	1.4161	6.4256	1.943
D35	E0	A150	23.194	0.070779	327.69	2.3795	0.0011354	2095.8	2.9944	1.6758	8.9676	2.295
D35	E0	A-60	24.603	0.081664	301.27	2.4446	0.0013576	1800.7	3.4837	1.9926	12.14	2.7153
D35	E0	A-90	5.3352	0.080041	66.656	3.42	0.0016622	2057.6	3.05	1.6052	9.3041	2.2772
D35	E0	A-135	21.67	0.05004	433.04	2.237	0.00074757	2992.4	2.0985	1.1143	4.4044	1.5698
D37	E35.5	A30	23.626	0.05414	436.39	2.2006	0.00080521	2733	2.2974	1.2726	5.2792	1.7438
D37	E35.5	A60	26.564	0.13357	198.88	2.4324	0.0022373	1087.2	5.7549	3.2791	33.128	4.4822
D43	E0	A0	20.812	0.046859	444.15	2.2362	0.00069617	3212.2	1.955	1.1826	3.8235	1.5575
D43	E0	A30	19.991	0.029627	674.73	2.2604	0.00044269	5105.9	1.2303	0.68683	1.5141	0.9486
D43	E0	A45	20.552	0.051087	402.29	2.2702	0.00076933	2950.9	2.1279	1.0303	4.5296	1.5445
D43	E0	A60	20.588	0.073216	281.2	2.427	0.0011791	2058.4	3.0488	1.6565	9.299	2.3139
D43	E0	A90	19.682	0.063561	309.66	3.4785	0.001459	2384.1	2.633	1.5097	6.9351	2.0361
D43	E0	A120	18.681	0.062853	297.22	2.3918	0.00098611	2425.5	2.5882	1.3741	6.7	1.9303
D43	E0	A135	17.385	0.041543	418.48	2.2424	0.00060631	3698.4	1.6982	0.85083	2.8847	1.2338
D43	E0	A150	17.612	0.03254	541.25	2.2183	0.00047043	4715.4	1.3321	0.74915	1.7752	1.0228
D43	E0	A180	17.02	0.047396	359.11	2.2675	0.00069798	3248.6	1.9331	1.081	3.7383	1.4911
D43	E30	A0	22.454	0.061127	367.33	2.1916	0.00089896	2437.9	2.575	1.4062	6.633	1.9629
D43	E30	A30	22.248	0.045138	492.88	2.2055	0.00066715	3305.8	1.8997	1.0811	3.6095	1.4627
D43	E30	A45	22.502	0.088054	255.55	2.2395	0.0013239	1691.6	3.7077	1.8581	13.75	2.6856
D43	E30	A60	25.241	0.12087	208.82	2.4033	0.0019839	1211.4	5.1688	2.8847	26.721	3.985
D43	E30	A90	20.84	0.069894	298.17	3.1922	0.0014827	2153	2.9151	1.4225	8.4998	2.0865
D43	E30	A120	23.736	0.060013	395.52	2.3781	0.00096521	2463.8	2.548	1.4709	6.4946	1.9944
D43	E30	A135	23.116	0.051584	448.12	2.2028	0.00076556	2877.4	2.1822	1.1976	4.7633	1.6658
D43	E30	A150	21.011	0.03815	550.75	2.2161	0.00056233	3940.9	1.5938	0.84786	2.5412	1.2037
D43	E30	A180	14.957	0.078014	191.72	2.3473	0.0011751	1997.6	3.1414	1.4312	9.8729	2.2254
D43	E45	A0	26.753	0.070966	376.98	2.186	0.0010689	2045	3.0686	1.6163	9.4196	2.303
D43	E45	A30	26.595	0.078533	338.65	2.2065	0.0011929	1849.7	3.3918	1.8345	11.506	2.5387
D43	E45	A45	27.151	0.12515	216.94	2.2462	0.0019428	1156.2	5.4141	2.7513	29.319	3.9439
D43	E45	A60	29.183	0.18144	160.84	2.5371	0.0032247	786.77	7.9223	4.8668	62.774	6.393
D43	E45	A90	27.678	0.088493	312.77	3.0327	0.0018601	1630.3	3.8465	1.9223	14.803	2.8306
D43	E45	A120	27.645	0.074108	373.04	2.408	0.0012365	1947.4	3.2221	1.8884	10.385	2.5334
D43	E45	A135	26.233	0.065634	399.68	2.2609	0.0010192	2218.4	2.8294	1.4773	8.0079	2.1257
D43	E45	A150	23.301	0.053892	432.37	2.2584	0.00082093	2751	2.2823	1.2211	5.2099	1.7064
D43	E45	A180	20.178	0.066288	304.41	2.3064	0.0010119	2279.2	2.754	1.3293	7.586	1.9668
D43	E60	A0	30.892	0.11137	277.38	2.299	0.0018113	1269.3	4.9348	2.5976	24.358	3.6861
D43	E60	A30	33.5	0.13968	239.83	2.2764	0.0022881	994.91	6.2836	3.3487	39.491	4.6973
D43	E60	A45	35.285	0.19917	177.16	2.3696	0.0034393	688.99	9.025	5.1176	81.475	7.006
D43	E60	A60	37.139	0.25482	145.75	2.6586	0.0050042	531.27	11.623	7.2675	135.14	9.4627
D43	E60	A90	35.209	0.11963	294.31	3.2077	0.0027912	1149.2	5.4466	2.8613	29.68	4.0978
D43	E60	A120	35.104	0.10837	323.92	2.4885	0.00196	1269.6	4.9334	2.9066	24.347	3.894
D43	E60	A135	32.511	0.098615	329.68	2.3176	0.0016334	1418.9	4.4172	2.4598	19.52	3.3973
D43	E60	A150	31.54	0.099375	317.39	2.2548	0.0015915	1416.8	4.4238	2.4145	19.575	3.3505
D43	E60	A180	29.467	0.098328	299.69	2.2858	0.0015755	1450.8	4.3205	2.2424	18.671	3.1824
D43	E90	A0	32.281	0.32022	100.81	5.1667	0.011861	435.61	14.059	7.7939	197.71	10.811
D43	E-30	A0	17.091	0.050519	338.31	2.2548	0.00074014	3046.5	2.0612	1.1101	4.2498	1.5569
D43	E-30	A30	29.615	0.046892	631.55	2.4039	0.00079066	3040.4	2.0654	0.99745	4.2687	1.4633
D43	E-30	A45	20.899	0.054879	380.81	2.3627	0.00086191	2741.2	2.2905	1.1861	5.2479	1.7037
D43	E-30	A60	19.251	0.075223	255.91	2.5644	0.0012697	2019.6	3.1072	1.6625	9.6568	2.3413
D43	E-30	A90	19.163	0.062037	308.89	3.1773	0.0012967	2450.3	2.562	1.2085	6.5674	1.8068
D43	E-30	A120	16.939	0.10171	166.54	2.4897	0.0016443	1514.1	4.1405	2.3363	17.149	3.2219
D43	E-30	A135	16.359	0.090636	180.49	2.3021	0.0013501	1705.1	3.6784	1.9543	13.533	2.7578
D43	E-30	A150	17.392	0.057577	302.07	2.2371	0.00083844	2668.2	2.3531	1.1838	5.5381	1.7069
D43	E-30	A180	17.091	0.050519	338.31	2.2548	0.00074014	3046.5	2.0612	1.11	4.2495	1.5568
D43	E-45	A0	22.035	0.054665	403.09	2.3204	0.00084901	2733.1	2.2973	1.3127	5.2795	1.7788
D43	E-45	A30	21.699	0.05857	370.48	2.3436	0.00091688	2556.1	2.4562	1.0582	6.0348	1.6563
D43	E-45	A45	22.588	0.083466	270.63	2.3954	0.0013429	1783.7	3.5169	1.8211	12.372	2.6017
D43	E-45	A60	22.918	0.097648	234.69	2.577	0.0016938	1521.4	4.1207	2.3227	16.989	3.1813
D43	E-45	A90	19.442	0.079074	245.88	3.2203	0.0016781	1919	3.2697	1.5635	10.695	2.3432
D43	E-45	A120	17.73	0.11721	151.28	2.6242	0.0020068	1307.6	4.7909	2.8825	22.958	3.8332
D43	E-45	A135	18.256	0.10224	178.56	2.3723	0.0015872	1494.6	4.1944	2.3054	17.597	3.2317
D43	E-45	A150	18.904	0.066682	283.5	2.2865	0.0010015	2283.1	2.7493	1.4655	7.5607	2.0536
D43	E-45	A180	18.386	0.062907	292.27	2.2852	0.0009413	2427.7	2.5859	1.306	6.6887	1.8936
D43	E-60	A0	23.324	0.095955	243.07	2.4664	0.001597	1544.5	4.0596	2.1158	16.486	2.999
D43	E-60	A30	22.853	0.085073	268.62	2.4971	0.0014292	1747.2	3.5902	1.4874	12.892	2.3802
D43	E-60	A45	23.03	0.080608	285.7	2.5329	0.001375	1842	3.4059	1.5649	11.608	2.3914
D43	E-60	A60	23.538	0.081228	289.78	2.6625	0.001461	1822.3	3.4426	1.9835	11.856	2.6865
D43	E-60	A90	25.041	0.094144	265.99	3.142	0.0020171	1557.7	4.0252	2.3321	16.211	3.1638
D43	E-60	A120	23.538	0.081228	289.78	2.6625	0.001461	1822.3	3.4426	1.9834	11.856	2.6864
D43	E-60	A135	20.892	0.12452	167.78	2.5483	0.0021102	1207.6	5.1851	3.0187	26.891	4.1224
D43	E-60	A150	21.371	0.079426	269.06	2.4484	0.0012965	1888.4	3.3225	1.7694	11.041	2.5008
D43	E-60	A180	21.361	0.063994	333.79	2.3949	0.0010216	2344.1	2.6778	1.3691	7.1726	1.9622
D43	E-90	A0	31.122	0.088849	350.28	3.6763	0.0023136	1589	3.9463	2.2152	15.581	3.051
D50	E0	A0	29.663	0.061531	482.09	2.3096	0.00099715	2316.2	2.7101	1.4975	7.3473	2.0728
D50	E30	A0	36.622	0.081338	450.25	2.252	0.001344	1675.5	3.7432	1.747	14.02	2.6556
D50	E45	A0	33.712	0.067861	496.77	2.2172	0.0010833	2046.6	3.0662	1.5787	9.4072	2.2605
D50	E60	A0	41.944	0.1192	351.87	2.3809	0.002158	1103.3	5.6716	2.719	32.178	4.0737
D50	E90	A0	43.9	0.28449	154.31	5.3591	0.011799	454.21	13.511	8.1464	182.59	10.781
D50	E-30	A0	30.382	0.060546	501.81	2.3627	0.0010083	2343.2	2.6789	1.3811	7.1803	1.9839
D50	E-30	A45	6.078	0.19711	30.836	2.7691	0.003331	831.31	7.504	3.6926	56.321	5.2997
D50	E-30	A60	15.322	0.076425	200.49	2.4918	0.0012246	2034.7	3.0841	1.5557	9.5141	2.2659
D50	E-30	A90	19.538	0.057485	339.88	3.0787	0.0011668	2638.5	2.3796	1.1146	5.6655	1.6751
D50	E-30	A120	12.782	0.1063	120.25	2.4156	0.0016272	1484.5	4.2227	2.3412	17.835	3.2415
D50	E-30	A135	21.688	0.13012	166.68	2.5135	0.0021857	1150	5.4429	3.173	29.632	4.2287
D50	E-30	A180	26.986	0.054496	495.19	2.3209	0.0008727	2659.5	2.3608	1.2351	5.5748	1.7686
D50	E-45	A0	31.111	0.065552	474.6	2.4421	0.0011336	2154.2	2.9134	1.589	8.4905	2.2181
D50	E-45	A30	22.765	0.054729	415.96	2.3619	0.00086905	2717.8	2.3102	1.0318	5.3391	1.5962
D50	E-45	A45	15.905	0.086187	184.54	2.3606	0.0013129	1798	3.489	1.8133	12.177	2.573
D50	E-45	A60	4.8273	0.2066	23.365	2.9921	0.0037464	798.64	7.8064	4.0411	60.971	5.5785
D50	E-45	A90	24.26	0.19272	125.88	51.601	0.067566	763.71	8.1575	4.4633	66.565	6.238
D50	E-45	A120	26.481	0.20692	127.98	2.8436	0.0040543	701.38	8.8687	5.747	78.671	7.2518
D50	E-45	A135	8.5682	0.11198	76.517	2.4191	0.0016755	1443.8	4.3412	2.5103	18.849	3.3621
D50	E-45	A150	19.933	0.064376	309.64	2.293	0.00097557	2350.4	2.6707	1.4391	7.1348	2.0116
D50	E-45	A180	29.459	0.061876	476.09	2.3693	0.0010274	2306.2	2.7218	1.3266	7.4121	1.9751
D50	E-60	A0	33.668	0.087796	383.48	2.6229	0.0016578	1582.1	3.9633	2.0727	15.713	2.9775
D50	E-60	A30	30.046	0.074397	403.87	2.5848	0.0013527	1910.8	3.2836	1.4442	10.787	2.2743
D50	E-60	A45	27.784	0.076243	364.41	2.5621	0.0013547	1891.2	3.3175	1.602	11.009	2.3915
D50	E-60	A60	26.074	0.081735	319.01	2.6462	0.0014842	1782.9	3.5184	2.0221	12.384	2.7399
D50	E-60	A90	26.566	0.093347	284.59	3.0532	0.0019619	1556.2	4.029	2.3631	16.238	3.1928
D50	E-60	A120	22.525	0.12081	186.45	2.7714	0.0022487	1232.5	5.0812	3.2069	25.823	4.1589
D50	E-60	A135	25.907	0.11377	227.72	2.5741	0.002008	1281.9	4.8864	2.8694	23.881	3.879
D50	E-60	A150	29.229	0.07502	389.62	2.5308	0.0013287	1904.7	3.294	1.6171	10.853	2.3916
D50	E-60	A180	32.995	0.07131	462.7	2.5261	0.0012911	1956.6	3.207	1.5191	10.288	2.2889
D52	E30	A-30	36.984	0.070259	526.4	2.2676	0.0011717	1935.3	3.2421	1.4935	10.515	2.2566

Table F. Virtual Random dataset, all stature linear model.
$c_0 = Int \quad c_1 = viewa \quad c_2 = stat.$

Dist	El	Az	Int	Int se	Int t-value	viewa	viewa se	viewa t-value	Stature	Stature se	Stature t-value	R se	CV RMSE	CV MSPE	CV MAPE
D35	E0	A0	17.689	0.1521	116.3	2.0601	0.0024749	832.4	2.0574	0.017031	120.8	2.5712	1.5761	6.6131	2.066
D35	E0	A30	20.978	0.097456	215.26	2.2082	0.0016586	1331.4	0.39725	0.011864	33.484	1.6215	0.97975	2.6299	1.287
D35	E0	A45	28.026	0.14719	190.4	2.3335	0.002529	922.72	-0.43586	0.017993	-24.224	2.3256	1.1197	5.4108	1.6792
D35	E0	A90	5.911	0.15447	38.266	3.4254	0.0045479	753.18	-0.056266	0.021545	-2.6115	2.8329	1.512	8.0269	2.1086
D35	E0	A150	31.796	0.17821	178.42	2.5608	0.0032815	780.37	-1.173	0.022193	-52.852	2.7374	1.4074	7.4962	2.0247
D35	E0	A-60	31.094	0.20403	152.4	2.5639	0.0037825	677.84	-0.81505	0.025028	-32.565	3.1353	1.659	9.8327	2.3625
D35	E0	A-90	-0.14065	0.20197	-0.69639	3.3452	0.0061007	548.32	0.54787	0.028499	19.224	3.8338	1.8139	14.703	2.6905
D35	E0	A-135	29.353	0.12396	236.8	2.3878	0.0021589	1106	-1.0414	0.015548	-66.979	1.9469	0.87013	3.792	1.3474
D37	E35.5	A30	24.238	0.13297	182.28	2.2055	0.0022134	996.45	-0.066279	0.016301	-4.0659	2.1571	1.2166	4.6548	1.6527
D37	E35.5	A60	33.94	0.35828	94.73	2.5409	0.0065102	390.3	-0.85286	0.043487	-19.612	5.2296	2.7496	27.359	3.9291
D43	E0	A0	12.701	0.087547	145.07	2.0891	0.0014924	1399.8	1.0894	0.010803	100.84	1.543	0.93259	2.3815	1.2254
D43	E0	A30	15.986	0.078339	204.06	2.175	0.0013584	1601.2	0.57358	0.0097583	58.779	1.3506	0.82289	1.8246	1.0795
D43	E0	A45	22.734	0.12225	185.96	2.2995	0.0021424	1073.3	-0.25773	0.015309	-16.835	2.0054	0.94693	4.0228	1.4348
D43	E0	A60	25.881	0.16988	152.35	2.5237	0.0032045	787.55	-0.68521	0.021386	-32.04	2.7132	1.4177	7.3629	2.0295
D43	E0	A90	8.9569	0.13252	67.59	3.1994	0.0035597	898.8	1.349	0.016538	81.569	2.386	1.356	5.6943	1.8603
D43	E0	A120	25.695	0.14585	176.18	2.5462	0.0027767	916.99	-1.0087	0.018715	-53.899	2.3398	1.1411	5.4767	1.6922
D43	E0	A135	22.918	0.098444	232.8	2.3631	0.0017696	1335.4	-0.81055	0.01271	-63.771	1.6167	0.75629	2.6148	1.1368
D43	E0	A150	14.917	0.069063	215.99	2.1726	0.0012049	1803.2	0.35429	0.008784	40.334	1.2003	0.67847	1.4409	0.92082
D43	E0	A180	12.056	0.10117	119.16	2.1807	0.0018092	1205.3	0.66058	0.012891	51.242	1.7889	0.95343	3.2008	1.3521
D43	E30	A0	17.329	0.18664	92.85	2.1723	0.0032157	675.54	0.42153	0.023327	18.07	3.1455	1.3575	9.8996	2.0947
D43	E30	A30	22.571	0.11292	199.88	2.201	0.0018959	1161	-0.01428	0.013949	-1.0237	1.8562	1.0796	3.4463	1.4431
D43	E30	A45	31.518	0.22244	141.69	2.3769	0.0038153	622.99	-1.0962	0.027669	-39.621	3.3983	1.4693	11.553	2.3061
D43	E30	A60	33.155	0.31136	106.48	2.5243	0.0056351	447.96	-0.93941	0.038101	-24.656	4.6215	2.4107	21.364	3.452
D43	E30	A90	9.433	0.14945	63.117	2.9135	0.0036464	799.02	1.4111	0.018525	76.174	2.6754	1.3774	7.1607	1.9959
D43	E30	A120	29.296	0.16841	173.96	2.5218	0.003103	812.68	-0.83413	0.020905	-39.902	2.6318	1.3812	6.9306	1.974
D43	E30	A135	28.303	0.15762	179.57	2.2922	0.0026562	862.99	-0.65597	0.019486	-33.664	2.4825	1.2676	6.1667	1.8339
D43	E30	A150	21.126	0.10321	204.68	2.2004	0.0017489	1258.2	0.030092	0.012838	2.344	1.7147	0.87366	2.9412	1.2682
D43	E30	A180	9.347	0.23671	39.487	2.2827	0.0045545	501.19	0.59942	0.031069	19.293	4.1688	1.9884	17.385	2.9011
D43	E45	A0	23.239	0.18483	125.73	2.1427	0.0030152	710.65	0.39536	0.022213	17.799	2.9962	1.7148	8.9787	2.3208
D43	E45	A30	27.418	0.1969	139.25	2.2104	0.0032234	685.73	-0.087952	0.023705	-3.7102	3.1006	1.6844	9.6169	2.3244
D43	E45	A45	33.274	0.31682	105.03	2.3401	0.0053128	440.46	-0.73649	0.038296	-19.231	4.6929	2.1858	22.027	3.2864
D43	E45	A60	31.149	0.49688	62.688	2.5532	0.0093306	273.64	-0.22264	0.059632	-3.7336	7.0482	3.9094	49.689	5.4336
D43	E45	A90	15.842	0.21575	73.426	2.7996	0.0048495	577.3	1.3469	0.025727	52.353	3.6525	1.9604	13.344	2.7599
D43	E45	A120	31.416	0.21279	147.64	2.5257	0.0038794	651.07	-0.63299	0.025782	-24.551	3.2585	1.6812	10.623	2.4146
D43	E45	A135	31.799	0.19234	165.33	2.3695	0.0032787	722.69	-0.73398	0.023368	-31.41	2.9481	1.4114	8.6944	2.1269
D43	E45	A150	28.297	0.13727	206.15	2.3335	0.0023534	991.54	-0.62802	0.016934	-37.086	2.1675	1.0224	4.7002	1.5527
D43	E45	A180	22.72	0.18559	122.42	2.3837	0.0033802	705.19	-0.45873	0.023566	-19.466	3.0184	1.3523	9.1142	2.0919
D43	E60	A0	26.11	0.29062	89.844	2.187	0.004772	458.29	0.68392	0.033785	20.243	4.5265	2.5883	20.493	3.487
D43	E60	A30	26.64	0.36177	73.638	2.1363	0.0058086	367.79	0.86733	0.041564	20.867	5.5095	2.9738	30.364	4.0993
D43	E60	A45	22.957	0.50799	45.192	2.1397	0.0084854	252.16	1.4635	0.058122	25.18	7.5093	3.9028	56.401	5.5534
D43	E60	A60	10.675	0.7196	14.834	2.0928	0.013253	157.91	3.231	0.081247	39.768	10.242	5.7853	104.91	7.9484
D43	E60	A90	9.338	0.26153	35.706	2.6294	0.0058087	452.66	3.0118	0.029216	103.09	4.5778	2.6113	20.961	3.5415
D43	E60	A120	29.741	0.33729	88.176	2.4341	0.006031	403.59	0.47463	0.038846	12.218	5.0765	2.8522	25.781	3.8908
D43	E60	A135	36.268	0.29968	121.02	2.4103	0.0050716	475.26	-0.58531	0.035191	-16.633	4.3782	2.2672	19.179	3.2515
D43	E60	A150	34.401	0.29945	114.88	2.2833	0.0048592	469.89	-0.34539	0.035093	-9.8421	4.4242	2.2617	19.584	3.2667
D43	E60	A180	31.821	0.2604	122.2	2.3269	0.0043712	532.32	-0.35077	0.031	-11.315	3.9416	1.797	15.543	2.741
D43	E90	A0	-28.13	0.2966	-94.841	3.2305	0.011185	288.82	6.7805	0.033129	204.67	6.7508	2.8329	45.592	4.6987
D43	E-30	A0	17.467	0.1139	153.36	2.2326	0.0020086	1111.5	0.043228	0.014519	2.9774	1.9375	1.0082	3.7548	1.4426
D43	E-30	A30	19.604	0.12596	155.63	2.2216	0.002179	1019.5	1.2729	0.014654	86.861	2.1091	1.2201	4.4499	1.6559
D43	E-30	A45	19.896	0.13097	151.91	2.3489	0.002391	982.38	0.13081	0.016339	8.006	2.1874	1.1751	4.786	1.6523
D43	E-30	A60	21.538	0.16611	129.66	2.6038	0.0033285	782.25	-0.29427	0.021043	-13.984	2.731	1.4116	7.461	2.0186
D43	E-30	A90	5.4062	0.1047	51.636	2.835	0.002554	1110	1.8061	0.012996	138.98	1.9401	0.91769	3.7651	1.3871
D43	E-30	A120	23.729	0.21483	110.45	2.666	0.0043522	612.56	-1.0335	0.028039	-36.861	3.4532	1.81	11.927	2.5903
D43	E-30	A135	24.063	0.20098	119.73	2.4609	0.0037473	656.71	-1.0768	0.026222	-41.064	3.2317	1.5461	10.446	2.2973
D43	E-30	A150	18.343	0.12604	145.53	2.2444	0.0022219	1010.1	-0.08766	0.016101	-5.4445	2.1284	1.0981	4.5313	1.5657
D43	E-30	A180	17.467	0.1139	153.36	2.2326	0.0020086	1111.5	0.043228	0.014519	2.9774	1.9375	1.0082	3.7553	1.4427
D43	E-45	A0	16.708	0.18519	90.223	2.2644	0.0033402	677.92	0.67034	0.022888	29.288	3.1349	1.9618	9.8303	2.5408
D43	E-45	A30	20.896	0.1385	150.87	2.3195	0.0024806	935.06	0.16474	0.017129	9.6177	2.2956	1.16	5.2722	1.6656
D43	E-45	A45	22.775	0.18339	124.18	2.4035	0.0033663	713.98	-0.026977	0.022686	-1.1891	2.9827	1.4444	8.9002	2.1436
D43	E-45	A60	18.851	0.22587	83.458	2.4546	0.0043602	562.94	0.62342	0.027631	22.563	3.7403	2.0311	13.993	2.8313
D43	E-45	A90	1.6365	0.11623	14.079	2.7453	0.002825	971.81	2.381	0.01427	166.86	2.2106	1.1731	4.8877	1.6655
D43	E-45	A120	24.336	0.25919	93.894	2.7485	0.0054041	508.61	-0.8711	0.033441	-26.049	4.1124	2.1729	16.917	3.106
D43	E-45	A135	23.414	0.25153	93.088	2.4737	0.0047477	521.02	-0.70203	0.032329	-21.715	4.0212	2.0347	16.173	2.9456
D43	E-45	A150	21.485	0.16826	127.69	2.3323	0.0030216	771.89	-0.32553	0.021364	-15.237	2.7665	1.44	7.6552	2.0539
D43	E-45	A180	21.256	0.15863	134	2.3037	0.0028169	817.79	-0.27293	0.020104	-13.576	2.6158	1.2866	6.8443	1.9021
D43	E-60	A0	18.881	0.26303	71.783	2.4441	0.0050668	482.37	0.51198	0.032453	15.776	4.3188	2.284	18.656	3.2487
D43	E-60	A30	24.053	0.19626	122.56	2.52	0.0037465	672.62	-0.11128	0.0242	-4.5981	3.1586	1.4748	9.9799	2.2225
D43	E-60	A45	23.824	0.17703	134.58	2.5344	0.0034012	745.15	-0.06511	0.021788	-2.9883	2.8623	1.3748	8.1951	2.0406
D43	E-60	A60	20.002	0.1844	108.47	2.5655	0.0036825	696.68	0.49117	0.022523	21.807	3.0538	1.7768	9.3285	2.3954
D43	E-60	A90	1.7986	0.16512	10.893	2.5335	0.0037104	682.81	3.0056	0.019412	154.83	3.1134	1.8645	9.6945	2.4589
D43	E-60	A120	20.002	0.1844	108.47	2.5655	0.0036825	696.68	0.49117	0.022523	21.807	3.0538	1.7768	9.3279	2.3954
D43	E-60	A135	23.849	0.28979	82.298	2.5795	0.0057002	452.53	-0.32945	0.036399	-9.0511	4.579	2.2949	20.972	3.3794
D43	E-60	A150	24.15	0.21863	110.46	2.4784	0.0041066	603.51	-0.29749	0.027266	-10.911	3.5023	1.7767	12.271	2.5737
D43	E-60	A180	26.584	0.19502	136.31	2.5121	0.0036484	688.54	-0.69871	0.024475	-28.548	3.0885	1.564	9.5436	2.2819
D43	E-90	A0	7.1292	0.19911	35.805	3.064	0.0052124	587.82	2.7943	0.022884	122.11	3.5908	1.9928	12.896	2.747
D50	E0	A0	15.269	0.12148	125.7	2.0699	0.0020173	1026.1	1.7754	0.014086	126.04	2.096	1.2967	4.3941	1.6889
D50	E30	A0	22.796	0.2126	107.22	2.0218	0.0032869	615.11	1.6604	0.023656	70.191	3.4397	1.9408	11.835	2.6473
D50	E45	A0	20.025	0.15601	128.36	2.0101	0.0024376	824.63	1.6086	0.017718	90.786	2.5948	1.5283	6.7352	2.0428
D50	E60	A0	22.975	0.32394	70.924	2.0484	0.0051026	401.44	2.218	0.03483	63.681	5.1008	2.9849	26.023	3.9854
D50	E90	A0	-21.08	0.26895	-78.379	3.3415	0.0097539	342.58	6.787	0.02801	242.31	5.8576	2.5282	34.323	4.0675
D50	E-30	A0	15.73	0.1671	94.132	2.1386	0.0028635	746.83	1.7795	0.019338	92.016	2.8561	1.8198	8.1588	2.3313
D50	E-30	A45	-9.0577	0.46742	-19.378	2.485	0.011775	211.03	1.9717	0.066944	29.453	8.5462	4.1308	73.069	6.0626
D50	E-30	A60	18.745	0.16742	111.96	2.5453	0.0033435	761.27	-0.43919	0.021807	-20.14	2.8038	1.4649	7.8629	2.0801
D50	E-30	A90	8.1689	0.10369	78.779	2.8052	0.002453	1143.6	1.482	0.012889	114.98	1.8841	0.92982	3.5508	1.3696
D50	E-30	A120	22.308	0.22745	98.081	2.6713	0.0046653	572.59	-1.5067	0.0308	-48.919	3.6809	2.0164	13.552	2.817
D50	E-30	A135	29.076	0.34713	83.76	2.7451	0.0070359	390.15	-1.2955	0.044615	-29.038	5.2314	2.5474	27.376	3.7669
D50	E-30	A180	20.218	0.12588	160.61	2.1899	0.0021375	1024.5	0.86727	0.014968	57.943	2.0991	1.1261	4.408	1.5905
D50	E-45	A0	17.523	0.2176	80.529	2.242	0.0038708	579.2	1.6424	0.025148	65.313	3.6412	2.2845	13.261	2.9604
D50	E-45	A30	19.765	0.13292	148.69	2.2929	0.0023712	967	0.4546	0.016272	27.937	2.2214	1.1425	4.9362	1.6319
D50	E-45	A45	17.824	0.17973	99.169	2.4032	0.0034128	704.19	-0.28137	0.023353	-12.048	3.0226	1.5722	9.1384	2.2274
D50	E-45	A60	1.4489	0.44745	3.2381	2.914	0.01199	243.04	0.47824	0.064242	7.4443	7.7208	3.9605	59.626	5.5349
D50	E-45	A90	-8.1337	0.63382	-12.833	39.899	0.26236	152.08	4.1928	0.078126	53.667	10.454	5.9021	109.31	8.0128
D50	E-45	A120	18.222	0.50711	35.933	2.7254	0.01118	243.79	0.7267	0.063049	11.526	7.703	4.3139	59.351	5.922
D50	E-45	A135	14.601	0.25091	58.192	2.545	0.005185	490.84	-0.86388	0.034633	-24.944	4.25	2.2906	18.066	3.2421
D50	E-45	A150	20.25	0.16592	122.04	2.2926	0.0029535	776.23	-0.018178	0.020859	-0.8715	2.7515	1.4549	7.573	2.0568
D50	E-45	A180	22.621	0.16305	138.74	2.2326	0.0027806	802.94	0.88318	0.019073	46.306	2.6628	1.3874	7.0937	1.9928
D50	E-60	A0	21.391	0.26121	81.894	2.4558	0.0049669	494.44	1.3994	0.029918	46.773	4.2215	2.2727	17.825	3.2017
D50	E-60	A30	25.339	0.19547	129.63	2.4952	0.0036627	681.23	0.6155	0.022856	26.93	3.1203	1.5436	9.7407	2.2672
D50	E-60	A45	24.17	0.17867	135.28	2.4918	0.0033674	739.99	0.46046	0.021248	21.671	2.8816	1.4201	8.3062	2.09
D50	E-60	A60	21.413	0.18822	113.77	2.5432	0.0036907	689.08	0.59552	0.022624	26.323	3.0862	1.7506	9.5269	2.3999
D50	E-60	A90	3.4754	0.16775	20.718	2.4703	0.00363	680.51	2.9556	0.019549	151.19	3.1235	1.8533	9.7567	2.4579
D50	E-60	A120	23.274	0.30167	77.151	2.705	0.0062523	432.64	0.065523	0.037174	1.7626	4.7696	2.7254	22.756	3.7472
D50	E-60	A135	23.801	0.27964	85.111	2.4996	0.0053286	469.09	0.31811	0.033771	9.4197	4.431	2.2351	19.641	3.2681
D50	E-60	A150	22.831	0.21287	107.25	2.3675	0.0038529	614.47	0.87823	0.024916	35.247	3.443	1.6675	11.858	2.4874
D50	E-60	A180	24.204	0.19798	122.26	2.3826	0.00357	667.4	1.0235	0.022734	45.02	3.1822	1.646	10.131	2.3638
D52	E30	A-30	23.63	0.17799	132.76	2.0508	0.002771	740.11	1.5681	0.019791	79.237	2.8812	1.7165	8.3035	2.2682

Table G. Virtual Random dataset, males stature. $c_0 = Int \quad c_1 = viewa \quad c_2 = stat$.

Dist	El	Az	Int	Int se	Int t-value	viewa	viewa se	viewa t-value	Stature	Stature se	Stature t-value	R se	CV RMSE	CV MSPE	CV MAPE
D35	E0	A0	17.616	0.21615	81.5	2.0516	0.0034331	597.59	2.1543	0.024143	89.232	2.5406	1.5582	6.4597	2.0351
D35	E0	A30	20.041	0.15112	132.61	2.1819	0.0025071	870.28	0.56433	0.018362	30.733	1.7573	1.107	3.0908	1.4212
D35	E0	A45	27.962	0.20013	139.72	2.3276	0.0033641	691.9	-0.41078	0.024469	-16.788	2.202	1.0525	4.8545	1.5732
D35	E0	A90	6.7172	0.23901	28.104	3.5044	0.0070092	499.97	-0.34363	0.033716	-10.192	3.0191	1.5837	9.1184	2.2341
D35	E0	A150	34.029	0.23796	143	2.6141	0.0043244	604.51	-1.5118	0.029786	-50.754	2.5124	1.3307	6.3171	1.8861
D35	E0	A-60	31.723	0.26465	119.87	2.5713	0.0048035	535.3	-0.88822	0.03249	-27.338	2.8268	1.4727	7.9982	2.1182
D35	E0	A-90	0.39727	0.34009	1.1682	3.4486	0.010347	333.31	0.21046	0.048899	4.304	4.4231	2.0586	19.578	3.1096
D35	E0	A-135	30.62	0.17385	176.13	2.4154	0.0029795	810.66	-1.225	0.02187	-56.014	1.8847	0.80891	3.5584	1.2678
D37	E35.5	A30	23.313	0.17569	132.7	2.1886	0.0028616	764.8	0.048908	0.02155	2.2695	1.9958	1.2028	3.9854	1.5773
D37	E35.5	A60	36.664	0.45678	80.267	2.5729	0.0080827	318.33	-1.1619	0.055416	-20.967	4.6129	2.2473	21.293	3.3523
D43	E0	A0	13.724	0.12286	111.71	2.099	0.0020461	1025.8	1.0137	0.015152	66.901	1.4936	0.91547	2.2321	1.19
D43	E0	A30	15.001	0.12207	122.89	2.1494	0.0020636	1041.6	0.73936	0.01518	48.705	1.4713	0.92776	2.1656	1.1894
D43	E0	A45	22.979	0.16382	140.27	2.2986	0.0028081	818.57	-0.28073	0.020529	-13.674	1.8668	0.88263	3.4877	1.326
D43	E0	A60	26.881	0.21425	125.47	2.5404	0.0039598	641.56	-0.82529	0.027019	-30.545	2.3708	1.29	5.6253	1.8092
D43	E0	A90	10.482	0.21281	49.258	3.2662	0.0056554	577.54	1.0761	0.026795	40.161	2.6263	1.5137	6.9004	2.0533
D43	E0	A120	28.072	0.18888	148.62	2.6075	0.0035535	733.78	-1.4051	0.0244	-57.588	2.0787	1.0691	4.3251	1.5435
D43	E0	A135	23.898	0.13871	172.29	2.3921	0.0024582	973.1	-0.98908	0.017983	-55	1.5737	0.72535	2.4791	1.0983
D43	E0	A150	14.416	0.089299	161.43	2.1652	0.001526	1418.8	0.42277	0.011362	37.208	1.0823	0.62166	1.1721	0.83861
D43	E0	A180	9.844	0.12737	77.285	2.1423	0.0022249	962.84	0.96857	0.016189	59.83	1.5903	0.87537	2.5309	1.2127
D43	E30	A0	15.707	0.28156	55.787	2.1968	0.0048626	451.77	0.44456	0.035602	12.487	3.3268	1.417	11.075	2.1913
D43	E30	A30	22.272	0.15562	143.12	2.1916	0.0025549	857.79	0.032093	0.019235	1.6685	1.7826	1.0787	3.179	1.4124
D43	E30	A45	31.496	0.29969	105.09	2.3606	0.0050055	471.6	-1.0494	0.037207	-28.206	3.193	1.284	10.205	2.0602
D43	E30	A60	35.583	0.38719	91.9	2.5537	0.0068321	373.78	-1.2261	0.047385	-25.875	3.9781	2.0156	15.84	2.9385
D43	E30	A90	15.56	0.23739	65.544	3.1004	0.0057831	536.11	0.46617	0.029971	15.554	2.8227	1.5331	7.9697	2.1457
D43	E30	A120	30.974	0.24094	128.55	2.5786	0.0044042	585.48	-1.1362	0.030124	-37.716	2.5917	1.2999	6.7233	1.9087
D43	E30	A135	29.018	0.25634	113.2	2.2996	0.0042334	543.2	-0.72386	0.031722	-22.818	2.787	1.4632	7.7743	2.0878
D43	E30	A150	22.004	0.14881	147.86	2.2089	0.0024666	895.54	-0.078894	0.018546	-4.254	1.7084	0.84577	2.921	1.246
D43	E30	A180	4.5215	0.38949	11.609	2.2271	0.0074399	299.35	1.1184	0.051459	21.735	4.8766	2.427	23.8	3.4635
D43	E45	A0	22.952	0.25805	88.942	2.1471	0.0041418	518.39	0.41054	0.031099	13.201	2.9157	1.7605	8.5078	2.3079
D43	E45	A30	26.475	0.24719	107.1	2.194	0.0039593	554.15	0.013246	0.029795	0.44457	2.7338	1.6215	7.4783	2.1369
D43	E45	A45	33.778	0.37318	90.514	2.3413	0.0061047	383.53	-0.78818	0.045064	-17.49	3.8836	1.7819	15.094	2.6885
D43	E45	A60	40.076	0.60789	65.926	2.7195	0.011184	243.16	-1.3767	0.073333	-18.773	5.8533	2.9496	34.292	4.3105
D43	E45	A90	22.051	0.34295	64.298	2.9817	0.0077127	386.59	0.44032	0.041598	10.585	3.8547	2.0993	14.862	2.9193
D43	E45	A120	34.913	0.29802	117.15	2.6307	0.0054269	484.74	-1.2276	0.03656	-33.578	3.1101	1.3938	9.6829	2.1792
D43	E45	A135	31.536	0.2921	107.96	2.3706	0.004897	484.09	-0.71986	0.035582	-20.231	3.1141	1.43	9.7042	2.2107
D43	E45	A150	28.946	0.17746	163.11	2.345	0.002985	785.6	-0.7513	0.021976	-34.187	1.9439	0.90142	3.7819	1.3729
D43	E45	A180	18.809	0.28214	66.666	2.3403	0.0050807	460.62	-0.066852	0.036004	-1.8568	3.2657	1.4509	10.674	2.2542
D43	E60	A0	25.653	0.37119	69.11	2.1576	0.0059017	365.59	0.82518	0.042952	19.212	4.061	2.4256	16.503	3.2068
D43	E60	A30	28.099	0.42659	65.868	2.1535	0.0066737	322.68	0.68587	0.049063	13.979	4.5561	2.5726	20.774	3.4804
D43	E60	A45	31.881	0.56298	56.629	2.2933	0.0091969	249.35	0.34842	0.064734	5.3823	5.7284	2.8775	32.843	4.1719
D43	E60	A60	20.593	0.94836	21.714	2.2487	0.016809	133.78	2.1502	0.10678	20.136	9.2092	5.1973	84.844	7.0641
D43	E60	A90	10.654	0.41993	25.372	2.6623	0.0091756	290.14	2.8823	0.047169	61.105	5.0151	2.9565	25.162	3.9464
D43	E60	A120	37.936	0.51669	73.42	2.6287	0.0092846	283.13	-0.69297	0.060653	-11.425	5.1257	2.6658	26.301	3.8188
D43	E60	A135	40.578	0.42021	96.568	2.5196	0.0070962	355.06	-1.2846	0.050037	-25.672	4.1727	1.9796	17.43	2.9879
D43	E60	A150	36.466	0.40523	89.988	2.3232	0.0064722	358.95	-0.68735	0.047865	-14.36	4.1308	1.9432	17.079	2.9163
D43	E60	A180	31.137	0.30861	100.89	2.336	0.0051137	456.81	-0.4036	0.037025	-10.901	3.2917	1.3266	10.845	2.1298
D43	E90	A0	-19.195	0.29906	-64.187	3.7845	0.011653	324.76	5.4634	0.034526	158.24	4.5295	2.2054	20.536	3.2854
D43	E-30	A0	16.173	0.13753	117.6	2.1941	0.0023548	931.76	0.26311	0.017467	15.064	1.6428	0.90654	2.6999	1.2581
D43	E-30	A30	17.85	0.18363	97.209	2.1783	0.003089	705.17	1.5784	0.021257	74.25	2.1614	1.3294	4.6748	1.7304
D43	E-30	A45	19.326	0.1744	110.81	2.3367	0.0031151	750.13	0.22959	0.021736	10.562	2.0342	1.095	4.1407	1.5427
D43	E-30	A60	23.259	0.19772	117.63	2.6382	0.0038854	679.01	-0.54095	0.02512	-21.535	2.2429	1.2057	5.0354	1.6928
D43	E-30	A90	5.7875	0.17254	33.544	2.8468	0.0041278	689.65	1.7743	0.021459	82.682	2.2091	1.1126	4.8818	1.6202
D43	E-30	A120	26.372	0.22666	116.35	2.7422	0.0045383	604.23	-1.4721	0.029735	-49.506	2.5135	1.4165	6.3206	1.9353
D43	E-30	A135	25.391	0.25052	101.35	2.4871	0.0045817	542.83	-1.2572	0.032707	-38.44	2.7889	1.2141	7.7844	1.8806
D43	E-30	A150	17.527	0.15865	110.47	2.2217	0.0027265	814.86	0.055991	0.020219	2.7692	1.8751	1.0189	3.5176	1.4191
D43	E-30	A180	16.173	0.13753	117.6	2.1941	0.0023548	931.76	0.26311	0.017467	15.064	1.6428	0.90643	2.7005	1.2581
D43	E-45	A0	18.082	0.17514	103.24	2.2889	0.0030904	740.65	0.6238	0.021495	29.021	2.0598	1.1875	4.246	1.6065
D43	E-45	A30	19.177	0.17095	112.18	2.2733	0.0029733	764.56	0.47292	0.021016	22.503	1.9965	1.0729	3.9889	1.5054
D43	E-45	A45	22.813	0.2036	112.04	2.4033	0.003656	657.35	-0.016326	0.025167	-0.64873	2.3152	1.1172	5.3645	1.6692
D43	E-45	A60	22.505	0.2872	78.361	2.5063	0.0054018	463.97	0.19816	0.035186	5.6318	3.2432	1.7773	10.525	2.4701
D43	E-45	A90	2.5778	0.19328	13.337	2.7593	0.0045919	600.91	2.295	0.023783	96.497	2.527	1.4045	6.3902	1.9337
D43	E-45	A120	30.831	0.28063	109.86	2.8852	0.0057504	501.73	-1.7961	0.036435	-49.295	3.0089	1.4991	9.0588	2.2038
D43	E-45	A135	25.728	0.35011	73.485	2.519	0.0064908	388.1	-1.0299	0.045145	-22.814	3.8407	1.8074	14.762	2.6695
D43	E-45	A150	20.073	0.24301	82.601	2.3014	0.0042609	540.12	-0.10892	0.030789	-3.5376	2.8024	1.4541	7.8579	2.0737
D43	E-45	A180	19.953	0.2204	90.53	2.261	0.0037972	595.42	-0.043165	0.027825	-1.5513	2.5496	1.2708	6.5049	1.8608
D43	E-60	A0	21.661	0.20638	104.96	2.5095	0.0038998	643.48	0.26705	0.025278	10.565	2.3639	1.2909	5.592	1.7969
D43	E-60	A30	23.295	0.22071	105.55	2.4909	0.0040962	608.11	0.076084	0.027054	2.8123	2.4979	1.2756	6.245	1.8477
D43	E-60	A45	24.62	0.19269	127.77	2.5419	0.003615	703.16	-0.14935	0.023714	-6.2983	2.1675	1.1653	4.7049	1.6423
D43	E-60	A60	24.03	0.22276	107.87	2.6421	0.004364	605.43	-0.039598	0.02736	-1.4473	2.5086	1.4243	6.2975	1.9442
D43	E-60	A90	3.092	0.27001	11.451	2.5497	0.0059258	430.27	2.8533	0.031932	89.356	3.4846	2.1083	12.146	2.7629
D43	E-60	A120	24.03	0.22276	107.87	2.6421	0.004364	605.43	-0.039598	0.02736	-1.4473	2.5086	1.4242	6.2967	1.9442
D43	E-60	A135	28.338	0.3422	82.813	2.659	0.0065798	404.12	-0.92464	0.043104	-21.451	3.6974	1.6214	13.68	2.5225
D43	E-60	A150	23.402	0.32615	71.751	2.4517	0.0059725	410.5	-0.17258	0.040642	-4.2464	3.6432	1.8362	13.283	2.6462
D43	E-60	A180	25.482	0.30277	84.165	2.4894	0.0055474	448.76	-0.51041	0.037921	-13.46	3.348	1.7696	11.215	2.5144
D43	E-90	A0	3.1936	0.31505	10.137	3.0034	0.0081588	368.12	3.1678	0.036474	86.852	4.0351	2.3556	16.286	3.1513
D50	E0	A0	15.632	0.16663	93.815	2.0691	0.0027022	765.69	1.8113	0.019283	93.934	1.9936	1.2293	3.9772	1.599
D50	E30	A0	21.404	0.29816	71.788	1.9882	0.0044837	443.43	1.8988	0.033075	57.408	3.3863	1.9688	11.474	2.6303
D50	E45	A0	18.981	0.21	90.382	1.9966	0.0032154	620.96	1.7601	0.023852	73.794	2.4475	1.4701	5.9959	1.9322
D50	E60	A0	20.736	0.4261	48.666	1.9926	0.0064849	307.26	2.5727	0.045535	56.499	4.7633	2.8776	22.707	3.7789
D50	E90	A0	-14.568	0.24661	-59.075	3.7677	0.0091618	411.24	5.8771	0.026353	223.02	3.637	1.8308	13.243	2.6343
D50	E-30	A0	16.367	0.19113	85.634	2.1477	0.003203	670.51	1.8233	0.022	82.88	2.2708	1.3627	5.1604	1.8052
D50	E-30	A45	-16.649	0.70283	-23.688	2.3594	0.017698	133.31	2.9369	0.10137	28.97	9.2298	4.6011	85.259	6.6789
D50	E-30	A60	20.496	0.20406	100.44	2.5787	0.0039933	645.75	-0.70677	0.026664	-26.507	2.3558	1.3604	5.5535	1.8257
D50	E-30	A90	9.3544	0.1675	55.847	2.8387	0.0038944	728.91	1.3249	0.020904	63.381	2.0923	1.0488	4.3788	1.536
D50	E-30	A120	23.552	0.25425	92.631	2.7243	0.0051566	528.31	-1.7566	0.034532	-50.87	2.8629	1.6918	8.2017	2.2671
D50	E-30	A135	33.012	0.45601	72.394	2.9181	0.0093693	311.45	-2.2208	0.059974	-37.029	4.7052	1.9792	22.16	3.1128
D50	E-30	A180	19.047	0.15764	120.82	2.1678	0.0026162	828.6	1.0116	0.018759	53.928	1.8445	1.0193	3.4051	1.4164
D50	E-45	A0	17.998	0.22616	79.582	2.2521	0.003934	572.48	1.7268	0.025925	66.606	2.6489	1.5865	7.0205	2.0969
D50	E-45	A30	18.183	0.1738	104.61	2.2485	0.0030105	746.89	0.74123	0.021162	35.026	2.0429	1.1081	4.1764	1.5494
D50	E-45	A45	18.187	0.21181	85.863	2.414	0.0039425	612.29	-0.34944	0.027548	-12.684	2.4812	1.35	6.1614	1.8716
D50	E-45	A60	3.0246	0.64586	4.6831	2.9629	0.01696	174.7	0.2391	0.092766	2.5774	7.6433	3.9982	58.456	5.5187
D50	E-45	A90	-2.244	0.92343	-2.4301	42.938	0.37836	113.48	3.6404	0.1127	32.302	10.167	5.5428	103.41	7.6922
D50	E-45	A120	39.786	0.52226	76.181	3.3481	0.011815	283.36	-2.67	0.067459	-39.579	5.1219	3.0524	26.254	4.0319
D50	E-45	A135	16.992	0.36024	47.167	2.5896	0.0072924	355.11	-1.1962	0.049787	-24.027	4.1722	2.1276	17.416	3.1066
D50	E-45	A150	19.622	0.24441	80.282	2.2764	0.0042522	535.35	0.084827	0.030709	2.7623	2.8265	1.4766	7.9943	2.0964
D50	E-45	A180	21.243	0.23036	92.215	2.2004	0.0038301	574.51	1.0834	0.026923	40.242	2.6398	1.4033	6.9744	1.9958
D50	E-60	A0	21.924	0.25914	84.602	2.4864	0.0048501	512.63	1.4118	0.029543	47.789	2.9473	1.6064	8.6931	2.2472
D50	E-60	A30	23.907	0.24703	96.777	2.4559	0.004505	545.16	0.87645	0.02874	30.496	2.7773	1.4959	7.7183	2.1023
D50	E-60	A45	24.186	0.21304	113.53	2.4886	0.003926	633.87	0.48493	0.025325	19.148	2.3989	1.3371	5.7583	1.8472
D50	E-60	A60	24.085	0.23653	101.83	2.5956	0.0045522	570.2	0.24901	0.028554	8.7206	2.6591	1.4975	7.0766	2.0605
D50	E-60	A90	5.2287	0.27386	19.093	2.4941	0.0057878	430.93	2.7521	0.032111	85.706	3.4796	2.0742	12.108	2.7419
D50	E-60	A120	31.391	0.33862	92.702	2.8491	0.0068381	416.65	-0.98346	0.041949	-23.444	3.5924	1.796	12.918	2.6297
D50	E-60	A135	27.935	0.34058	82.022	2.5731	0.0063537	404.98	-0.23119	0.041336	-5.5931	3.69	1.5601	13.629	2.4801
D50	E-60	A150	23.596	0.3079	76.637	2.3645	0.0054272	435.67	0.81222	0.03609	22.505	3.4436	1.5638	11.868	2.3934
D50	E-60	A180	23.371	0.28485	82.045	2.3736	0.0050443	470.56	1.1389	0.032744	34.782	3.1998	1.6145	10.246	2.3564
D52	E30	A-30	21.892	0.24578	89.072	2.0198	0.0037361	540.61	1.8064	0.027308	66.148	2.8	1.7121	7.8447	2.2218

Table H. Virtual Random dataset,females stature.
$c_0 = Int \quad c_1 = viewa \quad c_2 = stat$.

Dist	El	Az	Int	Int se	Int t-value	viewa	viewa se	viewa t-value	Stature	Stature se	Stature t-value	R se	CV RMSE	CV MSPE	CV MAPE
D35	E0	A0	15.697	0.1711	91.742	2.0116	0.0029211	688.66	2.2721	0.019407	117.08	2.0498	1.2364	4.2034	1.6315
D35	E0	A30	21.923	0.12487	175.56	2.2362	0.0022624	988.42	0.22905	0.01553	14.749	1.4358	0.83343	2.0627	1.1196
D35	E0	A45	28.413	0.22378	126.97	2.3478	0.004075	576.13	-0.50928	0.027879	-18.267	2.4394	1.1683	5.9558	1.7679
D35	E0	A90	4.9402	0.19483	25.356	3.3324	0.0059878	556.54	0.26643	0.0275	9.6884	2.5227	1.3786	6.3677	1.9104
D35	E0	A150	29.546	0.27038	109.28	2.5034	0.0052155	479.99	-0.82841	0.034103	-24.291	2.9095	1.5043	8.4685	2.157
D35	E0	A-60	31.251	0.32247	96.911	2.5765	0.0063371	406.57	-0.85788	0.040317	-21.278	3.4072	1.8527	11.616	2.603
D35	E0	A-90	-1.0107	0.21505	-4.6999	3.2147	0.0066939	480.25	0.9612	0.030453	31.564	2.908	1.5278	8.4598	2.1683
D35	E0	A-135	27.71	0.18018	153.79	2.3489	0.0032953	712.79	-0.79674	0.022933	-34.742	1.9818	0.97862	3.9291	1.4373
D37	E35.5	A30	25.934	0.20545	126.23	2.2419	0.0036333	617.02	-0.2991	0.025701	-11.638	2.282	1.2283	5.2117	1.7196
D37	E35.5	A60	35.022	0.57934	60.452	2.5985	0.011302	229.92	-1.0807	0.072074	-14.994	5.6912	3.1944	32.408	4.399
D43	E0	A0	10.739	0.11546	93.008	2.0516	0.0020834	984.74	1.3263	0.014505	91.435	1.4411	0.86444	2.0775	1.1465
D43	E0	A30	17.159	0.098912	173.47	2.2071	0.0018299	1206.1	0.37683	0.012608	29.887	1.1786	0.70622	1.3896	0.93759
D43	E0	A45	23.478	0.18551	126.56	2.3256	0.0034612	671.89	-0.38903	0.023735	-16.39	2.0999	0.96408	4.4125	1.4981
D43	E0	A60	26.166	0.27084	96.61	2.5403	0.0054291	467.91	-0.74321	0.034804	-21.354	2.9814	1.5582	8.8942	2.2398
D43	E0	A90	7.418	0.16105	46.062	3.1261	0.0045478	687.39	1.6286	0.020347	80.041	2.0535	1.1765	4.219	1.5961
D43	E0	A120	24.09	0.22351	107.78	2.5017	0.0044712	559.51	-0.73181	0.029095	-25.153	2.5097	1.2437	6.301	1.826
D43	E0	A135	21.625	0.14074	153.65	2.3238	0.0026562	874.85	-0.57912	0.018445	-31.397	1.6199	0.81393	2.6253	1.1799
D43	E0	A150	14.996	0.10662	140.65	2.1691	0.0019699	1101.1	0.35518	0.01383	25.682	1.2901	0.72011	1.6651	0.98117
D43	E0	A180	13.547	0.15436	87.762	2.2003	0.0029276	751.57	0.47404	0.020103	23.581	1.8813	0.98835	3.5413	1.417
D43	E30	A0	17.174	0.21105	81.376	2.0973	0.0037204	563.73	0.6885	0.026422	26.058	2.4915	1.3761	6.2105	1.9135
D43	E30	A30	23.761	0.16716	142.15	2.2329	0.0029871	747.5	-0.19829	0.021088	-9.4028	1.8914	1.0581	3.5792	1.448
D43	E30	A45	34.156	0.33751	101.2	2.4559	0.0061999	396.12	-1.5334	0.043048	-35.621	3.4918	1.6044	12.199	2.4551
D43	E30	A60	34.515	0.50728	68.038	2.5848	0.0098473	262.49	-1.1961	0.063609	-18.803	5.0796	2.7549	25.814	3.8623
D43	E30	A90	6.0397	0.16391	36.848	2.8066	0.0042008	668.12	1.944	0.020477	94.939	2.1115	1.07	4.4606	1.5586
D43	E30	A120	26.539	0.22908	115.85	2.4322	0.0043794	555.38	-0.36312	0.028663	-12.669	2.5278	1.4305	6.3954	1.9602
D43	E30	A135	27.447	0.19312	142.13	2.2808	0.0034368	663.63	-0.56422	0.024288	-23.23	2.1254	1.0957	4.5203	1.5793
D43	E30	A150	21.96	0.13843	158.64	2.2335	0.002505	891.62	-0.12554	0.017602	-7.1322	1.5898	0.8319	2.5293	1.1934
D43	E30	A180	13.488	0.26028	51.82	2.3172	0.0052217	443.76	0.20478	0.034609	5.917	3.136	1.4518	9.8408	2.2311
D43	E45	A0	22.404	0.26779	83.66	2.1109	0.0045822	460.68	0.5487	0.032613	16.824	3.026	1.6534	9.1633	2.3004
D43	E45	A30	30.212	0.31636	95.499	2.27	0.0055161	411.52	-0.45874	0.038886	-11.797	3.3685	1.7546	11.355	2.4969
D43	E45	A45	34.657	0.53945	64.245	2.3814	0.0096516	246.74	-0.95343	0.066685	-14.298	5.3596	2.6331	28.742	3.8712
D43	E45	A60	27.66	0.83019	33.317	2.5063	0.016717	149.93	0.19143	0.10182	1.8801	7.9213	4.8578	62.775	6.3891
D43	E45	A90	10.815	0.25868	41.808	2.6366	0.0060267	437.49	2.0999	0.030898	67.962	3.1787	1.7036	10.109	2.4111
D43	E45	A120	27.811	0.29702	93.634	2.4111	0.0055905	431.28	-0.020858	0.036117	-0.57752	3.2222	1.8888	10.39	2.5342
D43	E45	A135	31.691	0.26035	121.73	2.3594	0.0046621	506.09	-0.69415	0.032088	-21.633	2.7652	1.3862	7.6518	2.0364
D43	E45	A150	30.875	0.19729	156.5	2.3983	0.0036056	665.15	-0.98638	0.024853	-39.689	2.1207	0.99911	4.5011	1.5111
D43	E45	A180	27.279	0.24007	113.63	2.4439	0.0045889	532.57	-0.9495	0.030964	-30.665	2.6325	1.2584	6.9338	1.8779
D43	E60	A0	27.45	0.46612	58.891	2.238	0.0082229	272.16	0.42225	0.055544	7.6021	4.9208	2.65	24.225	3.6999
D43	E60	A30	28.415	0.61882	45.918	2.1884	0.010685	204.8	0.61348	0.072752	8.4324	6.2615	3.4112	39.225	4.7004
D43	E60	A45	17.054	0.89512	19.052	2.0415	0.016078	126.98	2.1828	0.1046	20.869	8.8341	4.9893	78.078	6.7983
D43	E60	A60	1.5886	1.1853	1.3403	1.9384	0.02398	80.835	4.2321	0.13809	30.647	11.111	6.4612	123.5	8.6878
D43	E60	A90	6.6702	0.31569	21.129	2.5431	0.0073532	345.85	3.3456	0.035574	94.045	3.9627	2.1288	15.709	2.9938
D43	E60	A120	24.08	0.44031	54.69	2.285	0.0081185	281.46	1.306	0.050662	25.778	4.7766	2.7971	22.828	3.7544
D43	E60	A135	34.615	0.43459	79.648	2.3548	0.0076571	307.53	-0.25509	0.051338	-4.9687	4.4119	2.4497	19.485	3.3851
D43	E60	A150	39.314	0.44206	88.933	2.39	0.0076579	312.09	-0.95156	0.052773	-18.031	4.3534	2.2768	18.97	3.2448
D43	E60	A180	37.849	0.42482	89.095	2.4359	0.0075707	321.76	-1.0427	0.051471	-20.259	4.2342	2.0791	17.942	3.0754
D43	E90	A0	-33.971	0.42089	-80.714	2.7161	0.015571	174.43	7.802	0.045761	170.49	7.0988	3.866	50.415	5.4419
D43	E-30	A0	20.875	0.17419	119.84	2.3281	0.0033139	702.53	-0.51826	0.022881	-22.651	2.0101	1.0816	4.0429	1.5251
D43	E-30	A30	19.769	0.14457	136.74	2.2227	0.0026452	840.3	1.2099	0.017131	70.625	1.6856	0.89448	2.8439	1.2629
D43	E-30	A45	19.571	0.19659	99.554	2.3367	0.003792	616.22	0.17562	0.024984	7.0292	2.2849	1.1875	5.2241	1.7004
D43	E-30	A60	21.16	0.27205	77.779	2.6057	0.0057992	449.32	-0.25687	0.035184	-7.3008	3.099	1.6805	9.6083	2.35
D43	E-30	A90	4.0112	0.11105	36.119	2.7798	0.002859	972.3	2.0155	0.014005	143.91	1.4593	0.77169	2.1314	1.0943
D43	E-30	A120	20.376	0.36517	55.799	2.5638	0.0077351	331.44	-0.47305	0.048291	-9.7958	4.1209	2.3228	16.988	3.2165
D43	E-30	A135	22.33	0.32198	69.351	2.4217	0.0063373	382.13	-0.82597	0.042808	-19.295	3.6116	1.9254	13.048	2.7209
D43	E-30	A150	19.512	0.20126	96.949	2.2777	0.0037862	601.58	-0.28935	0.026344	-10.983	2.339	1.161	5.4732	1.6922
D43	E-30	A180	20.875	0.17419	119.84	2.3281	0.0033139	702.53	-0.51826	0.022881	-22.651	2.0101	1.0815	4.0428	1.525
D43	E-45	A0	13.836	0.16851	82.109	2.1655	0.00314	689.66	1.0706	0.021065	50.824	2.0469	1.0509	4.1927	1.4834
D43	E-45	A30	21.826	0.21513	101.45	2.3461	0.004101	572.07	-0.016733	0.027237	-0.61435	2.4563	1.0593	6.0374	1.658
D43	E-45	A45	22.033	0.31239	70.53	2.3845	0.0060647	393.17	0.072698	0.039377	1.8462	3.5164	1.8135	12.372	2.5959
D43	E-45	A60	17.716	0.35367	50.093	2.4676	0.0073513	335.66	0.67783	0.044341	15.287	4.0734	2.179	16.604	3.0801
D43	E-45	A90	0.41801	0.13426	3.1135	2.7178	0.003479	781.2	2.5192	0.016805	149.9	1.8111	0.99901	3.282	1.3906
D43	E-45	A120	22.175	0.43299	51.214	2.7249	0.0096522	282.3	-0.60894	0.057128	-10.659	4.764	2.8817	22.702	3.8347
D43	E-45	A135	21.905	0.37428	58.524	2.4464	0.0074832	326.92	-0.49668	0.049027	-10.131	4.1731	2.3017	17.423	3.2239
D43	E-45	A150	22.74	0.23971	94.864	2.3607	0.0045714	516.41	-0.51774	0.031114	-16.64	2.7119	1.4041	7.3592	2.0031
D43	E-45	A180	25.483	0.22038	115.63	2.4234	0.0042282	573.16	-0.96337	0.028797	-33.454	2.4517	1.2233	6.0143	1.7949
D43	E-60	A0	15.301	0.33512	45.657	2.306	0.0066237	348.14	1.0404	0.041745	24.922	3.9386	1.7734	15.523	2.723
D43	E-60	A30	23.328	0.32224	72.393	2.5069	0.0065181	384.6	-0.062101	0.040594	-1.5298	3.5899	1.4954	12.896	2.3865
D43	E-60	A45	23.972	0.30617	78.295	2.5524	0.0062722	406.94	-0.12285	0.038517	-3.1896	3.4043	1.5736	11.602	2.3965
D43	E-60	A60	18.397	0.29244	62.906	2.5516	0.0062353	409.22	0.6657	0.036425	18.276	3.3864	1.8997	11.476	2.6158
D43	E-60	A90	2.5215	0.19158	13.162	2.5878	0.0046598	555.34	2.8491	0.023055	123.58	2.528	1.5682	6.393	2.0322
D43	E-60	A120	18.397	0.29244	62.906	2.5516	0.0062353	409.22	0.6657	0.036425	18.276	3.3864	1.8995	11.473	2.6155
D43	E-60	A135	23.164	0.47849	48.41	2.5969	0.010116	256.71	-0.30301	0.061627	-4.9168	5.1791	3.015	26.83	4.1222
D43	E-60	A150	27.063	0.29904	90.498	2.5643	0.0060186	426.07	-0.75388	0.038238	-19.715	3.2596	1.6714	10.63	2.4151
D43	E-60	A180	25.873	0.23721	109.07	2.4845	0.0046553	533.7	-0.59674	0.030252	-19.726	2.6271	1.2885	6.9069	1.897
D43	E-90	A0	10.898	0.24128	45.169	3.1176	0.0066372	469.72	2.4485	0.028067	87.236	2.9704	1.7445	8.8263	2.3254
D50	E0	A0	13.072	0.13775	94.897	2.0184	0.0024041	839.56	2.0311	0.016199	125.38	1.6871	1.0568	2.8471	1.3631
D50	E30	A0	22.95	0.3028	75.795	2.0279	0.0049643	408.5	1.5957	0.034278	46.553	3.392	1.7955	11.513	2.5507
D50	E45	A0	19.267	0.21712	88.741	1.98	0.0035588	556.36	1.7193	0.024973	68.845	2.5235	1.4351	6.3724	1.9581
D50	E60	A0	24.329	0.49845	48.809	2.0853	0.0083981	248.31	1.9847	0.054721	36.269	5.3303	2.9212	28.424	4.0451
D50	E90	A0	-26.342	0.39893	-66.032	2.8842	0.014358	200.88	7.6194	0.040762	186.92	6.3598	3.4628	40.474	4.8891
D50	E-30	A0	13.251	0.12627	104.95	2.057	0.0022422	917.41	2.0851	0.014769	141.18	1.5456	0.91713	2.3902	1.2239
D50	E-30	A45	-3.5192	0.58915	-5.9733	2.5127	0.015218	165.11	1.4666	0.084996	17.255	7.3945	3.6417	54.702	5.2474
D50	E-30	A60	19.671	0.26376	74.579	2.5866	0.0056406	458.57	-0.60586	0.035219	-17.203	3.0394	1.605	9.2416	2.2786
D50	E-30	A90	6.3488	0.12112	52.417	2.7436	0.0030229	907.59	1.7515	0.015284	114.6	1.5622	0.86001	2.442	1.1892
D50	E-30	A120	19.042	0.36496	52.176	2.5517	0.0077713	328.35	-0.89521	0.050001	-17.904	4.1565	2.2771	17.282	3.2129
D50	E-30	A135	28.836	0.52127	55.32	2.6642	0.010867	245.16	-0.94954	0.067097	-14.152	5.3892	3.0812	29.056	4.1822
D50	E-30	A180	22.226	0.20107	110.54	2.2341	0.0036379	614.11	0.59865	0.024399	24.536	2.2926	1.2408	5.2598	1.7467
D50	E-45	A0	16.038	0.18708	85.726	2.1646	0.0034297	631.12	1.8279	0.021853	83.642	2.2323	1.1783	4.9852	1.6543
D50	E-45	A30	20.765	0.19943	104.12	2.3234	0.0037922	612.68	0.26056	0.024993	10.425	2.2978	1.0069	5.2842	1.5651
D50	E-45	A45	17.545	0.29935	58.61	2.3942	0.0060289	397.13	-0.22711	0.039722	-5.7175	3.4835	1.854	12.141	2.5956
D50	E-45	A60	-1.3338	0.63983	-2.0846	2.8106	0.018225	154.22	0.95736	0.094151	10.168	7.7665	3.9692	60.364	5.5486
D50	E-45	A90	0.8592	0.63537	1.3523	41.78	0.26342	158.61	3.0278	0.078848	38.401	7.613	4.341	57.979	5.8959
D50	E-45	A120	12.4	0.84826	14.618	2.5173	0.019492	129.15	1.8079	0.10571	17.102	8.7415	5.4423	76.435	7.0582
D50	E-45	A135	12.888	0.36212	35.592	2.5168	0.0079709	315.75	-0.64184	0.051204	-12.535	4.3075	2.4549	18.559	3.3643
D50	E-45	A150	21.053	0.23284	90.418	2.3145	0.0044058	525.33	-0.14959	0.029903	-5.0024	2.6675	1.4229	7.1187	2.0022
D50	E-45	A180	24.451	0.2378	102.82	2.2778	0.0043221	527.01	0.61816	0.028389	21.775	2.6593	1.3483	7.0788	1.9632
D50	E-60	A0	19.382	0.31237	62.047	2.3443	0.0060798	385.58	1.7039	0.036036	47.284	3.5813	1.6847	12.832	2.5254
D50	E-60	A30	24.964	0.29202	85.488	2.4838	0.0057769	429.95	0.62503	0.034766	17.979	3.2316	1.4007	10.453	2.2027
D50	E-60	A45	23.405	0.29317	79.835	2.4742	0.0058428	423.47	0.54818	0.035473	15.453	3.2786	1.5457	10.755	2.3333
D50	E-60	A60	19.535	0.29894	65.345	2.509	0.0062165	403.6	0.82924	0.036537	22.696	3.4309	1.929	11.778	2.6536
D50	E-60	A90	3.8549	0.19536	19.732	2.5165	0.0045729	550.29	2.8397	0.023282	121.97	2.5504	1.5868	6.5063	2.0489
D50	E-60	A120	26.047	0.47671	54.639	2.8524	0.010835	263.26	-0.46353	0.060699	-7.6366	5.0666	3.2145	25.678	4.1565
D50	E-60	A135	24.093	0.45111	53.407	2.5366	0.0092379	274.59	0.23163	0.055734	4.156	4.8824	2.8805	23.845	3.8811
D50	E-60	A150	25.343	0.29588	85.654	2.4546	0.0057695	425.44	0.48125	0.035462	13.571	3.2641	1.6685	10.66	2.4198
D50	E-60	A180	23.133	0.26628	86.874	2.3396	0.005023	465.77	1.1832	0.030934	38.25	2.9944	1.5206	8.9711	2.2069
D52	E30	A-30	24.374	0.25968	93.86	2.0602	0.0042744	482	1.4675	0.029323	50.045	2.8978	1.6289	8.4026	2.2365

Table I. Virtual Random, kids. $c_0 = Int \quad c_1 = viewa$.

Dist	El	Az	Int	Int se	Int Tvalue	viewa	viewa se	viewa Tvalue	R se	CV RMSE	CV MSPE	CV MAPE
D35	E0	A0	30.167	0.072896	413.83	2.4968	0.0020444	1221.3	3.1436	1.7418	9.8911	2.4264
D35	E0	A30	22.3	0.036876	604.72	2.2851	0.00088095	2593.9	1.4824	0.79818	2.1985	1.1207
D35	E0	A45	22.689	0.039523	574.06	2.2995	0.00095345	2411.8	1.5942	0.85116	2.5428	1.2051
D35	E0	A90	6.2533	0.043565	143.54	3.3741	0.0013411	2515.8	1.5283	0.84669	2.3365	1.1906
D35	E0	A150	22.024	0.041509	530.59	2.3735	0.0010275	2309.9	1.6644	0.91337	2.772	1.2741
D35	E0	A-60	22.802	0.047663	478.4	2.4653	0.001234	1997.8	1.9241	1.0311	3.7042	1.4661
D35	E0	A-90	4.2089	0.052455	80.239	3.4128	0.0016067	2124.1	1.8099	0.88578	3.2784	1.3337
D35	E0	A-135	21.036	0.031144	675.45	2.2398	0.00072115	3105.9	1.2382	0.66752	1.5338	0.93629
D37	E35.5	A30	22.092	0.03907	565.45	2.2011	0.00089742	2452.7	1.5676	0.81982	2.4584	1.1777
D37	E35.5	A60	24.414	0.069087	353.38	2.4237	0.0017845	1358.2	2.8279	1.4904	8.0035	2.1358
D43	E0	A0	18.642	0.04015	464.31	2.3022	0.0009358	2460.1	1.5629	0.91736	2.4444	1.2288
D43	E0	A30	18.541	0.027693	669.52	2.2803	0.00063873	3570.1	1.0772	0.57644	1.1613	0.81538
D43	E0	A45	19.019	0.029773	638.81	2.2899	0.00069244	3307	1.1629	0.6377	1.3533	0.88924
D43	E0	A60	19.233	0.039058	492.41	2.4441	0.00097145	2516	1.5282	0.85416	2.3369	1.1809
D43	E0	A90	16.488	0.05109	322.72	3.5611	0.0018081	1969.6	1.9516	1.2585	3.8108	1.6014
D43	E0	A120	17.688	0.030794	574.41	2.3863	0.00073779	3234.4	1.189	0.66031	1.4146	0.91099
D43	E0	A135	16.686	0.02133	782.3	2.2526	0.00047824	4710.2	0.81657	0.43963	0.66718	0.61898
D43	E0	A150	16.107	0.021468	750.3	2.249	0.00047818	4703.3	0.81777	0.45475	0.66911	0.62982
D43	E0	A180	15.14	0.026129	579.42	2.3194	0.00059528	3896.3	0.98709	0.53586	0.97507	0.74788
D43	E30	A0	20.573	0.041062	501.02	2.2319	0.00094363	2365.2	1.6256	0.92321	2.6434	1.2597
D43	E30	A30	21.174	0.034407	615.41	2.199	0.00078315	2807.9	1.3695	0.7253	1.8759	1.0325
D43	E30	A45	21.444	0.041888	511.93	2.2292	0.00096888	2300.8	1.671	0.85988	2.7934	1.2329
D43	E30	A60	23.374	0.061683	378.94	2.3962	0.0015604	1535.6	2.502	1.3496	6.2637	1.9088
D43	E30	A90	19.499	0.054538	357.54	3.2341	0.0017993	1797.4	2.1382	1.4367	4.5758	1.7825
D43	E30	A120	21.337	0.044153	483.27	2.4172	0.0011063	2184.8	1.7596	0.95812	3.0979	1.3434
D43	E30	A135	21.047	0.038447	547.42	2.2333	0.00088777	2515.6	1.5285	0.81599	2.3383	1.1558
D43	E30	A150	19.466	0.024258	802.44	2.2295	0.00055143	4043.1	0.95128	0.45136	0.90548	0.68587
D43	E30	A180	17.123	0.029649	577.53	2.3119	0.00068486	3375.8	1.1392	0.54509	1.2988	0.7902
D43	E45	A0	23.349	0.052081	448.33	2.2431	0.0012329	1819.3	2.1126	1.1369	4.4664	1.6047
D43	E45	A30	24.216	0.051603	469.27	2.2094	0.0012126	1822	2.1095	1.1394	4.452	1.6024
D43	E45	A45	24.843	0.058386	425.49	2.2412	0.0013998	1601.1	2.3998	1.2488	5.7612	1.7862
D43	E45	A60	25.976	0.084642	306.89	2.5169	0.0023032	1092.8	3.5116	1.836	12.339	2.6402
D43	E45	A90	23.825	0.066975	355.72	3.1343	0.0022253	1408.5	2.7272	1.5714	7.4413	2.123
D43	E45	A120	24.563	0.057473	427.39	2.4665	0.0015125	1630.7	2.3564	1.3006	5.5553	1.8118
D43	E45	A135	23.807	0.05041	472.27	2.2971	0.0012271	1872	2.0532	1.0557	4.2187	1.5302
D43	E45	A150	22.206	0.0361	615.13	2.2551	0.00085038	2651.9	1.45	0.73596	2.1032	1.0713
D43	E45	A180	20.907	0.029614	705.99	2.2768	0.00069624	3270.1	1.176	0.62979	1.3836	0.88403
D43	E60	A0	26.981	0.070322	383.68	2.3434	0.0017976	1303.7	2.9457	1.5798	8.6836	2.2301
D43	E60	A30	28.716	0.081342	353.03	2.301	0.0020747	1109.1	3.4603	1.8785	11.984	2.6057
D43	E60	A45	29.621	0.093624	316.38	2.4007	0.0025129	955.34	4.0139	2.1506	16.118	3.0426
D43	E60	A60	30.809	0.12819	240.33	2.6285	0.0038126	689.43	5.546	3.04	30.774	4.2119
D43	E60	A90	28.07	0.1006	279.03	3.4218	0.0037947	901.75	4.2508	2.4888	18.084	3.3284
D43	E60	A120	29.737	0.096743	307.38	2.6048	0.0028206	923.49	4.1514	2.3962	17.247	3.2511
D43	E60	A135	28.895	0.082318	351.02	2.3698	0.002166	1094.1	3.5075	1.9183	12.31	2.6752
D43	E60	A150	28.419	0.077514	366.63	2.2672	0.0019425	1167.1	3.289	1.8302	10.823	2.5195
D43	E60	A180	26.776	0.063409	422.27	2.2921	0.0015823	1448.6	2.6518	1.3837	7.0365	1.9767
D43	E90	A0	22.284	0.12883	172.97	6.0037	0.0080961	741.55	5.1603	2.7777	26.644	3.8638
D43	E-30	A0	16.193	0.030032	539.18	2.2614	0.00067312	3359.5	1.1447	0.6494	1.3112	0.88551
D43	E-30	A30	25.796	0.051912	496.92	2.5119	0.0014069	1785.5	2.1525	1.1784	4.6378	1.6623
D43	E-30	A45	18.346	0.030806	595.52	2.4207	0.00075299	3214.8	1.1962	0.64449	1.4323	0.91019
D43	E-30	A60	18.335	0.036949	496.23	2.5874	0.00096526	2680.5	1.4345	0.81177	2.0591	1.1076
D43	E-30	A90	16.876	0.041894	402.82	3.2632	0.001363	2394.1	1.606	0.92083	2.5816	1.2424
D43	E-30	A120	16.523	0.030385	543.77	2.4506	0.00074014	3311.1	1.1615	0.59521	1.3499	0.85899
D43	E-30	A135	16.362	0.029666	551.53	2.2744	0.00066974	3396	1.1324	0.5801	1.2832	0.83308
D43	E-30	A150	16.654	0.025848	644.31	2.2317	0.00057401	3887.9	0.98923	0.55692	0.97905	0.76557
D43	E-30	A180	16.193	0.030032	539.18	2.2614	0.00067312	3359.5	1.1447	0.64959	1.3116	0.88569
D43	E-45	A0	19.398	0.047336	409.79	2.4344	0.0011744	2072.9	1.8545	1.1487	3.4412	1.4933
D43	E-45	A30	19.952	0.037539	531.5	2.3918	0.00091944	2601.4	1.4781	0.80878	2.1864	1.137
D43	E-45	A45	20.073	0.044411	451.98	2.4558	0.0011181	2196.4	1.7503	1.0012	3.0655	1.3617
D43	E-45	A60	19.295	0.046327	416.5	2.6698	0.0012594	2119.9	1.8134	1.0237	3.291	1.4024
D43	E-45	A90	16.396	0.050264	326.19	3.3262	0.0016601	2003.6	1.9186	1.0354	3.6835	1.4309
D43	E-45	A120	17.508	0.037515	466.69	2.5536	0.00096037	2659	1.4461	0.74381	2.0931	1.0647
D43	E-45	A135	17.63	0.032367	544.69	2.343	0.00076102	3078.7	1.2491	0.6318	1.5612	0.90811
D43	E-45	A150	17.919	0.029799	601.33	2.284	0.0006847	3335.8	1.1529	0.65172	1.3299	0.89092
D43	E-45	A180	17.885	0.03365	531.5	2.2716	0.00076877	2954.8	1.3014	0.72307	1.6951	1.0019
D43	E-60	A0	20.3	0.045532	445.84	2.6097	0.0012206	2138	1.7981	1.0717	3.2358	1.4258
D43	E-60	A30	20.849	0.039608	526.38	2.5561	0.001045	2446.1	1.5718	0.83201	2.4719	1.189
D43	E-60	A45	21.28	0.041453	513.34	2.5567	0.0010981	2328.3	1.6513	0.87388	2.728	1.2356
D43	E-60	A60	21.524	0.04758	452.39	2.6849	0.0013265	2024.1	1.8992	1.0949	3.6085	1.4841
D43	E-60	A90	20.878	0.070404	296.55	3.2255	0.0023452	1375.4	2.7926	1.6994	7.803	2.2048
D43	E-60	A120	21.524	0.04758	452.39	2.6849	0.0013265	2024.1	1.8992	1.0949	3.6089	1.4841
D43	E-60	A135	19.671	0.043036	457.08	2.515	0.0011057	2274.6	1.6902	0.87183	2.858	1.2456
D43	E-60	A150	19.991	0.040004	499.73	2.4462	0.0010024	2440.2	1.5756	0.83823	2.4841	1.1803
D43	E-60	A180	19.726	0.046774	421.72	2.4219	0.0011578	2091.8	1.8378	1.0374	3.3793	1.4203
D43	E-90	A0	27.039	0.083378	324.29	3.8288	0.0034848	1098.7	3.4928	2.0551	12.209	2.7559
D50	E0	A0	25.843	0.055763	463.44	2.4318	0.0014637	1661.4	2.3129	1.2774	5.3545	1.7812
D50	E30	A0	31.375	0.078759	398.37	2.3732	0.0021234	1117.6	3.4339	1.8285	11.803	2.6041
D50	E45	A0	28.6	0.063823	448.11	2.3428	0.0016552	1415.4	2.7138	1.4343	7.3704	2.0461
D50	E60	A0	35.526	0.10958	324.2	2.5157	0.0032592	771.86	4.9597	2.8009	24.611	3.8772
D50	E90	A0	30.889	0.14888	207.48	6.5062	0.010976	592.79	6.4365	3.7446	41.466	4.9825
D50	E-30	A0	25.765	0.062028	415.37	2.5303	0.001693	1494.6	2.5705	1.4943	6.6132	2.0128
D50	E-30	A45	16.022	0.053773	297.96	2.5595	0.0013623	1878.8	2.0458	1.0318	4.1891	1.4381
D50	E-30	A60	14.925	0.034656	430.65	2.5008	0.00084977	2942.9	1.3067	0.73315	1.708	1.0021
D50	E-30	A90	17.129	0.037838	452.7	3.1535	0.0011923	2644.9	1.4538	0.83434	2.115	1.1265
D50	E-30	A120	12.666	0.031468	402.49	2.3733	0.00071836	3303.7	1.1641	0.54064	1.3561	0.82673
D50	E-30	A135	19.758	0.050148	393.99	2.4744	0.0012686	1950.4	1.9708	1.019	3.8847	1.4748
D50	E-30	A180	24.079	0.048066	500.95	2.3753	0.0012128	1958.5	1.9627	1.073	3.856	1.5074
D50	E-45	A0	26.505	0.064355	411.85	2.6322	0.0018396	1430.9	2.6846	1.5299	7.2138	2.0925
D50	E-45	A30	20.586	0.039895	516.01	2.4225	0.00099519	2434.2	1.5795	0.88956	2.4969	1.2277
D50	E-45	A45	13.835	0.038797	356.58	2.4123	0.00090922	2653.2	1.4493	0.79992	2.1018	1.0944
D50	E-45	A60	14.132	0.047629	296.7	2.8107	0.0013038	2155.7	1.7833	0.93314	3.1825	1.3076
D50	E-45	A90	22.326	0.11189	199.53	52.314	0.06127	853.83	4.4875	2.3804	20.15	3.363
D50	E-45	A120	21.029	0.089979	233.71	2.7784	0.0025859	1074.5	3.5712	2.2769	12.758	2.9106
D50	E-45	A135	9.2846	0.02751	337.5	2.3561	0.00060616	3886.9	0.98949	0.48389	0.97936	0.69799
D50	E-45	A150	18.624	0.030608	608.45	2.2947	0.00071094	3227.7	1.1914	0.66119	1.4205	0.91396
D50	E-45	A180	26.042	0.059756	435.8	2.4384	0.0015757	1547.5	2.4827	1.4373	6.1687	1.9601
D50	E-60	A0	28.659	0.066818	428.91	2.834	0.0020974	1351.2	2.8424	1.6047	8.0844	2.216
D50	E-60	A30	26.648	0.055486	480.26	2.6889	0.0016222	1657.6	2.3183	1.2582	5.3776	1.7726
D50	E-60	A45	24.776	0.05012	494.33	2.6342	0.0014113	1866.5	2.0592	1.1153	4.2427	1.5716
D50	E-60	A60	23.222	0.050267	461.98	2.6982	0.0014298	1887.1	2.0368	1.1033	4.1514	1.5538
D50	E-60	A90	21.992	0.073831	297.86	3.1469	0.0024233	1298.6	2.9571	1.8044	8.7541	2.3412
D50	E-60	A120	21.499	0.048529	443.01	2.7006	0.0013606	1984.9	1.9366	1.0169	3.7537	1.4525
D50	E-60	A135	23.508	0.050634	464.27	2.5725	0.0013767	1868.7	2.0569	1.1045	4.2343	1.5651
D50	E-60	A150	25.961	0.057725	449.74	2.5844	0.001612	1603.2	2.3967	1.3382	5.7486	1.8684
D50	E-60	A180	28.031	0.069067	405.86	2.6634	0.0020259	1314.7	2.9211	1.7074	8.5401	2.3215
D52	E30	A-30	32.595	0.079548	409.75	2.3541	0.0021519	1094	3.5078	1.9352	12.309	2.7136

Table J. Virtual Random, kids stature.

$c_0 = Int \quad c_1 = viewa \quad c_2 = stat.$

Dist	El	Az	Int	Int se	Int t-value	viewa	viewa se	viewa t-value	Stature	Stature se	Stature t-value	R se	CV RMSE	CV MSPE	CV MAPE
D35	E0	A0	12.312	0.21703	56.729	2.0188	0.0058241	346.63	2.5039	0.029656	84.433	2.1042	1.3084	4.4307	1.7044
D35	E0	A30	17.734	0.14881	119.18	2.1615	0.0040036	539.9	0.70308	0.022308	31.517	1.3696	0.83684	1.8767	1.1011
D35	E0	A45	18.777	0.1675	112.1	2.1933	0.0045209	485.15	0.60003	0.025031	23.972	1.5206	0.84696	2.3138	1.164
D35	E0	A90	3.2371	0.13297	24.346	3.2251	0.0063688	506.4	0.57391	0.024032	23.881	1.4583	0.76091	2.1278	1.0878
D35	E0	A150	19.955	0.18402	108.44	2.315	0.0051752	447.32	0.32022	0.027759	11.536	1.6457	0.91733	2.712	1.2667
D35	E0	A-60	20.649	0.21582	95.679	2.4026	0.0062553	384.09	0.33015	0.032297	10.222	1.9072	1.025	3.6408	1.448
D35	E0	A-90	1.1735	0.15561	7.541	3.2563	0.0077414	420.64	0.59568	0.028876	20.629	1.7469	0.98211	3.0541	1.3469
D35	E0	A-135	20.1	0.13737	146.32	2.2145	0.0036859	600.81	0.14665	0.020956	6.9981	1.2331	0.67782	1.5218	0.94117
D37	E35.5	A30	18.184	0.16315	111.45	2.0989	0.004242	494.79	0.60358	0.024537	24.599	1.4917	0.86552	2.2267	1.1757
D37	E35.5	A60	17.987	0.30395	59.178	2.2432	0.0085035	263.8	0.96682	0.044621	21.667	2.7199	1.3817	7.4053	1.9999
D43	E0	A0	10.946	0.11629	94.126	2.0838	0.0032646	638.3	1.2362	0.018054	68.469	1.1617	0.70217	1.3503	0.92524
D43	E0	A30	13.976	0.092767	150.66	2.1515	0.002592	830.05	0.73536	0.014476	50.8	0.89588	0.53138	0.80327	0.71002
D43	E0	A45	15.524	0.1133	137.01	2.1912	0.0031676	691.76	0.56015	0.017616	31.798	1.073	0.59355	1.1528	0.81719
D43	E0	A60	17.032	0.16231	104.94	2.3779	0.0048441	490.88	0.3521	0.025234	13.953	1.5033	0.81704	2.2618	1.1423
D43	E0	A90	6.0774	0.11662	52.111	3.0934	0.0051626	599.19	1.7145	0.018453	92.914	1.2363	0.67813	1.5296	0.94778
D43	E0	A120	16.621	0.12647	131.42	2.3543	0.003752	627.49	0.17412	0.020022	8.6964	1.1814	0.64958	1.3971	0.90163
D43	E0	A135	16.068	0.086186	186.44	2.2349	0.002441	915.56	0.10212	0.013802	7.3989	0.81281	0.43398	0.6614	0.613
D43	E0	A150	13.286	0.074617	178.06	2.1679	0.0021174	1023.9	0.46894	0.011991	39.108	0.72733	0.40085	0.52927	0.5572
D43	E0	A180	12.967	0.096624	134.2	2.2541	0.0028627	787.41	0.3659	0.015719	23.278	0.94395	0.50237	0.89196	0.7126
D43	E30	A0	14.783	0.15183	97.37	2.0758	0.0040594	511.37	0.90973	0.023156	39.287	1.4444	0.87455	2.0878	1.1497
D43	E30	A30	17.875	0.1421	125.79	2.1118	0.0037288	566.36	0.51532	0.021591	23.867	1.3068	0.76007	1.7085	1.0317
D43	E30	A45	20.232	0.18678	108.32	2.1968	0.0049694	442.06	0.1889	0.02838	6.6562	1.6648	0.85649	2.7735	1.2245
D43	E30	A60	18.529	0.27167	68.205	2.26	0.0076001	297.36	0.73783	0.040353	18.284	2.4329	1.2758	5.9242	1.8172
D43	E30	A90	7.6555	0.12782	59.891	2.7691	0.0049671	557.48	1.8781	0.019546	96.088	1.3272	0.73694	1.7624	1.0187
D43	E30	A120	17.168	0.18226	94.196	2.2963	0.0052474	437.6	0.64991	0.027636	23.517	1.6812	0.92717	2.829	1.2808
D43	E30	A135	18.199	0.16273	111.84	2.1567	0.0043465	496.18	0.44556	0.02478	17.98	1.4876	0.80709	2.2157	1.127
D43	E30	A150	16.942	0.096066	176.36	2.1604	0.0026043	829.57	0.40229	0.014877	27.041	0.89639	0.46917	0.80442	0.67141
D43	E30	A180	15.475	0.11819	130.93	2.2637	0.0034159	662.7	0.27079	0.018816	14.391	1.1195	0.53019	1.2547	0.76564
D43	E45	A0	15.284	0.19648	77.791	2.0319	0.0051186	396.97	1.2255	0.029042	42.196	1.8475	1.1115	3.4151	1.4669
D43	E45	A30	18.149	0.21752	83.436	2.0541	0.0055452	370.44	0.91364	0.031942	28.603	1.9746	1.1742	3.9022	1.5644
D43	E45	A45	19.509	0.26056	74.874	2.1035	0.0067065	313.65	0.79814	0.038074	20.963	2.3138	1.237	5.357	1.7312
D43	E45	A60	16.607	0.37238	44.596	2.2485	0.010645	211.22	1.3846	0.053745	25.763	3.3263	1.6573	11.076	2.4134
D43	E45	A90	10.495	0.20722	50.65	2.6518	0.0074654	355.21	2.0093	0.030296	66.323	2.0556	1.2172	4.2284	1.6207
D43	E45	A120	15.89	0.22482	70.677	2.2202	0.0063602	349.07	1.2996	0.032812	39.609	2.0902	1.1648	4.3718	1.5972
D43	E45	A135	18.58	0.21601	86.015	2.1572	0.0057542	374.89	0.79132	0.031883	24.82	1.9521	1.031	3.8145	1.4646
D43	E45	A150	20.32	0.16032	126.74	2.2046	0.0042739	515.82	0.29117	0.024131	12.066	1.4322	0.75774	2.0529	1.078
D43	E45	A180	19.44	0.12845	151.35	2.2365	0.003506	637.9	0.23001	0.01961	11.73	1.1624	0.63819	1.3522	0.88301
D43	E60	A0	15.29	0.27598	55.404	2.0369	0.0072276	281.82	1.7036	0.03922	43.438	2.5582	1.5152	6.5496	2.0171
D43	E60	A30	14.099	0.31522	44.727	1.9322	0.0079576	242.81	2.0886	0.043949	47.524	2.9348	1.739	8.6209	2.3078
D43	E60	A45	12.677	0.36434	34.796	1.9589	0.009513	205.91	2.3976	0.050317	47.649	3.4018	1.8211	11.579	2.5499
D43	E60	A60	8.3641	0.51513	16.237	1.9929	0.014622	136.3	3.1421	0.070431	44.612	4.7842	2.5233	22.902	3.6045
D43	E60	A90	5.357	0.26755	20.022	2.5723	0.010013	256.9	3.2535	0.037141	87.596	2.7872	1.6835	7.7731	2.2223
D43	E60	A120	9.7508	0.33017	29.532	2.0425	0.0093067	219.47	2.8191	0.045358	62.153	3.2149	1.8602	10.343	2.517
D43	E60	A135	14.917	0.33207	44.92	2.0068	0.0086286	232.58	1.9946	0.046267	43.11	3.0517	1.734	9.3199	2.3639
D43	E60	A150	16.553	0.32696	50.628	1.9704	0.0081784	240.93	1.7031	0.045853	37.144	2.9557	1.737	8.7438	2.3169
D43	E60	A180	18.304	0.27259	67.151	2.0738	0.0070156	295.59	1.2389	0.038934	31.82	2.4466	1.3781	5.9901	1.872
D43	E90	A0	-5.2524	0.19008	-27.633	4.0991	0.013041	314.33	4.1845	0.027524	152.03	2.309	1.1948	5.3372	1.6906
D43	E-30	A0	13.225	0.11054	119.64	2.1756	0.0031521	690.2	0.49297	0.017751	27.772	1.0754	0.60013	1.1574	0.82575
D43	E-30	A30	13.801	0.15427	89.462	2.1717	0.0043657	497.44	1.768	0.022119	79.933	1.4839	0.88753	2.2036	1.1851
D43	E-30	A45	15.831	0.12221	129.54	2.345	0.003644	643.53	0.40661	0.019169	21.211	1.1524	0.65709	1.3296	0.89511
D43	E-30	A60	17.178	0.15421	111.4	2.55	0.0049264	517.62	0.18728	0.024244	7.7249	1.4273	0.80728	2.0389	1.0998
D43	E-30	A90	8.0414	0.097237	82.7	2.9007	0.0039359	736.98	1.4491	0.015356	94.367	1.0079	0.54326	1.0173	0.75761
D43	E-30	A120	16.417	0.12402	132.36	2.4473	0.0038336	638.39	0.017599	0.019921	0.88345	1.1615	0.5944	1.3507	0.85834
D43	E-30	A135	16.357	0.12081	135.39	2.2743	0.0034725	654.95	0.00080788	0.019443	0.041552	1.1325	0.5802	1.2839	0.83328
D43	E-30	A150	14.592	0.099358	146.86	2.1732	0.0027843	780.52	0.34065	0.015889	21.439	0.95221	0.56867	0.90724	0.75619
D43	E-30	A180	13.225	0.11054	119.64	2.1756	0.0031521	690.2	0.49297	0.017751	27.772	1.0754	0.60014	1.1575	0.82576
D43	E-45	A0	13.333	0.17377	76.731	2.2535	0.0051341	438.93	0.9667	0.026846	36.009	1.6763	0.99112	2.8123	1.3259
D43	E-45	A30	15.463	0.14417	107.26	2.261	0.0041645	542.93	0.71105	0.022169	32.073	1.3621	0.75207	1.8565	1.0446
D43	E-45	A45	15.016	0.1718	87.403	2.3048	0.0050889	452.9	0.79989	0.02638	30.322	1.626	0.89256	2.6458	1.2312
D43	E-45	A60	13.579	0.1718	79.042	2.4825	0.005575	445.29	0.91245	0.026582	34.326	1.653	0.91895	2.7355	1.2601
D43	E-45	A90	6.4637	0.12103	53.406	2.9086	0.0050137	580.13	1.6382	0.019187	85.383	1.2763	0.6697	1.631	0.95189
D43	E-45	A120	17.268	0.15642	110.4	2.5459	0.004984	510.82	0.039281	0.024851	1.5807	1.4459	0.74382	2.0933	1.0642
D43	E-45	A135	15.88	0.13052	121.67	2.2914	0.0038013	602.8	0.28571	0.02066	13.829	1.229	0.62844	1.5121	0.89389
D43	E-45	A150	15.433	0.1169	132.02	2.213	0.0033042	669.74	0.40408	0.018423	21.933	1.1078	0.65149	1.2281	0.87077
D43	E-45	A180	14.023	0.12464	112.51	2.1619	0.0034996	617.76	0.62762	0.019618	31.992	1.1997	0.68964	1.4406	0.93102
D43	E-60	A0	16.133	0.18431	87.531	2.4776	0.0057948	427.56	0.65778	0.028267	23.27	1.7195	0.99692	2.9598	1.3491
D43	E-60	A30	17.754	0.16599	106.96	2.4606	0.0050846	483.94	0.48533	0.025326	19.163	1.5243	0.83052	2.3254	1.1641
D43	E-60	A45	16.677	0.16642	100.21	2.4156	0.005067	476.74	0.71764	0.025231	28.443	1.5468	0.86354	2.3943	1.1838
D43	E-60	A60	15.721	0.1869	84.116	2.4987	0.0059552	419.59	0.9019	0.028233	31.945	1.7512	1.0783	3.0682	1.4069
D43	E-60	A90	7.8464	0.20727	37.857	2.7229	0.0079252	343.58	2.0338	0.03125	65.081	2.122	1.3155	4.5052	1.7089
D43	E-60	A120	15.721	0.1869	84.116	2.4987	0.0059552	419.59	0.9019	0.028233	31.945	1.7512	1.0784	3.0688	1.4071
D43	E-60	A135	17.069	0.17956	95.058	2.4348	0.0054876	443.7	0.41418	0.027778	14.91	1.6588	0.86939	2.7541	1.2241
D43	E-60	A150	16.003	0.1592	100.52	2.3273	0.0047055	494.59	0.63172	0.024491	25.794	1.4923	0.82185	2.2282	1.1315
D43	E-60	A180	16.692	0.19423	85.937	2.3319	0.0057122	408.24	0.48254	0.030023	16.072	1.7982	1.0265	3.2365	1.3929
D43	E-90	A0	6.6506	0.1891	35.17	2.9666	0.0079913	371.22	2.9525	0.026523	111.32	1.9706	1.1633	3.8857	1.5541
D50	E0	A0	12.994	0.1634	79.527	2.0794	0.0044714	465.05	1.8922	0.023397	80.875	1.5847	0.98724	2.513	1.2808
D50	E30	A0	12.645	0.25387	49.807	1.9024	0.006404	297.06	2.5938	0.034294	75.633	2.4352	1.4793	5.935	1.9424
D50	E45	A0	13.799	0.20199	68.316	1.9637	0.0051764	379.36	2.1134	0.028104	75.2	1.93	1.1718	3.7271	1.5482
D50	E60	A0	7.8787	0.33167	23.755	1.8132	0.0085019	213.27	3.6607	0.042842	85.447	3.2979	1.9771	10.883	2.6133
D50	E90	A0	-5.6232	0.15255	-36.862	4.0468	0.010357	390.74	5.0213	0.020112	249.67	1.8759	0.9974	3.5231	1.4125
D50	E-30	A0	11.921	0.18393	64.812	2.135	0.0052384	407.58	2.0402	0.026337	77.464	1.8011	1.0709	3.2455	1.4291
D50	E-30	A45	24.556	0.21192	115.88	2.8428	0.0069623	408.31	-1.4303	0.034622	-41.311	1.7979	0.92771	3.2363	1.2818
D50	E-30	A60	14.792	0.13714	107.86	2.4965	0.0044077	566.4	0.022409	0.022443	0.99848	1.3067	0.73193	1.7086	1.001
D50	E-30	A90	9.6605	0.10056	96.068	2.8577	0.003931	726.96	1.2224	0.015872	77.016	1.0216	0.57055	1.0446	0.78362
D50	E-30	A120	15.653	0.11625	134.65	2.4687	0.0036563	675.2	-0.52144	0.019619	-26.578	1.099	0.53304	1.209	0.79456
D50	E-30	A135	21.938	0.2245	97.72	2.5408	0.0067851	374.47	-0.34758	0.034903	-9.9585	1.9542	0.96974	3.8207	1.4376
D50	E-30	A180	13.669	0.14698	93.002	2.0902	0.0040087	521.4	1.5657	0.021481	72.886	1.4172	0.83026	2.0107	1.1154
D50	E-45	A0	13.428	0.21794	61.612	2.2463	0.0064211	349.84	1.9128	0.031029	61.646	2.0858	1.2122	4.3534	1.6418
D50	E-45	A30	14.475	0.14306	101.18	2.2438	0.0041488	540.84	0.95974	0.021805	44.015	1.3672	0.76549	1.871	1.0545
D50	E-45	A45	12.25	0.14625	83.758	2.3619	0.0045842	515.21	0.27175	0.024196	11.231	1.4338	0.7581	2.0581	1.06
D50	E-45	A60	20.704	0.1802	114.9	3.0558	0.0066331	460.69	-1.128	0.030043	-37.545	1.5993	0.88128	2.5597	1.2141
D50	E-45	A90	12.162	0.4758	25.562	45.965	0.29546	155.57	1.5714	0.071659	21.929	4.3123	2.1879	18.61	3.1826
D50	E-45	A120	17.607	0.40501	43.471	2.6629	0.013572	196.21	0.5377	0.062068	8.6631	3.5486	2.2272	12.598	2.869
D50	E-45	A135	12.163	0.09226	131.83	2.4518	0.0030015	816.86	-0.52636	0.016224	-32.443	0.91023	0.44814	0.82922	0.6478
D50	E-45	A150	14.596	0.11183	130.51	2.1803	0.0031447	693.32	0.64845	0.017451	37.157	1.0706	0.63533	1.147	0.84724
D50	E-45	A180	12.613	0.17967	70.202	2.0699	0.0049191	420.8	1.9732	0.025666	76.878	1.7463	1.0632	3.0517	1.3962
D50	E-60	A0	14.558	0.23431	62.133	2.3967	0.0072711	329.62	2.0137	0.03263	61.712	2.2075	1.2649	4.8763	1.7203
D50	E-60	A30	16.142	0.20512	78.697	2.3721	0.0061775	383.98	1.5357	0.029233	52.534	1.9077	1.1015	3.6414	1.4969
D50	E-60	A45	15.687	0.18206	86.166	2.3597	0.0054796	430.63	1.3576	0.026475	51.276	1.7076	0.97228	2.9182	1.3268
D50	E-60	A60	15.546	0.1907	81.524	2.4561	0.0059854	410.35	1.1682	0.028233	41.378	1.7893	1.0521	3.2032	1.409
D50	E-60	A90	7.6927	0.21329	36.066	2.6166	0.0078547	333.13	2.201	0.031739	69.346	2.1854	1.3572	4.7796	1.7665
D50	E-60	A120	17.626	0.20516	85.916	2.5753	0.006594	390.56	0.60271	0.031079	19.393	1.8767	0.97729	3.5261	1.3872
D50	E-60	A135	15.757	0.19329	81.521	2.3402	0.0057667	405.81	1.1757	0.028531	41.208	1.8086	0.95855	3.2748	1.3384
D50	E-60	A150	13.409	0.18224	73.579	2.2187	0.0052977	418.8	1.8468	0.026082	70.808	1.7544	0.98336	3.0794	1.3415
D50	E-60	A180	12.337	0.21401	57.648	2.2039	0.0062671	351.66	2.2544	0.029923	75.34	2.0755	1.1797	4.3119	1.6043
D52	E30	A-30	12.494	0.24377	51.252	1.8601	0.0060208	308.95	2.7463	0.0325	84.501	2.347	1.4416	5.5102	1.8844

Table K. Virtual Random, adults. $c_0 = Int \quad c_1 = viewa$.

Dist	El	Az	Int	Int se	Int Tvalue	viewa	viewa se	viewa Tvalue	R se	CV RMSE	CV MSPE	CV MAPE
D35	E0	A0	40.099	0.056907	704.63	2.2851	0.00079851	2861.7	2.3386	1.4399	5.4696	1.8757
D35	E0	A30	26.93	0.033802	796.71	2.23	0.00043003	5185.6	1.2913	0.78003	1.6677	1.024
D35	E0	A45	27.415	0.060869	450.4	2.2408	0.00078025	2871.9	2.3302	1.2024	5.4309	1.7104
D35	E0	A90	5.8739	0.092814	63.287	3.4107	0.0016207	2104.5	3.1777	1.7671	10.1	2.43
D35	E0	A150	25.183	0.081415	309.32	2.3633	0.0010875	2173	3.0777	1.7717	9.474	2.3819
D35	E0	A-60	27.732	0.088279	314.14	2.4057	0.0012171	1976.6	3.3825	2.0281	11.443	2.6795
D35	E0	A-90	4.2868	0.12941	33.125	3.4497	0.0022682	1520.9	4.3906	2.1491	19.285	3.1371
D35	E0	A-135	22.467	0.063686	352.78	2.2346	0.00079282	2818.6	2.3743	1.1414	5.6382	1.6764
D37	E35.5	A30	27.114	0.048388	560.35	2.1602	0.00059696	3618.7	1.8499	1.0035	3.4228	1.3728
D37	E35.5	A60	32.55	0.13991	232.65	2.3508	0.001936	1214.2	5.4899	3.0997	30.144	4.2419
D43	E0	A0	22.759	0.045534	499.83	2.2141	0.00056249	3936.3	1.7008	1.0292	2.8931	1.3559
D43	E0	A30	22.958	0.032261	711.63	2.2234	0.00040062	5550.1	1.2066	0.722	1.4561	0.95254
D43	E0	A45	23.204	0.054781	423.57	2.2363	0.00068511	3264.1	2.0507	1.0414	4.2062	1.4979
D43	E0	A60	22.715	0.081485	278.76	2.3981	0.0010901	2199.9	3.0402	1.7931	9.2452	2.3873
D43	E0	A90	22.351	0.073015	306.12	3.4255	0.0013925	2459.9	2.7197	1.5198	7.3985	2.0872
D43	E0	A120	20.011	0.07434	269.18	2.3793	0.00097272	2446	2.7351	1.5084	7.4826	2.0717
D43	E0	A135	17.408	0.055824	311.83	2.2474	0.00068064	3301.8	2.0273	0.95703	4.111	1.4147
D43	E0	A150	19.107	0.033166	576.08	2.2022	0.00039976	5508.9	1.2156	0.70415	1.4777	0.94475
D43	E0	A180	18.187	0.055505	327.66	2.2555	0.00068196	3307.4	2.0239	1.1742	4.0967	1.5837
D43	E30	A0	20.351	0.098135	207.38	2.231	0.0012063	1849.4	3.6142	1.6639	13.069	2.4909
D43	E30	A30	25.395	0.04155	611.2	2.168	0.00050969	4253.5	1.5741	0.88909	2.4781	1.1973
D43	E30	A45	26.478	0.098717	268.22	2.1914	0.0012314	1779.6	3.7554	1.7612	14.107	2.6012
D43	E30	A60	30.419	0.1267	240.1	2.3328	0.0017192	1356.9	4.9173	2.7983	24.185	3.8115
D43	E30	A90	19.868	0.089029	223.16	3.1867	0.0015592	2043.8	3.2716	1.8764	10.706	2.5535
D43	E30	A120	25.266	0.075938	332.71	2.3712	0.0010182	2328.7	2.8725	1.5269	8.2541	2.1554
D43	E30	A135	26.327	0.067199	391.78	2.1697	0.00082918	2616.7	2.5571	1.3286	6.5406	1.8737
D43	E30	A150	24.152	0.041857	577.01	2.1734	0.00051133	4250.5	1.5752	0.80438	2.4817	1.1521
D43	E30	A180	8.6142	0.13181	65.354	2.428	0.001661	1461.8	4.567	2.3572	20.864	3.343
D43	E45	A0	30.484	0.072769	418.92	2.1484	0.00090946	2362.3	2.8318	1.5689	8.0214	2.1603
D43	E45	A30	31.548	0.068676	459.38	2.1459	0.00086235	2488.5	2.6886	1.4049	7.229	1.9515
D43	E45	A45	32.712	0.12305	265.84	2.181	0.001581	1379.5	4.8374	2.2582	23.404	3.3752
D43	E45	A60	36.454	0.18392	198.21	2.4303	0.0026906	903.27	7.3516	4.3285	54.058	5.7746
D43	E45	A90	29.591	0.10487	282.17	2.9979	0.0018202	1647	4.0561	2.1863	16.457	3.0654
D43	E45	A120	28.693	0.090535	316.93	2.4027	0.0012532	1917.3	3.4868	1.7478	12.161	2.5525
D43	E45	A135	29.197	0.078859	370.24	2.2311	0.0010163	2195.2	3.0467	1.463	9.2834	2.1772
D43	E45	A150	25.68	0.060642	423.47	2.2228	0.0007639	2909.8	2.3	1.2033	5.2907	1.695
D43	E45	A180	17.854	0.095049	187.84	2.3374	0.0012083	1934.5	3.4558	1.7396	11.948	2.5243
D43	E60	A0	38.829	0.098521	394.11	2.1973	0.0013199	1664.7	4.0132	2.1442	16.109	2.981
D43	E60	A30	42.349	0.11754	360.3	2.1564	0.0015774	1367.1	4.881	2.3821	23.828	3.4425
D43	E60	A45	44.592	0.18017	247.49	2.2355	0.0025415	879.59	7.546	3.9778	56.956	5.5804
D43	E60	A60	51.931	0.23006	225.73	2.4292	0.003689	658.48	10.009	5.6504	100.21	7.7114
D43	E60	A90	43.071	0.11699	368.16	3.0685	0.0022435	1367.7	4.8789	2.6814	23.807	3.7286
D43	E60	A120	38.148	0.12386	307.99	2.4443	0.001839	1329.1	5.0194	2.8216	25.201	3.8345
D43	E60	A135	34.922	0.11244	310.59	2.2843	0.0015318	1491.3	4.4772	2.3602	20.049	3.3293
D43	E60	A150	36.65	0.10614	345.31	2.1772	0.0013916	1564.6	4.2687	2.1355	18.225	3.0699
D43	E60	A180	33.036	0.10143	325.7	2.2316	0.0013356	1670.8	3.9988	1.8603	15.993	2.7522
D43	E90	A0	27.435	0.35221	77.896	5.3754	0.010861	494.92	13.157	6.5372	173.16	9.6379
D43	E-30	A0	20.041	0.053974	371.31	2.2133	0.00065704	3368.6	1.9871	1.082	3.9498	1.5004
D43	E-30	A30	34.499	0.038026	907.25	2.3471	0.00053086	4421.4	1.5144	0.98942	2.2936	1.2475
D43	E-30	A45	23.672	0.057885	408.94	2.3332	0.0007572	3081.3	2.1721	1.2303	4.7189	1.6589
D43	E-30	A60	19.977	0.084179	237.32	2.5486	0.0011797	2160.4	3.0957	1.6732	9.5855	2.3324
D43	E-30	A90	20.884	0.078325	266.63	3.149	0.0013627	2310.9	2.8946	1.455	8.3804	2.1243
D43	E-30	A120	18.66	0.10549	176.89	2.4826	0.0014303	1735.7	3.8499	2.2331	14.825	3.0166
D43	E-30	A135	17.944	0.10369	173.06	2.2927	0.0012935	1772.5	3.7704	1.9897	14.218	2.8074
D43	E-30	A150	20.169	0.060032	335.96	2.2065	0.00072904	3026.6	2.2114	1.2476	4.8907	1.6817
D43	E-30	A180	20.041	0.053974	371.31	2.2133	0.00065704	3368.6	1.9871	1.082	3.9495	1.5004
D43	E-45	A0	23.4	0.091717	255.13	2.3386	0.001201	1947.3	3.4332	2.3468	11.789	2.8951
D43	E-45	A30	24.463	0.062207	393.25	2.3142	0.00081054	2855.1	2.3439	1.2249	5.4956	1.7106
D43	E-45	A45	25.128	0.083384	301.36	2.3668	0.0011152	2122.3	3.151	1.4881	9.9321	2.2402
D43	E-45	A60	27.606	0.1013	272.51	2.4964	0.0014483	1723.6	3.8767	2.1076	15.034	2.9366
D43	E-45	A90	22.769	0.095376	238.73	3.1517	0.0016775	1878.9	3.5578	1.8294	12.663	2.6496
D43	E-45	A120	21.73	0.11875	182.99	2.5663	0.0016915	1517.2	4.4013	2.6053	19.375	3.4727
D43	E-45	A135	21.208	0.12213	173.66	2.3403	0.0015821	1479.3	4.5133	2.5834	20.375	3.4692
D43	E-45	A150	21.802	0.080454	270.98	2.2574	0.0010083	2238.9	2.9875	1.8047	8.9268	2.3511
D43	E-45	A180	21.889	0.075056	291.64	2.2369	0.0009325	2398.9	2.7887	1.5205	7.7796	2.1122
D43	E-60	A0	24.153	0.13017	185.55	2.5017	0.001831	1366.3	4.8837	2.8767	23.856	3.8774
D43	E-60	A30	25.721	0.09061	283.87	2.4694	0.0012685	1946.8	3.4341	1.5849	11.796	2.4088
D43	E-60	A45	26.262	0.078705	333.68	2.4866	0.0011126	2234.9	2.9928	1.3027	8.9594	2.0425
D43	E-60	A60	27.146	0.083298	325.89	2.5993	0.0012369	2101.5	3.1821	1.8307	10.128	2.4724
D43	E-60	A90	31.931	0.11445	278.98	2.9917	0.0020083	1489.7	4.482	2.5402	20.092	3.4814
D43	E-60	A120	27.146	0.083298	325.89	2.5993	0.0012369	2101.5	3.1821	1.8309	10.127	2.4725
D43	E-60	A135	25.775	0.13057	197.41	2.4759	0.0018335	1350.4	4.9409	2.815	24.418	3.8013
D43	E-60	A150	25.438	0.099248	256.31	2.392	0.0013438	1780	3.7545	2.2083	14.1	2.9227
D43	E-60	A180	23.984	0.089512	267.94	2.3776	0.0011953	1989.1	3.3613	1.845	11.302	2.5455
D43	E-90	A0	33.208	0.12472	266.27	3.6181	0.0026656	1357.3	4.9158	2.5758	24.172	3.6853
D50	E0	A0	33.323	0.057753	576.99	2.2712	0.0007751	2930.2	2.284	1.3777	5.2174	1.8162
D50	E30	A0	43.706	0.059174	738.59	2.1638	0.00080295	2694.9	2.483	1.4227	6.1667	1.9173
D50	E45	A0	38.852	0.048472	801.52	2.1601	0.00063833	3383.9	1.9781	1.1867	3.9136	1.5698
D50	E60	A0	51.918	0.08724	595.12	2.2441	0.0012898	1739.8	3.8409	2.1216	14.754	2.9005
D50	E90	A0	44.282	0.29498	150.12	5.3992	0.010049	537.27	12.171	6.1327	148.16	8.9392
D50	E-30	A0	34.388	0.074585	461.05	2.3403	0.0010377	2255.3	2.9658	2.0983	8.7974	2.5465
D50	E-30	A45	-14.715	0.26637	-55.242	3.0811	0.0038175	807.11	8.2098	3.9742	67.42	5.815
D50	E-30	A60	15.616	0.090135	173.25	2.4773	0.0012004	2063.7	3.2403	1.795	10.502	2.4628
D50	E-30	A90	21.601	0.068061	317.38	3.044	0.0011489	2649.5	2.5255	1.215	6.3799	1.8181
D50	E-30	A120	14.146	0.11753	120.36	2.424	0.0015202	1594.5	4.1891	2.4471	17.552	3.2987
D50	E-30	A135	22.211	0.15972	139.07	2.5141	0.0022349	1124.9	5.9212	3.1456	35.071	4.3704
D50	E-30	A180	30.828	0.045258	681.15	2.2658	0.00059761	3791.4	1.7658	0.98298	3.1188	1.3499
D50	E-45	A0	34.939	0.096742	361.16	2.4331	0.0014038	1733.2	3.8554	2.722	14.866	3.3214
D50	E-45	A30	25.948	0.056718	457.49	2.3239	0.00074804	3106.7	2.1544	1.156	4.6427	1.5932
D50	E-45	A45	17.115	0.09353	182.98	2.3454	0.0011884	1973.5	3.3878	1.7695	11.48	2.4965
D50	E-45	A60	-11.61	0.23665	-49.059	3.2356	0.0036114	895.93	7.4108	4.1193	54.946	5.5935
D50	E-45	A90	32.451	0.32027	101.32	51.647	0.097519	529.61	12.338	7.5047	152.27	9.7985
D50	E-45	A120	32.88	0.19424	169.28	2.7328	0.0031318	872.59	7.6054	4.4441	57.856	5.9171
D50	E-45	A135	11.514	0.13555	84.946	2.3905	0.0017064	1400.9	4.7639	2.9396	22.698	3.7902
D50	E-45	A150	23.251	0.077161	301.33	2.2555	0.00097362	2316.6	2.8875	1.7761	8.3384	2.2948
D50	E-45	A180	34.368	0.053268	645.2	2.3009	0.00072848	3158.5	2.1192	1.137	4.4917	1.5891
D50	E-60	A0	36.924	0.11047	334.24	2.6227	0.0017476	1500.8	4.449	2.6727	19.799	3.5453
D50	E-60	A30	34.374	0.073352	468.62	2.5346	0.0011051	2293.4	2.9166	1.4233	8.5078	2.0958
D50	E-60	A45	31.501	0.070773	445.09	2.5133	0.0010406	2415.3	2.7698	1.2428	7.673	1.9307
D50	E-60	A60	29.935	0.079916	374.58	2.5858	0.0011986	2157.5	3.0999	1.7304	9.6112	2.3879
D50	E-60	A90	33.874	0.10694	316.77	2.8993	0.0018382	1577.3	4.2344	2.3891	17.936	3.2848
D50	E-60	A120	29.472	0.12605	233.82	2.642	0.001927	1371	4.8671	3.0722	23.695	3.9546
D50	E-60	A135	31.383	0.11831	265.27	2.4842	0.0017186	1445.5	4.618	2.6435	21.333	3.5744
D50	E-60	A150	35.069	0.081022	432.83	2.4326	0.0011762	2068.2	3.2333	1.7613	10.456	2.435
D50	E-60	A180	37.732	0.062962	599.28	2.469	0.00094174	2621.8	2.5521	1.4165	6.5146	1.9392
D52	E30	A-30	43.079	0.042022	1025.1	2.191	0.00057521	3809	1.7576	1.0832	3.0898	1.4059

Table L. Virtual Random, adults males stature. $c_0 = Int \quad c_1 = viewa \quad c_2 = stat.$

Dist	El	Az	Int	Int se	Int t-value	viewa	viewa se	viewa t-value	Stature	Stature se	Stature t-value	R se	CV RMSE
D35	E0	A0	37.064	0.26495	139.89	2.2054	0.0033506	658.21	0.53787	0.026431	20.35	1.7443	1.076
D35	E0	A30	28.085	0.20832	134.82	2.223	0.0027799	799.68	-0.023051	0.022438	-1.0273	1.4395	0.89841
D35	E0	A45	48.573	0.23459	207.06	2.5303	0.0032129	787.55	-2.3691	0.025686	-92.235	1.4614	0.79819
D35	E0	A90	5.6332	0.44783	12.579	3.4672	0.01062	326.49	-0.15794	0.055279	-2.8571	3.433	1.9445
D35	E0	A150	58.865	0.27999	210.24	2.8917	0.0041735	692.86	-3.9653	0.031449	-126.09	1.6584	0.92937
D35	E0	A-60	60.668	0.34194	177.42	2.9133	0.0050948	571.81	-3.7946	0.037809	-100.36	2.0027	1.1096
D35	E0	A-90	-7.5609	0.61353	-12.324	3.269	0.01501	217.79	1.2182	0.076561	15.912	4.9571	2.5097
D35	E0	A-135	47.666	0.22062	216.05	2.6121	0.0031327	833.81	-3.0033	0.025005	-120.11	1.3812	0.64379
D37	E35.5	A30	34.637	0.23015	150.49	2.2599	0.0030185	748.68	-0.85011	0.02504	-33.95	1.5364	0.93969
D37	E35.5	A60	101.45	0.58212	174.28	3.3484	0.008366	400.24	-7.6961	0.063581	-121.05	2.83	1.5738
D43	E0	A0	20.884	0.19706	105.98	2.1672	0.0026635	813.68	0.32929	0.021633	15.222	1.415	0.84746
D43	E0	A30	20.543	0.18586	110.53	2.1696	0.0025191	861.23	0.37067	0.020392	18.177	1.3376	0.82407
D43	E0	A45	39.043	0.21514	181.48	2.4586	0.003001	819.25	-1.8349	0.024056	-76.277	1.4055	0.79401
D43	E0	A60	48.608	0.29229	166.3	2.816	0.004457	631.81	-3.1242	0.033181	-94.155	1.816	1.0073
D43	E0	A90	15.88	0.39332	40.374	3.2997	0.0083547	394.95	0.7092	0.04359	16.27	2.8663	1.7054
D43	E0	A120	46.994	0.24797	189.51	2.8317	0.0038307	739.21	-3.3519	0.028665	-116.94	1.5558	0.85853
D43	E0	A135	36.021	0.20123	179	2.5487	0.0029545	862.64	-2.3486	0.023428	-100.25	1.3355	0.66071
D43	E0	A150	19.585	0.14833	132.03	2.2082	0.002056	1074	-0.045985	0.01673	-2.7487	1.0744	0.63024
D43	E0	A180	10.5	0.23387	44.896	2.1426	0.0033109	647.12	0.93143	0.026294	35.423	1.7737	1.0295
D43	E30	A0	8.6531	0.49242	17.572	2.1583	0.0071547	301.66	0.98505	0.056132	17.549	3.6958	1.761
D43	E30	A30	31.335	0.20667	151.62	2.2426	0.0027353	819.88	-0.65863	0.022639	-29.092	1.4044	0.86377
D43	E30	A45	62.093	0.43792	141.79	2.6715	0.0059504	448.96	-4.0413	0.048681	-83.016	2.534	1.1021
D43	E30	A60	88.114	0.45578	193.33	3.1728	0.0065523	484.22	-6.5352	0.050202	-130.18	2.3551	1.3718
D43	E30	A90	16.453	0.438	37.563	3.1806	0.0089515	355.31	0.15261	0.049958	3.0548	3.1699	1.9633
D43	E30	A120	59.378	0.26419	224.76	2.9036	0.0039441	736.19	-3.97	0.029605	-134.1	1.5621	0.90427
D43	E30	A135	60.135	0.28146	213.65	2.6059	0.0037584	693.35	-3.7334	0.031099	-120.05	1.6572	0.94797
D43	E30	A150	35.127	0.20299	173.05	2.3181	0.0027231	851.26	-1.2598	0.022493	-56.011	1.3532	0.7641
D43	E30	A180	-16.573	0.54633	-30.336	2.1085	0.0090943	231.85	2.7384	0.065612	41.737	4.6925	2.5513
D43	E45	A0	40.916	0.41249	99.194	2.2925	0.0053297	430.13	-1.166	0.044271	-26.338	2.6409	1.3659
D43	E45	A30	46.137	0.30612	150.72	2.3385	0.0039244	595.89	-1.6381	0.032743	-50.03	1.9234	1.1697
D43	E45	A45	74.139	0.51344	144.4	2.7372	0.0067715	404.23	-4.611	0.055444	-83.166	2.8033	1.3467
D43	E45	A60	138.66	0.87924	157.71	4.0151	0.013178	304.69	-11.526	0.095905	-120.18	3.6617	1.9916
D43	E45	A90	37.079	0.65072	56.982	3.1894	0.011999	265.81	-1.013	0.070976	-14.272	4.1513	2.4497
D43	E45	A120	70.735	0.35615	198.61	3.0788	0.0053555	574.87	-4.9127	0.039516	-124.32	1.9922	1.1086
D43	E45	A135	64.528	0.36272	177.9	2.7065	0.0049326	548.68	-3.8942	0.039579	-98.391	2.0852	1.1628
D43	E45	A150	45.23	0.22906	197.46	2.5117	0.0031655	793.47	-2.3452	0.025465	-92.095	1.4506	0.83158
D43	E45	A180	9.3361	0.47443	19.679	2.2806	0.0072496	314.58	0.68946	0.054748	12.593	3.5544	1.8437
D43	E60	A0	65.51	0.44569	146.98	2.4921	0.005561	448.14	-2.6277	0.04568	-57.523	2.5385	1.4954
D43	E60	A30	74.55	0.53839	138.47	2.5606	0.0066322	386.09	-3.4168	0.05501	-62.113	2.9292	1.6381
D43	E60	A45	104.45	0.89351	116.9	3.0728	0.011678	263.12	-6.5479	0.092342	-70.909	4.1898	2.1227
D43	E60	A60	195.26	2.1562	90.557	4.3388	0.029175	148.72	-14.288	0.21455	-66.596	6.7729	3.3613
D43	E60	A90	33.85	0.75593	44.78	2.8609	0.012762	224.17	1.1263	0.074798	15.058	4.8338	2.7348
D43	E60	A120	118.31	0.63195	187.22	3.6768	0.0093361	393.83	-8.8572	0.067524	-131.17	2.8741	1.502
D43	E60	A135	100.48	0.42597	235.89	3.2536	0.0059648	545.46	-7.4479	0.046218	-161.15	2.0972	1.111
D43	E60	A150	87.743	0.48989	179.11	2.8658	0.0063733	449.66	-5.6962	0.05222	-109.08	2.5302	1.3486
D43	E60	A180	53.099	0.50192	105.79	2.5586	0.0068244	374.92	-2.4976	0.054245	-46.044	3.0123	1.3605
D43	E90	A0	-41.278	0.36144	-114.2	3.5273	0.011655	302.65	7.073	0.036788	192.26	3.6845	2.0722
D43	E-30	A0	25.878	0.2154	120.14	2.2727	0.0029733	764.35	-0.60064	0.024209	-24.81	1.5052	0.84127
D43	E-30	A30	35.96	0.18056	199.16	2.315	0.0024082	961.3	0.10057	0.018543	5.4235	1.1995	0.70522
D43	E-30	A45	33.501	0.25927	129.21	2.4601	0.0037247	660.47	-1.0319	0.028649	-36.017	1.7385	1.008
D43	E-30	A60	35.729	0.33263	107.41	2.8233	0.0054275	520.17	-1.9453	0.03808	-51.084	2.1966	1.2625
D43	E-30	A90	3.2791	0.31917	10.274	2.7869	0.0061384	454.01	2.1043	0.034963	60.186	2.5067	1.3799
D43	E-30	A120	40.105	0.36944	108.56	2.8802	0.0060174	478.64	-2.7426	0.043008	-63.77	2.3817	1.5535
D43	E-30	A135	39.897	0.45044	88.574	2.639	0.0067398	391.56	-2.6893	0.052422	-51.3	2.89	1.6046
D43	E-30	A150	26.016	0.26148	99.495	2.2755	0.0036138	629.68	-0.62426	0.029419	-21.22	1.822	1.109
D43	E-30	A180	25.878	0.2154	120.14	2.2727	0.0029733	764.35	-0.60064	0.024209	-24.81	1.5052	0.8411
D43	E-45	A0	34.058	0.2415	141.03	2.4453	0.0034378	711.29	-0.86896	0.026381	-32.939	1.616	0.9215
D43	E-45	A30	32.968	0.25221	130.71	2.3987	0.0035424	677.15	-0.78166	0.027584	-28.337	1.6963	0.96324
D43	E-45	A45	42.499	0.27977	151.91	2.6117	0.0040771	640.56	-1.9144	0.030882	-61.99	1.7916	1.0076
D43	E-45	A60	58.179	0.36976	157.34	2.9365	0.0056203	522.47	-3.3651	0.04057	-82.944	2.1872	1.2442
D43	E-45	A90	-0.050499	0.34941	-0.14453	2.6743	0.0065825	406.27	2.7148	0.037613	72.176	2.7898	1.6203
D43	E-45	A120	56.807	0.41218	137.82	3.1958	0.0068667	465.41	-4.3675	0.04765	-91.657	2.4473	1.4948
D43	E-45	A135	43.415	0.66653	65.137	2.7012	0.010076	268.07	-2.731	0.076646	-35.631	4.1194	2.4489
D43	E-45	A150	31.637	0.43529	72.681	2.3856	0.0061433	388.33	-1.0745	0.048797	-22.019	2.913	1.8711
D43	E-45	A180	33.591	0.37257	90.16	2.3711	0.0051661	458.98	-1.2409	0.041652	-29.791	2.4805	1.4292
D43	E-60	A0	42.37	0.27971	151.48	2.7385	0.004277	640.28	-1.7117	0.030587	-55.96	1.7924	1.002
D43	E-60	A30	44.246	0.31255	141.56	2.7068	0.0046819	578.14	-1.8748	0.034146	-54.904	1.9812	1.0671
D43	E-60	A45	42.969	0.2325	184.81	2.7211	0.0035202	772.99	-1.8085	0.025461	-71.029	1.4886	0.81877
D43	E-60	A60	40.31	0.33395	120.71	2.7969	0.0052736	530.36	-1.4643	0.036464	-40.157	2.1555	1.2257
D43	E-60	A90	2.3808	0.46539	5.1157	2.4584	0.0079829	307.96	3.208	0.048027	66.795	3.6256	2.1689
D43	E-60	A120	40.31	0.33395	120.71	2.7969	0.0052736	530.36	-1.4643	0.036464	-40.157	2.1555	1.2261
D43	E-60	A135	54.49	0.59691	91.287	2.9566	0.0093312	316.85	-3.4749	0.067182	-51.724	3.5306	1.9281
D43	E-60	A150	42.088	0.60478	69.593	2.6365	0.0089708	293.9	-1.9057	0.067185	-28.365	3.7861	2.3773
D43	E-60	A180	47.75	0.51775	92.226	2.721	0.007688	353.93	-2.6335	0.05783	-45.538	3.1816	1.9753
D43	E-90	A0	-6.7263	0.5388	-12.484	2.8296	0.011292	250.58	4.1592	0.055306	75.204	4.3786	2.7508
D50	E0	A0	27.287	0.24279	112.39	2.1566	0.0031579	682.93	0.8444	0.02504	33.722	1.6822	1.019
D50	E30	A0	53.726	0.31474	170.7	2.2389	0.0037234	601.31	-0.80112	0.031091	-25.767	1.9063	1.0545
D50	E45	A0	38.273	0.23504	162.84	2.1336	0.0028569	746.8	0.18629	0.023754	7.8423	1.5402	0.89347
D50	E60	A0	73.167	0.47496	154.05	2.4415	0.0056091	435.28	-1.8312	0.04524	-40.477	2.6108	1.5771
D50	E90	A0	-29.386	0.37841	-77.657	3.5466	0.011365	312.08	7.0079	0.035882	195.3	3.581	2.0797
D50	E-30	A0	35.597	0.20956	169.87	2.3048	0.0027886	826.52	0.20377	0.021444	9.5024	1.3932	0.81978
D50	E-30	A45	-53.791	0.71986	-74.725	2.2715	0.017505	129.77	5.2292	0.097849	53.441	7.4825	4.0908
D50	E-30	A60	31.458	0.3495	90.01	2.7663	0.0057146	484.07	-2.0882	0.041205	-50.679	2.3558	1.4351
D50	E-30	A90	10.271	0.31404	32.707	2.8225	0.0058756	480.37	1.3369	0.03459	38.649	2.3734	1.2928
D50	E-30	A120	36.609	0.41633	87.934	2.8254	0.0067781	416.85	-2.8486	0.049622	-57.406	2.7218	1.8556
D50	E-30	A135	40.373	0.88405	45.668	3.0111	0.015234	197.65	-2.9696	0.10499	-28.284	5.3876	2.4369
D50	E-30	A180	32.477	0.21169	153.42	2.2781	0.0028294	805.14	-0.15152	0.022441	-6.752	1.4298	0.83439
D50	E-45	A0	40.793	0.26699	152.79	2.4511	0.0036825	665.6	-0.20888	0.027226	-7.6722	1.7253	1.0142
D50	E-45	A30	32.556	0.25734	126.51	2.3814	0.0035962	662.2	-0.5704	0.027896	-20.447	1.734	1.0054
D50	E-45	A45	33.265	0.36349	91.517	2.6077	0.0055516	469.73	-2.0004	0.042279	-47.316	2.4255	1.4666
D50	E-45	A60	-24.881	0.83361	-29.847	2.923	0.021027	139.01	1.8207	0.11517	15.809	7.1218	3.8973
D50	E-45	A90	10.577	1.7497	6.0449	42.504	0.53201	79.892	3.1671	0.18197	17.405	9.9719	5.3848
D50	E-45	A120	75.144	0.87689	85.694	3.8308	0.016201	236.45	-6.1927	0.10124	-61.167	4.6116	2.4609
D50	E-45	A135	27.127	0.70014	38.745	2.6539	0.011354	233.75	-1.9898	0.084747	-23.479	4.6588	3.0178
D50	E-45	A150	31.447	0.43454	72.369	2.3601	0.0060732	388.61	-0.88891	0.048296	-18.406	2.911	1.8757
D50	E-45	A180	43.426	0.30463	142.55	2.3972	0.0040575	590.81	-0.87144	0.03176	-27.439	1.9396	1.1017
D50	E-60	A0	48.505	0.33835	143.36	2.7619	0.0050656	545.22	-0.92877	0.034514	-26.91	2.0981	1.2066
D50	E-60	A30	50.926	0.27945	182.24	2.726	0.0040781	668.43	-1.5245	0.029027	-52.521	1.7181	0.90288
D50	E-60	A45	46.17	0.23734	194.53	2.7034	0.0035141	769.3	-1.4697	0.025154	-58.428	1.4957	0.8523
D50	E-60	A60	43.564	0.33883	128.57	2.7862	0.0052442	531.3	-1.4694	0.036409	-40.358	2.1518	1.2376
D50	E-60	A90	8.4505	0.47268	17.878	2.4492	0.0077966	314.14	2.7555	0.04847	56.85	3.559	2.058
D50	E-60	A120	67.603	0.48035	140.74	3.292	0.0078448	419.64	-4.5046	0.053161	-84.735	2.7044	1.6609
D50	E-60	A135	56.737	0.58033	97.766	2.9069	0.0088207	329.56	-3.0277	0.063267	-47.856	3.4029	1.781
D50	E-60	A150	51.586	0.50155	102.85	2.6603	0.0071426	372.45	-1.7933	0.052756	-33.993	3.0313	1.6625
D50	E-60	A180	54.841	0.37866	144.83	2.71	0.0053973	502.1	-1.7567	0.039082	-44.948	2.2736	1.2851
D52	E30	A-30	44.65	0.23749	188.01	2.1865	0.0028654	763.08	-0.041428	0.023537	-1.7601	1.5077	0.92304

Table M. Virtual Random dataset,adults females stature, $c_0 = Int \quad c_1 = viewa \quad c_2 = stat$.

Dist	El	Az	Int	Int se	Int t-value	viewa	viewa se	viewa t-value	Stature	Stature se	Stature t-value	R se	CV RMSE
D35	E0	A0	25.478	0.22498	113.25	2.0837	0.0032053	650.08	1.4793	0.023338	63.383	1.7125	1.0915
D35	E0	A30	28.699	0.13687	209.68	2.2766	0.0020879	1090.4	-0.29455	0.015491	-19.015	1.0265	0.62292
D35	E0	A45	45.967	0.28664	160.36	2.524	0.0044069	572.75	-2.1763	0.032671	-66.613	1.9391	1.1123
D35	E0	A90	5.6406	0.32937	17.125	3.3276	0.0085008	391.45	0.24939	0.04175	5.9735	2.8035	1.5747
D35	E0	A150	44.934	0.39598	113.48	2.6494	0.0064392	411.45	-2.2236	0.045569	-48.797	2.6728	1.4154
D35	E0	A-60	52.004	0.48001	108.34	2.7941	0.0079339	352.17	-2.7965	0.05481	-51.022	3.1	1.7778
D35	E0	A-90	-2.4299	0.35786	-6.7899	3.1311	0.00918	341.08	1.3021	0.044912	28.992	3.1951	1.6616
D35	E0	A-135	38.726	0.25815	150.01	2.4607	0.0040259	611.22	-1.8623	0.030116	-61.84	1.8194	1.0335
D37	E35.5	A30	38.674	0.24938	155.08	2.3333	0.0036883	632.62	-1.3612	0.028326	-48.053	1.759	1.0093
D37	E35.5	A60	72.675	0.959	75.782	3.0151	0.015515	194.34	-4.6534	0.10846	-42.905	5.299	3.1937
D43	E0	A0	12.53	0.18807	66.626	2.0657	0.0028712	719.45	1.1691	0.021505	54.365	1.5497	0.95611
D43	E0	A30	22.322	0.12299	181.5	2.2361	0.001913	1168.9	-0.017511	0.01422	-1.2314	0.9579	0.58553
D43	E0	A45	36.902	0.25614	144.07	2.4592	0.0040334	609.71	-1.6733	0.029888	-55.986	1.8238	1.0261
D43	E0	A60	41.668	0.42612	97.785	2.7111	0.0072258	375.2	-2.2465	0.050021	-44.911	2.9192	1.6717
D43	E0	A90	11.07	0.26557	41.685	3.1441	0.0062403	503.83	1.3891	0.030273	45.886	2.1976	1.2188
D43	E0	A120	35.423	0.34469	102.77	2.6082	0.0058153	448.5	-1.7644	0.040812	-43.231	2.4599	1.2908
D43	E0	A135	29.451	0.21452	137.29	2.4093	0.0034518	697.99	-1.3723	0.025691	-53.414	1.5967	0.89161
D43	E0	A150	18.637	0.16684	111.71	2.1904	0.0026006	842.26	0.069114	0.019633	3.5203	1.3262	0.76307
D43	E0	A180	17.234	0.26164	65.87	2.2325	0.0042009	531.43	0.13579	0.03098	4.3832	2.0863	1.2006
D43	E30	A0	21.538	0.35461	60.736	2.1292	0.0053036	401.47	0.31959	0.040542	7.8831	2.7365	1.5182
D43	E30	A30	32.613	0.20554	158.67	2.2861	0.0030808	742.04	-0.89014	0.023536	-37.821	1.5032	0.87935
D43	E30	A45	58.59	0.48927	119.75	2.7059	0.0075635	357.76	-3.8915	0.05695	-68.332	3.0541	1.6671
D43	E30	A60	66.864	0.82219	81.324	2.9361	0.013304	220.7	-4.2707	0.093858	-45.501	4.7514	2.7617
D43	E30	A90	2.0601	0.26318	7.8278	2.7782	0.0057885	479.95	2.2411	0.030071	74.527	2.3037	1.2653
D43	E30	A120	43.114	0.30484	141.43	2.6131	0.0049302	530.02	-1.9694	0.034921	-56.397	2.0917	1.2561
D43	E30	A135	44.807	0.19969	224.38	2.4536	0.003	817.86	-2.2373	0.022958	-97.45	1.3654	0.80817
D43	E30	A150	31.595	0.16722	188.95	2.306	0.0025417	907.25	-0.95101	0.019317	-49.231	1.2321	0.70819
D43	E30	A180	6.2008	0.40711	15.232	2.28	0.0071996	316.68	0.7535	0.050029	15.061	3.4255	1.8235
D43	E45	A0	39.203	0.38614	101.53	2.2461	0.0054956	408.7	-0.91687	0.04276	-21.442	2.6901	1.5744
D43	E45	A30	54.013	0.38878	138.93	2.4737	0.0056186	440.27	-2.5716	0.043362	-59.305	2.5043	1.4175
D43	E45	A45	70.103	0.88111	79.563	2.731	0.013059	209.13	-4.261	0.098981	-43.049	4.9784	2.823
D43	E45	A60	67.984	1.5576	43.647	2.9359	0.025538	114.96	-3.4507	0.17233	-20.024	7.8732	5.3322
D43	E45	A90	14.278	0.43452	32.86	2.6461	0.0084756	312.2	1.8911	0.04723	40.04	3.4713	1.9078
D43	E45	A120	48.911	0.4358	112.23	2.6676	0.006987	381.79	-2.1404	0.048888	-43.782	2.8711	1.8245
D43	E45	A135	55.261	0.29447	187.66	2.618	0.004468	585.93	-3.0225	0.033346	-90.639	1.8964	1.1753
D43	E45	A150	46.884	0.2243	209.03	2.5586	0.0034753	736.23	-2.5129	0.025867	-97.147	1.5149	0.87885
D43	E45	A180	32.238	0.40676	79.256	2.5114	0.0067413	372.54	-1.4955	0.048413	-30.892	2.939	1.5112
D43	E60	A0	59.84	0.73221	81.726	2.5388	0.010606	239.36	-2.4742	0.079216	-31.234	4.4234	2.573
D43	E60	A30	78.499	0.98963	79.321	2.6712	0.013784	193.8	-3.9124	0.105	-37.261	5.3114	2.9577
D43	E60	A45	57.602	1.8208	31.636	2.4035	0.026118	92.027	-1.2635	0.19132	-6.6037	8.993	5.7792
D43	E60	A60	47.126	2.7734	16.992	2.4181	0.043731	55.294	0.34617	0.28739	1.2045	11.131	6.9307
D43	E60	A90	15.641	0.50436	31.012	2.5784	0.0095346	270.43	2.7858	0.051304	54.3	3.9629	2.061
D43	E60	A120	55.428	0.72512	76.44	2.6607	0.01127	236.08	-1.7125	0.077158	-22.194	4.4779	2.9123
D43	E60	A135	73.213	0.61286	119.46	2.8148	0.0091671	307.06	-4.1044	0.067009	-61.252	3.5254	2.3722
D43	E60	A150	86.22	0.46795	184.25	2.9102	0.0067847	428.94	-5.5286	0.051259	-107.86	2.568	1.4662
D43	E60	A180	75.548	0.55581	135.93	2.8477	0.0083069	342.81	-4.6911	0.061716	-76.012	3.1799	1.7257
D43	E90	A0	-52.517	0.6069	-86.533	2.4315	0.01868	130.16	9.3067	0.058571	158.9	7.2379	4.2748
D43	E-30	A0	30.631	0.26064	117.52	2.4282	0.0042014	577.96	-1.4753	0.031195	-47.294	1.922	1.1143
D43	E-30	A30	32.801	0.1298	252.7	2.3444	0.0019912	1177.4	0.049848	0.014063	3.5447	0.95105	0.53141
D43	E-30	A45	30.854	0.2992	103.12	2.4471	0.0048579	503.74	-0.87974	0.034635	-25.4	2.198	1.2573
D43	E-30	A60	27.922	0.4676	59.713	2.6961	0.0085519	315.27	-0.96554	0.055542	-17.384	3.4398	2.0429
D43	E-30	A90	1.0163	0.17247	5.8929	2.7186	0.0037261	729.63	2.369	0.019627	120.7	1.5284	0.8589
D43	E-30	A120	27.293	0.60529	45.092	2.5725	0.01066	241.33	-0.85104	0.07201	-11.818	4.3913	2.8812
D43	E-30	A135	29.949	0.54876	54.576	2.4666	0.009097	271.14	-1.4093	0.066136	-21.309	3.9535	2.5304
D43	E-30	A150	26.019	0.32103	81.047	2.3127	0.0050712	456.04	-0.77906	0.038036	-20.482	2.4206	1.3632
D43	E-30	A180	30.631	0.26064	117.52	2.4282	0.0042014	577.96	-1.4753	0.031195	-47.294	1.922	1.1143
D43	E-45	A0	18.818	0.28408	66.24	2.229	0.0045128	493.92	0.53626	0.032544	16.478	2.2404	1.2043
D43	E-45	A30	32.545	0.34729	93.712	2.4648	0.0056301	437.78	-1.0779	0.040283	-26.758	2.518	1.2682
D43	E-45	A45	37.233	0.52744	70.592	2.5609	0.0086893	294.72	-1.4479	0.060992	-23.74	3.662	2.1033
D43	E-45	A60	32.288	0.62512	51.651	2.635	0.010948	240.69	-0.72535	0.071692	-10.118	4.4017	2.6409
D43	E-45	A90	-3.1517	0.20487	-15.384	2.6408	0.0044218	597.22	2.9481	0.023045	127.93	1.8613	1.0895
D43	E-45	A120	31.893	0.69887	45.635	2.75	0.012843	214.13	-1.1855	0.08262	-14.349	4.878	3.3321
D43	E-45	A135	31.828	0.63264	50.311	2.4983	0.010537	237.1	-1.2175	0.074793	-16.279	4.4609	2.9351
D43	E-45	A150	33.153	0.3812	86.971	2.4427	0.0061081	399.92	-1.4124	0.044903	-31.454	2.7467	1.709
D43	E-45	A180	39.427	0.3284	120.06	2.5758	0.0053466	481.76	-2.3561	0.039196	-60.111	2.2953	1.3786
D43	E-60	A0	22.059	0.59876	36.841	2.4078	0.010194	236.2	0.27479	0.06893	3.9865	4.476	2.3507
D43	E-60	A30	35.343	0.56214	62.872	2.6474	0.0096909	273.19	-1.2435	0.065051	-19.116	3.9264	1.9388
D43	E-60	A45	36.532	0.51955	70.314	2.6886	0.0090203	298.06	-1.3054	0.059847	-21.812	3.6241	1.8787
D43	E-60	A60	25.024	0.50154	49.893	2.5963	0.0090008	288.45	0.15563	0.056909	2.7347	3.7355	2.255
D43	E-60	A90	1.704	0.26104	6.5276	2.4904	0.0051583	482.8	3.204	0.027988	114.48	2.2905	1.4227
D43	E-60	A120	25.024	0.50154	49.893	2.5963	0.0090008	288.45	0.15563	0.056909	2.7347	3.7355	2.2548
D43	E-60	A135	37.009	0.81608	45.349	2.6792	0.014235	188.22	-1.3213	0.094611	-13.966	5.4429	3.7855
D43	E-60	A150	44.98	0.45456	98.952	2.7426	0.0076598	358.05	-2.3972	0.052822	-45.383	3.0518	1.9859
D43	E-60	A180	41.419	0.33644	123.11	2.6439	0.0055608	475.45	-2.0592	0.039107	-52.656	2.3248	1.3107
D43	E-90	A0	16.44	0.41019	40.079	3.1933	0.0095148	335.62	1.9641	0.043741	44.902	3.244	1.9861
D50	E0	A0	17.524	0.21018	83.376	2.0445	0.0030812	663.54	1.691	0.022559	74.96	1.6783	1.0587
D50	E30	A0	47.373	0.39197	120.86	2.2391	0.0053148	421.3	-0.52511	0.040581	-12.94	2.6128	1.5583
D50	E45	A0	34.913	0.27202	128.35	2.1004	0.0037011	567.52	0.4018	0.028559	14.069	1.9566	1.2079
D50	E60	A0	70.348	0.72996	96.371	2.5326	0.0099878	253.57	-2.0438	0.073138	-27.944	4.2009	2.474
D50	E90	A0	-39.214	0.63806	-61.458	2.6078	0.018788	138.8	8.7508	0.058711	149.05	6.9118	4.048
D50	E-30	A0	20.777	0.16905	122.91	2.1194	0.0025169	842.06	1.4406	0.018066	79.741	1.3265	0.80949
D50	E-30	A45	-28.18	0.66872	-42.14	2.462	0.016965	145.12	3.0039	0.09344	32.148	6.6883	3.4689
D50	E-30	A60	26.64	0.45549	58.487	2.6906	0.0083743	321.3	-1.399	0.055816	-25.065	3.3795	1.9625
D50	E-30	A90	5.031	0.19138	26.288	2.6941	0.0039951	674.35	1.985	0.021779	91.141	1.6518	0.95474
D50	E-30	A120	26.144	0.61143	42.759	2.5619	0.010803	237.15	-1.2921	0.075083	-17.209	4.46	2.8509
D50	E-30	A135	46.855	0.87359	53.635	2.7981	0.015049	185.93	-2.3905	0.10139	-23.577	5.4985	3.4994
D50	E-30	A180	35.55	0.27096	131.2	2.3583	0.0041242	571.81	-0.60658	0.030053	-20.183	1.9422	1.141
D50	E-45	A0	26.109	0.28948	90.195	2.2711	0.0044846	506.43	0.88755	0.031072	28.564	2.1866	1.186
D50	E-45	A30	31.769	0.31146	102	2.4421	0.0050212	486.36	-0.80875	0.035729	-22.636	2.2742	1.147
D50	E-45	A45	30.281	0.51575	58.712	2.5576	0.008837	289.42	-1.602	0.062625	-25.58	3.724	2.1932
D50	E-45	A60	-21.449	0.80311	-26.707	2.9206	0.023333	125.17	1.6563	0.11845	13.983	7.4376	4.1814
D50	E-45	A90	-8.3569	1.0409	-8.0283	37.659	0.35933	104.8	4.3069	0.11649	36.973	8.3448	5.1173
D50	E-45	A120	54.177	1.472	36.804	2.956	0.026216	112.75	-1.7581	0.1609	-10.927	7.9725	5.3291
D50	E-45	A135	16.453	0.60564	27.167	2.474	0.010986	225.2	-0.62223	0.076147	-8.1715	4.6684	3.0884
D50	E-45	A150	30.681	0.36849	83.26	2.3814	0.0058381	407.91	-0.93488	0.042886	-21.799	2.6951	1.6945
D50	E-45	A180	44.742	0.28461	157.21	2.4864	0.0043392	573	-1.2692	0.031114	-40.79	1.9383	1.0923
D50	E-60	A0	34.649	0.52914	65.481	2.5242	0.0087234	289.37	0.26771	0.056354	4.7505	3.7246	1.913
D50	E-60	A30	42.891	0.46032	93.178	2.6927	0.007706	349.43	-1.0738	0.05043	-21.293	3.123	1.5175
D50	E-60	A45	38.503	0.47933	80.327	2.6426	0.0080776	327.15	-0.86859	0.053243	-16.314	3.3228	1.7323
D50	E-60	A60	29.432	0.50532	58.245	2.5924	0.0088176	294	0.0089844	0.056323	0.15952	3.6703	2.2153
D50	E-60	A90	4.8713	0.26942	18.08	2.4453	0.0051228	477.33	3.0214	0.028673	105.38	2.316	1.4161
D50	E-60	A120	43.352	0.75117	57.713	2.9759	0.013984	212.81	-1.7721	0.085817	-20.649	4.904	3.5942
D50	E-60	A135	42.035	0.75002	56.045	2.6758	0.01265	211.53	-1.2019	0.083704	-14.359	4.9297	3.4422
D50	E-60	A150	47.574	0.41092	115.77	2.6806	0.0066653	402.17	-1.5145	0.044899	-33.731	2.7319	1.7142
D50	E-60	A180	47.693	0.31113	153.29	2.5983	0.0048798	532.45	-1.0436	0.033069	-31.558	2.0824	1.199
D52	E30	A-30	45.779	0.26891	170.24	2.2383	0.0036691	610.05	-0.35128	0.027766	-12.651	1.8229	1.1032

Table N. Virtual Random dataset,normal stature.
$c_0 = Int \quad c_1 = viewa.$

Dist	El	Az	Int	Int se	Int Tvalue	viewa	viewa se	viewa Tvalue	R se	CV RMSE	CV MSPE	CV MAPE
D35	E0	A0	39.481	0.083282	474.07	2.2937	0.0015983	1435.1	2.2285	1.3794	4.9674	1.7891
D35	E0	A30	25.059	0.056954	439.99	2.2562	0.00096201	2345.3	1.3662	0.86594	1.8668	1.1039
D35	E0	A45	24.349	0.09019	269.97	2.2913	0.0015392	1488.6	2.1488	1.1297	4.6187	1.5914
D35	E0	A90	9.6249	0.1181	81.497	3.3222	0.0026391	1258.8	2.5382	1.5372	6.4442	2.0233
D35	E0	A150	21.789	0.11766	185.19	2.4191	0.0020814	1162.3	2.7473	1.578	7.548	2.1371
D35	E0	A-60	24.052	0.12483	192.68	2.4705	0.0022923	1077.7	2.9605	1.6791	8.7665	2.2911
D35	E0	A-90	5.2776	0.15393	34.286	3.4272	0.0034498	993.47	3.2086	1.8909	10.297	2.5144
D35	E0	A-135	21.254	0.087912	241.76	2.2536	0.0014431	1561.6	2.0489	1.0375	4.1992	1.4858
D37	E35.5	A30	23.868	0.088304	270.29	2.2064	0.0014461	1525.7	2.0968	1.2166	4.397	1.6068
D37	E35.5	A60	25.653	0.20549	124.83	2.4711	0.0038206	646.79	4.8872	2.6857	23.889	3.7442
D43	E0	A0	23.817	0.068992	345.22	2.1971	0.0011246	1953.6	1.6393	1.006	2.6878	1.3132
D43	E0	A30	21.337	0.048531	439.67	2.2458	0.00079433	2827.3	1.1336	0.706	1.2854	0.91025
D43	E0	A45	20.462	0.078644	260.18	2.2804	0.001299	1755.5	1.8235	0.88128	3.3264	1.2997
D43	E0	A60	20.295	0.11333	179.08	2.4403	0.0020009	1219.6	2.6192	1.4318	6.8617	1.9826
D43	E0	A90	23.408	0.10041	233.12	3.3914	0.0025192	1346.2	2.3746	1.3596	5.6396	1.8359
D43	E0	A120	17.048	0.10638	160.25	2.4273	0.0018262	1329.1	2.405	1.2876	5.785	1.8007
D43	E0	A135	17.039	0.073106	233.07	2.2539	0.0011652	1934.4	1.6555	0.76905	2.7415	1.1435
D43	E0	A150	18.831	0.050576	372.33	2.2049	0.00079843	2761.5	1.1606	0.6561	1.3473	0.89021
D43	E0	A180	18.992	0.074569	254.69	2.2414	0.0011981	1870.8	1.7116	0.9902	2.93	1.3347
D43	E30	A0	26.191	0.06593	397.25	2.1373	0.0010638	2009.2	1.5941	0.891	2.5415	1.2227
D43	E30	A30	22.659	0.076669	295.54	2.206	0.0012444	1772.7	1.8059	1.087	3.2618	1.414
D43	E30	A45	21.411	0.15083	141.96	2.2707	0.0024981	909	3.5025	1.6072	12.271	2.4026
D43	E30	A60	24.452	0.18595	131.49	2.4347	0.0033761	721.16	4.3957	2.4058	19.325	3.355
D43	E30	A90	24.855	0.11253	220.87	3.0723	0.0025849	1188.6	2.687	1.4799	7.2209	2.0552
D43	E30	A120	21.85	0.097533	224.03	2.4317	0.001735	1401.6	2.2815	1.2777	5.2058	1.7527
D43	E30	A135	21.854	0.090879	240.48	2.2422	0.0014907	1504.2	2.1267	1.1791	4.5241	1.6226
D43	E30	A150	20.523	0.059165	346.89	2.2291	0.00095562	2332.6	1.3736	0.73252	1.8872	1.0298
D43	E30	A180	20.899	0.076183	274.33	2.2311	0.001235	1806.6	1.7721	0.99134	3.1412	1.3603
D43	E45	A0	27.967	0.1002	279.11	2.1878	0.001677	1304.6	2.4498	1.4031	6.0034	1.8867
D43	E45	A30	27.115	0.12445	217.88	2.2107	0.0020916	1057	3.018	1.6745	9.1097	2.2615
D43	E45	A45	26.586	0.19908	133.54	2.277	0.0034341	663.06	4.7706	2.2343	22.76	3.3196
D43	E45	A60	30.071	0.27393	109.78	2.5398	0.0054134	469.17	6.6514	3.8978	44.25	5.2394
D43	E45	A90	31.407	0.12718	246.96	2.9497	0.0029456	1001.4	3.1836	1.7454	10.137	2.4362
D43	E45	A120	26.27	0.11365	231.15	2.4488	0.0021024	1164.8	2.7414	1.4659	7.5168	2.0508
D43	E45	A135	24.178	0.10312	234.48	2.3166	0.0017771	1303.6	2.4517	1.2732	6.0124	1.815
D43	E45	A150	23.187	0.094403	245.62	2.2604	0.0015761	1434.2	2.2299	1.2136	4.9729	1.6743
D43	E45	A180	22.585	0.084711	266.61	2.2587	0.001407	1605.3	1.9934	0.99632	3.9746	1.4213
D43	E60	A0	32.111	0.1629	197.12	2.3087	0.0029698	777.39	4.0844	2.3742	16.685	3.133
D43	E60	A30	34.997	0.20185	173.38	2.2755	0.0037104	613.27	5.146	2.6942	26.488	3.7435
D43	E60	A45	39.167	0.27994	139.91	2.3188	0.0054264	427.31	7.2632	3.9413	52.764	5.4389
D43	E60	A60	40.347	0.35521	113.59	2.6457	0.0079411	333.17	9.1295	5.5097	83.362	7.2481
D43	E60	A90	38.993	0.15506	251.48	3.1603	0.0040855	773.53	4.1043	2.2678	16.85	3.1376
D43	E60	A120	37.016	0.16565	223.45	2.4655	0.0033517	735.6	4.3113	2.4948	18.591	3.3587
D43	E60	A135	34.511	0.15343	224.93	2.2886	0.0028249	810.18	3.9222	2.1221	15.387	2.9495
D43	E60	A150	33.042	0.15762	209.64	2.2331	0.0027993	797.71	3.9823	2.1459	15.862	2.9391
D43	E60	A180	30.063	0.15644	192.17	2.2757	0.0027675	822.29	3.8655	1.9194	14.945	2.7313
D43	E90	A0	49.785	0.33565	148.32	4.4041	0.013541	325.24	9.329	4.4822	87.048	6.8537
D43	E-30	A0	17.495	0.072059	242.79	2.2521	0.0011512	1956.3	1.637	0.8716	2.6802	1.2163
D43	E-30	A30	31.499	0.054611	576.79	2.4023	0.0010307	2330.9	1.3746	0.83487	1.8901	1.0952
D43	E-30	A45	20.806	0.07532	276.23	2.3822	0.0013028	1828.6	1.751	0.97419	3.0665	1.3341
D43	E-30	A60	19.993	0.099989	199.95	2.5493	0.0018402	1385.3	2.3081	1.2593	5.3282	1.7466
D43	E-30	A90	23.203	0.099311	233.64	3.0913	0.0022678	1363.1	2.3453	1.1821	5.5025	1.7213
D43	E-30	A120	14.265	0.1552	91.911	2.5529	0.0027495	928.49	3.4301	1.8832	11.767	2.6237
D43	E-30	A135	14.454	0.14809	97.605	2.346	0.002414	971.82	3.2792	1.6314	10.754	2.3718
D43	E-30	A150	17.199	0.092536	185.86	2.2495	0.0014737	1526.4	2.0958	1.0942	4.3936	1.5354
D43	E-30	A180	17.495	0.072059	242.79	2.2521	0.0011512	1956.3	1.637	0.87158	2.6803	1.2163
D43	E-45	A0	23.748	0.1281	185.38	2.3367	0.0022201	1052.5	3.0308	2.0244	9.1876	2.5347
D43	E-45	A30	23.064	0.083493	276.24	2.3381	0.0014405	1623.1	1.9716	1.0195	3.8886	1.4387
D43	E-45	A45	23.643	0.11088	213.24	2.393	0.0019663	1217	2.6247	1.2238	6.8904	1.8544
D43	E-45	A60	25.07	0.12716	197.16	2.5442	0.0024227	1050.2	3.0373	1.6204	9.2277	2.3016
D43	E-45	A90	25.161	0.12061	208.62	3.0864	0.0027894	1106.5	2.8844	1.5461	8.322	2.1781
D43	E-45	A120	16.681	0.17886	93.262	2.648	0.0033421	792.33	4.0089	2.2802	16.074	3.1163
D43	E-45	A135	16.939	0.17528	96.642	2.4062	0.0029813	807.1	3.9369	2.1183	15.503	2.9634
D43	E-45	A150	17.936	0.119	150.72	2.3171	0.0019623	1180.8	2.7045	1.471	7.3155	2.036
D43	E-45	A180	17.986	0.10124	177.65	2.2979	0.001656	1387.6	2.3043	1.2078	5.3107	1.7117
D43	E-60	A0	24.149	0.17292	139.66	2.5076	0.0032261	777.29	4.0849	2.3488	16.69	3.2082
D43	E-60	A30	23.366	0.12049	193.93	2.513	0.0022395	1122.1	2.8446	1.2984	8.0938	1.9814
D43	E-60	A45	23.735	0.10777	220.23	2.5299	0.002022	1251.2	2.5535	1.2278	6.5228	1.8137
D43	E-60	A60	25.395	0.11494	220.95	2.6261	0.0022656	1159.1	2.7546	1.6032	7.5891	2.1522
D43	E-60	A90	33.412	0.13744	243.1	2.931	0.0032127	912.31	3.49	2.2569	12.184	2.8599
D43	E-60	A120	25.395	0.11494	220.95	2.6261	0.0022656	1159.1	2.7546	1.6033	7.5895	2.1523
D43	E-60	A135	21.105	0.19311	109.29	2.552	0.0035873	711.39	4.4546	2.4085	19.848	3.3831
D43	E-60	A150	21.202	0.14211	149.19	2.463	0.002549	966.26	3.2978	1.8163	10.878	2.4987
D43	E-60	A180	20.048	0.12536	159.92	2.446	0.0022148	1104.4	2.8898	1.5525	8.3525	2.1759
D43	E-90	A0	39.033	0.14312	272.73	3.4314	0.0040956	837.83	3.7951	2.2359	14.406	2.9749
D50	E0	A0	33.481	0.082859	404.07	2.2666	0.0014982	1512.8	2.1146	1.29	4.4725	1.6877
D50	E30	A0	38.837	0.093055	417.36	2.249	0.001742	1291	2.4754	1.4612	6.1284	1.9414
D50	E45	A0	35.977	0.070049	513.6	2.2075	0.0012579	1754.9	1.8242	1.0749	3.3281	1.4348
D50	E60	A0	45.589	0.13989	325.9	2.3651	0.0029137	811.72	3.9149	2.3512	15.329	3.0653
D50	E90	A0	60.381	0.28038	215.35	4.5758	0.012959	353.09	8.6625	4.3024	75.054	6.4275
D50	E-30	A0	33.289	0.10439	318.88	2.3623	0.0019645	1202.5	2.6562	1.8612	7.0569	2.2635
D50	E-30	A45	12.452	0.084961	146.56	2.6013	0.0015147	1717.3	1.8639	1.0575	3.4749	1.4292
D50	E-30	A60	16.569	0.10985	150.82	2.4624	0.0019068	1291.4	2.4747	1.3582	6.1251	1.872
D50	E-30	A90	22.816	0.088021	259.21	3.012	0.0019529	1542.3	2.0744	1.0081	4.3034	1.4991
D50	E-30	A120	8.4094	0.17507	48.035	2.5126	0.0029357	855.88	3.7164	2.0728	13.814	2.8574
D50	E-30	A135	20.102	0.2178	92.294	2.5419	0.0040021	635.15	4.9742	2.7319	24.749	3.7385
D50	E-30	A180	28.515	0.062395	457.01	2.3037	0.001104	2086.7	1.535	0.84895	2.3568	1.1677
D50	E-45	A0	33.819	0.1331	254.09	2.458	0.0026174	939.12	3.3918	2.3346	11.506	2.8821
D50	E-45	A30	24.556	0.076128	322.56	2.3484	0.0013336	1761	1.8179	0.96611	3.3056	1.3417
D50	E-45	A45	17.247	0.12324	139.95	2.3451	0.0020469	1145.7	2.7866	1.3648	7.7671	1.9991
D50	E-45	A60	12.995	0.16392	79.275	2.7787	0.0031339	886.66	3.5894	1.8311	12.886	2.6584
D50	E-45	A90	29.561	0.47297	62.502	52.029	0.19117	272.17	10.905	6.813	118.95	8.7838
D50	E-45	A120	30.81	0.32205	95.666	2.7374	0.0069019	396.62	7.7853	4.5662	60.623	6.0845
D50	E-45	A135	6.3271	0.20562	30.77	2.4653	0.003338	738.57	4.2944	2.4949	18.445	3.3449
D50	E-45	A150	19.341	0.11361	170.23	2.316	0.001891	1224.7	2.6083	1.456	6.8044	1.9873
D50	E-45	A180	30.841	0.067945	453.9	2.3642	0.0012556	1882.8	1.7007	0.88657	2.8927	1.2573
D50	E-60	A0	35.566	0.14113	252.02	2.655	0.0030392	873.58	3.6423	2.1117	13.268	2.8532
D50	E-60	A30	31.378	0.09693	323.72	2.5951	0.0019745	1314.3	2.4319	1.2094	5.9157	1.7635

Table O. Virtual Random dataset,big stature. $c_0 = Int \quad c_1 = viewa$.

Dist	El	Az	Int	Int se	Int Tvalue	viewa	viewa se	viewa Tvalue	R se	CV RMSE	CV MSPE	CV MAPE
D35	E0	A0	44.316	0.21032	210.71	2.2468	0.002131	1054.3	2.4073	1.4863	5.7986	1.9351
D35	E0	A30	25.874	0.12889	200.74	2.2389	0.0012027	1861.6	1.3653	0.85557	1.8649	1.1055
D35	E0	A45	21.84	0.2073	105.36	2.2886	0.001945	1176.7	2.1578	1.0388	4.6583	1.5429
D35	E0	A90	-5.7414	0.37983	-15.116	3.5623	0.0049892	714	3.5461	1.9767	12.586	2.7169
D35	E0	A150	14.851	0.32257	46.04	2.4571	0.00316	777.56	3.2585	1.7961	10.626	2.4663
D35	E0	A-60	20.93	0.35392	59.139	2.4685	0.0035688	691.7	3.6593	2.2321	13.392	2.918
D35	E0	A-90	-18.002	0.58745	-30.645	3.7354	0.0077451	482.29	5.2219	2.5639	27.284	3.7978
D35	E0	A-135	13.628	0.23742	57.402	2.3114	0.0021774	1061.6	2.3909	1.1213	5.7245	1.6671
D37	E35.5	A30	23.065	0.13882	166.15	2.1934	0.0012545	1748.4	1.4535	0.88614	2.1133	1.1526
D37	E35.5	A60	21.894	0.57773	37.896	2.4468	0.0057981	422.01	5.9501	3.2262	35.429	4.5022
D43	E0	A0	26.072	0.16964	153.69	2.1874	0.0015477	1413.3	1.7975	1.0835	3.2324	1.4303
D43	E0	A30	23.581	0.13181	178.9	2.2176	0.0012069	1837.5	1.3832	0.83726	1.9142	1.0991
D43	E0	A45	19.558	0.19734	99.109	2.2664	0.0018168	1247.5	2.0357	1.0398	4.148	1.4968
D43	E0	A60	17.046	0.32115	53.078	2.4496	0.0031639	774.25	3.2723	1.9738	10.714	2.5964
D43	E0	A90	23.51	0.28123	83.597	3.4138	0.0039628	861.45	2.9433	1.7287	8.6663	2.3065
D43	E0	A120	12.518	0.28941	43.252	2.4461	0.0027967	874.64	2.8991	1.5368	8.4126	2.1637
D43	E0	A135	10.249	0.21749	47.124	2.3092	0.0019666	1174.2	2.1624	1.0966	4.6813	1.5739
D43	E0	A150	19.096	0.118	161.83	2.2032	0.0010541	2090.2	1.2161	0.74336	1.4799	0.97199
D43	E0	A180	21.026	0.23152	90.817	2.2333	0.0021128	1057	2.4012	1.4792	5.7685	1.9262
D43	E30	A0	-2.0884	0.48158	-4.3365	2.4349	0.0043824	555.6	4.5438	2.4553	20.664	3.4164
D43	E30	A30	21.733	0.12564	172.98	2.198	0.0011316	1942.4	1.3086	0.81564	1.7132	1.053
D43	E30	A45	18.288	0.34519	52.979	2.2592	0.0031519	716.76	3.5325	1.638	12.49	2.4271
D43	E30	A60	20.325	0.50919	39.917	2.423	0.005028	481.89	5.2261	2.8536	27.324	3.9638
D43	E30	A90	22.353	0.36606	61.064	3.1628	0.0047568	664.91	3.8053	2.3702	14.488	3.0804
D43	E30	A120	17.53	0.32848	53.367	2.4407	0.0032305	755.51	3.3529	1.8615	11.246	2.5638
D43	E30	A135	20.333	0.26467	76.825	2.218	0.0023921	927.23	2.7356	1.3167	7.4898	1.9299
D43	E30	A150	23.869	0.16348	146	2.1735	0.0014688	1479.8	1.7169	0.87957	2.9502	1.2627
D43	E30	A180	-17.617	0.63933	-27.556	2.6811	0.0060584	442.54	5.6806	3.2624	32.289	4.3946
D43	E45	A0	20.162	0.28424	70.934	2.2379	0.0025902	863.98	2.9347	1.7482	8.6167	2.3214
D43	E45	A30	24.557	0.19076	128.73	2.2045	0.0017433	1264.6	2.0083	1.1526	4.0352	1.532
D43	E45	A45	21.635	0.41193	52.521	2.2754	0.0038399	592.58	4.2641	2.0159	18.187	2.9777
D43	E45	A60	22.107	0.79897	27.669	2.5704	0.0084329	304.81	8.1443	4.4641	66.367	6.2334
D43	E45	A90	27.203	0.47702	57.027	3.0326	0.0060632	500.16	5.0388	2.8506	25.404	3.894
D43	E45	A120	20.311	0.40077	50.68	2.4813	0.0040521	612.34	4.1281	2.1569	17.055	3.096
D43	E45	A135	24.027	0.33454	71.822	2.2729	0.0031453	722.63	3.5041	1.76	12.286	2.5738
D43	E45	A150	20.776	0.20356	102.06	2.265	0.0018821	1203.4	2.11	1.109	4.4545	1.5727
D43	E45	A180	-4.502	0.41978	-10.725	2.5457	0.0039583	643.12	3.9328	2.3739	15.476	3.1213
D43	E60	A0	34.477	0.31785	108.47	2.2331	0.003067	728.11	3.478	1.6788	12.103	2.4721
D43	E60	A30	34.056	0.38	89.621	2.2275	0.0036511	610.09	4.1432	1.9636	17.174	2.8515
D43	E60	A45	32.982	0.69888	47.192	2.3455	0.0070409	333.12	7.481	3.4942	56.002	5.2327
D43	E60	A60	43.771	1.1569	37.835	2.5049	0.013057	191.84	12.498	7.2624	156.28	9.7372
D43	E60	A90	51.06	0.48505	105.27	2.962	0.0066812	443.32	5.6708	3.2156	32.174	4.383
D43	E60	A120	29.377	0.52469	55.989	2.5346	0.0056249	450.6	5.5813	3.0107	31.177	4.1961
D43	E60	A135	18.147	0.42965	42.236	2.4446	0.004243	576.15	4.384	2.1843	19.23	3.2033
D43	E60	A150	24.569	0.41455	59.266	2.285	0.0039273	581.83	4.3418	2.2304	18.859	3.1904
D43	E60	A180	23.748	0.35675	66.568	2.3155	0.0034132	678.39	3.7304	1.5626	13.922	2.447
D43	E90	A0	69.625	1.0737	64.843	4.5587	0.024921	182.93	13.034	7.0127	170.12	9.9216
D43	E-30	A0	19.707	0.23106	85.29	2.2144	0.0020797	1064.8	2.3837	1.3808	5.6851	1.8572
D43	E-30	A30	37.132	0.12908	287.67	2.3199	0.0013086	1772.8	1.4336	0.86207	2.0563	1.1405
D43	E-30	A45	22.24	0.24709	90.01	2.3449	0.0023792	985.57	2.5745	1.491	6.6359	1.9992
D43	E-30	A60	18.321	0.39385	46.519	2.5653	0.0040841	628.1	4.0258	2.2761	16.215	3.1058
D43	E-30	A90	27.716	0.30168	91.872	3.0703	0.00389	789.26	3.2105	1.6432	10.317	2.3859
D43	E-30	A120	8.6869	0.44673	19.446	2.5736	0.0044752	575.08	4.3921	2.7054	19.3	3.5266
D43	E-30	A135	9.1418	0.41629	21.96	2.3671	0.0038423	616.05	4.1036	2.2524	16.855	3.0955
D43	E-30	A150	17.341	0.21664	80.046	2.2286	0.0019438	1146.5	2.2145	1.2939	4.9065	1.7239
D43	E-30	A180	19.707	0.23106	85.29	2.2144	0.0020797	1064.8	2.3837	1.3813	5.6865	1.8576
D43	E-45	A0	23.29	0.36127	64.468	2.3402	0.0034867	671.17	3.7701	2.4873	14.224	3.127
D43	E-45	A30	23.205	0.25346	91.553	2.325	0.0024294	957.04	2.6508	1.3737	7.0285	1.9293
D43	E-45	A45	22.263	0.34653	64.244	2.3931	0.0034059	702.64	3.6029	1.7071	12.989	2.5795
D43	E-45	A60	29.827	0.45471	65.595	2.4741	0.004767	519	4.859	2.7911	23.617	3.7782
D43	E-45	A90	34.134	0.34954	97.655	3.0189	0.004553	663.05	3.8158	2.1698	14.569	2.9569
D43	E-45	A120	12.137	0.51791	23.434	2.6578	0.0054306	489.42	5.1473	3.0951	26.509	4.0894
D43	E-45	A135	12.905	0.50155	25.73	2.4125	0.0047879	503.87	5.0023	2.8756	25.04	3.8636
D43	E-45	A150	17.089	0.29944	57.07	2.2957	0.002765	830.25	3.0531	1.9003	9.3257	2.4537
D43	E-45	A180	18.749	0.31764	59.027	2.2608	0.0029077	777.51	3.2587	1.8416	10.626	2.5104
D43	E-60	A0	23.952	0.54302	44.11	2.504	0.0056238	445.26	5.6467	3.0619	31.895	4.3645
D43	E-60	A30	23.417	0.37276	62.821	2.4906	0.0038309	650.13	3.8909	1.7783	15.146	2.7402
D43	E-60	A45	23.426	0.32483	72.117	2.5132	0.0033687	746.05	3.395	1.4168	11.532	2.2844
D43	E-60	A60	26.658	0.3483	76.538	2.6048	0.0037938	686.6	3.6862	2.2402	13.593	2.9535
D43	E-60	A90	44.229	0.45468	97.275	2.8522	0.0058474	487.76	5.1644	3.1952	26.689	4.1537
D43	E-60	A120	26.658	0.3483	76.538	2.6048	0.0037938	686.6	3.6862	2.2403	13.595	2.9537
D43	E-60	A135	17.769	0.52948	33.56	2.5513	0.0054493	468.2	5.3759	2.9098	28.914	4.0549
D43	E-60	A150	19.853	0.39047	50.845	2.4414	0.0038774	629.66	4.0159	2.3417	16.144	3.1154
D43	E-60	A180	16.64	0.3566	46.663	2.4425	0.0034974	698.39	3.6246	2.0598	13.148	2.7926
D43	E-90	A0	41.379	0.50019	82.727	3.5151	0.0078291	448.98	5.6009	3.184	31.379	4.351
D50	E0	A0	38.631	0.21705	177.98	2.2243	0.0021236	1047.4	2.4231	1.4904	5.874	1.9468
D50	E30	A0	45.755	0.17792	257.16	2.1423	0.00173	1238.3	2.0508	1.1496	4.2079	1.5742
D50	E45	A0	41.697	0.19189	217.29	2.134	0.0018255	1169	2.172	1.3358	4.7193	1.7425
D50	E60	A0	49.336	0.2736	180.32	2.265	0.0028587	792.33	3.1982	1.5562	10.234	2.2937
D50	E90	A0	83.376	0.95354	87.438	4.5787	0.023865	191.86	12.497	6.8282	156.22	9.5667
D50	E-30	A0	39.02	0.28815	135.41	2.2959	0.002915	787.61	3.2172	2.1289	10.353	2.6784
D50	E-30	A45	-78.441	1.5063	-52.076	3.7958	0.01666	227.85	10.697	6.2584	114.51	8.3973
D50	E-30	A60	13.523	0.41079	32.92	2.4981	0.0040702	613.74	4.1188	2.3539	16.981	3.1811
D50	E-30	A90	26.292	0.26969	97.49	2.992	0.0033689	888.14	2.8553	1.4361	8.1568	2.099
D50	E-30	A120	2.1635	0.49945	4.3318	2.5286	0.0047956	527.28	4.784	3.0173	22.892	3.8831
D50	E-30	A135	-7.446	0.67906	-10.965	2.8088	0.0069907	401.79	6.2415	3.4187	38.999	4.696
D50	E-30	A180	31.644	0.18742	168.84	2.2571	0.0018059	1249.9	2.0319	1.164	4.1299	1.5771
D50	E-45	A0	38.041	0.38266	99.413	2.402	0.0040331	595.57	4.2429	2.8507	18.011	3.5485
D50	E-45	A30	26.052	0.23156	112.51	2.3224	0.002243	1035.4	2.4511	1.3172	6.0128	1.8121
D50	E-45	A45	12.312	0.39385	31.26	2.39	0.0037158	643.19	3.9324	2.2225	15.479	3.0125
D50	E-45	A60	-64.317	1.137	-56.569	3.8623	0.013338	289.56	8.5513	4.8836	73.161	6.566
D50	E-45	A90	35.376	1.3739	25.748	51.024	0.30462	167.5	14.071	8.3728	198.11	11.097
D50	E-45	A120	3.8975	0.78898	4.9399	3.0626	0.0092377	331.53	7.5154	4.3463	56.516	5.8375
D50	E-45	A135	3.4683	0.54419	6.3734	2.4588	0.0051062	481.54	5.2299	3.2444	27.365	4.2025
D50	E-45	A150	19.738	0.29057	67.929	2.2834	0.0026971	846.6	2.9945	1.8648	8.9735	2.4115
D50	E-45	A180	33.684	0.22778	147.88	2.3042	0.0022601	1019.5	2.4891	1.3903	6.1993	1.9019
D50	E-60	A0	37.946	0.47963	79.116	2.6116	0.0054945	475.31	5.2971	3.0576	28.079	4.1718
D50	E-60	A30	33.267	0.30004	110.87	2.544	0.0032812	775.31	3.2679	1.4571	10.687	2.2836
D50	E-60	A45	29.619	0.28699	103.21	2.5311	0.003075	823.13	3.0794	1.2275	9.4875	2.0502

Table P. Virtual NHANES, all. $c_0 = Int \quad c_1 = viewa$.

Dist	El	Az	Int	Int se	Int Tvalue	viewa	viewa se	viewa Tvalue	R se	CV RMSE	CV MSPE	CV MAPE
D43	E0	A0	19.57	0.05558	352.1	2.242	0.0009663	2320	2.47	1.502	6.104	1.961
D43	E0	A30	18.18	0.03485	521.9	2.279	0.0006099	3738	1.534	0.8903	2.355	1.199
D43	E0	A45	17.58	0.04623	380.2	2.319	0.0008197	2829	2.026	0.9566	4.108	1.423
D43	E0	A60	18.28	0.06814	268.2	2.471	0.001294	1910	2.999	1.677	8.995	2.304
D43	E0	A90	18.44	0.07561	243.9	3.562	0.002072	1719	3.331	1.943	11.1	2.562
D43	E0	A120	16.08	0.06067	265	2.44	0.00112	2178	2.631	1.296	6.921	1.882
D43	E0	A135	15.86	0.042	377.7	2.272	0.0007207	3152	1.819	0.748	3.311	1.15
D43	E0	A150	16.23	0.02876	564.2	2.241	0.0004882	4591	1.249	0.6925	1.561	0.9585
D43	E0	A180	15.88	0.04363	364	2.292	0.0007557	3034	1.89	1.034	3.572	1.455
D43	E30	A0	21.09	0.04597	458.7	2.204	0.0007944	2775	2.066	1.213	4.269	1.619
D43	E30	A30	19.92	0.04056	491	2.244	0.0007076	3171	1.808	0.8498	3.27	1.261
D43	E30	A45	18.98	0.07436	255.2	2.301	0.001322	1741	3.288	1.397	10.82	2.144
D43	E30	A60	18.77	0.1277	147	2.548	0.00251	1015	5.619	2.759	31.58	4.038
D43	E30	A90	22.29	0.0867	257.1	3.2	0.002194	1458	3.923	2.184	15.39	2.976
D43	E30	A120	20.16	0.0705	286	2.472	0.001357	1821	3.144	1.536	9.889	2.267
D43	E30	A135	19.87	0.0584	340.2	2.283	0.001036	2203	2.6	1.185	6.764	1.799
D43	E30	A150	18.66	0.03261	572.3	2.266	0.0005693	3980	1.441	0.6173	2.077	0.9739
D43	E30	A180	18.04	0.04034	447.2	2.277	0.0007046	3232	1.774	0.8974	3.148	1.312
D43	E45	A0	22.32	0.06057	368.5	2.27	0.001088	2087	2.745	1.366	7.534	1.97
D43	E45	A30	22.14	0.06758	327.7	2.286	0.001221	1873	3.058	1.246	9.353	1.931
D43	E45	A45	21.62	0.09998	216.3	2.358	0.001856	1270	4.499	1.931	20.24	2.961
D43	E45	A60	21.46	0.1579	135.9	2.667	0.003316	804.4	7.064	3.294	49.91	4.986
D43	E45	A90	26.54	0.1031	257.5	3.151	0.002651	1189	4.805	2.529	23.1	3.583
D43	E45	A120	23.48	0.07834	299.7	2.518	0.001574	1600	3.577	1.818	12.8	2.577
D43	E45	A135	21.72	0.06767	320.9	2.37	0.001263	1876	3.052	1.424	9.319	2.133
D43	E45	A150	20.69	0.06004	344.6	2.301	0.00108	2130	2.689	1.182	7.234	1.785
D43	E45	A180	19.99	0.05329	375.2	2.3	0.0009535	2412	2.376	0.9317	5.645	1.5
D43	E60	A0	24.93	0.09498	262.5	2.428	0.00186	1305	4.379	2.015	19.18	2.927
D43	E60	A30	25.66	0.1204	213.1	2.454	0.002397	1024	5.57	2.196	31.03	3.457
D43	E60	A45	25.76	0.1435	179.5	2.547	0.002968	858	6.63	2.893	43.97	4.436
D43	E60	A60	23.91	0.1616	148	3.044	0.003942	772.3	7.352	3.623	54.06	5.322
D43	E60	A90	30.92	0.1392	222.2	3.478	0.004086	851.2	6.683	3.72	44.67	5.102
D43	E60	A120	28.6	0.1157	247.3	2.619	0.002512	1043	5.471	3.402	29.93	4.363
D43	E60	A135	27.29	0.1062	256.9	2.418	0.002108	1147	4.979	2.701	24.79	3.713
D43	E60	A150	25.94	0.105	247	2.355	0.00201	1172	4.874	2.236	23.76	3.311
D43	E60	A180	24.23	0.1026	236.1	2.373	0.001954	1214	4.705	1.843	22.14	2.956
D43	E90	A0	30.23	0.2242	134.8	4.783	0.009029	529.8	10.6	6.136	112.3	8.173
D43	E-30	A0	19.22	0.05867	327.5	2.318	0.001052	2203	2.601	1.586	6.765	2.083
D43	E-30	A30	18.71	0.03832	488.2	2.348	0.0006933	3386	1.693	0.9425	2.868	1.297
D43	E-30	A45	17.82	0.05193	343.1	2.436	0.0009688	2514	2.28	1.108	5.197	1.612
D43	E-30	A60	17.79	0.06268	283.9	2.604	0.00125	2083	2.75	1.407	7.563	2.038
D43	E-30	A90	19.52	0.07313	267	3.192	0.00181	1764	3.247	1.635	10.55	2.367
D43	E-30	A120	12.68	0.08117	156.2	2.562	0.001537	1667	3.435	1.819	11.8	2.564
D43	E-30	A135	14.13	0.06645	212.6	2.338	0.00116	2016	2.841	1.252	8.078	1.881
D43	E-30	A150	15.57	0.04422	352.2	2.274	0.0007581	3000	1.911	0.8421	3.654	1.288
D43	E-30	A180	15.94	0.04158	383.3	2.277	0.0007154	3182	1.802	0.9135	3.247	1.304
D43	E-45	A0	19.13	0.07306	261.9	2.41	0.001361	1770	3.234	1.768	10.46	2.476
D43	E-45	A30	19.08	0.0633	301.4	2.408	0.001178	2044	2.802	1.228	7.855	1.868
D43	E-45	A45	19.26	0.07079	272	2.476	0.001357	1825	3.137	1.348	9.843	2.09
D43	E-45	A60	20.74	0.07887	263	2.624	0.001619	1621	3.531	2.073	12.47	2.766
D43	E-45	A90	20.94	0.08389	249.6	3.218	0.002115	1522	3.76	2.113	14.14	2.872
D43	E-45	A120	13.72	0.09694	141.5	2.661	0.001921	1385	4.128	2.074	17.04	2.969
D43	E-45	A135	15.2	0.08469	179.4	2.408	0.001534	1570	3.645	1.621	13.29	2.421
D43	E-45	A150	15.88	0.06491	244.7	2.34	0.001148	2039	2.81	1.174	7.897	1.827
D43	E-45	A180	16.43	0.05692	288.7	2.32	0.001002	2316	2.474	1.076	6.125	1.673
D43	E-60	A0	19.95	0.09468	210.7	2.599	0.001914	1358	4.211	1.909	17.74	2.95
D43	E-60	A30	19.82	0.08073	245.5	2.598	0.00163	1594	3.59	1.572	12.89	2.406
D43	E-60	A45	20.18	0.07155	282.1	2.619	0.00146	1794	3.191	1.494	10.19	2.217
D43	E-60	A60	20.86	0.07228	288.6	2.733	0.001547	1767	3.24	1.819	10.5	2.491
D43	E-60	A90	23.37	0.09687	241.2	3.13	0.002418	1295	4.415	2.679	19.5	3.538
D43	E-60	A120	16.99	0.1035	164.1	2.778	0.00219	1268	4.506	2.38	20.31	3.328
D43	E-60	A135	16.98	0.1035	164.1	2.587	0.00204	1268	4.506	2.121	20.31	3.121
D43	E-60	A150	16.99	0.1035	164.1	2.778	0.00219	1268	4.506	2.379	20.31	3.327
D43	E-60	A180	17.65	0.08235	214.3	2.46	0.00155	1587	3.606	1.484	13.01	2.374
D43	E-90	A0	28.84	0.1314	219.4	3.551	0.003877	916	6.217	3.113	38.67	4.524

Table Q. Virtual NHANES, males. $c_0 = Int \quad c_1 = viewa$.

Dist	El	Az	Int	Int se	Int Tvalue	viewa	viewa se	viewa Tvalue	R se	CV RMSE	CV MSPE	CV MAPE
D43	E0	A0	20.158	0.070701	285.12	2.2463	0.0011515	1950.7	2.117	1.2719	4.4832	1.6874
D43	E0	A30	19.043	0.058738	324.19	2.2676	0.00095854	2365.7	1.7461	1.086	3.0499	1.4083
D43	E0	A45	18.262	0.067524	270.45	2.3037	0.0011137	2068.6	1.9965	0.95901	3.9884	1.3873
D43	E0	A60	18.801	0.10501	179.05	2.4544	0.0018521	1325.2	3.1131	1.6926	9.6953	2.3585
D43	E0	A90	18.386	0.10434	176.21	3.5625	0.0026637	1337.4	3.0848	1.8219	9.5205	2.4201
D43	E0	A120	15.591	0.10023	155.54	2.4419	0.0017219	1418.2	2.9098	1.3804	8.4709	2.0397
D43	E0	A135	15.917	0.070588	225.49	2.2731	0.0011311	2009.7	2.055	0.80714	4.2254	1.2492
D43	E0	A150	16.752	0.044076	380.07	2.236	0.00069852	3201.1	1.2908	0.73771	1.6667	1.0022
D43	E0	A180	16.675	0.063642	262.02	2.2831	0.0010294	2218	1.8622	1.0545	3.4691	1.4422
D43	E30	A0	21.791	0.066228	329.03	2.197	0.0010667	2059.7	2.0052	1.1847	4.0224	1.5745
D43	E30	A30	20.687	0.064946	318.53	2.2325	0.001055	2116.1	1.9518	0.98539	3.8129	1.4015
D43	E30	A45	19.938	0.11478	173.71	2.2851	0.0018993	1203.1	3.4277	1.3579	11.755	2.1249
D43	E30	A60	19.779	0.19574	101.04	2.5145	0.0035623	705.85	5.8185	2.6793	33.867	4.0355
D43	E30	A90	21.037	0.11587	181.57	3.2131	0.0027159	1183.1	3.4856	1.9475	12.153	2.6686
D43	E30	A120	19.468	0.1159	167.98	2.4806	0.0020753	1195.3	3.4501	1.4453	11.908	2.3359
D43	E30	A135	19.972	0.093983	212.51	2.2817	0.001553	1469.2	2.809	1.2833	7.8923	1.9458
D43	E30	A150	19.001	0.052266	363.55	2.258	0.00084907	2659.4	1.5534	0.707	2.4142	1.0811
D43	E30	A180	18.852	0.062438	301.94	2.2641	0.0010161	2228.4	1.8536	1.016	3.4366	1.4219
D43	E45	A0	23.369	0.094376	247.61	2.2584	0.0015796	1429.8	2.8863	1.5017	8.3335	2.1235
D43	E45	A30	23.183	0.093469	248.03	2.2653	0.0015672	1445.5	2.855	1.2891	8.1571	1.9285
D43	E45	A45	22.956	0.14481	158.53	2.3331	0.0024975	934.17	4.4083	1.9106	19.451	2.8859
D43	E45	A60	22.943	0.22534	101.82	2.6132	0.0043558	599.94	6.8292	3.0863	46.67	4.7076
D43	E45	A90	25.692	0.1473	174.41	3.1578	0.0035041	901.16	4.5686	2.5229	20.879	3.4846
D43	E45	A120	22.109	0.13192	167.59	2.5358	0.0024584	1031.5	3.995	1.6597	15.975	2.6473
D43	E45	A135	21.419	0.10959	195.44	2.3733	0.0019023	1247.6	3.3061	1.4366	10.935	2.2333
D43	E45	A150	21.004	0.087542	239.93	2.2894	0.0014615	1566.4	2.6352	1.2647	6.9461	1.8285
D43	E45	A180	20.52	0.075702	271.06	2.2897	0.0012599	1817.4	2.272	1.0162	5.1645	1.5122
D43	E60	A0	26.867	0.11665	230.32	2.3927	0.0021194	1128.9	3.652	2.0089	13.344	2.7666
D43	E60	A30	27.529	0.14607	188.46	2.4065	0.0026823	897.19	4.5887	2.215	21.069	3.2262
D43	E60	A45	27.853	0.18645	149.39	2.4851	0.0035449	701.04	5.8579	2.76	34.325	4.0504
D43	E60	A60	26.561	0.22044	120.49	2.949	0.0049307	598.1	6.8499	3.3372	46.946	4.8386
D43	E60	A90	30.277	0.18836	160.73	3.4902	0.0051172	682.05	6.0188	3.5511	36.238	4.7333
D43	E60	A120	26.959	0.18444	146.17	2.6287	0.003686	713.16	5.7596	3.1921	33.188	4.3497
D43	E60	A135	26.451	0.16355	161.73	2.4117	0.0029875	807.25	5.0952	2.5193	25.975	3.6239
D43	E60	A150	25.924	0.16141	160.61	2.3351	0.0028443	820.96	5.0109	2.3677	25.117	3.4201
D43	E60	A180	24.434	0.15998	152.73	2.3546	0.0028133	836.93	4.9161	1.8696	24.188	2.9948
D43	E90	A0	30.111	0.33755	89.203	4.7867	0.012591	380.17	10.641	5.9425	113.31	8.0283
D43	E-30	A0	20.235	0.063112	320.63	2.3288	0.0010662	2184.2	1.891	1.1032	3.5775	1.4888
D43	E-30	A30	19.733	0.054944	359.15	2.3381	0.00092874	2517.5	1.6409	0.95925	2.6945	1.2865
D43	E-30	A45	18.562	0.064481	287.87	2.4239	0.0011212	2161.9	1.9105	1.0073	3.652	1.429
D43	E-30	A60	18.127	0.078671	230.41	2.588	0.0014564	1777	2.3235	1.1987	5.4016	1.7102
D43	E-30	A90	19.827	0.10398	190.67	3.1934	0.0024026	1329.1	3.1041	1.6899	9.6401	2.3695
D43	E-30	A120	12.921	0.11596	111.43	2.5613	0.0020535	1247.3	3.307	1.698	10.942	2.4052
D43	E-30	A135	14.582	0.10429	139.82	2.3389	0.0017048	1372	3.0074	1.2195	9.0488	1.8866
D43	E-30	A150	16.224	0.066174	245.17	2.2653	0.0010588	2139.4	1.9305	0.89027	3.7282	1.3242
D43	E-30	A180	16.7	0.050972	327.63	2.2592	0.00081592	2768.9	1.4921	0.88423	2.2272	1.1754
D43	E-45	A0	20.476	0.077665	263.64	2.419	0.0013651	1772	2.3301	1.3718	5.4323	1.8311
D43	E-45	A30	20.475	0.073746	277.64	2.3903	0.0012809	1866.2	2.2127	1.172	4.8984	1.6549
D43	E-45	A45	20.193	0.081733	247.06	2.4573	0.0014567	1687	2.4473	1.2263	5.9905	1.7706
D43	E-45	A60	21.489	0.10605	202.62	2.5965	0.0020149	1288.6	3.2012	1.9086	10.253	2.5222
D43	E-45	A90	21.555	0.12124	177.79	3.2063	0.0028458	1126.7	3.6593	2.0721	13.403	2.8329
D43	E-45	A120	14.492	0.15051	96.291	2.6445	0.0027807	951.03	4.3307	2.0232	18.762	2.9565
D43	E-45	A135	16.101	0.14147	113.82	2.3982	0.0023952	1001.2	4.1149	1.7952	16.942	2.7042
D43	E-45	A150	16.69	0.10531	158.49	2.3293	0.0017381	1340.1	3.0787	1.3872	9.4822	2.0692
D43	E-45	A180	17.382	0.084953	204.61	2.2992	0.0013903	1653.7	2.4963	1.1609	6.2348	1.7405
D43	E-60	A0	21.23	0.090473	234.66	2.6052	0.0017215	1513.3	2.7274	1.6014	7.4428	2.1298
D43	E-60	A30	21.183	0.087788	241.3	2.5782	0.0016526	1560.1	2.6457	1.5255	7.0021	2.0547
D43	E-60	A45	21.296	0.091062	233.86	2.5944	0.0017263	1502.9	2.7463	1.4948	7.5445	2.0835
D43	E-60	A60	21.705	0.10658	203.64	2.7091	0.0021159	1280.4	3.2218	1.8465	10.384	2.5065
D43	E-60	A90	24.4	0.1443	169.1	3.0919	0.003331	928.23	4.4363	2.5716	19.689	3.4759
D43	E-60	A120	18.75	0.16026	116.99	2.7361	0.003151	868.33	4.7399	2.3426	22.483	3.4044
D43	E-60	A135	18.57	0.16367	113.46	2.5569	0.0030036	851.27	4.8341	2.2102	23.374	3.2854
D43	E-60	A150	18.75	0.16026	116.99	2.7361	0.003151	868.33	4.7399	2.3423	22.472	3.4038
D43	E-60	A180	18.803	0.13795	136.31	2.4459	0.0024252	1008.6	4.0853	1.9631	16.702	2.9023
D43	E-90	A0	30.798	0.20043	153.66	3.4897	0.0054654	638.52	6.4231	3.3475	41.28	4.805

Table R. Virtual NHANES,females. $c_0 = Int \quad c_1 = viewa$.

Dist	El	Az	Int	Int se	Int Tvalue	viewa	viewa se	viewa Tvalue	R se	CV RMSE	CV MSPE	CV MAPE
D43	E0	A0	19.694	0.076477	257.51	2.2212	0.0014375	1545.2	2.4623	1.4897	6.0663	1.9533
D43	E0	A30	17.497	0.037826	462.57	2.2896	0.00072054	3177.6	1.1986	0.72797	1.4373	0.95478
D43	E0	A45	16.716	0.062398	267.89	2.3404	0.0012079	1937.6	1.9646	0.87773	3.8616	1.3722
D43	E0	A60	17.407	0.087409	199.14	2.498	0.0018158	1375.8	2.7647	1.5839	7.6447	2.1527
D43	E0	A90	18.472	0.11199	164.95	3.5618	0.0033446	1064.9	3.5678	2.0529	12.736	2.7046
D43	E0	A120	16.152	0.07083	228.04	2.4484	0.0014283	1714.2	2.2202	1.1852	4.9309	1.657
D43	E0	A135	15.947	0.048509	328.74	2.2667	0.00090416	2507	1.5189	0.67562	2.3077	1.0341
D43	E0	A150	15.915	0.036781	432.7	2.2426	0.00067806	3307.3	1.1516	0.61646	1.3269	0.86548
D43	E0	A180	15.328	0.059822	256.22	2.2981	0.0011252	2042.3	1.864	0.95427	3.4764	1.4018
D43	E30	A0	20.636	0.064054	322.17	2.2075	0.0012052	1831.6	2.0781	1.2163	4.3209	1.627
D43	E30	A30	19.244	0.049697	387.22	2.2558	0.00094526	2386.4	1.5955	0.70582	2.5475	1.0745
D43	E30	A45	18.057	0.097279	185.62	2.3194	0.0018857	1230	3.0911	1.3953	9.5604	2.1106
D43	E30	A60	16.954	0.16394	103.42	2.6073	0.0035446	735.59	5.15	2.6675	26.533	3.8589
D43	E30	A90	22.82	0.12737	179.17	3.2066	0.0035428	905.1	4.1935	2.0583	17.601	2.9809
D43	E30	A120	20.627	0.085242	241.98	2.4665	0.0017922	1376.3	2.7637	1.6171	7.6402	2.1841
D43	E30	A135	19.781	0.07338	269.57	2.2845	0.0014195	1609.4	2.3644	1.0988	5.5943	1.6438
D43	E30	A150	18.217	0.039796	457.77	2.2775	0.0007582	3003.8	1.2679	0.53415	1.6086	0.84119
D43	E30	A180	17.274	0.051499	335.43	2.2918	0.00098029	2337.8	1.6287	0.75472	2.6538	1.1507
D43	E45	A0	21.623	0.076599	282.29	2.2759	0.0014975	1519.8	2.5034	1.1674	6.2685	1.727
D43	E45	A30	20.964	0.097214	215.65	2.3145	0.0019231	1203.5	3.1588	1.1775	9.9851	1.8976
D43	E45	A45	20.222	0.13985	144.6	2.3901	0.0028416	841.13	4.5098	1.91	20.343	2.972
D43	E45	A60	18.507	0.21456	86.256	2.772	0.0049944	555.02	6.7969	3.4189	46.217	5.014
D43	E45	A90	26.821	0.14604	183.65	3.1624	0.004137	764.41	4.9579	2.3548	24.601	3.5225
D43	E45	A120	24.353	0.0891	273.32	2.5092	0.0019623	1278.7	2.9738	1.8188	8.8478	2.3851
D43	E45	A135	21.918	0.084269	260.1	2.3674	0.0017177	1378.2	2.7597	1.4145	7.6195	2.028
D43	E45	A150	20.047	0.082093	244.2	2.3225	0.0016179	1435.5	2.6499	1.1191	7.0266	1.7174
D43	E45	A180	19.408	0.076205	254.68	2.3141	0.001489	1554.1	2.4482	0.89146	5.9979	1.5077
D43	E60	A0	23.051	0.14851	155.21	2.4685	0.0031866	774.65	4.8931	1.8732	23.956	2.9822
D43	E60	A30	23.092	0.18674	123.65	2.529	0.0041087	615.54	6.1398	2.0962	37.711	3.5751
D43	E60	A45	22.404	0.20928	107.06	2.6555	0.0048102	552.05	6.8327	2.7824	46.703	4.4785
D43	E60	A60	19.204	0.21734	88.358	3.2245	0.0059169	544.97	6.9197	3.5438	47.895	5.1169
D43	E60	A90	31.348	0.20814	150.61	3.472	0.0067264	516.18	7.2972	3.8895	53.287	5.4631
D43	E60	A120	29.043	0.13947	208.24	2.6414	0.00336	786.14	4.8223	3.1118	23.267	3.9486
D43	E60	A135	26.999	0.13239	203.93	2.4538	0.0029139	842.12	4.5045	2.5345	20.302	3.4494
D43	E60	A150	24.838	0.12886	192.75	2.4076	0.0027347	880.39	4.3103	1.8744	18.585	2.8857
D43	E60	A180	23.248	0.12832	181.17	2.414	0.0026961	895.34	4.2389	1.7216	17.985	2.7703
D43	E90	A0	30.315	0.3067	98.845	4.7815	0.013567	352.45	10.552	6.342	111.46	8.3213
D43	E-30	A0	19.415	0.061402	316.2	2.2789	0.0011815	1928.9	1.9735	1.2233	3.8967	1.583
D43	E-30	A30	18.133	0.049281	367.96	2.3486	0.00096764	2427.1	1.5688	0.81641	2.4627	1.149
D43	E-30	A45	17.167	0.081686	210.16	2.4477	0.0016596	1474.9	2.5794	1.1495	6.659	1.7517
D43	E-30	A60	16.989	0.095066	178.7	2.6338	0.0020756	1268.9	2.9966	1.5234	8.9847	2.2284
D43	E-30	A90	19.551	0.104	188	3.1798	0.0027958	1137.4	3.3417	1.7159	11.175	2.4434
D43	E-30	A120	12.628	0.11646	108.44	2.5588	0.0023912	1070.1	3.5507	1.9345	12.609	2.7056
D43	E-30	A135	14.139	0.082519	171.35	2.3272	0.0015581	1493.7	2.5471	1.1843	6.491	1.7651
D43	E-30	A150	15.065	0.05987	251.63	2.2811	0.0011156	2044.7	1.8618	0.74637	3.4688	1.203
D43	E-30	A180	14.891	0.061869	240.68	2.3033	0.0011626	1981.2	1.9214	0.95448	3.6948	1.3701
D43	E-45	A0	19.134	0.088781	215.52	2.3694	0.0017726	1336.7	2.8452	1.2572	8.1009	1.9109
D43	E-45	A30	18.05	0.101	178.71	2.4211	0.0020437	1184.7	3.2089	1.0952	10.302	1.9144
D43	E-45	A45	18.252	0.11532	158.27	2.5003	0.0024138	1035.9	3.6674	1.3816	13.457	2.3514
D43	E-45	A60	19.351	0.11219	172.48	2.6731	0.0025317	1055.8	3.5984	1.8714	12.954	2.7016
D43	E-45	A90	20.463	0.11902	171.93	3.2281	0.0032717	986.66	3.8491	2.162	14.825	2.9209
D43	E-45	A120	12.856	0.12685	101.34	2.6828	0.0027357	980.66	3.8725	2.0859	15.002	2.9432
D43	E-45	A135	14.619	0.097964	149.23	2.4129	0.0019248	1253.5	3.0332	1.365	9.2055	2.0623
D43	E-45	A150	15.285	0.079135	193.15	2.3472	0.00152	1544.3	2.4638	0.92875	6.0738	1.5262
D43	E-45	A180	15.227	0.074003	205.77	2.3497	0.0014223	1652.1	2.3034	0.98396	5.3083	1.5342
D43	E-60	A0	19.882	0.1482	134.16	2.5621	0.0032196	795.78	4.7644	1.6866	22.711	2.9512
D43	E-60	A30	18.807	0.13453	139.81	2.6119	0.0029544	884.07	4.2925	1.5143	18.433	2.6458
D43	E-60	A45	18.92	0.10955	172.71	2.6531	0.0024454	1084.9	3.5024	1.3638	12.271	2.2739
D43	E-60	A60	19.705	0.097239	202.64	2.7707	0.0022804	1215	3.129	1.4966	9.794	2.2696
D43	E-60	A90	21.781	0.12779	170.45	3.1938	0.0035116	909.51	4.1733	2.2843	17.428	3.2038
D43	E-60	A120	14.887	0.13038	114.18	2.8366	0.0030185	939.75	4.04	2.2288	16.329	3.0775
D43	E-60	A135	15.398	0.12988	118.55	2.6223	0.0027903	939.78	4.0398	1.951	16.331	2.8545
D43	E-60	A150	14.887	0.13038	114.18	2.8366	0.0030185	939.75	4.04	2.2285	16.327	3.0773
D43	E-60	A180	16.889	0.093573	180.49	2.4665	0.0019118	1290.2	2.9475	1.0382	8.6925	1.7421
D43	E-90	A0	26.67	0.17199	155.07	3.6334	0.0055931	649.61	5.8225	2.715	33.939	4.0065

Table S. Virtual NHANES, all stature. $c_0 = Int \quad c_1 = viewa$**.**

Dist	El	Az	Int	Int se	Int t-value	viewa	viewa se	viewa t-value	Stature	Stature se	Stature t-value	R se	CV RMSE	CV MSPE	CV MAPE
D43	E0	A0	10.703	0.12601	84.932	2.069	0.0024207	854.72	1.2639	0.016717	75.604	2.0455	1.152	4.1867	1.588
D43	E0	A30	14.586	0.090549	161.09	2.2066	0.0018016	1224.8	0.52151	0.012245	42.589	1.4337	0.78001	2.0561	1.0924
D43	E0	A45	22.367	0.12748	175.46	2.4196	0.0026374	917.41	-0.70453	0.017616	-39.995	1.9079	0.97518	3.6426	1.4026
D43	E0	A60	27.784	0.18862	147.3	2.6848	0.0041829	641.85	-1.3969	0.026196	-53.324	2.7064	1.5906	7.3275	2.1377
D43	E0	A90	6.4035	0.1622	39.479	3.1872	0.0049637	642.11	1.7287	0.021554	80.201	2.7054	1.3973	7.3239	2.0204
D43	E0	A120	25.921	0.15431	167.98	2.6637	0.0034334	775.82	-1.4744	0.021776	-67.706	2.2495	1.204	5.0624	1.6795
D43	E0	A135	22.529	0.10351	217.66	2.4115	0.0021301	1132.1	-0.99698	0.014525	-68.637	1.55	0.71912	2.4041	1.0591
D43	E0	A150	15.216	0.078611	193.56	2.2206	0.0015664	1417.6	0.14931	0.01083	13.787	1.2399	0.65877	1.5382	0.93435
D43	E0	A180	12.409	0.11287	109.94	2.2202	0.0022968	966.63	0.51383	0.015519	33.11	1.8121	0.95914	3.2847	1.3677
D43	E30	A0	15.141	0.11827	128.02	2.0913	0.002224	940.32	0.83857	0.015625	53.668	1.862	0.98246	3.4691	1.3957
D43	E30	A30	22.128	0.11869	186.44	2.2879	0.002325	984.03	-0.31721	0.016048	-19.766	1.7804	0.88139	3.1726	1.2808
D43	E30	A45	31.968	0.20161	158.56	2.5735	0.0041678	617.47	-1.9019	0.028014	-67.889	2.81	1.2782	7.9029	1.9422
D43	E30	A60	40.184	0.38863	103.4	3.0576	0.009129	334.93	-3.1801	0.055205	-57.606	4.9941	2.6454	24.952	3.7447
D43	E30	A90	10.023	0.21237	47.196	2.8666	0.0057165	501.47	1.7078	0.027609	61.857	3.4321	1.7141	11.783	2.5262
D43	E30	A120	31.672	0.19718	160.62	2.7273	0.0043276	630.21	-1.6645	0.027087	-61.45	2.7549	1.5882	7.5941	2.1446
D43	E30	A135	30.572	0.15375	198.84	2.5028	0.003114	803.74	-1.55	0.021111	-73.421	2.1729	1.1283	4.7248	1.6144
D43	E30	A150	22.657	0.088396	256.31	2.347	0.0017681	1327.4	-0.5807	0.01209	-48.033	1.3237	0.62369	1.7536	0.93861
D43	E30	A180	15.721	0.11089	141.77	2.2302	0.0022139	1007.4	0.33681	0.015059	22.366	1.7397	0.86158	3.0281	1.2631
D43	E45	A0	23.322	0.18532	125.85	2.2898	0.0036146	633.49	-0.14129	0.024644	-5.7331	2.7411	1.3854	7.5167	1.9856
D43	E45	A30	27.803	0.20678	134.46	2.3996	0.0041019	584.99	-0.80084	0.027758	-28.85	2.9606	1.2617	8.7729	1.9443
D43	E45	A45	35.741	0.30841	115.89	2.6569	0.0064543	411.65	-2.0262	0.042254	-47.953	4.1339	1.8423	17.102	2.8336
D43	E45	A60	39.666	0.54195	73.191	3.1144	0.013169	236.5	-2.6503	0.07579	-34.969	6.7416	3.2122	45.474	4.8346
D43	E45	A90	12.272	0.27294	44.961	2.7823	0.0070557	394.33	1.9194	0.034549	55.556	4.3022	2.1741	18.518	3.1832
D43	E45	A120	31.25	0.24625	126.91	2.6883	0.0053563	501.89	-1.0896	0.03289	-33.128	3.4293	1.9066	11.769	2.5831
D43	E45	A135	33.642	0.19061	176.5	2.6202	0.0039647	660.87	-1.7011	0.025886	-65.715	2.6307	1.4441	6.9265	1.9999
D43	E45	A150	30.359	0.16697	181.83	2.4992	0.0033834	738.67	-1.3886	0.022758	-61.015	2.3602	1.0504	5.5739	1.6235
D43	E45	A180	24.726	0.15532	159.19	2.3969	0.0031357	764.41	-0.68091	0.021106	-32.261	2.2824	1.0172	5.2145	1.5386
D43	E60	A0	28.385	0.30997	91.576	2.4997	0.0064233	389.16	-0.47767	0.040857	-11.691	4.3551	2.0357	18.978	2.9413
D43	E60	A30	30.089	0.40649	74.02	2.5477	0.008551	297.94	-0.61229	0.053688	-11.405	5.5416	2.2092	30.722	3.4738
D43	E60	A45	31.191	0.49833	62.591	2.6672	0.010983	242.84	-0.75331	0.066277	-11.366	6.5966	2.9301	43.533	4.4558
D43	E60	A60	28.561	0.55358	51.592	3.1704	0.014881	213.05	-0.65767	0.074975	-8.7719	7.33	3.6758	53.748	5.3439
D43	E60	A90	3.2409	0.30017	10.797	2.731	0.0081913	333.4	3.5602	0.036191	98.373	5.0148	2.5922	25.159	3.7315
D43	E60	A120	28.285	0.39577	71.467	2.6125	0.0086412	302.33	0.042526	0.050839	0.83648	5.4707	3.3947	29.946	4.3591
D43	E60	A135	33.195	0.3651	90.918	2.5386	0.0074599	340.3	-0.80361	0.047598	-16.883	4.923	2.834	24.252	3.7885
D43	E60	A150	33.886	0.35462	95.556	2.5162	0.0071413	352.34	-1.0953	0.046779	-23.413	4.7705	2.343	22.774	3.399
D43	E60	A180	32.088	0.33664	95.319	2.5354	0.0069079	367.03	-1.099	0.044929	-24.461	4.596	1.8964	21.132	3.0199
D43	E90	A0	-19.367	0.20536	-94.306	3.0581	0.0073571	415.66	6.2144	0.023325	266.42	4.0967	2.1093	16.792	3.048
D43	E-30	A0	14.25	0.15769	90.367	2.2168	0.0031698	699.34	0.71367	0.021166	33.717	2.4897	1.485	6.2003	1.9854
D43	E-30	A30	18.29	0.10981	166.56	2.3388	0.0022578	1035.9	0.060381	0.014899	4.0528	1.6924	0.93573	2.865	1.2917
D43	E-30	A45	21.699	0.14729	147.32	2.5214	0.0031932	789.61	-0.56934	0.020299	-28.049	2.211	1.1706	4.89	1.6249
D43	E-30	A60	23.76	0.17748	133.87	2.745	0.0041354	663.77	-0.87772	0.024583	-35.704	2.6196	1.4602	6.8635	2.0059
D43	E-30	A90	5.1639	0.13304	38.816	2.798	0.0035987	777.49	2.0354	0.01744	116.7	2.2448	1.0697	5.0417	1.6186
D43	E-30	A120	24.866	0.2044	121.65	2.8642	0.0049388	579.94	-1.8936	0.029809	-63.526	2.9855	1.4744	8.9176	2.156
D43	E-30	A135	22.078	0.17446	126.55	2.5138	0.003766	667.51	-1.2122	0.024926	-48.632	2.6053	1.1861	6.7932	1.7825
D43	E-30	A150	17.563	0.12202	143.94	2.3155	0.0024978	926.99	-0.29704	0.017014	-17.459	1.8884	0.87268	3.5679	1.3091
D43	E-30	A180	16.982	0.11547	147.07	2.2984	0.0023553	975.86	-0.15548	0.016025	-9.7023	1.7951	0.93245	3.2244	1.3138
D43	E-45	A0	19.524	0.21377	91.328	2.4187	0.0045322	533.67	-0.056753	0.029086	-1.9512	3.2339	1.7758	10.461	2.4781
D43	E-45	A30	22.949	0.18537	123.8	2.4921	0.0039453	631.65	-0.56215	0.025352	-22.174	2.7488	1.2923	7.559	1.8969
D43	E-45	A45	26.013	0.20683	125.77	2.6269	0.0045476	577.64	-0.98195	0.028411	-34.562	2.997	1.4426	8.9858	2.1076
D43	E-45	A60	24.168	0.23951	100.9	2.7035	0.0055016	491.4	-0.49095	0.032422	-15.142	3.499	2.1616	12.248	2.803
D43	E-45	A90	4.3494	0.15336	28.362	2.7664	0.0041322	669.46	2.3197	0.019848	116.87	2.5979	1.3441	6.7513	1.9359
D43	E-45	A120	26.566	0.26082	101.86	2.9901	0.006525	458.26	-1.9819	0.037881	-52.32	3.7383	1.8098	13.978	2.6712
D43	E-45	A135	24.444	0.2338	104.55	2.6179	0.0051883	504.58	-1.4002	0.033306	-42.039	3.4119	1.6306	11.648	2.3946
D43	E-45	A150	22.246	0.17842	124.68	2.4776	0.0037925	653.29	-0.95283	0.025086	-37.982	2.6604	1.2208	7.0812	1.8345
D43	E-45	A180	21.775	0.15701	138.68	2.4337	0.0032854	740.76	-0.79459	0.021925	-36.241	2.3537	1.1415	5.5437	1.6794
D43	E-60	A0	24.418	0.28779	84.848	2.7035	0.0066229	408.21	-0.64649	0.039358	-16.426	4.1663	2.0128	17.365	2.9781
D43	E-60	A30	25.423	0.24221	104.96	2.728	0.0055657	490.15	-0.81018	0.033127	-24.457	3.5074	1.655	12.307	2.435
D43	E-60	A45	24.777	0.21426	115.64	2.7267	0.0049344	552.58	-0.66146	0.02914	-22.7	3.1276	1.5724	9.7868	2.2526
D43	E-60	A60	21.377	0.21691	98.552	2.7458	0.0051533	532.82	-0.073681	0.029163	-2.5265	3.2388	1.8352	10.493	2.4993
D43	E-60	A90	3.5141	0.17528	20.048	2.6176	0.0045051	581.03	2.7131	0.022224	122.08	2.9801	1.5816	8.8829	2.2368
D43	E-60	A120	27.095	0.3029	89.452	3.0394	0.0076979	394.83	-1.5097	0.042784	-35.287	4.2972	2.2831	18.476	3.2222
D43	E-60	A135	26.618	0.30324	87.78	2.8189	0.0071718	393.05	-1.4392	0.042804	-33.624	4.3152	2.1206	18.632	3.0978
D43	E-60	A150	27.095	0.3029	89.452	3.0394	0.0076979	394.83	-1.5097	0.042784	-35.287	4.2972	2.283	18.472	3.2219
D43	E-60	A180	26.573	0.23605	112.58	2.6612	0.0052467	507.22	-1.3198	0.032981	-40.018	3.395	1.549	11.532	2.3644
D43	E-90	A0	1.9335	0.25943	7.4527	2.8009	0.0072916	384.13	3.5099	0.031589	111.11	4.4077	2.2895	19.432	3.2922

Table T. Virtual NHANES, males stature.
$c_0 = Int \quad c_1 = viewa \quad c_2 = stat.$

Dist	El	Az	Int	Int se	Int t-value	viewa	viewa se	viewa t-value	Stature	Stature se	Stature t-value	R se	CV RMSE	CV MSPE	CV MAPE
D43	E0	A0	13.765	0.17061	80.679	2.1225	0.0032373	655.63	0.91604	0.022711	40.334	1.8889	1.1587	3.5704	1.5122
D43	E0	A30	15.19	0.15036	101.02	2.1913	0.0029148	751.77	0.55866	0.020262	27.572	1.6502	0.99194	2.7245	1.309
D43	E0	A45	24.623	0.17442	141.17	2.4352	0.0035258	690.7	-0.93824	0.024121	-38.898	1.7943	0.94357	3.2243	1.3326
D43	E0	A60	33.127	0.25924	127.78	2.7732	0.0056528	490.59	-2.12	0.036259	-58.467	2.51	1.5436	6.3088	2.0201
D43	E0	A90	9.9459	0.24751	40.184	3.2995	0.007525	438.47	1.2274	0.033256	36.906	2.7993	1.5361	7.8395	2.1458
D43	E0	A120	31.152	0.21077	147.8	2.7971	0.0046897	596.43	-2.3732	0.030243	-78.472	2.0732	1.1252	4.3045	1.5689
D43	E0	A135	25.06	0.15824	158.37	2.4645	0.0032264	763.87	-1.3813	0.022369	-61.75	1.6243	0.74346	2.6456	1.1105
D43	E0	A150	15.488	0.11645	133	2.2107	0.0022708	973.54	0.18744	0.016012	11.706	1.2771	0.70305	1.6332	0.97504
D43	E0	A180	11.237	0.14764	76.107	2.1726	0.0029163	744.98	0.80375	0.020134	39.921	1.665	0.90969	2.7746	1.2722
D43	E30	A0	16.393	0.1695	96.713	2.096	0.0031187	672.06	0.76349	0.022372	34.127	1.8433	1.0536	3.4007	1.4251
D43	E30	A30	23.451	0.18471	126.96	2.2863	0.0035344	646.89	-0.39781	0.024952	-15.943	1.914	1.0145	3.6705	1.4181
D43	E30	A45	34.749	0.30045	115.66	2.5893	0.0060599	427.28	-2.1715	0.041736	-52.03	2.8701	1.2399	8.2524	1.9364
D43	E30	A60	51.051	0.5563	91.769	3.2441	0.012774	253.97	-4.6672	0.079619	-58.619	4.6869	2.4279	21.997	3.491
D43	E30	A90	18.879	0.32852	57.468	3.1529	0.0090003	350.31	0.30928	0.044079	7.0165	3.4725	1.913	12.062	2.6462
D43	E30	A120	38.548	0.26948	143.05	2.9099	0.0059531	488.8	-2.8166	0.037768	-74.578	2.5189	1.3783	6.3552	1.9197
D43	E30	A135	33.105	0.23127	143.14	2.5497	0.0046333	550.3	-1.9199	0.031981	-60.033	2.2436	1.2201	5.0409	1.7077
D43	E30	A150	24.003	0.13437	178.63	2.3584	0.002639	893.66	-0.73154	0.018425	-39.704	1.3904	0.7086	1.9359	1.0276
D43	E30	A180	14.262	0.15599	91.428	2.1732	0.0030188	719.91	0.66626	0.021015	31.704	1.7224	0.95313	2.9684	1.3257
D43	E45	A0	25.68	0.28288	90.782	2.3031	0.005389	427.37	-0.32547	0.037576	-8.6616	2.8696	1.5234	8.2449	2.141
D43	E45	A30	28.976	0.27698	104.61	2.3783	0.0053278	446.4	-0.81923	0.037038	-22.118	2.7511	1.3228	7.5843	1.9371
D43	E45	A45	38.735	0.43168	89.731	2.6573	0.0087478	303.76	-2.2594	0.058922	-38.346	3.9725	1.8142	15.811	2.7781
D43	E45	A60	56.242	0.73871	76.135	3.4048	0.017354	196.2	-4.8515	0.10383	-46.724	5.8913	2.9659	34.764	4.3611
D43	E45	A90	22.136	0.44498	49.746	3.0636	0.011659	262.76	0.49089	0.058008	8.4625	4.5434	2.5112	20.653	3.4687
D43	E45	A120	42.317	0.35103	120.55	2.9914	0.0078001	383.5	-2.9187	0.04837	-60.341	3.1849	1.6216	10.168	2.3315
D43	E45	A135	37.086	0.28323	130.94	2.7032	0.0058704	460.49	-2.2669	0.038932	-58.227	2.6694	1.3832	7.1342	1.9759
D43	E45	A150	31.819	0.22792	139.6	2.508	0.0045293	553.73	-1.5636	0.031163	-50.174	2.23	1.0649	4.9795	1.5945
D43	E45	A180	24.176	0.21524	112.32	2.3631	0.0042399	557.33	-0.52757	0.029178	-18.081	2.2158	1.0923	4.9182	1.5601
D43	E60	A0	28.846	0.3695	78.069	2.4321	0.007303	333.03	-0.27102	0.048021	-5.6438	3.6431	2.0191	13.287	2.7775
D43	E60	A30	33.56	0.47908	70.052	2.5281	0.0095839	263.78	-0.82598	0.062567	-13.202	4.5273	2.2263	20.525	3.2623
D43	E60	A45	38.167	0.63821	59.803	2.7024	0.013341	202.56	-1.4189	0.084131	-16.865	5.7314	2.8101	32.886	4.116
D43	E60	A60	41.382	0.77136	53.648	3.3287	0.019579	170.02	-2.0756	0.10379	-19.997	6.6443	3.3409	44.209	4.8726
D43	E60	A90	11.118	0.48835	22.767	2.9612	0.01346	219.99	2.5219	0.060415	41.742	5.3318	3.008	28.433	4.1124
D43	E60	A120	47.203	0.62061	76.059	3.0872	0.013938	221.49	-2.8184	0.083111	-33.912	5.2999	3.1351	28.138	4.1962
D43	E60	A135	44.966	0.52884	85.029	2.7956	0.010869	257.21	-2.5808	0.070738	-36.484	4.6329	2.467	21.502	3.4798
D43	E60	A150	41.34	0.52144	79.281	2.6449	0.01038	254.82	-2.1547	0.069781	-30.878	4.6726	2.2869	21.86	3.3424
D43	E60	A180	35.556	0.50803	69.986	2.5818	0.010255	251.77	-1.5705	0.068381	-22.968	4.7241	1.862	22.355	3.0202
D43	E90	A0	-20.876	0.26275	-79.45	3.0333	0.0091914	330.02	6.4181	0.029642	216.52	3.6746	2.1057	13.53	2.8517
D43	E-30	A0	15.866	0.1629	97.4	2.2408	0.0032153	696.9	0.62675	0.021762	28.8	1.7785	1.0304	3.1649	1.3939
D43	E-30	A30	18.535	0.15213	121.83	2.3136	0.0030431	760.27	0.17318	0.020518	8.4404	1.6319	0.94205	2.6658	1.2718
D43	E-30	A45	21.868	0.17689	123.63	2.4953	0.0037331	668.43	-0.48506	0.024274	-19.983	1.8532	1.0534	3.4389	1.4352
D43	E-30	A60	26.766	0.19824	135.02	2.7898	0.004528	616.12	-1.2789	0.027567	-46.392	2.0081	1.1765	4.0363	1.5851
D43	E-30	A90	6.5754	0.197	33.378	2.8323	0.005252	539.28	1.8908	0.025893	73.024	2.2884	1.1563	5.2394	1.6881
D43	E-30	A120	29.904	0.24504	122.04	2.98	0.0058628	508.29	-2.6625	0.036029	-73.897	2.4247	1.3861	5.8871	1.8849
D43	E-30	A135	24.427	0.26214	93.183	2.5547	0.0055823	457.64	-1.5097	0.03758	-40.174	2.6855	1.2085	7.2263	1.8207
D43	E-30	A150	18.377	0.17765	103.44	2.3096	0.0035539	649.87	-0.32222	0.024727	-13.031	1.9053	0.92841	3.6354	1.3485
D43	E-30	A180	16.628	0.13722	121.17	2.2577	0.0027128	832.25	0.010752	0.018931	0.56798	1.4921	0.88291	2.228	1.1748
D43	E-45	A0	19.543	0.21916	89.175	2.3993	0.0045248	530.27	0.13408	0.029473	4.5493	2.3265	1.3663	5.4178	1.8237
D43	E-45	A30	22.277	0.21082	105.66	2.428	0.0043217	561.81	-0.25966	0.02849	-9.1141	2.1985	1.1917	4.8397	1.6694
D43	E-45	A45	27.205	0.22396	121.48	2.6097	0.0047748	546.55	-1.0179	0.030612	-33.252	2.2586	1.1829	5.1048	1.7009
D43	E-45	A60	29.915	0.30515	98.033	2.7887	0.0068464	407.32	-1.2125	0.041509	-29.212	3.0058	1.8461	9.0447	2.421
D43	E-45	A90	6.9156	0.24666	28.037	2.8117	0.0065298	430.59	2.0583	0.032041	64.24	2.8488	1.5547	8.1211	2.1791
D43	E-45	A120	35.864	0.36058	99.462	3.1859	0.0089025	357.87	-3.3196	0.052911	-62.74	3.4026	1.7064	11.591	2.472
D43	E-45	A135	28.146	0.38381	73.334	2.6675	0.0083657	318.86	-1.8302	0.054844	-33.371	3.7957	1.8846	14.423	2.7029
D43	E-45	A150	23.478	0.28501	82.375	2.4739	0.0059213	417.8	-1.0183	0.040024	-25.443	2.933	1.474	8.6129	2.1006
D43	E-45	A180	22.48	0.23006	97.716	2.4053	0.0046686	515.2	-0.75794	0.031988	-23.695	2.3929	1.2231	5.732	1.7563
D43	E-60	A0	24.026	0.26291	91.382	2.6687	0.0058648	455.03	-0.40097	0.035457	-11.309	2.7005	1.613	7.2996	2.1311
D43	E-60	A30	25.484	0.25351	100.52	2.6751	0.0056107	476.78	-0.61781	0.034279	-18.023	2.5807	1.5149	6.665	2.0309
D43	E-60	A45	26.734	0.26238	101.89	2.7178	0.005852	464.43	-0.78132	0.035526	-21.993	2.6474	1.4701	7.0144	2.0339
D43	E-60	A60	25.659	0.31402	81.712	2.8024	0.0072902	384.41	-0.56609	0.042361	-13.364	3.1777	1.8439	10.104	2.4826
D43	E-60	A90	6.4661	0.29826	21.68	2.6381	0.0074577	353.74	2.4587	0.037903	64.869	3.4404	1.9314	11.842	2.6463
D43	E-60	A120	35.763	0.45431	78.718	3.1635	0.011191	282.69	-2.5356	0.064239	-39.472	4.2475	2.2149	18.074	3.1545
D43	E-60	A135	31.129	0.4752	65.508	2.851	0.010898	261.6	-1.8706	0.066933	-27.947	4.5619	2.2844	20.83	3.2805
D43	E-60	A150	35.763	0.45431	78.718	3.1635	0.011191	282.69	-2.5356	0.064239	-39.472	4.2475	2.2148	18.062	3.1539
D43	E-60	A180	28.535	0.39385	72.452	2.6616	0.008547	311.4	-1.4396	0.054943	-26.202	3.8811	2.0165	15.085	2.8789
D43	E-90	A0	3.288	0.40839	8.0511	2.7498	0.011005	249.87	3.5686	0.049354	72.307	4.7566	2.6706	22.637	3.6798

Table U. Virtual NHANES,females stature.

$c_0 = Int \quad c_1 = viewa \quad c_2 = stat.$

Dist	El	Az	Int	Int se	Int t-value	viewa	viewa se	viewa t-value	Stature	Stature se	Stature t-value	R se	CV RMSE	CV MSPE	CV MAPE
D43	E0	A0	7.0614	0.11517	61.314	1.9657	0.0023077	851.79	1.8169	0.015408	117.92	1.3615	0.72884	1.8548	1.0325
D43	E0	A30	13.854	0.094372	146.8	2.2106	0.0020174	1095.7	0.54003	0.013079	41.288	1.0601	0.56973	1.125	0.80254
D43	E0	A45	21.577	0.17387	124.1	2.4511	0.0038962	629.1	-0.73313	0.024699	-29.682	1.837	0.93409	3.3788	1.3551
D43	E0	A60	25.533	0.24748	103.17	2.6958	0.0059372	454.06	-1.2225	0.035235	-34.695	2.5276	1.5207	6.3911	2.0151
D43	E0	A90	2.9316	0.20043	14.627	3.054	0.00647	472.03	2.2562	0.0269	83.872	2.434	1.2243	5.9286	1.7834
D43	E0	A120	23.431	0.18904	123.95	2.6236	0.0044741	586.39	-1.1065	0.027106	-40.822	1.9685	1.0071	3.8778	1.4369
D43	E0	A135	20.23	0.12953	156.18	2.3617	0.0028217	836.97	-0.65006	0.018475	-35.185	1.3854	0.66817	1.921	0.98147
D43	E0	A150	14.666	0.10175	144.13	2.2155	0.002167	1022.4	0.18854	0.014344	13.144	1.1358	0.56212	1.2916	0.82334
D43	E0	A180	13.387	0.16374	81.756	2.2547	0.00359	628.05	0.29457	0.023179	12.708	1.84	0.89796	3.3901	1.3456
D43	E30	A0	13.444	0.15686	85.71	2.0623	0.0031434	656.06	1.0325	0.021124	48.875	1.7626	0.82713	3.1108	1.2472
D43	E30	A30	21.173	0.14808	142.98	2.2969	0.0031167	736.96	-0.28304	0.020504	-13.804	1.5714	0.74792	2.4737	1.1049
D43	E30	A45	31.148	0.26179	118.98	2.6158	0.005851	447.06	-1.9653	0.037383	-52.571	2.5659	1.2498	6.5938	1.8534
D43	E30	A60	41.189	0.47658	86.428	3.2467	0.012405	261.73	-3.7265	0.070247	-53.048	4.263	2.2091	18.186	3.1678
D43	E30	A90	4.0697	0.23633	17.22	2.6785	0.006646	403.02	2.6124	0.030661	85.203	2.8367	1.3215	8.0552	2.0254
D43	E30	A120	27.37	0.25906	105.65	2.6233	0.0059688	439.5	-0.98235	0.035874	-27.383	2.6087	1.6538	6.8105	2.1213
D43	E30	A135	29.036	0.20128	144.25	2.4858	0.0043332	573.67	-1.3604	0.028131	-48.361	2.0114	1.0505	4.0533	1.4907
D43	E30	A150	22.192	0.10745	206.54	2.364	0.0023079	1024.3	-0.5897	0.015042	-39.204	1.1336	0.51102	1.2875	0.78515
D43	E30	A180	17.579	0.14941	117.66	2.2985	0.0032329	710.97	-0.04549	0.020935	-2.173	1.6282	0.75943	2.6537	1.1563
D43	E45	A0	20.749	0.23624	87.832	2.2576	0.0049172	459.12	0.12528	0.032039	3.9102	2.5005	1.1392	6.2591	1.7036
D43	E45	A30	28.655	0.30052	95.352	2.4823	0.0065004	381.86	-1.1187	0.041616	-26.881	2.9877	1.2475	8.9458	1.9361
D43	E45	A45	36.23	0.43575	83.145	2.7601	0.0099738	276.73	-2.367	0.061696	-38.366	4.0495	1.8391	16.417	2.8165
D43	E45	A60	44.258	0.73194	60.467	3.4943	0.020305	172.09	-3.9304	0.1077	-36.493	6.1603	3.0387	37.986	4.5295
D43	E45	A90	5.5302	0.30453	18.16	2.591	0.0082295	314.84	2.8657	0.03844	74.551	3.5897	1.7254	12.896	2.5951
D43	E45	A120	24.886	0.29086	85.561	2.5212	0.0065692	383.8	-0.074757	0.038802	-1.9266	2.9732	1.8251	8.8486	2.3893
D43	E45	A135	31.693	0.25024	126.65	2.5838	0.0055008	469.71	-1.4097	0.034446	-40.926	2.4456	1.4431	5.9932	1.9336
D43	E45	A150	31.725	0.22072	143.73	2.5811	0.0048415	533.12	-1.7161	0.030908	-55.522	2.1613	0.94718	4.6796	1.4763
D43	E45	A180	26.392	0.2216	119.1	2.468	0.0048318	510.78	-1.0288	0.030965	-33.224	2.2537	0.99143	5.0885	1.5286
D43	E60	A0	30.203	0.50116	60.266	2.6333	0.011485	229.29	-1.0253	0.068735	-14.917	4.8069	1.9605	23.143	3.0582
D43	E60	A30	33.699	0.65844	51.18	2.7832	0.015681	177.48	-1.5315	0.091335	-16.767	6.004	2.193	36.087	3.6544
D43	E60	A45	37.05	0.75608	49.003	3.0305	0.019228	157.61	-2.1404	0.10645	-20.107	6.6182	2.8713	43.846	4.5409
D43	E60	A60	35.706	0.75194	47.486	3.7558	0.02394	156.88	-2.4928	0.10912	-22.844	6.6429	3.4827	44.153	4.9976
D43	E60	A90	-3.0019	0.34407	-8.7246	2.5206	0.0097475	258.59	4.4056	0.041216	106.89	4.3106	2.1054	18.589	3.1222
D43	E60	A120	20.448	0.45169	45.27	2.4458	0.010337	236.6	1.1531	0.057825	19.942	4.6733	2.8454	21.857	3.739
D43	E60	A135	29.864	0.46446	64.298	2.5166	0.010176	247.3	-0.39444	0.061314	-6.4331	4.4898	2.605	20.187	3.4951
D43	E60	A150	34.844	0.44006	79.18	2.6287	0.0096923	271.21	-1.4104	0.059542	-23.687	4.1257	1.9916	17.048	2.9529
D43	E60	A180	34.241	0.42486	80.595	2.6613	0.0095088	279.88	-1.5733	0.058268	-27.001	4.0073	1.8196	16.089	2.7903
D43	E90	A0	-18.295	0.29736	-61.523	2.998	0.011323	264.76	6.1595	0.034328	179.43	4.2183	2.0919	17.809	3.1138
D43	E-30	A0	11.855	0.13923	85.151	2.1197	0.0029013	730.61	1.0967	0.018889	58.063	1.5849	0.78288	2.5162	1.1435
D43	E-30	A30	17.532	0.14365	122.05	2.3352	0.0031585	739.33	0.089008	0.019959	4.4596	1.5664	0.79648	2.4571	1.1322
D43	E-30	A45	22.049	0.23701	93.031	2.5637	0.0055476	462.12	-0.73384	0.033607	-21.836	2.4847	1.2692	6.183	1.794
D43	E-30	A60	23.426	0.27631	84.783	2.7994	0.0070008	399.86	-0.97166	0.039395	-24.665	2.8582	1.6474	8.1756	2.23
D43	E-30	A90	3.1174	0.15494	20.12	2.7071	0.0044163	612.99	2.3575	0.020574	114.59	1.8845	0.96949	3.5546	1.4065
D43	E-30	A120	20.789	0.31944	65.078	2.7732	0.0081976	338.29	-1.2909	0.047444	-27.209	3.3539	1.6298	11.251	2.3973
D43	E-30	A135	19.348	0.2272	85.159	2.4485	0.0051818	472.53	-0.80699	0.033017	-24.442	2.4314	1.1149	5.9174	1.6787
D43	E-30	A150	16.879	0.16833	100.27	2.3217	0.003701	627.32	-0.27711	0.024074	-11.511	1.8421	0.77985	3.3987	1.2287
D43	E-30	A180	18.903	0.16968	111.41	2.3947	0.0037896	631.92	-0.61555	0.024411	-25.216	1.8289	1.0105	3.3528	1.3913
D43	E-45	A0	18.518	0.26496	69.887	2.3556	0.0058606	401.94	0.090524	0.036665	2.469	2.844	1.2241	8.1036	1.8862
D43	E-45	A30	23.922	0.30186	79.249	2.5586	0.0069724	366.96	-0.87719	0.042669	-20.558	3.1038	1.3063	9.6445	2.0448
D43	E-45	A45	26.516	0.34776	76.248	2.701	0.0083343	324.08	-1.2362	0.049354	-25.047	3.4931	1.627	12.216	2.4616
D43	E-45	A60	22.189	0.34512	64.295	2.7453	0.0086861	316.06	-0.41823	0.048119	-8.6916	3.5767	1.9812	12.8	2.7487
D43	E-45	A90	1.5315	0.17172	8.9186	2.6826	0.0049153	545.76	2.6869	0.022539	119.21	2.1122	1.1532	4.4651	1.6116
D43	E-45	A120	20.764	0.35428	58.608	2.9005	0.0095297	304.37	-1.2493	0.052578	-23.76	3.7057	1.8618	13.738	2.6856
D43	E-45	A135	21.218	0.27424	77.37	2.5722	0.0064913	396.25	-1.0199	0.03987	-25.582	2.8833	1.3338	8.3215	2.0142
D43	E-45	A150	21.354	0.21848	97.74	2.4883	0.004986	499.06	-0.93015	0.031501	-29.527	2.3054	0.98974	5.3198	1.5438
D43	E-45	A180	23.224	0.19246	120.67	2.5364	0.0044111	575.02	-1.2279	0.027844	-44.099	2.0068	1.0662	4.0329	1.4952
D43	E-60	A0	23.367	0.4691	49.813	2.6475	0.011371	232.83	-0.51325	0.065583	-7.8261	4.7412	1.8355	22.504	3.05
D43	E-60	A30	25.803	0.4185	61.657	2.7887	0.010451	266.83	-1.042	0.059186	-17.605	4.1881	1.7659	17.556	2.7936
D43	E-60	A45	24.669	0.33481	73.683	2.7995	0.0084276	332.19	-0.85234	0.047045	-18.117	3.4124	1.5724	11.655	2.3804
D43	E-60	A60	18.962	0.29311	64.692	2.7514	0.0075575	364.06	0.10857	0.040418	2.6862	3.1274	1.4647	9.7892	2.2532
D43	E-60	A90	1.2554	0.18609	6.7465	2.6171	0.0052123	502.11	2.8754	0.024143	119.1	2.2917	1.2279	5.2565	1.7354
D43	E-60	A120	23.194	0.37783	61.387	3.0738	0.010585	290.39	-1.2864	0.055219	-23.297	3.8723	2.0492	15.004	2.8857
D43	E-60	A135	24.441	0.37851	64.572	2.86	0.0097733	292.64	-1.3941	0.055151	-25.278	3.8445	1.865	14.795	2.7706
D43	E-60	A150	23.194	0.37783	61.387	3.0738	0.010585	290.39	-1.2864	0.055219	-23.297	3.8723	2.0495	15.006	2.8859
D43	E-60	A180	24.794	0.26696	92.874	2.6576	0.0063534	418.29	-1.1956	0.038178	-31.317	2.7366	1.1847	7.5042	1.8022
D43	E-90	A0	0.72756	0.31775	2.2897	2.8398	0.0098017	289.73	3.4814	0.03977	87.538	3.8805	1.9073	15.074	2.8138

Table V. Virtual NHANES,kids. $c_0 = Int \quad c_1 = viewa$**.**

Dist	El	Az	Int	Int se	Int Tvalue	viewa	viewa se	viewa Tvalue	R se	CV RMSE	CV MSPE	CV MAPE
D43	E0	A0	17.031	0.057341	297.01	2.3205	0.0017231	1346.7	1.7985	1.0142	3.2367	1.3909
D43	E0	A30	16.869	0.038167	441.99	2.3164	0.0011426	2027.3	1.1956	0.65355	1.4304	0.91471
D43	E0	A45	17.11	0.034785	491.86	2.3382	0.0010541	2218.2	1.0928	0.56333	1.1949	0.81044
D43	E0	A60	17.988	0.050325	357.43	2.4972	0.0016455	1517.6	1.5964	0.9055	2.5507	1.2362
D43	E0	A90	14.839	0.063024	235.45	3.7373	0.0029751	1256.2	1.9277	1.0271	3.7184	1.4446
D43	E0	A120	16.953	0.046087	367.83	2.4203	0.0014431	1677.1	1.4448	0.7805	2.0888	1.0992
D43	E0	A135	16.224	0.027405	591.99	2.2671	0.00079702	2844.5	0.85233	0.45224	0.72682	0.64002
D43	E0	A150	15.417	0.025268	610.15	2.2655	0.00072761	3113.6	0.7787	0.40587	0.60674	0.58182
D43	E0	A180	14.84	0.036757	403.73	2.321	0.0010773	2154.4	1.1251	0.58102	1.2664	0.84035
D43	E30	A0	18.743	0.046235	405.39	2.2781	0.0013913	1637.4	1.4798	0.78758	2.1908	1.1254
D43	E30	A30	19.538	0.038521	507.19	2.2562	0.0011587	1947.2	1.2447	0.61887	1.5496	0.91164
D43	E30	A45	19.485	0.050314	387.26	2.2937	0.0015378	1491.6	1.6242	0.76201	2.6397	1.1446
D43	E30	A60	19.718	0.087422	225.56	2.5206	0.0029458	855.68	2.8251	1.282	7.9855	1.9595
D43	E30	A90	19.342	0.072564	266.55	3.3406	0.0032254	1035.7	2.3364	1.4581	5.4627	1.8681
D43	E30	A120	19.867	0.05372	369.81	2.5018	0.001799	1390.7	1.7417	0.98995	3.0361	1.3442
D43	E30	A135	19.539	0.044678	437.32	2.3079	0.0013748	1678.7	1.4434	0.76381	2.0842	1.0876
D43	E30	A150	18.584	0.028491	652.29	2.2682	0.00085192	2662.5	0.91056	0.45355	0.82972	0.66488
D43	E30	A180	17.223	0.032236	534.28	2.2985	0.00096149	2390.5	1.0141	0.52427	1.0289	0.75682
D43	E45	A0	20.804	0.054135	384.31	2.3263	0.0017046	1364.8	1.7747	0.88889	3.1514	1.3083
D43	E45	A30	21.684	0.052005	416.95	2.3058	0.0016402	1405.8	1.723	0.90746	2.9704	1.2882
D43	E45	A45	21.812	0.068825	316.92	2.3621	0.0022275	1060.4	2.2822	1.1318	5.2122	1.6418
D43	E45	A60	22.13	0.094576	234	2.6425	0.0034389	768.4	3.1436	1.5468	9.8888	2.2734
D43	E45	A90	22.615	0.089398	252.98	3.3515	0.0041467	808.24	2.9898	1.7684	8.9466	2.349
D43	E45	A120	22.41	0.066174	338.66	2.5826	0.0023585	1095	2.2104	1.3948	4.8887	1.7859
D43	E45	A135	21.531	0.053886	399.57	2.3934	0.0017609	1359.2	1.7819	0.98375	3.1767	1.3671
D43	E45	A150	20.837	0.048707	427.8	2.3025	0.0015184	1516.3	1.5977	0.78953	2.5539	1.1621
D43	E45	A180	19.824	0.040755	486.41	2.3075	0.001258	1834.2	1.3212	0.61469	1.747	0.9312
D43	E60	A0	23.584	0.076543	308.11	2.4855	0.0026636	933.14	2.5919	1.4907	6.7203	2.0146
D43	E60	A30	23.899	0.081336	293.83	2.5302	0.0028926	874.7	2.764	1.5298	7.6444	2.1173
D43	E60	A45	23.971	0.095361	251.37	2.6216	0.003518	745.19	3.2407	1.7022	10.509	2.4065
D43	E60	A60	22.08	0.10746	205.47	3.1208	0.0046134	676.47	3.5666	1.7411	12.729	2.5712
D43	E60	A90	22.662	0.11494	197.16	3.9477	0.0062872	627.9	3.8394	2.2242	14.757	2.9791
D43	E60	A120	25.032	0.10673	234.54	2.8052	0.0042697	657	3.6712	2.2435	13.486	2.9248
D43	E60	A135	24.834	0.089299	278.1	2.5387	0.0032238	787.48	3.068	1.838	9.419	2.43
D43	E60	A150	24.216	0.076853	315.09	2.4347	0.00264	922.22	2.6224	1.4332	6.8807	2.0013
D43	E60	A180	23.422	0.071312	328.44	2.4088	0.0024	1003.7	2.4107	1.2369	5.8176	1.7761
D43	E90	A0	20.29	0.18758	108.17	5.2964	0.013406	395.09	6.0475	3.3729	36.616	4.591
D43	E-30	A0	17.014	0.057051	298.22	2.3963	0.0017701	1353.8	1.7891	1.0517	3.2036	1.4071
D43	E-30	A30	17.156	0.037901	452.65	2.4035	0.0011812	2034.8	1.1912	0.66851	1.4199	0.91048
D43	E-30	A45	16.589	0.038112	435.27	2.4862	0.0012207	2036.7	1.19	0.64848	1.417	0.90345
D43	E-30	A60	16.853	0.045245	372.48	2.6526	0.0015509	1710.4	1.4168	0.79966	2.0082	1.0956
D43	E-30	A90	16.657	0.054069	308.07	3.3066	0.0023053	1434.3	1.6888	0.85851	2.8548	1.2186
D43	E-30	A120	15.204	0.051992	292.42	2.4731	0.0016306	1516.6	1.5974	0.70106	2.554	1.0964
D43	E-30	A135	15.481	0.03874	399.62	2.2964	0.0011317	2029.2	1.1944	0.5505	1.4275	0.84804
D43	E-30	A150	15.566	0.029092	535.08	2.2726	0.00084178	2699.7	0.89801	0.48454	0.80685	0.68399
D43	E-30	A180	15.08	0.032701	461.16	2.3064	0.00095502	2415	1.0038	0.57581	1.0081	0.78521
D43	E-45	A0	17.405	0.061202	284.4	2.4827	0.0019763	1256.2	1.9276	1.0883	3.7169	1.4975
D43	E-45	A30	17.774	0.048753	364.56	2.4626	0.0015681	1570.4	1.5428	0.79552	2.3809	1.1365
D43	E-45	A45	18.007	0.052919	340.28	2.5324	0.0017552	1442.8	1.6789	0.88805	2.82	1.2574
D43	E-45	A60	18.012	0.054957	327.74	2.7432	0.0019746	1389.2	1.7436	1.0006	3.0434	1.3541
D43	E-45	A90	16.713	0.068408	244.32	3.3926	0.0029949	1132.8	2.1369	1.1245	4.5718	1.5726
D43	E-45	A120	15.863	0.062888	252.25	2.5757	0.0020699	1244.4	1.9459	0.81896	3.7894	1.3171
D43	E-45	A135	16.258	0.054287	299.48	2.3721	0.0016528	1435.2	1.6879	0.74949	2.8514	1.1786
D43	E-45	A150	16.173	0.039363	410.87	2.333	0.0011775	1981.4	1.2232	0.59709	1.4968	0.89026
D43	E-45	A180	16.169	0.036331	445.06	2.3337	0.001087	2146.9	1.129	0.61728	1.2753	0.86459
D43	E-60	A0	18.472	0.068372	270.16	2.6714	0.0024055	1110.5	2.1796	1.1468	4.7534	1.6415
D43	E-60	A30	18.501	0.058589	315.78	2.6587	0.002052	1295.7	1.8691	0.95805	3.4958	1.3767
D43	E-60	A45	18.791	0.060675	309.69	2.6786	0.0021483	1246.9	1.9421	0.98939	3.7736	1.4199
D43	E-60	A60	19.132	0.066791	286.44	2.7989	0.0024811	1128.1	2.1458	1.0925	4.6061	1.5793
D43	E-60	A90	19.118	0.080942	236.2	3.2849	0.0035289	930.84	2.5983	1.3765	6.7537	1.9383
D43	E-60	A120	17.91	0.082002	218.41	2.7269	0.0029263	931.86	2.5954	1.0943	6.739	1.765
D43	E-60	A135	17.609	0.07422	237.26	2.5583	0.0024759	1033.3	2.3419	0.98326	5.4865	1.5917
D43	E-60	A150	17.91	0.082002	218.41	2.7269	0.0029263	931.86	2.5954	1.0944	6.7409	1.7653
D43	E-60	A180	17.441	0.060688	287.38	2.474	0.0019537	1266.3	1.9123	0.83008	3.6596	1.3253
D43	E-90	A0	23.009	0.11748	195.85	3.7954	0.0062045	611.71	3.9397	2.1463	15.533	2.9841

Table W. Virtual NHANES,kids stature.
$c_0 = Int \quad c_1 = viewa \quad c_2 = stat.$

Dist	El	Az	Int	Int se	Int t-value	viewa	viewa se	viewa t-value	Stature	Stature se	Stature t-value	R se	CV RMSE	CV MSPE	CV MAPE
D43	E0	A0	11.139	0.1741	63.979	2.1274	0.0056659	375.47	1.0657	0.030109	35.396	1.599	0.83924	2.5599	1.2157
D43	E0	A30	14.363	0.1294	110.99	2.2339	0.0042339	527.61	0.45553	0.022555	20.196	1.1472	0.58557	1.3172	0.85317
D43	E0	A45	18.716	0.12772	146.54	2.3919	0.0042386	564.31	-0.29193	0.022372	-13.049	1.0737	0.57411	1.154	0.81218
D43	E0	A60	21.611	0.19018	113.64	2.6255	0.0067024	391.72	-0.65223	0.033106	-19.701	1.5347	0.96985	2.3593	1.2442
D43	E0	A90	7.2082	0.16113	44.735	3.3237	0.008627	385.27	1.4208	0.028459	49.925	1.5597	0.77681	2.4358	1.1465
D43	E0	A120	19.407	0.17007	114.11	2.5056	0.0058704	426.83	-0.44781	0.029922	-14.966	1.4118	0.807	1.9963	1.0982
D43	E0	A135	18.183	0.096032	189.34	2.3314	0.0031243	746.2	-0.36069	0.017011	-21.204	0.81453	0.45122	0.6644	0.6227
D43	E0	A150	14.486	0.086531	167.41	2.2348	0.0028247	791.17	0.17289	0.015391	11.233	0.76858	0.39152	0.59145	0.57024
D43	E0	A180	12.618	0.11935	105.72	2.2454	0.0040123	559.63	0.41583	0.021334	19.491	1.0825	0.56687	1.1728	0.81084
D43	E30	A0	14.455	0.15448	93.572	2.1431	0.0048478	442.07	0.7583	0.026252	28.886	1.3643	0.6741	1.8623	1.0055
D43	E30	A30	18.984	0.14861	127.74	2.239	0.0046044	486.27	0.097047	0.025183	3.8537	1.2429	0.61422	1.5462	0.90763
D43	E30	A45	24.044	0.19551	122.98	2.4391	0.0062203	392.11	-0.80414	0.03345	-24.04	1.5332	0.79419	2.3554	1.1424
D43	E30	A60	28.336	0.36656	77.301	2.8255	0.012937	218.41	-1.5241	0.063168	-24.128	2.6658	1.4302	7.117	1.9924
D43	E30	A90	9.2116	0.19558	47.098	2.8812	0.0088548	325.39	1.7682	0.032656	54.145	1.8354	0.98195	3.372	1.3928
D43	E30	A120	20.884	0.21417	97.509	2.5369	0.0073637	344.51	-0.17806	0.036298	-4.9054	1.7375	1.0074	3.0233	1.3522
D43	E30	A135	23.108	0.17357	133.14	2.4221	0.0055406	437.15	-0.62839	0.029618	-21.216	1.3793	0.77521	1.9052	1.0655
D43	E30	A150	20.324	0.10708	189.8	2.3234	0.0033838	686.62	-0.30982	0.018414	-16.825	0.88453	0.43879	0.78366	0.64218
D43	E30	A180	16.546	0.11695	141.49	2.2764	0.0037925	600.22	0.12259	0.020365	6.0198	1.0103	0.52052	1.0219	0.75156
D43	E45	A0	18.568	0.21011	88.373	2.256	0.0066076	341.43	0.38557	0.035026	11.008	1.7526	0.84976	3.0747	1.2749
D43	E45	A30	20.623	0.21107	97.706	2.2731	0.0065223	348.51	0.18091	0.034884	5.1861	1.7183	0.89692	2.9569	1.2761
D43	E45	A45	24.306	0.29393	82.693	2.4415	0.0093665	260.66	-0.42621	0.048859	-8.7232	2.2643	1.1635	5.135	1.6682
D43	E45	A60	27.765	0.42742	64.959	2.8444	0.01533	185.54	-0.96357	0.071348	-13.505	3.0849	1.6288	9.5363	2.3049
D43	E45	A90	10.246	0.26841	38.173	2.8123	0.011759	239.17	2.0679	0.043164	47.908	2.4528	1.2976	6.0221	1.846
D43	E45	A120	17.825	0.25548	69.771	2.4262	0.0087398	277.6	0.77306	0.041702	18.537	2.1343	1.2628	4.5611	1.6737
D43	E45	A135	22.577	0.22376	100.9	2.4271	0.0072152	336.39	-0.17901	0.037174	-4.8154	1.7778	0.99753	3.1637	1.3743
D43	E45	A150	23.142	0.19876	116.43	2.3747	0.0062232	381.58	-0.39836	0.033338	-11.949	1.5743	0.80731	2.4823	1.1653
D43	E45	A180	20.808	0.16099	129.25	2.3387	0.005098	458.75	-0.17225	0.02726	-6.3188	1.3158	0.62453	1.7345	0.93623
D43	E60	A0	18.904	0.30967	61.047	2.3338	0.010083	231.45	0.77788	0.049956	15.571	2.5281	1.3928	6.3975	1.9226
D43	E60	A30	19.739	0.33828	58.351	2.3931	0.011196	213.74	0.68939	0.05447	12.656	2.7186	1.4457	7.399	2.0298
D43	E60	A45	21.025	0.41438	50.739	2.5204	0.014281	176.48	0.48921	0.06698	7.3038	3.2228	1.6354	10.402	2.3511
D43	E60	A60	21.486	0.46762	45.948	3.0958	0.01975	156.75	0.10151	0.07774	1.3058	3.5664	1.7232	12.735	2.5619
D43	E60	A90	4.665	0.28097	16.603	3.0335	0.01437	211.1	2.9931	0.044679	66.992	2.7494	1.3539	7.5681	2.0181
D43	E60	A120	11.725	0.36833	31.833	2.3314	0.013224	176.3	2.1628	0.057891	37.36	3.2257	1.6532	10.415	2.3883
D43	E60	A135	16.494	0.34666	47.579	2.2678	0.011338	200.01	1.3623	0.054936	24.798	2.8861	1.5777	8.3428	2.1868
D43	E60	A150	19.751	0.31801	62.109	2.294	0.010076	227.67	0.73626	0.050957	14.449	2.5666	1.3382	6.5957	1.9065
D43	E60	A180	20.885	0.29905	69.839	2.3287	0.0094747	245.78	0.42301	0.048454	8.7301	2.3917	1.1892	5.7311	1.725
D43	E90	A0	-9.4807	0.16715	-56.72	3.3018	0.011308	291.99	4.9956	0.025972	192.35	2.0348	1.0378	4.1483	1.4991
D43	E-30	A0	12.873	0.18937	67.976	2.2555	0.0063929	352.81	0.75084	0.032898	22.823	1.6981	0.92042	2.8859	1.3048
D43	E-30	A30	15.786	0.13526	116.71	2.3568	0.0045872	513.78	0.24826	0.023551	10.541	1.1775	0.63202	1.3878	0.88963
D43	E-30	A45	19.518	0.13604	143.48	2.5913	0.0048446	534.88	-0.53723	0.024046	-22.341	1.1318	0.64134	1.2825	0.8702
D43	E-30	A60	19.882	0.16535	120.24	2.7682	0.0062685	441.6	-0.55365	0.029155	-18.99	1.3657	0.81892	1.8669	1.0789
D43	E-30	A90	9.0227	0.13462	67.023	2.9502	0.0062355	473.13	1.3843	0.023258	59.522	1.2767	0.59778	1.6324	0.90466
D43	E-30	A120	21.998	0.16988	129.49	2.7224	0.0061755	440.84	-1.2764	0.030801	-41.441	1.3681	0.71273	1.8744	1.0152
D43	E-30	A135	18.009	0.1357	132.71	2.3814	0.0045239	526.4	-0.4709	0.024311	-19.37	1.1498	0.58512	1.3235	0.84726
D43	E-30	A150	15.102	0.10176	148.42	2.2572	0.0033303	677.78	0.086099	0.018088	4.7599	0.89595	0.47558	0.80347	0.67975
D43	E-30	A180	13.847	0.11074	125.03	2.2648	0.0036956	612.83	0.23028	0.019777	11.644	0.98979	0.54882	0.98041	0.76255
D43	E-45	A0	16.243	0.22459	72.323	2.4416	0.0078749	310.05	0.21038	0.039107	5.3796	1.922	1.0637	3.6955	1.4866
D43	E-45	A30	18.975	0.18434	102.94	2.5045	0.006397	391.51	-0.21651	0.032044	-6.7566	1.5355	0.81378	2.3592	1.1436
D43	E-45	A45	21.369	0.20153	106.03	2.6531	0.007201	368.44	-0.60496	0.03507	-17.25	1.6286	0.91561	2.6551	1.2458
D43	E-45	A60	19.286	0.20995	91.86	2.7926	0.0081011	344.72	-0.22897	0.036419	-6.2869	1.7365	1.0113	3.0196	1.3521
D43	E-45	A90	7.9675	0.17918	44.467	2.9742	0.0085061	349.65	1.5844	0.030902	51.272	1.7129	0.84593	2.9382	1.2534
D43	E-45	A120	22.169	0.22459	98.71	2.8146	0.0084393	333.52	-1.1738	0.040394	-29.06	1.7925	0.83504	3.2174	1.265
D43	E-45	A135	19	0.19882	95.562	2.4666	0.0067969	362.89	-0.50585	0.035339	-14.314	1.6526	0.79523	2.7352	1.185
D43	E-45	A150	17.259	0.14223	121.35	2.3697	0.0047629	497.53	-0.20005	0.025192	-7.9411	1.2153	0.61286	1.478	0.89558
D43	E-45	A180	16.934	0.13098	129.29	2.3594	0.0043831	538.3	-0.14073	0.023179	-6.0712	1.1248	0.63034	1.2663	0.87105
D43	E-60	A0	19.569	0.26632	73.48	2.7127	0.009973	272	-0.19613	0.046008	-4.263	2.1757	1.1688	4.7374	1.6496
D43	E-60	A30	20.271	0.22768	89.031	2.7249	0.0084758	321.49	-0.31598	0.039307	-8.0388	1.8567	0.98688	3.452	1.3836
D43	E-60	A45	20.602	0.23792	86.592	2.7465	0.0088955	308.76	-0.32227	0.040943	-7.8712	1.9297	1.0081	3.7263	1.4226
D43	E-60	A60	19.267	0.26155	73.667	2.8042	0.01016	276	-0.023915	0.044738	-0.53455	2.146	1.0938	4.6078	1.5801
D43	E-60	A90	9.2268	0.23479	39.297	2.8411	0.010509	270.36	1.7342	0.039393	44.024	2.188	1.0463	4.791	1.5863
D43	E-60	A120	23.108	0.32528	71.041	2.9299	0.012637	231.85	-0.94068	0.057065	-16.484	2.5241	1.1397	6.3759	1.7577
D43	E-60	A135	21.225	0.2883	73.62	2.6907	0.010505	256.14	-0.65572	0.050589	-12.962	2.3015	1.0404	5.3036	1.5986
D43	E-60	A150	23.108	0.32528	71.041	2.9299	0.012637	231.85	-0.94068	0.057065	-16.484	2.5241	1.1401	6.3805	1.7583
D43	E-60	A180	19.121	0.23008	83.105	2.5334	0.0080773	313.64	-0.30466	0.040253	-7.5687	1.901	0.85561	3.6191	1.3357
D43	E-90	A0	6.3437	0.33143	19.14	2.979	0.016341	182.3	2.7686	0.05283	52.406	3.133	1.4966	9.8237	2.2475

Table X. Virtual NHANES,adults males stature:
$WBSA = Intercept + viewaVBSA + StatStature$

Dist	El	Az	Int	Int se	Int t-value	viewa	viewa se	viewa t-value	Stature	Stature se	Stature t-value	R se	CV RMSE
D43	E0	A0	25.918	0.40787	63.544	2.1465	0.0035837	598.96	0.084977	0.034399	2.4703	1.8021	1.0806
D43	E0	A30	25.56	0.36554	69.924	2.1981	0.0032884	668.45	-0.086806	0.031037	-2.7969	1.6166	1.0102
D43	E0	A45	39.355	0.38371	102.56	2.5017	0.0038235	654.29	-2.1075	0.034174	-61.669	1.6512	0.90874
D43	E0	A60	50.2	0.49874	100.65	2.9512	0.0057443	513.76	-3.8716	0.046324	-83.575	2.0968	1.3508
D43	E0	A90	19.046	0.70094	27.172	3.3396	0.0097966	340.9	0.569	0.059065	9.6334	3.1301	1.8468
D43	E0	A120	38.522	0.46181	83.416	2.9166	0.0053827	541.85	-3.3147	0.043073	-76.955	1.9896	1.1292
D43	E0	A135	35.079	0.37832	92.724	2.5394	0.0038596	657.94	-2.3159	0.034244	-67.629	1.6421	0.71622
D43	E0	A150	23.775	0.29911	79.486	2.2275	0.0027329	815.07	-0.38237	0.025753	-14.848	1.3277	0.73889
D43	E0	A180	15.194	0.41423	36.681	2.1682	0.0037519	577.88	0.58888	0.034974	16.838	1.8671	1.0554
D43	E30	A0	29.931	0.38496	77.752	2.1207	0.0033141	639.91	-0.15493	0.032499	-4.7671	1.688	0.8938
D43	E30	A30	36.254	0.44441	81.577	2.3242	0.0041453	560.68	-1.3267	0.03873	-34.254	1.9237	1.1184
D43	E30	A45	56.815	0.67378	84.324	2.737	0.0071249	384.14	-4.1295	0.062414	-66.163	2.7877	1.2778
D43	E30	A60	96.665	1.0356	93.344	3.7569	0.013843	271.39	-9.4013	0.10499	-89.543	3.8942	2.1906
D43	E30	A90	24.926	0.89334	27.902	3.2533	0.012123	268.37	-0.36633	0.077456	-4.7296	3.9356	2.3275
D43	E30	A120	61.217	0.4353	140.63	3.1371	0.0051975	603.58	-5.082	0.041153	-123.49	1.7885	1.0253
D43	E30	A135	56.903	0.39515	144	2.6973	0.0040922	659.14	-3.9833	0.036281	-109.79	1.6392	0.93904
D43	E30	A150	34.948	0.30263	115.48	2.399	0.0029138	823.32	-1.564	0.026639	-58.712	1.3145	0.75422
D43	E30	A180	19.423	0.42957	45.214	2.1628	0.0038524	561.43	0.40608	0.036244	11.204	1.9211	1.1175
D43	E45	A0	51.843	0.62719	82.66	2.4076	0.0058907	408.72	-2.3337	0.055028	-42.41	2.6242	1.3596
D43	E45	A30	49.16	0.63828	77.019	2.4759	0.0062028	399.16	-2.4407	0.056555	-43.156	2.6855	1.4628
D43	E45	A45	71.671	0.94105	76.16	2.9015	0.010282	282.18	-5.2404	0.088055	-59.512	3.7525	1.9174
D43	E45	A60	122.35	1.4034	87.183	4.2362	0.020075	211.01	-11.861	0.14516	-81.709	4.9251	2.7319
D43	E45	A90	39.413	1.1748	33.548	3.2319	0.015574	207.53	-1.0559	0.10219	-10.333	5.0008	2.8177
D43	E45	A120	72.257	0.5959	121.26	3.3076	0.0073425	450.48	-5.9241	0.056681	-104.52	2.3859	1.2004
D43	E45	A135	61.031	0.53087	114.96	2.891	0.0058544	493.81	-4.4734	0.049215	-90.894	2.1801	1.1515
D43	E45	A150	49.374	0.46562	106.04	2.6131	0.0047535	549.72	-3.0647	0.042081	-72.828	1.9615	1.0704
D43	E45	A180	35.618	0.54965	64.801	2.4033	0.0053219	451.58	-1.3843	0.04813	-28.761	2.3802	1.2899
D43	E60	A0	61.726	0.80315	76.855	2.6151	0.0080566	324.59	-2.9544	0.070717	-41.778	3.2817	1.9129
D43	E60	A30	75.887	1.0324	73.508	2.8356	0.010949	258.99	-4.5566	0.093567	-48.699	4.0702	1.9985
D43	E60	A45	96.611	1.394	69.306	3.1917	0.016027	199.14	-6.7512	0.13064	-51.679	5.1924	2.6308
D43	E60	A60	131.28	1.5674	83.755	4.3116	0.022424	192.28	-10.571	0.15297	-69.107	5.3596	3.0191
D43	E60	A90	34.845	1.3802	25.247	3.1061	0.018003	172.53	0.7156	0.11392	6.2814	5.903	3.4645
D43	E60	A120	120.09	0.91925	130.64	3.8534	0.011878	324.4	-9.9108	0.089379	-110.89	3.2835	2.0106
D43	E60	A135	102.63	0.84021	122.15	3.322	0.0097411	341.03	-8.0867	0.080285	-100.72	3.129	1.6706
D43	E60	A150	90.331	1.0406	86.806	3.0425	0.011441	265.93	-6.6967	0.098115	-68.254	3.9697	1.9578
D43	E60	A180	65.428	1.247	52.47	2.7988	0.013506	207.23	-4.2397	0.11513	-36.824	5.0073	2.0487
D43	E90	A0	-44.751	0.68717	-65.124	2.9317	0.0092763	316.04	8.059	0.047737	168.82	3.3671	2.0072
D43	E-30	A0	32.764	0.32224	101.67	2.2862	0.0029706	769.62	-0.57251	0.027465	-20.845	1.4056	0.80072
D43	E-30	A30	35.18	0.29362	119.81	2.3586	0.0027779	849.07	-1.0096	0.025309	-39.893	1.2748	0.77837
D43	E-30	A45	37.228	0.40004	93.061	2.545	0.0040741	624.67	-1.6091	0.035141	-45.789	1.7287	1.0251
D43	E-30	A60	37.312	0.44069	84.667	2.8894	0.0050989	566.68	-2.3034	0.03972	-57.991	1.9035	1.0725
D43	E-30	A90	6.4707	0.5845	11.07	2.7969	0.0069727	401.13	2.0076	0.047581	42.193	2.6726	1.4573
D43	E-30	A120	37.284	0.62516	59.64	3.0548	0.0076841	397.55	-3.4011	0.058785	-57.856	2.6961	1.6608
D43	E-30	A135	34.622	0.72296	47.89	2.6172	0.0076803	340.77	-2.3877	0.06602	-36.167	3.1312	1.6416
D43	E-30	A150	28.534	0.48921	58.326	2.3443	0.0046794	500.98	-1.081	0.042926	-25.183	2.1494	1.1875
D43	E-30	A180	25.935	0.34246	75.731	2.2972	0.0032159	714.31	-0.72316	0.029752	-24.306	1.5136	0.91471
D43	E-45	A0	40.303	0.48356	83.346	2.4791	0.0047754	519.13	-1.4299	0.041973	-34.066	2.0754	1.1296
D43	E-45	A30	42.278	0.44089	95.893	2.5053	0.004378	572.24	-1.7731	0.03858	-45.96	1.8853	1.1158
D43	E-45	A45	49.608	0.41252	120.25	2.7213	0.0043793	621.41	-2.8073	0.0369	-76.077	1.7377	1.0132
D43	E-45	A60	65.729	0.48693	134.99	3.0125	0.0055329	544.47	-4.1989	0.044228	-94.938	1.9801	1.1477
D43	E-45	A90	14.986	0.70824	21.16	2.7991	0.008364	334.66	1.6159	0.057767	27.973	3.1864	1.801
D43	E-45	A120	60.195	0.84493	71.242	3.3754	0.010993	307.05	-5.4944	0.081717	-67.237	3.4617	1.9314
D43	E-45	A135	47.905	1.0212	46.908	2.7813	0.011337	245.32	-3.4841	0.094984	-36.681	4.283	2.4519
D43	E-45	A150	39.202	0.77121	50.832	2.5478	0.0079127	321.99	-2.2743	0.069536	-32.706	3.3072	1.947
D43	E-45	A180	36.895	0.59885	61.61	2.4767	0.005968	415	-1.9325	0.05341	-36.183	2.5854	1.4474
D43	E-60	A0	48.158	0.5704	84.427	2.7847	0.0062366	446.51	-2.2779	0.0503	-45.287	2.4067	1.3642
D43	E-60	A30	49.087	0.52796	92.975	2.7912	0.0057686	483.86	-2.4693	0.046764	-52.803	2.2242	1.26
D43	E-60	A45	50.478	0.52141	96.811	2.8228	0.0057436	491.47	-2.5951	0.046255	-56.105	2.1904	1.295
D43	E-60	A60	49.652	0.71819	69.135	2.8867	0.0081478	354.29	-2.2985	0.06332	-36.3	3.0156	1.8001
D43	E-60	A90	12.305	0.83076	14.812	2.602	0.0092173	282.29	2.2274	0.06666	33.414	3.7512	2.1704
D43	E-60	A120	76.805	0.98235	78.185	3.4232	0.012528	273.24	-5.9152	0.093033	-63.582	3.8691	2.3631
D43	E-60	A135	61.685	1.1991	51.444	3.0239	0.014128	214.04	-4.3607	0.11204	-38.923	4.8611	2.7487
D43	E-60	A150	76.805	0.98235	78.185	3.4232	0.012528	273.24	-5.9152	0.093033	-63.582	3.8691	2.3642
D43	E-60	A180	56.864	0.97697	58.204	2.8177	0.010744	262.25	-3.7625	0.089849	-41.876	4.0223	2.3258
D43	E-90	A0	23.337	1.1157	20.918	2.7293	0.012899	211.58	2.4479	0.087425	28	4.913	3.0222

Table Y. Virtual NHANES, adults females stature.
$c_0 = Int \quad c_1 = viewa \quad c_2 = stat.$

Dist	El	Az	Int	Int se	Int t-value	viewa	viewa se	viewa t-value	Stature	Stature se	Stature t-value	R se	CV RMSE
D43	E0	A0	11.783	0.3493	33.733	1.9676	0.0028662	686.49	1.5196	0.029189	52.06	1.4496	0.88549
D43	E0	A30	22.035	0.25795	85.422	2.2373	0.002353	950.82	-0.074886	0.022381	-3.3459	1.0486	0.64047
D43	E0	A45	35.59	0.50323	70.722	2.5318	0.0050639	499.98	-1.9294	0.04545	-42.452	1.9836	1.1782
D43	E0	A60	38.967	0.71671	54.369	2.8067	0.007975	351.94	-2.4775	0.065689	-37.715	2.7973	1.7173
D43	E0	A90	4.8538	0.69957	6.9382	3.0394	0.0090774	334.83	2.1732	0.058066	37.427	2.9358	1.6919
D43	E0	A120	31.121	0.54912	56.675	2.6886	0.0059322	453.21	-1.8384	0.04995	-36.806	2.1848	1.0657
D43	E0	A135	27.776	0.38475	72.194	2.4063	0.0037353	644.2	-1.3051	0.034523	-37.805	1.544	0.8058
D43	E0	A150	21.196	0.31794	66.669	2.2374	0.0029077	769.47	-0.30853	0.027884	-11.065	1.2943	0.75564
D43	E0	A180	21.679	0.53869	40.244	2.2802	0.0050373	452.66	-0.32236	0.047319	-6.8125	2.1874	1.3372
D43	E30	A0	26.945	0.46629	57.786	2.1053	0.0039738	529.79	0.016334	0.039963	0.40873	1.8735	1.0216
D43	E30	A30	32.129	0.42097	76.322	2.3535	0.003962	594.03	-1.1932	0.037277	-32.009	1.6731	0.91798
D43	E30	A45	48.833	0.72587	67.275	2.7548	0.0077496	355.48	-3.6192	0.067718	-53.445	2.7703	1.5642
D43	E30	A60	66.588	1.32	50.447	3.508	0.017518	200.24	-6.231	0.13044	-47.771	4.774	2.5087
D43	E30	A90	-1.6605	0.80657	-2.0587	2.645	0.0092691	285.35	3.0606	0.065669	46.606	3.4242	1.8649
D43	E30	A120	54.325	0.58972	92.12	2.8232	0.0063502	444.59	-3.4079	0.053829	-63.31	2.2264	1.3532
D43	E30	A135	52.291	0.3813	137.14	2.6386	0.0038422	686.74	-3.4267	0.034928	-98.109	1.4491	0.69228
D43	E30	A150	32.7	0.27113	120.61	2.4059	0.0025997	925.44	-1.4116	0.024144	-58.468	1.0772	0.5893
D43	E30	A180	25.024	0.47075	53.158	2.3248	0.004449	522.54	-0.61276	0.041466	-14.778	1.8991	1.0532
D43	E45	A0	38.638	0.70161	55.071	2.3476	0.0065321	359.39	-1.3437	0.06176	-21.756	2.7409	1.3813
D43	E45	A30	51.531	0.85505	60.267	2.6392	0.0087213	302.62	-3.1538	0.078131	-40.366	3.2367	1.5404
D43	E45	A45	62.95	1.2366	50.906	3.0013	0.014127	212.44	-4.9409	0.11781	-41.939	4.5212	2.3712
D43	E45	A60	62.08	2.0821	29.816	3.7896	0.03141	120.65	-6.0032	0.21151	-28.383	7.3948	4.1888
D43	E45	A90	6.0769	1.0352	5.8702	2.5839	0.011565	223.42	2.8477	0.084034	33.887	4.3147	2.3534
D43	E45	A120	56.129	0.66059	84.968	2.7575	0.0069286	397.98	-2.8672	0.058959	-48.63	2.4815	1.4802
D43	E45	A135	60.587	0.44756	135.37	2.8037	0.0047052	595.88	-4.0533	0.041121	-98.571	1.6679	0.97237
D43	E45	A150	52.914	0.50577	104.62	2.7295	0.0052745	517.49	-3.618	0.046619	-77.608	1.9174	0.92313
D43	E45	A180	44.317	0.62208	71.239	2.5722	0.0062508	411.49	-2.5542	0.056407	-45.281	2.4018	1.2388
D43	E60	A0	60.909	1.516	40.177	2.8718	0.01686	170.33	-3.7906	0.14037	-27.005	5.524	2.4227
D43	E60	A30	74.075	2.0192	36.685	3.1361	0.024249	129.33	-5.274	0.19291	-27.339	6.9908	3.0645
D43	E60	A45	80.523	2.2962	35.069	3.4545	0.030245	114.22	-6.2527	0.22481	-27.813	7.7211	4.2846
D43	E60	A60	59.184	2.2604	26.183	4.028	0.036989	108.9	-4.7051	0.22267	-21.131	8.0102	5.158
D43	E60	A90	6.8545	1.2282	5.5807	2.5189	0.013507	186.49	3.7922	0.096317	39.372	5.0934	2.7942
D43	E60	A120	71.708	1.1963	59.94	2.851	0.01268	224.85	-3.3672	0.10547	-31.926	4.2891	2.5792
D43	E60	A135	88.326	1.0389	85.016	2.9927	0.011034	271.21	-5.727	0.095142	-60.195	3.5945	2.0468
D43	E60	A150	86.611	1.0017	86.464	3.05	0.010879	280.36	-6.1751	0.093461	-66.071	3.4826	1.6777
D43	E60	A180	74.735	1.1131	67.139	2.9704	0.012166	244.16	-5.2281	0.10377	-50.383	3.9705	1.9936
D43	E90	A0	-35.441	1.0687	-33.163	2.8484	0.014301	199.17	7.5107	0.078263	95.967	4.7974	2.7299
D43	E-30	A0	25.914	0.389	66.617	2.1718	0.0034226	634.54	0.011698	0.033397	0.35027	1.5673	0.84274
D43	E-30	A30	34.505	0.35125	98.235	2.4201	0.0033778	716.47	-1.3043	0.031049	-42.007	1.3894	0.68372
D43	E-30	A45	45.068	0.65232	69.088	2.6964	0.0068643	392.82	-2.6781	0.059329	-45.14	2.5134	1.3349
D43	E-30	A60	44.587	0.79947	55.771	2.945	0.009235	318.9	-2.8138	0.073297	-38.389	3.0774	1.8806
D43	E-30	A90	-2.6465	0.50573	-5.2331	2.6602	0.0057932	459.2	2.857	0.041393	69.022	2.1568	1.2243
D43	E-30	A120	17.001	1.0148	16.753	2.7445	0.01174	233.77	-0.93381	0.092924	-10.049	4.136	2.414
D43	E-30	A135	21.649	0.74067	29.228	2.4666	0.0075369	327.27	-1.0161	0.066946	-15.178	3.0013	1.6492
D43	E-30	A150	22.4	0.55311	40.499	2.3494	0.0053227	441.39	-0.73024	0.049253	-14.826	2.2423	1.1461
D43	E-30	A180	31.582	0.49034	64.41	2.4764	0.0048681	508.71	-1.7416	0.044372	-39.25	1.95	1.1888
D43	E-45	A0	42.252	0.79727	52.996	2.4776	0.0077899	318.05	-1.8613	0.07102	-26.208	3.0853	1.425
D43	E-45	A30	49.209	0.89056	55.257	2.718	0.0094168	288.63	-3.0636	0.081609	-37.54	3.387	1.5039
D43	E-45	A45	57.067	0.98749	57.79	2.9081	0.010996	264.48	-3.9089	0.091645	-42.653	3.6815	1.8844
D43	E-45	A60	55.998	0.98234	57.005	2.9715	0.011204	265.21	-3.3075	0.089482	-36.963	3.6717	2.1853
D43	E-45	A90	3.0153	0.56772	5.3112	2.648	0.0064136	412.87	2.6987	0.046271	58.324	2.394	1.421
D43	E-45	A120	18.132	1.1171	16.231	2.8699	0.013548	211.82	-0.9577	0.1024	-9.3523	4.5334	2.749
D43	E-45	A135	28.528	0.8802	32.411	2.6122	0.0093866	278.29	-1.621	0.080317	-20.183	3.5072	2.0036
D43	E-45	A150	31.723	0.6882	46.095	2.5613	0.0070987	360.81	-1.8653	0.062695	-29.752	2.7304	1.3732
D43	E-45	A180	36.039	0.54296	66.375	2.6438	0.0057044	463.47	-2.4575	0.049944	-49.206	2.1373	1.2391
D43	E-60	A0	57.922	1.4715	39.361	2.8717	0.01646	174.46	-3.4639	0.13555	-25.555	5.4075	2.4433
D43	E-60	A30	57.849	1.2731	45.439	3.0125	0.014811	203.39	-3.8264	0.11826	-32.357	4.7062	2.2744
D43	E-60	A45	53.932	0.97735	55.182	2.9897	0.01127	265.27	-3.3359	0.089552	-37.251	3.671	1.8274
D43	E-60	A60	41.528	0.89579	46.36	2.8603	0.010154	281.7	-1.6565	0.079462	-20.847	3.4667	1.9683
D43	E-60	A90	-1.6927	0.57959	-2.9205	2.5521	0.0063715	400.54	3.2562	0.046677	69.762	2.466	1.3524
D43	E-60	A120	26.698	1.1796	22.633	3.069	0.015042	204.03	-1.4679	0.10824	-13.561	4.6927	3.0675
D43	E-60	A135	34.944	1.1997	29.126	2.9225	0.014294	204.46	-2.2568	0.1112	-20.294	4.6837	2.9067
D43	E-60	A150	26.698	1.1796	22.633	3.069	0.015042	204.03	-1.4679	0.10824	-13.561	4.6927	3.0678
D43	E-60	A180	45.363	0.7763	58.435	2.805	0.0085198	329.24	-3.0296	0.071565	-42.334	2.984	1.3795
D43	E-90	A0	7.3675	1.0892	6.7643	2.8051	0.013212	212.32	3.1661	0.08718	36.317	4.5236	2.5416

Table Z. Virtual Random combined errors, MAPE

Dist	El	Az	all	all stat	adults	adults stat	kids	kids stat	small	normal	big
D35	E0	A0	2.541	2.066	1.8757	1.8042	2.4264	1.7044	1.6183	1.7891	1.9351
D35	E0	A30	1.286	1.287	1.024	1.0183	1.1207	1.1011	0.92349	1.1039	1.1055
D35	E0	A45	1.736	1.6792	1.7104	1.3256	1.2051	1.164	0.89672	1.5914	1.5429
D35	E0	A90	2.103	2.1086	2.43	2.4302	1.1906	1.0878	0.72697	2.0233	2.7169
D35	E0	A150	2.241	2.0247	2.3819	1.6915	1.2741	1.2667	0.85738	2.1371	2.4663
D35	E0	A-60	2.47	2.3625	2.6795	2.0143	1.4661	1.448	1.0202	2.2911	2.918
D35	E0	A-90	2.701	2.6905	3.1371	3.0503	1.3337	1.3469	0.61922	2.5144	3.7978
D35	E0	A-135	1.516	1.3474	1.6764	1.1872	0.93629	0.94117	0.7374	1.4858	1.6671
D37	E35.5	A30	1.656	1.6527	1.3728	1.2946	1.1777	1.1757	0.92061	1.6068	1.1526
D37	E35.5	A60	3.998	3.9291	4.2419	3.5366	2.1358	1.9999	1.2877	3.7442	4.5022
D43	E0	A0	1.511	1.2254	1.3559	1.2805	1.2288	0.92524	0.81871	1.3132	1.4303
D43	E0	A30	1.121	1.0795	0.95254	0.94587	0.81538	0.71002	0.5471	0.91025	1.0991
D43	E0	A45	1.453	1.4348	1.4979	1.2852	0.88924	0.81719	0.5648	1.2997	1.4968
D43	E0	A60	2.094	2.0295	2.3873	1.8933	1.1809	1.1423	0.75722	1.9826	2.5964
D43	E0	A90	2.173	1.8603	2.0872	1.9922	1.6014	0.94778	0.85569	1.8359	2.3065
D43	E0	A120	1.846	1.6922	2.0717	1.5844	0.91099	0.90163	0.59311	1.8007	2.1637
D43	E0	A135	1.194	1.1368	1.4147	1.1088	0.61898	0.613	0.42127	1.1435	1.5739
D43	E0	A150	0.9678	0.92082	0.94475	0.94524	0.62982	0.5572	0.3931	0.89021	0.97199
D43	E0	A180	1.483	1.3521	1.5837	1.5504	0.74788	0.7126	0.52907	1.3347	1.9262
D43	E30	A0	2.13	2.0947	2.4909	2.4942	1.2597	1.1497	0.87396	1.2227	3.4164
D43	E30	A30	1.444	1.4431	1.1973	1.1638	1.0325	1.0317	0.82546	1.414	1.053
D43	E30	A45	2.454	2.3061	2.6012	2.0927	1.2329	1.2245	0.94106	2.4026	2.4271
D43	E30	A60	3.545	3.452	3.8115	3.0862	1.9088	1.8172	1.1564	3.355	3.9638
D43	E30	A90	2.345	1.9959	2.5535	2.3172	1.7825	1.0187	1.144	2.0552	3.0804
D43	E30	A120	2.057	1.974	2.1554	1.5472	1.3434	1.2808	0.78703	1.7527	2.5638
D43	E30	A135	1.916	1.8339	1.8737	1.2741	1.1558	1.127	0.71772	1.6226	1.9299
D43	E30	A150	1.267	1.2682	1.1521	1.0869	0.68587	0.67141	0.54142	1.0298	1.2627
D43	E30	A180	2.921	2.9011	3.343	3.2014	0.7902	0.76564	0.53521	1.3603	4.3946
D43	E45	A0	2.315	2.3208	2.1603	2.0509	1.6047	1.4669	1.0916	1.8867	2.3214
D43	E45	A30	2.33	2.3244	1.9515	1.758	1.6024	1.5644	1.0955	2.2615	1.532
D43	E45	A45	3.35	3.2864	3.3752	2.921	1.7862	1.7312	1.2538	3.3196	2.9777
D43	E45	A60	5.437	5.4336	5.7746	5.4338	2.6402	2.4134	1.5827	5.2394	6.2334
D43	E45	A90	2.933	2.7599	3.0654	3.0643	2.123	1.6207	1.2523	2.4362	3.894
D43	E45	A120	2.436	2.4146	2.5525	2.073	1.8118	1.5972	1.1284	2.0508	3.096
D43	E45	A135	2.208	2.1269	2.1772	1.5727	1.5302	1.4646	1.0243	1.815	2.5738
D43	E45	A150	1.644	1.5527	1.695	1.4081	1.0713	1.078	0.87994	1.6743	1.5727
D43	E45	A180	2.114	2.0919	2.5243	2.5158	0.88403	0.88301	0.79908	1.4213	3.1213
D43	E60	A0	3.482	3.487	2.981	2.7937	2.2301	2.0171	1.4664	3.133	2.4721
D43	E60	A30	4.101	4.0993	3.4425	3.1924	2.6057	2.3078	1.5247	3.7435	2.8515
D43	E60	A45	5.608	5.5534	5.5804	5.5024	3.0426	2.5499	1.7793	5.4389	5.2327
D43	E60	A60	8.418	7.9484	7.7114	7.6695	4.2119	3.6045	2.0185	7.2481	9.7372
D43	E60	A90	4.312	3.5415	3.7286	3.5513	3.3284	2.2223	1.6388	3.1376	4.383
D43	E60	A120	3.92	3.8908	3.8345	3.4595	3.2511	2.517	1.68	3.3587	4.1961
D43	E60	A135	3.281	3.2515	3.3293	2.6877	2.6752	2.3639	1.5735	2.9495	3.2033
D43	E60	A150	3.29	3.2667	3.0699	2.6417	2.5195	2.3169	1.4842	2.9391	3.1904
D43	E60	A180	2.764	2.741	2.7522	2.5726	1.9767	1.872	1.4027	2.7313	2.447
D43	E90	A0	8.453	4.6987	9.6379	4.9682	3.8638	1.6906	2.3873	6.8537	9.9216
D43	E-30	A0	1.442	1.4426	1.5004	1.4393	0.88551	0.82575	0.51561	1.2163	1.8572
D43	E-30	A30	1.798	1.6559	1.2475	1.2465	1.6623	1.1851	0.99839	1.0952	1.1405
D43	E-30	A45	1.654	1.6523	1.6589	1.5851	0.91019	0.89511	0.5597	1.3341	1.9992
D43	E-30	A60	2.012	2.0186	2.3324	2.2394	1.1076	1.0998	0.69928	1.7466	3.1058
D43	E-30	A90	2.009	1.3871	2.1243	1.6075	1.2424	0.75761	0.84963	1.7213	2.3859
D43	E-30	A120	2.665	2.5903	3.0166	2.8663	0.85899	0.85834	0.52811	2.6237	3.5266
D43	E-30	A135	2.357	2.2973	2.8074	2.7074	0.83308	0.83328	0.53712	2.3718	3.0955
D43	E-30	A150	1.569	1.5657	1.6817	1.6545	0.76557	0.75619	0.4611	1.5354	1.7239
D43	E-30	A180	1.442	1.4427	1.5004	1.4393	0.88569	0.82576	0.51563	1.2163	1.8576
D43	E-45	A0	2.602	2.5408	2.8951	2.8878	1.4933	1.3259	0.95317	2.5347	3.127
D43	E-45	A30	1.674	1.6656	1.7106	1.6749	1.137	1.0446	0.69719	1.4387	1.9293
D43	E-45	A45	2.142	2.1436	2.2402	2.1189	1.3617	1.2312	0.71178	1.8544	2.5795
D43	E-45	A60	2.909	2.8313	2.9366	2.8529	1.4024	1.2601	0.58125	2.3016	3.7782
D43	E-45	A90	2.577	1.6655	2.6496	1.8499	1.4309	0.95189	0.89965	2.1781	2.9569
D43	E-45	A120	3.167	3.106	3.4727	3.3036	1.0647	1.0642	0.61636	3.1163	4.0894
D43	E-45	A135	2.957	2.9456	3.4692	3.4559	0.90811	0.89389	0.6079	2.9634	3.8636
D43	E-45	A150	2.072	2.0539	2.3511	2.2982	0.89092	0.87077	0.52785	2.036	2.4537
D43	E-45	A180	1.918	1.9021	2.1122	2.0243	1.0019	0.93102	0.57642	1.7117	2.5104
D43	E-60	A0	3.309	3.2487	3.8774	3.8739	1.4258	1.3491	0.94031	3.2082	4.3645
D43	E-60	A30	2.218	2.2225	2.4088	2.3221	1.189	1.1641	0.8249	1.9814	2.7402
D43	E-60	A45	2.041	2.0406	2.0425	1.9599	1.2356	1.1838	0.80927	1.8137	2.2844
D43	E-60	A60	2.433	2.3954	2.4724	2.4727	1.4841	1.4069	1.0664	2.1522	2.9535
D43	E-60	A90	3.726	2.4589	3.4814	2.3971	2.2048	1.7089	1.2308	2.8599	4.1537
D43	E-60	A120	2.433	2.3954	2.4725	2.4728	1.4841	1.4071	1.0664	2.1523	2.9537
D43	E-60	A135	3.387	3.3794	3.8013	3.733	1.2456	1.2241	0.7835	3.3831	4.0549
D43	E-60	A150	2.593	2.5737	2.9227	2.8284	1.1803	1.1315	0.68614	2.4987	3.1154
D43	E-60	A180	2.336	2.2819	2.5455	2.2484	1.4203	1.3929	0.75802	2.1759	2.7926
D43	E-90	A0	3.569	2.747	3.6853	3.209	2.7559	1.5541	1.7254	2.9749	4.351
D50	E0	A0	2.166	1.6889	1.8162	1.6778	1.7812	1.2808	1.2506	1.6877	1.9468
D50	E30	A0	2.766	2.6473	1.9173	1.8975	2.6041	1.9424	1.7274	1.9414	1.5742
D50	E45	A0	2.275	2.0428	1.5698	1.5704	2.0461	1.5482	1.3855	1.4348	1.7425
D50	E60	A0	4.103	3.9854	2.9005	2.7902	3.8772	2.6133	2.291	3.0653	2.2937
D50	E90	A0	8.604	4.0675	8.9392	4.6556	4.9825	1.4125	3.1875	6.4275	9.5667
D50	E-30	A0	2.709	2.3313	2.5465	2.5097	2.0128	1.4291	1.2406	2.2635	2.6784
D50	E-30	A45	6.189	6.0626	5.815	5.3848	1.4381	1.2818	0.94417	1.4292	8.3973
D50	E-30	A60	2.067	2.0801	2.4628	2.3591	1.0021	1.001	0.56537	1.872	3.1811
D50	E-30	A90	1.785	1.3696	1.8181	1.5634	1.1265	0.78362	0.7362	1.4991	2.099
D50	E-30	A120	2.935	2.817	3.2987	3.1012	0.82673	0.79456	0.47334	2.8574	3.8831
D50	E-30	A135	3.861	3.7669	4.3704	4.2664	1.4748	1.4376	1.6297	3.7385	4.696
D50	E-30	A180	1.662	1.5905	1.3499	1.3476	1.5074	1.1154	0.92448	1.1677	1.5771
D50	E-45	A0	3.218	2.9604	3.3214	3.3117	2.0925	1.6418	1.2838	2.8821	3.5485
D50	E-45	A30	1.675	1.6319	1.5932	1.5849	1.2277	1.0545	0.678	1.3417	1.8121
D50	E-45	A45	2.201	2.2274	2.4965	2.412	1.0944	1.06	0.57861	1.9991	3.0125
D50	E-45	A60	5.551	5.5349	5.5935	5.485	1.3076	1.2141	0.92685	2.6584	6.566
D50	E-45	A90	8.643	8.0128	9.7985	8.5963	3.363	3.1826	2.122	8.7838	11.097
D50	E-45	A120	5.95	5.922	5.9171	5.9135	2.9106	2.869	1.8162	6.0845	5.8375
D50	E-45	A135	3.233	3.2421	3.7902	3.8137	0.69799	0.6478	0.49451	3.3449	4.2025
D50	E-45	A150	2.057	2.0568	2.2948	2.2761	0.91396	0.84724	0.53189	1.9873	2.4115
D50	E-45	A180	2.033	1.9928	1.5891	1.5304	1.9601	1.3962	0.99904	1.2573	1.9019
D50	E-60	A0	3.401	3.2017	3.5453	3.5488	2.216	1.7203	1.4258	2.8532	4.1718
D50	E-60	A30	2.3	2.2672	2.0958	2.0312	1.7726	1.4969	1.0843	1.7635	2.2836
D50	E-60	A45	2.106	2.09	1.9307	1.8611	1.5716	1.3268	0.90796	1.6936	2.0502
D50	E-60	A60	2.435	2.3999	2.3879	2.3746	1.5538	1.409	1.0334	2.0964	2.7164
D50	E-60	A90	3.693	2.4579	3.2848	2.3641	2.3412	1.7665	1.1985	2.762	3.9544
D50	E-60	A120	3.746	3.7472	3.9546	3.9128	1.4525	1.3872	0.88136	3.6381	4.4883
D50	E-60	A135	3.261	3.2681	3.5744	3.5353	1.5651	1.3384	0.9172	3.1536	3.7951
D50	E-60	A150	2.504	2.4874	2.435	2.4101	1.8684	1.3415	1.0018	2.0173	2.6961
D50	E-60	A180	2.433	2.3638	1.9392	1.7803	2.3215	1.6043	1.1952	1.5638	2.2491
D52	E30	A-30	2.332	2.2682	1.4059	1.4009	2.7136	1.8844	1.7754	1.5857	1.2177

Table AA. Virtual Random combined errors, RMSE

Dist	El	Az	all	all stat	adults	adults stat	kids	kids stat	females	males	small	normal	big
D35	E0	A0	1.829	1.5761	1.4399	1.4399	1.7418	1.3084	1.6766	1.7537	1.23	1.3794	1.4863
D35	E0	A30	0.9405	0.97975	0.78003	0.78003	0.79818	0.83684	0.80591	1.087	0.73051	0.86594	0.85557
D35	E0	A45	1.197	1.1197	1.2024	1.2024	0.85116	0.84696	1.2838	1.107	0.68606	1.1297	1.0388
D35	E0	A90	1.508	1.512	1.7671	1.7671	0.84669	0.76091	1.4161	1.5883	0.47507	1.5372	1.9767
D35	E0	A150	1.644	1.4074	1.7717	1.7717	0.91337	0.91733	1.6758	1.5924	0.66104	1.578	1.7961
D35	E0	A-60	1.784	1.659	2.0281	2.0281	1.0311	1.025	1.9926	1.583	0.76248	1.6791	2.2321
D35	E0	A-90	1.805	1.8139	2.1491	2.1491	0.88578	0.98211	1.6052	2.0386	0.41893	1.8909	2.5639
D35	E0	A-135	1.026	0.87013	1.1414	1.1414	0.66752	0.67782	1.1143	0.98533	0.56485	1.0375	1.1213
D37	E35.5	A30	1.225	1.2166	1.0035	1.0035	0.81982	0.86552	1.2726	1.1984	0.72205	1.2166	0.88614
D37	E35.5	A60	2.827	2.7496	3.0997	3.0997	1.4904	1.3817	3.2791	2.3838	0.94965	2.6857	3.2262
D43	E0	A0	1.147	0.93259	1.0292	1.0292	0.91736	0.70217	1.1826	1.0521	0.60491	1.006	1.0835
D43	E0	A30	0.8062	0.82289	0.722	0.722	0.57644	0.53138	0.68683	0.93828	0.41146	0.706	0.83726
D43	E0	A45	0.9729	0.94693	1.0414	1.0414	0.6377	0.59355	1.0303	0.89237	0.42676	0.88128	1.0398
D43	E0	A60	1.488	1.4177	1.7931	1.7931	0.85416	0.81704	1.6565	1.3344	0.56155	1.4318	1.9738
D43	E0	A90	1.654	1.356	1.5198	1.5198	1.2585	0.67813	1.5097	1.7044	0.60597	1.3596	1.7287
D43	E0	A120	1.297	1.1411	1.5084	1.5084	0.66031	0.64958	1.3741	1.2275	0.44289	1.2876	1.5368
D43	E0	A135	0.7769	0.75629	0.95703	0.95703	0.43963	0.43398	0.85083	0.74106	0.31634	0.76905	1.0966
D43	E0	A150	0.7165	0.67847	0.70415	0.70415	0.45475	0.40085	0.74915	0.68093	0.2844	0.6561	0.74336
D43	E0	A180	1.091	0.95343	1.1742	1.1742	0.53586	0.50237	1.081	1.0986	0.3783	0.9902	1.4792
D43	E30	A0	1.391	1.3575	1.6639	1.6639	0.92321	0.87455	1.4062	1.4291	0.67212	0.891	2.4553
D43	E30	A30	1.081	1.0796	0.88909	0.88909	0.7253	0.76007	1.0811	1.0763	0.64409	1.087	0.81564
D43	E30	A45	1.66	1.4693	1.7612	1.7612	0.85988	0.85649	1.8581	1.4395	0.70703	1.6072	1.638
D43	E30	A60	2.516	2.4107	2.7983	2.7983	1.3496	1.2758	2.8847	2.1531	0.86583	2.4058	2.8536
D43	E30	A90	1.713	1.3774	1.8764	1.8764	1.4367	0.73694	1.4225	1.6234	0.88284	1.4799	2.3702
D43	E30	A120	1.455	1.3812	1.5269	1.5269	0.95812	0.92717	1.4709	1.3822	0.60584	1.2777	1.8615
D43	E30	A135	1.363	1.2676	1.3286	1.3286	0.81599	0.80709	1.1976	1.5192	0.54601	1.1791	1.3167
D43	E30	A150	0.8719	0.87366	0.80438	0.80438	0.45136	0.46917	0.84786	0.84842	0.41109	0.73252	0.87957
D43	E30	A180	1.944	1.9884	2.3572	2.3572	0.54509	0.53019	1.4312	2.4828	0.3938	0.99134	3.2624
D43	E45	A0	1.687	1.7148	1.5689	1.5689	1.1369	1.1115	1.6163	1.7316	0.85095	1.4031	1.7482
D43	E45	A30	1.698	1.6844	1.4049	1.4049	1.1394	1.1742	1.8345	1.6202	0.8487	1.6745	1.1526
D43	E45	A45	2.282	2.1858	2.2582	2.2582	1.2488	1.237	2.7513	1.8835	0.95028	2.2343	2.0159
D43	E45	A60	3.913	3.9094	4.3285	4.3285	1.836	1.6573	4.8668	3.0531	1.1613	3.8978	4.4641
D43	E45	A90	2.075	1.9604	2.1863	2.1863	1.5714	1.2172	1.9223	2.1236	0.94364	1.7454	2.8506
D43	E45	A120	1.686	1.6812	1.7478	1.7478	1.3006	1.1648	1.8884	1.4671	0.84688	1.4659	2.1569
D43	E45	A135	1.495	1.4114	1.463	1.463	1.0557	1.031	1.4773	1.4851	0.78974	1.2732	1.76
D43	E45	A150	1.128	1.0224	1.2033	1.2033	0.73596	0.75774	1.2211	0.99999	0.67057	1.2136	1.109
D43	E45	A180	1.376	1.3523	1.7396	1.7396	0.62979	0.63819	1.3293	1.4556	0.60672	0.99632	2.3739
D43	E60	A0	2.512	2.5883	2.1442	2.1442	1.5798	1.5152	2.5976	2.3636	1.1275	2.3742	1.6788
D43	E60	A30	2.882	2.9738	2.3821	2.3821	1.8785	1.739	3.3487	2.5371	1.1701	2.6942	1.9636
D43	E60	A45	3.833	3.9028	3.9778	3.9778	2.1506	1.8211	5.1176	2.8412	1.329	3.9413	3.4942
D43	E60	A60	6.267	5.7853	5.6504	5.6504	3.04	2.5233	7.2675	5.2384	1.5082	5.5097	7.2624
D43	E60	A90	3.063	2.6113	2.6814	2.6814	2.4888	1.6835	2.8613	3.2773	1.2339	2.2678	3.2156
D43	E60	A120	2.876	2.8522	2.8216	2.8216	2.3962	1.8602	2.9066	2.7033	1.2619	2.4948	3.0107
D43	E60	A135	2.301	2.2672	2.3602	2.3602	1.9183	1.734	2.4598	2.0815	1.213	2.1221	2.1843
D43	E60	A150	2.304	2.2617	2.1355	2.1355	1.8302	1.737	2.4145	2.0235	1.1559	2.1459	2.2304
D43	E60	A180	1.848	1.797	1.8603	1.8603	1.3837	1.3781	2.2424	1.3876	1.0782	1.9194	1.5626
D43	E90	A0	5.525	2.8329	6.5372	6.5372	2.7777	1.1948	7.7939	3.6128	1.7577	4.4822	7.0127
D43	E-30	A0	1.006	1.0082	1.082	1.082	0.6494	0.60013	1.1101	0.89656	0.38159	0.8716	1.3808
D43	E-30	A30	1.238	1.2201	0.98942	0.98942	1.1784	0.88753	0.99745	1.4022	0.75008	0.83487	0.86207
D43	E-30	A45	1.174	1.1751	1.2303	1.2303	0.64449	0.65709	1.1861	1.0973	0.43194	0.97419	1.491
D43	E-30	A60	1.399	1.4116	1.6732	1.6732	0.81177	0.80728	1.6625	1.1704	0.53656	1.2593	2.2761
D43	E-30	A90	1.396	0.91769	1.455	1.455	0.92083	0.54326	1.2085	1.6295	0.61856	1.1821	1.6432
D43	E-30	A120	1.857	1.81	2.2331	2.2331	0.59521	0.5944	2.3363	1.4862	0.39774	1.8832	2.7054
D43	E-30	A135	1.557	1.5461	1.9897	1.9897	0.5801	0.5802	1.9543	1.2468	0.39682	1.6314	2.2524
D43	E-30	A150	1.105	1.0981	1.2476	1.2476	0.55692	0.56867	1.1838	1.0139	0.35612	1.0942	1.2939
D43	E-30	A180	1.006	1.0082	1.082	1.082	0.64959	0.60014	1.11	0.89638	0.38151	0.87158	1.3813
D43	E-45	A0	2.04	1.9618	2.3468	2.3468	1.1487	0.99112	1.3127	1.2046	0.72189	2.0244	2.4873
D43	E-45	A30	1.164	1.16	1.2249	1.2249	0.80878	0.75207	1.0582	1.0868	0.53591	1.0195	1.3737
D43	E-45	A45	1.443	1.4444	1.4881	1.4881	1.0012	0.89256	1.8211	1.1176	0.53475	1.2238	1.7071
D43	E-45	A60	2.115	2.0311	2.1076	2.1076	1.0237	0.91895	2.3227	1.7859	0.44671	1.6204	2.7911
D43	E-45	A90	1.839	1.1731	1.8294	1.8294	1.0354	0.6697	1.5635	2.0438	0.64308	1.5461	2.1698
D43	E-45	A120	2.218	2.1729	2.6053	2.6053	0.74381	0.74382	2.8825	1.6356	0.45745	2.2802	3.0951
D43	E-45	A135	2.03	2.0347	2.5834	2.5834	0.6318	0.62844	2.3054	1.7757	0.44918	2.1183	2.8756
D43	E-45	A150	1.468	1.44	1.8047	1.8047	0.65172	0.65149	1.4655	1.4593	0.40994	1.471	1.9003
D43	E-45	A180	1.307	1.2866	1.5205	1.5205	0.72307	0.68964	1.306	1.2726	0.44032	1.2078	1.8416
D43	E-60	A0	2.387	2.284	2.8767	2.8767	1.0717	0.99692	2.1158	1.2928	0.70703	2.3488	3.0619
D43	E-60	A30	1.469	1.4748	1.5849	1.5849	0.83201	0.83052	1.4874	1.2727	0.64631	1.2984	1.7783
D43	E-60	A45	1.375	1.3748	1.3027	1.3027	0.87388	0.86354	1.5649	1.1839	0.62596	1.2278	1.4168
D43	E-60	A60	1.8	1.7768	1.8307	1.8307	1.0949	1.0783	1.9835	1.4298	0.84621	1.6032	2.2402
D43	E-60	A90	2.858	1.8645	2.5402	2.5402	1.6994	1.3155	2.3321	2.8882	0.9035	2.2569	3.1952
D43	E-60	A120	1.8	1.7768	1.8309	1.8309	1.0949	1.0784	1.9834	1.4298	0.84614	1.6033	2.2403
D43	E-60	A135	2.312	2.2949	2.815	2.815	0.87183	0.86939	3.0187	1.6644	0.57655	2.4085	2.9098
D43	E-60	A150	1.804	1.7767	2.2083	2.2083	0.83823	0.82185	1.7694	1.8401	0.52979	1.8163	2.3417
D43	E-60	A180	1.631	1.564	1.845	1.845	1.0374	1.0265	1.3691	1.7993	0.57873	1.5525	2.0598
D43	E-90	A0	2.53	1.9928	2.5758	2.5758	2.0551	1.1633	2.2152	2.8441	1.3307	2.2359	3.184
D50	E0	A0	1.582	1.2967	1.3777	1.3777	1.2774	0.98724	1.4975	1.4484	0.93628	1.29	1.4904
D50	E30	A0	1.885	1.9408	1.4227	1.4227	1.8285	1.4793	1.747	1.945	1.3238	1.4612	1.1496
D50	E45	A0	1.589	1.5283	1.1867	1.1867	1.4343	1.1718	1.5787	1.51	1.0616	1.0749	1.3358
D50	E60	A0	2.837	2.9849	2.1216	2.1216	2.8009	1.9771	2.719	2.8108	1.7479	2.3512	1.5562
D50	E90	A0	6.009	2.5282	6.1327	6.1327	3.7446	0.9974	8.1464	3.8795	2.3945	4.3024	6.8282
D50	E-30	A0	2.078	1.8198	2.0983	2.0983	1.4943	1.0709	1.3811	1.5392	0.927	1.8612	2.1289
D50	E-30	A45	4.303	4.1308	3.9742	3.9742	1.0318	0.92771	3.6926	4.8401	0.68909	1.0575	6.2584
D50	E-30	A60	1.426	1.4649	1.795	1.795	0.73315	0.73193	1.5557	1.274	0.41273	1.3582	2.3539
D50	E-30	A90	1.231	0.92982	1.215	1.215	0.83434	0.57055	1.1146	1.3607	0.52536	1.0081	1.4361
D50	E-30	A120	2.108	2.0164	2.4471	2.4471	0.54064	0.53304	2.3412	1.7739	0.34002	2.0728	3.0173
D50	E-30	A135	2.664	2.5474	3.1456	3.1456	1.019	0.96974	3.173	2.2724	1.2677	2.7319	3.4187
D50	E-30	A180	1.128	1.1261	0.98298	0.98298	1.073	0.83026	1.2351	1.0637	0.70284	0.84895	1.164
D50	E-45	A0	2.511	2.2845	2.722	2.722	1.5299	1.2122	1.589	1.7054	0.97949	2.3346	2.8507
D50	E-45	A30	1.171	1.1425	1.156	1.156	0.88956	0.76549	1.0318	1.1781	0.5156	0.96611	1.3172
D50	E-45	A45	1.529	1.5722	1.7695	1.7695	0.79992	0.7581	1.8133	1.2952	0.3807	1.3648	2.2225
D50	E-45	A60	4.013	3.9605	4.1193	4.1193	0.93314	0.88128	4.0411	4.0269	0.66276	1.8311	4.8836
D50	E-45	A90	6.267	5.9021	7.5047	7.5047	2.3804	2.1879	4.4633	6.2052	1.4463	6.813	8.3728
D50	E-45	A120	4.377	4.3139	4.4441	4.4441	2.2769	2.2272	5.747	3.1541	1.333	4.5662	4.3463
D50	E-45	A135	2.351	2.2906	2.9396	2.9396	0.48389	0.44814	2.5103	2.19	0.34731	2.4949	3.2444
D50	E-45	A150	1.456	1.4549	1.7761	1.7761	0.66119	0.63533	1.4391	1.4738	0.41272	1.456	1.8648
D50	E-45	A180	1.362	1.3874	1.137	1.137	1.4373	1.0632	1.3266	1.4099	0.76317	0.88657	1.3903
D50	E-60	A0	2.462	2.2727	2.6727	2.6727	1.6047	1.2649	2.0727	1.6525	1.0913	2.1117	3.0576
D50	E-60	A30	1.554	1.5436	1.4233	1.4233	1.2582	1.1015	1.4442	1.4468	0.83327	1.2094	1.4571
D50	E-60	A45	1.43	1.4201	1.2428	1.2428	1.1153	0.97228	1.602	1.2908	0.68874	1.1324	1.2275
D50	E-60	A60	1.767	1.7506	1.7304	1.7304	1.1033	1.0521	2.0221	1.4626	0.80041	1.5347	1.966
D50	E-60	A90	2.831	1.8533	2.3891	2.3891	1.8044	1.3572	2.3631	2.7396	0.89443	2.1678	3.0484
D50	E-60	A120	2.722	2.7254	3.0722	3.0722	1.0169	0.97729	3.2069	1.886	0.65263	2.7676	3.4597
D50	E-60	A135	2.207	2.2351	2.6435	2.6435	1.1045	0.95855	2.8694	1.5657	0.67993	2.2348	2.7002
D50	E-60	A150	1.622	1.6675	1.7613	1.7613	1.3382	0.98336	1.6171	1.5883	0.75804	1.3977	1.9421
D50	E-60	A180	1.65	1.646	1.4165	1.4165	1.7074	1.1797	1.5191	1.6594	0.91474	1.1144	1.683
D52	E30	A-30	1.589	1.7165	1.0832	1.0832	1.9352	1.4416	1.4935	1.6623	1.3628	1.2563	0.95487

Table AB. Virtual Random combined errors, MSPE

Dist	El	Az	all	all stat	adults	adults stat	kids	kids stat	small	normal	big
D35	E0	A0	11.44	6.6131	NA	NA	NA	NA	NA	NA	NA
D35	E0	A0	11.44	6.6131	5.4696	4.9252	9.8911	4.4307	4.1368	4.9674	5.7986
D35	E0	A30	2.777	2.6299	1.6677	1.6532	2.1985	1.8767	0.84758	1.8668	1.8649
D35	E0	A45	5.568	5.4108	5.4309	3.0355	2.5428	2.3138	1.1355	4.6187	4.6583
D35	E0	A90	8.029	8.0269	10.1	10.101	2.3365	2.1278	1.2607	6.4442	12.586
D35	E0	A150	8.541	7.4962	9.474	5.3107	2.772	2.712	1.2627	7.548	10.626
D35	E0	A-60	10.35	9.8327	11.443	7.2436	3.7042	3.6408	1.6696	8.7665	13.392
D35	E0	A-90	14.97	14.703	19.285	18.483	3.2784	3.0541	1.001	10.297	27.284
D35	E0	A-135	4.641	3.792	5.6382	2.7732	1.5338	1.5218	0.69863	4.1992	5.7245
D37	E35.5	A30	4.657	4.6548	3.4228	2.8245	2.4584	2.2267	1.2923	4.397	2.1133
D37	E35.5	A60	27.88	27.359	30.144	21.666	8.0035	7.4053	2.706	23.889	35.429
D43	E0	A0	3.592	2.3815	2.8931	2.5554	2.4444	1.3503	1.1039	2.6878	3.2324
D43	E0	A30	2.14	1.8246	1.4561	1.4306	1.1613	0.80327	0.57466	1.2854	1.9142
D43	E0	A45	4.079	4.0228	4.2062	2.8611	1.3533	1.1528	0.28565	3.3264	4.148
D43	E0	A60	7.74	7.3629	9.2452	6.4807	2.3369	2.2618	0.26406	6.8617	10.714
D43	E0	A90	7.588	5.6943	7.3985	6.6575	3.8108	1.5296	0.48489	5.6396	8.6663
D43	E0	A120	6.271	5.4767	7.4826	4.6921	1.4146	1.3971	0.48181	5.785	8.4126
D43	E0	A135	3.146	2.6148	4.111	2.3287	0.66718	0.6614	0.50256	2.7415	4.6813
D43	E0	A150	1.558	1.4409	1.4777	1.4777	0.66911	0.52927	0.93525	1.3473	1.4799
D43	E0	A180	3.621	3.2008	4.0967	3.9665	0.97507	0.89196	1.3638	2.93	5.7685
D43	E30	A0	10.06	9.8996	13.069	12.954	2.6434	2.0878	1.2959	2.5415	20.664
D43	E30	A30	3.446	3.4466	2.478	2.203	1.8764	1.7087	1.1604	3.2617	1.7133
D43	E30	A45	12.46	11.55	14.105	8.9499	2.7945	2.7751	0.95233	12.27	12.486
D43	E30	A60	22.01	21.364	24.183	16.95	6.2644	5.9258	5.6055	19.326	27.321
D43	E30	A90	9.236	7.1592	10.706	8.9009	4.5742	1.7624	0.80219	7.2209	14.49
D43	E30	A120	7.478	6.929	8.2524	4.0425	3.099	2.8302	0.46362	5.2064	11.248
D43	E30	A135	6.513	6.1661	6.5401	2.7024	2.3372	2.2141	0.44135	4.5236	7.4891
D43	E30	A150	2.942	2.9416	2.4819	2.0431	0.90583	0.80454	0.47414	1.8871	2.9497
D43	E30	A180	17.71	17.387	20.865	18.794	1.2996	1.2559	1.0402	3.1409	32.288
D43	E45	A0	9.12	8.9799	8.0207	7.2734	4.4649	3.4145	1.4493	6.0028	8.6192
D43	E45	A30	9.622	9.6175	7.2292	5.4584	4.4519	3.9023	2.1593	9.1099	4.0359
D43	E45	A45	22.43	22.03	23.405	17.235	5.7616	5.3576	2.1286	22.764	18.195
D43	E45	A60	49.71	49.69	54.053	47.141	12.342	11.076	1.4467	44.251	66.365
D43	E45	A90	15.17	13.344	16.456	16.142	7.4411	4.2278	1.8058	10.138	25.407
D43	E45	A120	10.94	10.623	12.161	7.0583	5.5565	4.3726	2.0138	7.5171	17.049
D43	E45	A135	9.121	8.6957	9.2841	4.1207	4.218	3.8144	1.5922	6.0122	12.286
D43	E45	A150	5.022	4.6995	5.2906	3.2742	2.1033	2.0527	1.212	4.9732	4.4552
D43	E45	A180	9.286	9.1145	11.947	11.892	1.3837	1.3529	1.0098	3.9742	15.473
D43	E60	A0	20.91	20.494	16.107	13.671	8.6817	6.5475	1.8498	16.686	12.104
D43	E60	A30	31.02	30.36	23.828	20.261	11.981	8.6184	2.5151	26.488	17.172
D43	E60	A45	58.18	56.401	56.95	54.697	16.122	11.581	4.1646	52.762	55.992
D43	E60	A60	113.2	104.91	100.21	98.382	30.78	22.904	2.5508	83.38	156.28
D43	E60	A90	32.1	20.962	23.807	21.031	18.086	7.7762	1.4232	16.85	32.178
D43	E60	A120	25.96	25.781	25.199	18.873	17.244	10.341	3.354	18.593	31.165
D43	E60	A135	19.44	19.177	20.053	10.844	12.312	9.3203	4.4843	15.387	19.234
D43	E60	A150	19.67	19.582	18.225	11.349	10.826	8.7418	0.93273	15.863	18.856
D43	E60	A180	15.64	15.54	15.995	12.687	7.0347	5.9906	3.7836	14.945	13.922
D43	E90	A0	141.1	45.593	173.16	45.598	26.651	5.3421	0.63813	87.04	169.97
D43	E-30	A0	6.75	5.9729	6.7737	6.7672	3.1984	1.9539	3.3522	5.1495	8.6273
D43	E-30	A135	204.1	166.54	204.12	135.05	29.257	29.252	0.9168	37.589	135.78
D43	E-30	A180	3.756	3.7553	3.9493	3.6312	1.3113	1.1575	3.0281	2.6804	5.6855
D43	E-45	A0	10.23	9.8149	11.771	11.749	3.4154	2.8	0.89607	9.1636	14.229
D43	E-60	A0	18.89	18.656	23.857	23.672	3.2343	2.9582	3.6144	16.688	31.897
D43	E-60	A30	10.25	10.21	12.438	11.015	2.0544	2.0286	5.1546	8.2352	16.872
D43	E-60	A60	11.04	11.038	13.359	12.991	2.573	2.538	1.5925	9.8801	17.254
D43	E-60	A90	17.43	10.45	17.482	10.204	6.3003	5.0486	6.8493	11.732	23.608
D43	E-60	A135	24.68	24.27	28.758	27.234	2.5376	2.5154	2.5817	24.504	33.559
D43	E-60	A150	14.18	14.039	16.576	14.997	2.1114	2.0335	4.4473	12.813	19.816
D43	E-60	A180	9.928	9.5423	11.301	8.2901	3.3794	3.2363	9.7534	8.3533	13.148
D50	E0	A0	7.884	4.3941	5.2173	4.2755	5.3546	2.5127	2.5437	4.472	5.8745
D50	E30	A0	14.75	11.837	6.1664	5.879	11.799	5.9329	2.4758	6.1295	4.2093
D50	E45	A0	9.51	6.7355	3.9138	3.9048	7.3701	3.7276	4.544	3.3284	4.7195
D50	E60	A0	31.3	26.026	14.756	13.072	24.612	10.88	2.5663	15.33	10.236
D50	E90	A0	135.1	34.325	148.16	40.599	41.474	3.5231	2.9606	75.047	156.24
D50	E-30	A0	11.61	8.1591	8.7971	8.3946	6.6113	3.2457	5.5427	7.0567	10.354
D50	E-45	A0	16.09	13.262	14.866	14.747	7.2141	4.3535	0.46227	11.506	18.016
D50	E-45	A30	5.128	4.9362	4.6427	4.3518	2.4964	1.8705	0.43269	3.3057	6.0112
D50	E-45	A45	9.202	9.1378	11.479	9.9472	2.1023	2.0584	1.5375	7.7675	15.478
D50	E-45	A60	59.78	59.625	54.935	53.141	3.1835	2.5611	0.7266	12.886	73.156
D50	E-45	A90	125	109.31	152.27	125.31	20.153	18.614	0.65801	118.95	198.09
D50	E-45	A120	59.73	59.351	57.861	57.377	12.757	12.597	1.5123	60.621	56.512
D50	E-45	A135	18.63	18.067	22.7	22	0.97935	0.82908	8.2769	18.445	27.365
D50	E-45	A150	7.572	7.5727	8.3384	7.9025	1.4207	1.1474	3.133	6.8043	8.9727
D50	E-45	A180	7.852	7.0936	4.4923	3.9859	6.1712	3.0538	7.8596	2.8931	6.1986
D50	E-60	A0	19.77	17.827	19.797	19.744	8.0835	4.8759	1.2785	13.269	28.068
D50	E-60	A30	10.09	9.7411	8.5085	7.4439	5.3783	3.641	1.3632	5.9149	10.685
D50	E-60	A45	8.5	8.3066	7.673	6.8915	4.2442	2.9182	1.5422	5.5609	9.4868
D50	E-60	A60	9.855	9.526	9.6113	9.4235	4.1516	3.2038	2.1941	7.3405	12.13
D50	E-60	A90	20.91	9.758	17.933	9.3573	8.751	4.7786	1.8239	11.513	24.123
D50	E-60	A120	22.75	22.754	23.693	22.863	3.7533	3.5265	1.2945	20.568	30.553
D50	E-60	A135	19.72	19.639	21.33	20.507	4.2322	3.2732	1.652	17.129	25.335
D50	E-60	A150	12.59	11.861	10.457	9.838	5.749	3.0807	2.4889	7.5229	13.079
D50	E-60	A180	11.15	10.129	6.5151	5.2301	8.542	4.3123	16.796	4.3865	8.3215
D52	E30	A-30	10.91	8.304	3.0893	3.0456	12.316	5.5111	4.769	3.7794	2.2188

Table AC. Virtual NHANES combined errors, MAPE

Dist	El	Az	all	all stat	adults	adults stat	kids	kids stat	females	males	small	normal	big
D43	E0	A0	1.961	1.588	1.885	1.7655	1.3909	1.2157	1.9533	1.6874	1.1018	1.818	1.5324
D43	E0	A30	1.199	1.0924	1.1391	1.1397	0.91471	0.85317	0.95478	1.4083	0.75175	1.1088	1.1495
D43	E0	A45	1.423	1.4026	1.759	1.4563	0.81044	0.81218	1.3722	1.3873	0.63737	1.5962	1.5856
D43	E0	A60	2.304	2.1377	2.8414	2.1646	1.2362	1.2442	2.1527	2.3585	0.93984	2.6517	2.49
D43	E0	A90	2.562	2.0204	2.5514	2.4638	1.4446	1.1465	2.7046	2.4201	1.0798	2.4203	2.5323
D43	E0	A120	1.882	1.6795	2.2439	1.826	1.0992	1.0982	1.657	2.0397	0.84172	2.0953	1.9535
D43	E0	A135	1.15	1.0591	1.4396	1.1204	0.64002	0.6227	1.0341	1.2492	0.55555	1.32	1.8638
D43	E0	A150	0.9585	0.93435	1.0602	1.0571	0.58182	0.57024	0.86548	1.0022	0.4469	1.0065	1.0351
D43	E0	A180	1.455	1.3677	1.6775	1.6711	0.84035	0.81084	1.4018	1.4422	0.67232	1.5835	1.7095
D43	E30	A0	1.619	1.3957	1.4126	1.4147	1.1254	1.0055	1.627	1.5745	0.89737	1.3551	1.4123
D43	E30	A30	1.261	1.2808	1.4455	1.3881	0.91164	0.90763	1.0745	1.4015	0.77975	1.3413	1.0084
D43	E30	A45	2.144	1.9422	2.7054	2.0758	1.1446	1.1424	2.1106	2.1249	0.94382	2.4763	2.4974
D43	E30	A60	4.038	3.7447	5.2364	4.2065	1.9595	1.9924	3.8589	4.0355	1.4176	4.7756	4.5146
D43	E30	A90	2.976	2.5262	3.277	3.1839	1.8681	1.3928	2.9809	2.6686	1.4651	3.093	2.5781
D43	E30	A120	2.267	2.1446	2.7759	1.8199	1.3442	1.3522	2.1841	2.3359	0.93977	2.5827	2.5084
D43	E30	A135	1.799	1.6144	2.1797	1.1885	1.0876	1.0655	1.6438	1.9458	0.8339	2.038	1.6154
D43	E30	A150	0.9739	0.93861	1.1595	0.95303	0.66488	0.64218	0.84119	1.0811	0.60147	1.0714	1.4678
D43	E30	A180	1.312	1.2631	1.5136	1.514	0.75682	0.75156	1.1507	1.4219	0.65751	1.4353	2.0874
D43	E45	A0	1.97	1.9856	2.1922	2.0384	1.3083	1.2749	1.727	2.1235	1.0865	2.0216	2.2044
D43	E45	A30	1.931	1.9443	2.3076	2.1282	1.2882	1.2761	1.8976	1.9285	1.0519	2.0995	1.9023
D43	E45	A45	2.961	2.8336	3.7229	3.1377	1.6418	1.6682	2.972	2.8859	1.3115	3.3981	3.1728
D43	E45	A60	4.986	4.8346	6.6078	6.0686	2.2734	2.3049	5.014	4.7076	1.6897	5.9205	6.815
D43	E45	A90	3.583	3.1832	3.8819	3.8679	2.349	1.846	3.5225	3.4846	1.7587	3.6464	3.8595
D43	E45	A120	2.577	2.5831	2.9663	2.2922	1.7859	1.6737	2.3851	2.6473	1.268	2.7829	3.0453
D43	E45	A135	2.133	1.9999	2.552	1.5525	1.3671	1.3743	2.028	2.2333	1.0368	2.3795	2.4253
D43	E45	A150	1.785	1.6235	2.1436	1.5559	1.1621	1.1653	1.7174	1.8285	0.93032	1.9868	1.3417
D43	E45	A180	1.5	1.5386	1.8463	1.7929	0.9312	0.93623	1.5077	1.5122	0.82956	1.7048	1.8756
D43	E60	A0	2.927	2.9413	3.3946	3.1111	2.0146	1.9226	2.9822	2.7666	1.6619	3.1365	3.0858
D43	E60	A30	3.457	3.4738	4.1747	3.8634	2.1173	2.0298	3.5751	3.2262	1.682	3.7955	3.489
D43	E60	A45	4.436	4.4558	5.5971	5.3016	2.4065	2.3511	4.4785	4.0504	1.847	5.0623	5.1225
D43	E60	A60	5.322	5.3439	6.9003	6.6368	2.5712	2.5619	5.1169	4.8386	1.9525	6.202	6.9731
D43	E60	A90	5.102	3.7315	4.7192	4.5168	2.9791	2.0181	5.4631	4.7333	2.0672	4.448	4.7203
D43	E60	A120	4.363	4.3591	4.7387	3.9287	2.9248	2.3883	3.9486	4.3497	1.8106	4.483	4.8703
D43	E60	A135	3.713	3.7885	4.1708	3.2662	2.43	2.1868	3.4494	3.6239	1.5891	3.8952	3.782
D43	E60	A150	3.311	3.399	3.93	3.2342	2.0013	1.9065	2.8857	3.4201	1.3921	3.6038	3.5394
D43	E60	A180	2.956	3.0199	3.6413	3.3633	1.7761	1.725	2.7703	2.9948	1.3171	3.3492	3.7696
D43	E90	A0	8.173	3.048	7.4535	3.2109	4.591	1.4991	8.3213	8.0283	3.7082	7.3487	5.2778
D43	E-30	A0	2.083	1.9854	2.307	2.307	1.4071	1.3048	1.583	1.4888	1.1295	2.1757	1.1177
D43	E-30	A30	1.297	1.2917	1.3142	1.1856	0.91048	0.88963	1.149	1.2865	0.71878	1.2206	1.2725
D43	E-30	A45	1.612	1.6249	1.8997	1.6267	0.90345	0.8702	1.7517	1.429	0.71514	1.7569	1.595
D43	E-30	A60	2.038	2.0059	2.5034	2.1042	1.0956	1.0789	2.2284	1.7102	0.80841	2.3113	1.3669
D43	E-30	A90	2.367	1.6186	2.4966	1.9845	1.2186	0.90466	2.4434	2.3695	0.96322	2.3356	2.9191
D43	E-30	A120	2.564	2.156	2.9877	2.8001	1.0964	1.0152	2.7056	2.4052	0.86345	2.7261	2.6247
D43	E-30	A135	1.881	1.7825	2.392	2.3012	0.84804	0.84726	1.7651	1.8866	0.68441	2.182	2.4765
D43	E-30	A150	1.288	1.3091	1.6532	1.6375	0.68399	0.67975	1.203	1.3242	0.538	1.509	1.4041
D43	E-30	A180	1.304	1.3138	1.5325	1.4405	0.78521	0.76255	1.3701	1.1754	0.61381	1.43	1.394
D43	E-45	A0	2.476	2.4781	2.9102	2.8338	1.4975	1.4866	1.9109	1.8311	1.184	2.7647	2.3306
D43	E-45	A30	1.868	1.8969	2.1676	1.9772	1.1365	1.1436	1.9144	1.6549	0.93997	1.9919	1.7644
D43	E-45	A45	2.09	2.1076	2.4772	2.0346	1.2574	1.2458	2.3514	1.7706	0.92303	2.2805	1.616
D43	E-45	A60	2.766	2.803	3.1685	2.4699	1.3541	1.3521	2.7016	2.5222	0.82801	2.9137	2.0512
D43	E-45	A90	2.872	1.9359	2.6207	2.2493	1.5726	1.2534	2.9209	2.8329	1.1841	2.5466	3.0435
D43	E-45	A120	2.969	2.6712	3.7168	3.4401	1.3171	1.265	2.9432	2.9565	1.0569	3.3787	3.5862
D43	E-45	A135	2.421	2.3946	3.151	3.0433	1.1786	1.185	2.0623	2.7042	0.92003	2.8774	3.5629
D43	E-45	A150	1.827	1.8345	2.3898	2.3016	0.89026	0.89558	1.5262	2.0692	0.66505	2.1629	2.6365
D43	E-45	A180	1.673	1.6794	2.1423	1.9708	0.86459	0.87105	1.5342	1.7405	0.629	1.936	2.4072
D43	E-60	A0	2.95	2.9781	3.5964	3.4195	1.6415	1.6496	2.9512	2.1298	1.3076	3.3363	2.2327
D43	E-60	A30	2.406	2.435	2.8948	2.5455	1.3767	1.3836	2.6458	2.0547	1.1483	2.6471	2.0018
D43	E-60	A45	2.217	2.2526	2.5441	2.193	1.4199	1.4226	2.2739	2.0835	1.1743	2.3404	2.453
D43	E-60	A60	2.491	2.4993	2.759	2.5827	1.5793	1.5801	2.2696	2.5065	1.3215	2.5828	3.7361
D43	E-60	A90	3.538	2.2368	3.1685	2.4768	1.9383	1.5863	3.2038	3.4759	1.4815	3.1953	3.5015
D43	E-60	A120	3.328	3.2222	4.245	3.8764	1.765	1.7577	3.0775	3.4044	1.4417	3.9152	4.2791
D43	E-60	A135	3.121	3.0978	4.051	3.8616	1.5917	1.5986	2.8545	3.2854	1.2254	3.7217	4.5122
D43	E-60	A150	3.327	3.2219	4.2448	3.8762	1.7653	1.7583	3.0773	3.4038	1.4422	3.9152	4.2295
D43	E-60	A180	2.374	2.3644	2.9934	2.6618	1.3253	1.3357	1.7421	2.9023	0.94046	2.7731	4.4024
D43	E-90	A0	4.524	3.2922	4.179	3.7829	2.9841	2.2475	4.0065	4.805	2.3862	4.2601	3.7923

Table AD. Virtual NHANES combined errors, RMSE

Dist	El	Az	all	all stat	adults	adults stat	kids	kids stat	females	males	small	normal	big
D43	E0	A0	1.502	1.152	1.4476	1.4476	1.0142	0.83924	1.4897	1.2719	0.76552	1.3865	1.2435
D43	E0	A30	0.8903	0.78001	0.86027	0.86027	0.65355	0.58557	0.72797	1.086	0.5159	0.82689	0.8749
D43	E0	A45	0.9566	0.97518	1.2256	1.2256	0.56333	0.57411	0.87773	0.95901	0.42819	1.1014	1.2068
D43	E0	A60	1.677	1.5906	2.1858	2.1858	0.9055	0.96985	1.5839	1.6926	0.67204	2.0271	1.8883
D43	E0	A90	1.943	1.3973	1.8483	1.8483	1.0271	0.77681	2.0529	1.8219	0.75269	1.7574	1.9253
D43	E0	A120	1.296	1.204	1.5988	1.5988	0.7805	0.807	1.1852	1.3804	0.60943	1.488	1.4594
D43	E0	A135	0.748	0.71912	0.95344	0.95344	0.45224	0.45122	0.67562	0.80714	0.40419	0.88096	1.2712
D43	E0	A150	0.6925	0.65877	0.78122	0.78122	0.40587	0.39152	0.61646	0.73771	0.29679	0.73461	0.74615
D43	E0	A180	1.034	0.95914	1.2614	1.2614	0.58102	0.56687	0.95427	1.0545	0.45657	1.1772	1.1721
D43	E30	A0	1.213	0.98246	1.0161	1.0161	0.78758	0.6741	1.2163	1.1847	0.5961	0.97208	1.0959
D43	E30	A30	0.8498	0.88139	0.99363	0.99363	0.61887	0.61422	0.70582	0.98539	0.52057	0.91471	0.67297
D43	E30	A45	1.397	1.2782	1.8118	1.8118	0.76201	0.79419	1.3953	1.3579	0.59879	1.6684	1.6658
D43	E30	A60	2.759	2.6454	3.8924	3.8924	1.282	1.4302	2.6675	2.6793	0.90857	3.4983	3.4364
D43	E30	A90	2.184	1.7141	2.3635	2.3635	1.4581	0.98195	2.0583	1.9475	1.0896	2.2311	2.0614
D43	E30	A120	1.536	1.5882	2.0105	2.0105	0.98995	1.0074	1.6171	1.4453	0.69633	1.8461	2.0045
D43	E30	A135	1.185	1.1283	1.4866	1.4866	0.76381	0.77521	1.0988	1.2833	0.58427	1.3871	1.2839
D43	E30	A150	0.6173	0.62369	0.75048	0.75048	0.45355	0.43879	0.53415	0.707	0.40283	0.69521	1.1026
D43	E30	A180	0.8974	0.86158	1.0872	1.0872	0.52427	0.52052	0.75472	1.016	0.44639	1.0284	1.6026
D43	E45	A0	1.366	1.3854	1.5251	1.5251	0.88889	0.84976	1.1674	1.5017	0.71735	1.396	1.4996
D43	E45	A30	1.246	1.2617	1.4781	1.4781	0.90746	0.89692	1.1775	1.2891	0.75033	1.3444	1.3618
D43	E45	A45	1.931	1.8423	2.4729	2.4729	1.1318	1.1635	1.91	1.9106	0.8979	2.2607	2.0295
D43	E45	A60	3.294	3.2122	4.8131	4.8131	1.5468	1.6288	3.4189	3.0863	1.1205	4.1873	4.9788
D43	E45	A90	2.529	2.1741	2.7486	2.7486	1.7684	1.2976	2.3548	2.5229	1.3143	2.5657	2.8235
D43	E45	A120	1.818	1.9066	2.0751	2.0751	1.3948	1.2628	1.8188	1.6597	0.96895	1.9438	2.3127
D43	E45	A135	1.424	1.4441	1.7526	1.7526	0.98375	0.99753	1.4145	1.4366	0.75577	1.6354	1.8446
D43	E45	A150	1.182	1.0504	1.4333	1.4333	0.78953	0.80731	1.1191	1.2647	0.63562	1.3184	0.93597
D43	E45	A180	0.9317	1.0172	1.1879	1.1879	0.61469	0.62453	0.89146	1.0162	0.54551	1.0864	1.378
D43	E60	A0	2.015	2.0357	2.3255	2.3255	1.4907	1.3928	1.8732	2.0089	1.2268	2.1501	2.4919
D43	E60	A30	2.196	2.2092	2.631	2.631	1.5298	1.4457	2.0962	2.215	1.2541	2.3845	2.4481
D43	E60	A45	2.893	2.9301	3.7827	3.7827	1.7022	1.6354	2.7824	2.76	1.3249	3.3708	3.6019
D43	E60	A60	3.623	3.6758	5.0742	5.0742	1.7411	1.7232	3.5438	3.3372	1.2662	4.5025	4.9364
D43	E60	A90	3.72	2.5922	3.3897	3.3897	2.2242	1.3539	3.8895	3.5511	1.5326	3.1681	3.2157
D43	E60	A120	3.402	3.3947	3.6298	3.6298	2.2435	1.6532	3.1118	3.1921	1.3847	3.4112	4.0593
D43	E60	A135	2.701	2.834	2.9792	2.9792	1.838	1.5777	2.5345	2.5193	1.2184	2.7731	2.8575
D43	E60	A150	2.236	2.343	2.6653	2.6653	1.4332	1.3382	1.8744	2.3677	1.0625	2.4255	2.4393
D43	E60	A180	1.843	1.8964	2.2924	2.2924	1.2369	1.1892	1.7216	1.8696	0.9672	2.1089	2.3926
D43	E90	A0	6.136	2.1093	5.453	5.453	3.3729	1.0378	6.342	5.9425	2.7952	5.5988	4.3744
D43	E-30	A0	1.586	1.485	1.8365	1.8365	1.0517	0.92042	1.2233	1.1032	0.81469	1.7178	0.77068
D43	E-30	A30	0.9425	0.93573	0.93242	0.93242	0.66851	0.63202	0.81641	0.95925	0.49566	0.85637	0.9052
D43	E-30	A45	1.108	1.1706	1.3207	1.3207	0.64848	0.64134	1.1495	1.0073	0.49546	1.2031	1.2166
D43	E-30	A60	1.407	1.4602	1.8192	1.8192	0.79966	0.81892	1.5234	1.1987	0.55699	1.639	1.0031
D43	E-30	A90	1.635	1.0697	1.6987	1.6987	0.85851	0.59778	1.7159	1.6899	0.69253	1.5802	2.1742
D43	E-30	A120	1.819	1.4744	2.159	2.159	0.70106	0.71273	1.9345	1.698	0.54751	1.9415	1.9592
D43	E-30	A135	1.252	1.1861	1.6457	1.6457	0.5505	0.58512	1.1843	1.2195	0.43006	1.5024	1.6531
D43	E-30	A150	0.8421	0.87268	1.1479	1.1479	0.48454	0.47558	0.74637	0.89027	0.37434	1.0402	1.003
D43	E-30	A180	0.9135	0.93245	1.1022	1.1022	0.57581	0.54882	0.95448	0.88423	0.44772	1.0289	1.0903
D43	E-45	A0	1.768	1.7758	2.151	2.151	1.0883	1.0637	1.2572	1.3718	0.826	2.021	1.8052
D43	E-45	A30	1.228	1.2923	1.4066	1.4066	0.79552	0.81378	1.0952	1.172	0.62858	1.2754	1.302
D43	E-45	A45	1.348	1.4426	1.5897	1.5897	0.88805	0.91561	1.3816	1.2263	0.61346	1.4428	1.1907
D43	E-45	A60	2.073	2.1616	2.3664	2.3664	1.0006	1.0113	1.8714	1.9086	0.57312	2.1557	1.4957
D43	E-45	A90	2.113	1.3441	1.8341	1.8341	1.1245	0.84593	2.162	2.0721	0.82463	1.7843	2.3046
D43	E-45	A120	2.074	1.8098	2.7179	2.7179	0.81896	0.83504	2.0859	2.0232	0.65299	2.4331	2.6886
D43	E-45	A135	1.621	1.6306	2.1829	2.1829	0.74949	0.79523	1.365	1.7952	0.5636	1.9854	2.4025
D43	E-45	A150	1.174	1.2208	1.6143	1.6143	0.59709	0.61286	0.92875	1.3872	0.42599	1.4533	1.8554
D43	E-45	A180	1.076	1.1415	1.452	1.452	0.61728	0.63034	0.98396	1.1609	0.45094	1.3105	1.7526
D43	E-60	A0	1.909	2.0128	2.4407	2.4407	1.1468	1.1688	1.6866	1.6014	0.89185	2.2096	1.606
D43	E-60	A30	1.572	1.655	1.9203	1.9203	0.95805	0.98688	1.5143	1.5255	0.78066	1.7221	1.5285
D43	E-60	A45	1.494	1.5724	1.7217	1.7217	0.98939	1.0081	1.3638	1.4948	0.75656	1.559	1.95
D43	E-60	A60	1.819	1.8352	2.0244	2.0244	1.0925	1.0938	1.4966	1.8465	0.85803	1.8905	3.0376
D43	E-60	A90	2.679	1.5816	2.3525	2.3525	1.3765	1.0463	2.2843	2.5716	0.96536	2.4297	2.5588
D43	E-60	A120	2.38	2.2831	3.2273	3.2273	1.0943	1.1397	2.2288	2.3426	0.8473	2.9107	3.1397
D43	E-60	A135	2.121	2.1206	2.919	2.919	0.98326	1.0404	1.951	2.2102	0.72585	2.6335	3.1988
D43	E-60	A150	2.379	2.283	3.2271	3.2271	1.0944	1.1401	2.2285	2.3423	0.84786	2.9107	3.1123
D43	E-60	A180	1.484	1.549	1.9583	1.9583	0.83008	0.85561	1.0382	1.9631	0.57616	1.8248	3.4128
D43	E-90	A0	3.113	2.2895	3.1052	3.1052	2.1463	1.4966	2.715	3.3475	1.6334	3.1656	2.6007

Table AE. Virtual NHANES combined errors, MSPE

Dist	El	Az	all	all stat	adults	adults stat	kids	kids stat	females	males	small	normal	big
D43	E0	A0	6.104	4.1867	5.514	4.9599	3.2367	2.5599	6.0663	4.4832	2.1527	5.1945	3.5444
D43	E0	A30	2.355	2.0561	2.0778	2.0752	1.4304	1.3172	1.4373	3.0499	1.0045	2.0026	2.2412
D43	E0	A45	4.108	3.6426	5.8215	3.6299	1.1949	1.154	3.8616	3.9884	0.78486	4.9235	4.1944
D43	E0	A60	8.995	7.3275	12.396	7.6625	2.5507	2.3593	7.6447	9.6953	1.5329	10.944	9.2123
D43	E0	A90	11.1	7.3239	11.324	10.011	3.7184	2.4358	12.736	9.5205	2.2302	10.163	10.191
D43	E0	A120	6.921	5.0624	9.1166	5.8785	2.0888	1.9963	4.9309	8.4709	1.1921	8.0007	6.1786
D43	E0	A135	3.311	2.4041	4.6186	2.7544	0.72682	0.6644	2.3077	4.2254	0.52313	3.9457	7.4354
D43	E0	A150	1.561	1.5382	1.917	1.8443	0.60674	0.59145	1.3269	1.6667	0.38416	1.7394	2.1273
D43	E0	A180	3.572	3.2847	4.4694	4.442	1.2664	1.1728	3.4764	3.4691	0.86046	4.049	5.3299
D43	E30	A0	4.269	3.4691	3.5111	3.5112	2.1908	1.8623	4.3209	4.0224	1.5042	3.2404	3.1671
D43	E30	A30	3.27	3.1726	4.2629	3.334	1.5496	1.5462	2.5475	3.8129	1.161	3.6892	2.2556
D43	E30	A45	10.82	7.9029	15.323	8.3437	2.6397	2.3554	9.5604	11.755	1.8292	13.039	13.27
D43	E30	A60	31.58	24.952	45.28	28.612	7.9855	7.117	26.533	33.867	4.2348	39.022	33.508
D43	E30	A90	15.39	11.783	18.428	16.819	5.4627	3.372	17.601	12.153	3.667	16.388	10.262
D43	E30	A120	9.889	7.5941	13.364	5.2854	3.0361	3.0233	7.6402	11.908	1.4459	11.851	9.3056
D43	E30	A135	6.764	4.7248	9.2243	2.5203	2.0842	1.9052	5.5943	7.8923	1.2216	8.1067	4.3686
D43	E30	A150	2.077	1.7536	2.8357	1.5733	0.82972	0.78366	1.6086	2.4142	0.69638	2.4185	3.8062
D43	E30	A180	3.148	3.0281	4.032	4.0335	1.0289	1.0219	2.6538	3.4366	0.8008	3.6226	6.4989
D43	E45	A0	7.534	7.5167	9.5031	7.5791	3.1514	3.0747	6.2685	8.3335	2.242	8.1977	9.8883
D43	E45	A30	9.353	8.7729	13.179	9.4743	2.9704	2.9569	9.9851	8.1571	1.9821	11.124	7.3702
D43	E45	A45	20.24	17.102	29.088	18.757	5.2122	5.135	20.343	19.451	3.2644	24.828	21.395
D43	E45	A60	49.91	45.474	74.112	60.52	9.8888	9.5363	46.217	46.67	5.5483	62.802	78.431
D43	E45	A90	23.1	18.518	26.179	25.343	8.9466	6.0221	24.601	20.879	5.2576	23.334	25.778
D43	E45	A120	12.8	11.769	16.811	8.8797	4.8887	4.5611	8.8478	15.975	2.5047	14.978	15.294
D43	E45	A135	9.319	6.9265	12.466	4.0908	3.1767	3.1637	7.6195	10.935	1.774	10.971	9.9715
D43	E45	A150	7.234	5.5739	9.9536	4.8115	2.5539	2.4823	7.0266	6.9461	1.5983	8.7318	3.5016
D43	E45	A180	5.645	5.2145	8.0179	6.1159	1.747	1.7345	5.9979	5.1645	1.3499	6.9451	6.1226
D43	E60	A0	19.18	18.978	26.177	21.276	6.7203	6.3975	23.956	13.344	4.5471	22.503	15.828
D43	E60	A30	31.03	30.722	44.259	36.444	7.6444	7.399	37.711	21.069	4.7036	37.477	25.595
D43	E60	A45	43.97	43.533	63.155	53	10.509	10.402	46.703	34.325	5.9742	53.929	49.36
D43	E60	A60	54.06	53.748	77.495	68.009	12.729	12.735	47.895	46.946	7.4095	65.539	90.714
D43	E60	A90	44.67	25.159	38.111	33.753	14.757	7.5681	53.287	36.238	7.4768	34.422	39.589
D43	E60	A120	29.93	29.946	35.841	23.145	13.486	10.415	23.267	33.188	5.2258	32.31	34.606
D43	E60	A135	24.79	24.252	32.127	16.419	9.419	8.3428	20.302	25.975	3.9187	28.284	24.237
D43	E60	A150	23.76	22.774	33.078	19.548	6.8807	6.5957	18.585	25.117	3.0543	28.499	27.15
D43	E60	A180	22.14	21.132	31.898	23.684	5.8176	5.7311	17.985	24.188	2.9289	27.445	35.405
D43	E90	A0	112.3	16.792	90.589	18.083	36.616	4.1483	111.46	113.31	23.26	85.231	38.674
D43	E-30	A0	6.765	6.2003	7.5698	7.5716	3.2036	2.8859	3.8967	3.5775	2.1281	6.8718	2.3107
D43	E-30	A30	2.868	2.865	3.0774	2.3497	1.4199	1.3878	2.4627	2.6945	0.8977	2.7047	2.9367
D43	E-30	A45	5.197	4.89	7.1208	4.8124	1.417	1.2825	6.659	3.652	0.91288	6.231	4.2204
D43	E-30	A60	7.563	6.8635	10.573	7.4877	2.0082	1.8669	8.9847	5.4016	1.1508	9.3408	3.1568
D43	E-30	A90	10.55	5.0417	11.439	6.7942	2.8548	1.6324	11.175	9.6401	1.7908	10.215	13.285
D43	E-30	A120	11.8	8.9176	15.452	12.974	2.554	1.8744	12.609	10.942	1.4904	13.334	13.194
D43	E-30	A135	8.078	6.7932	11.464	9.8088	1.4275	1.3235	6.491	9.0488	0.93101	9.7371	13.84
D43	E-30	A150	3.654	3.5679	5.3839	4.8811	0.80685	0.80347	3.4688	3.7282	0.51289	4.5756	4.1689
D43	E-30	A180	3.247	3.2244	4.4287	3.7013	1.0081	0.98041	3.6948	2.2272	0.635	3.8331	3.0653
D43	E-45	A0	10.46	10.461	13.793	12.878	3.7169	3.6955	8.1009	5.4323	2.3779	12.385	8.9512
D43	E-45	A30	7.855	7.559	10.746	7.9552	2.3809	2.3592	10.302	4.8984	1.6299	9.3136	5.4836
D43	E-45	A45	9.843	8.9858	13.677	8.5139	2.82	2.6551	13.457	5.9905	1.5586	11.926	4.9426
D43	E-45	A60	12.47	12.248	16.258	9.8789	3.0434	3.0196	12.954	10.253	1.1897	14.125	7.1814
D43	E-45	A90	14.14	6.7513	12.165	8.6807	4.5718	2.9382	14.825	13.403	2.7705	11.466	14.568
D43	E-45	A120	17.04	13.978	23.88	19.437	3.7894	3.2174	15.002	18.762	2.3871	20.599	24.395
D43	E-45	A135	13.29	11.648	19.317	15.912	2.8514	2.7352	9.2055	16.942	1.7435	16.448	29.01
D43	E-45	A150	7.897	7.0812	11.729	9.3867	1.4968	1.478	6.0738	9.4822	0.87079	9.8127	15.029
D43	E-45	A180	6.125	5.5437	9.0245	6.6696	1.2753	1.2663	5.3083	6.2348	0.68345	7.4691	10.901
D43	E-60	A0	17.74	17.365	24.993	21.746	4.7534	4.7374	22.711	7.4428	3.1264	21.957	8.215
D43	E-60	A30	12.89	12.307	18.142	13.626	3.4958	3.452	18.433	7.0021	2.4457	15.702	6.4526
D43	E-60	A45	10.19	9.7868	13.556	9.3808	3.7736	3.7263	12.271	7.5445	2.7462	11.698	8.8378
D43	E-60	A60	10.5	10.493	12.872	10.748	4.6061	4.6078	9.794	10.384	3.5037	11.336	18.818
D43	E-60	A90	19.5	8.8829	16.154	10.552	6.7537	4.791	17.428	19.689	4.4544	15.943	20.493
D43	E-60	A120	20.31	18.476	28.2	22.648	6.739	6.3759	16.329	22.483	4.5668	24.959	34.228
D43	E-60	A135	20.31	18.632	29.247	24.162	5.4865	5.3036	16.331	23.374	3.2859	25.487	42.121
D43	E-60	A150	20.31	18.472	28.199	22.646	6.7409	6.3805	16.327	22.472	4.5679	24.959	33.14
D43	E-60	A180	13.01	11.532	18.621	12.916	3.6596	3.6191	8.6925	16.702	1.9015	16.034	32.483
D43	E-90	A0	38.67	19.432	29.37	23.345	15.533	9.8237	33.939	41.28	10.778	30.086	27.423
