# Supplementary figures and images for: A Framework for Analyzing the Whole Body Surface Area from a Single View
Source: PLoS One. 2017 Jan 3;12(1):e0166749. doi: 10.1371/journal.pone.0166749 (PMC5207503; doi:10.1371/journal.pone.0166749)

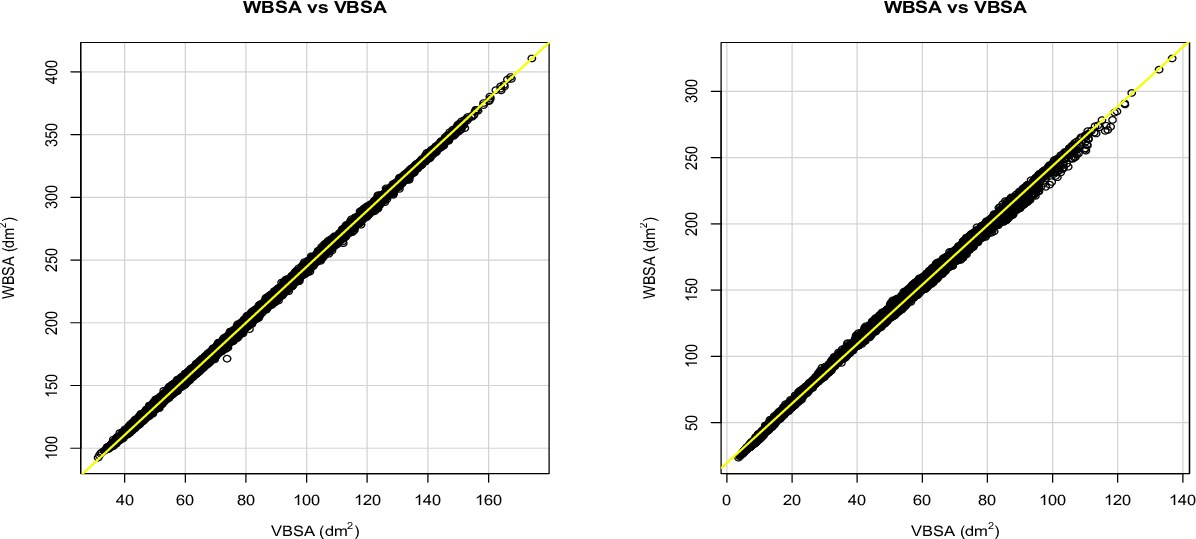

Supplement: S1 Fig — (Left) Using Virtual Random dataset at θ = 0°, ϕ = 0°. (Right) Using Virtual NHANES dataset at θ = 0°, ϕ = 0°. (TIF) [file pone.0166749.s002.tif]

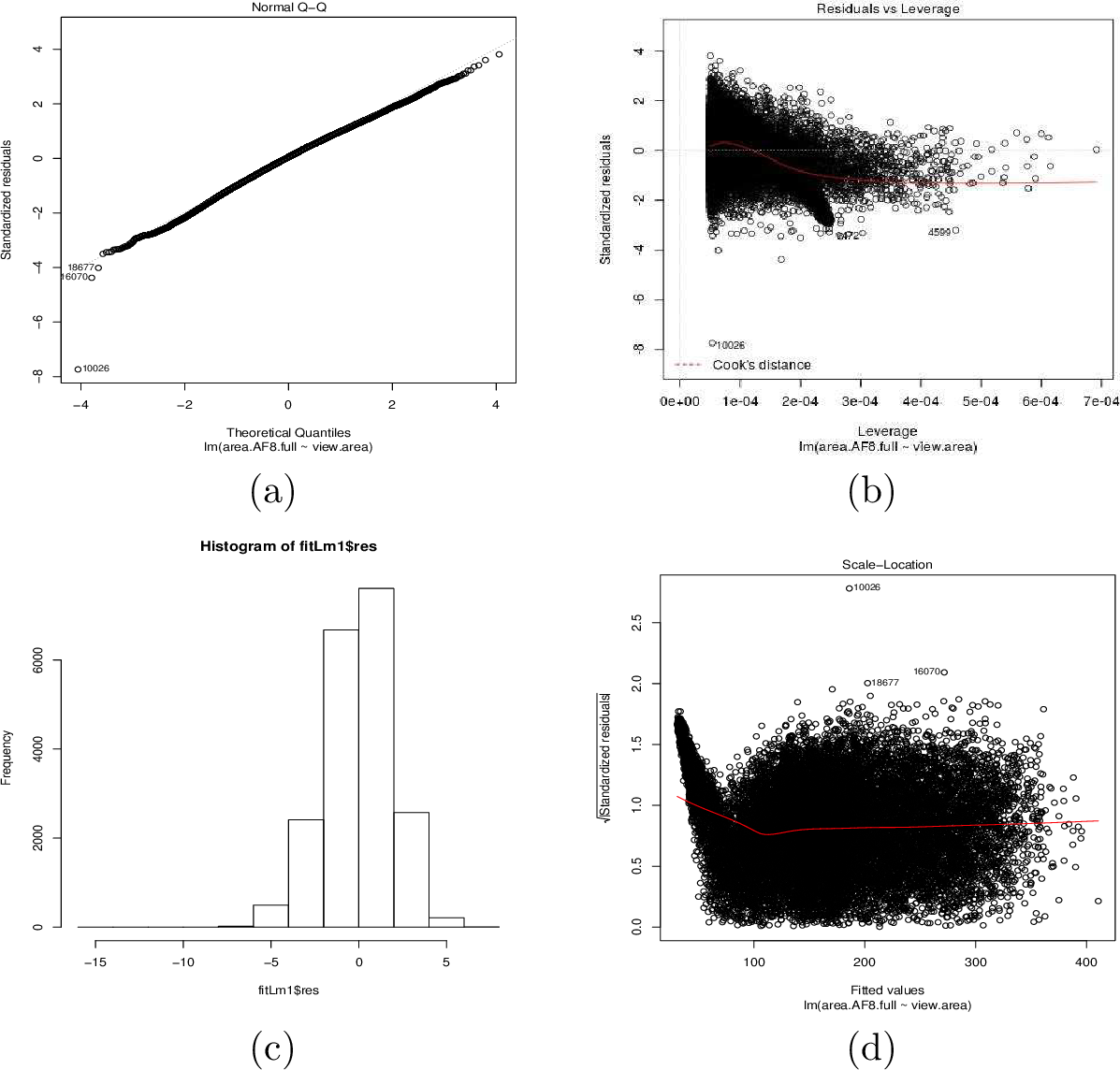

Supplement: S2 Fig — (a) QQ plot of residual. (b) Residuals. (c) Residuals histogram. (d) Residuals scale plot. (TIF) [file pone.0166749.s003.tif]
